# Supplementary material for: A universal O-glycosylation platform enabled by pyridinium catalysis using gas-releasing oxazolidinone-based carbamates donors
Source: Nat Commun. 2025 Dec 4;17:141. doi: 10.1038/s41467-025-66857-8 (PMC12775465; doi:10.1038/s41467-025-66857-8)
Supplement: Supplementary file 1 — Supplementary Information [file 41467_2025_66857_MOESM1_ESM.pdf]

Supplementary Information for

# A Universal *O*-Glycosylation Platform Enabled by Pyridinium Catalysis Using Gas-Releasing Oxazolidinone-Based Carbamates Donors

Xiaoting Qin,[a] Lin Ke,[b] Qinbo Jiao,[b] Wenxu Zhen,[b] Wentao Lin,[b] Jiaxin Luo,[b] Wenyang Chen,[d] Tingbo Liu,[d] Shiping Wang,[a] Chunfa Xu*[b,c]
*Email: xucf@fzu.edu.cn

# Contents


1. General information ........ 3
2. Preparation of the oxazolidinone-based glycosyl carbamate donors ........ 4
   - 2.1 Optimization of glycosyl donor synthesis ........ 4
   - 2.2 General procedures for glycosyl donor synthesis ........ 5
3. Synthesis of Pyridinium Catalyst ........ 15
4. The glycosyl acceptors in this work (2a-2g, 2l-2x, 2ad-2au) ........ 18
5. *O*-Glycosylation using oxazolidinone-based glycosyl carbamate donors ........ 19
   - 5.1 Optimization of *O*-glycosylation ........ 19
   - 5.2 General procedure for *O*-glycosylation ........ 23
   - 5.3 Procedure for gram-scale synthesis ........ 55
6. Comparison of glycosyl donor reactivities ........ 55
7. Synthesis of oligosaccharides 10, 14, 15, 19, 20 ........ 57
8. Mechanistic studies and proposed mechanism ........ 64
   - 8.1 Analysis and recovery of by-products in glycosylation reactions ........ 64
   - 8.2 Analysis of by-products in glycosylation reactions using deuterated catalyst ........ 65
   - 8.3 NMR titration study: catalyst-phenol interaction analysis ........ 67
   - 8.4 Kinetic Isotope Effect (KIE) analysis of glycosylation with MeOH and MeOD ........ 68
   - 8.5 Competition experiment between glycosyl donors 1j and *d*-1j ........ 70
   - 8.6 Study on the stability of glycosyl carbamate donor 1a ........ 74
   - 8.7 Proposed mechanism ........ 75
9. X-ray crystal structure data of compound 1a ........ 75
10. NMR Spectra ........ 77
11. NMR evidence for the high β selectivity ........ 174
12. References ........ 183

# 1. General information

Unless otherwise stated, all reactions were set up under inert atmosphere ($N_2$) utilizing glassware that were oven dried and cooled under nitrogen atmosphere. Silica Gel Flash Column Chromatography was performed on silica gel (particle size 300-400 mesh). Starting materials were purchased directly from commercial suppliers (Sigma Aldrich, Energy Chemical, Bidepharm, Tansoole) and used without further purifications unless otherwise stated. All solvents were dried according to standard procedures or brought from commercial suppliers. Reactions were monitored using thin-layer chromatography (TLC) with F254 indicator. Visualization of the developed plates was performed under UV light (254 nm) or $H_2SO_4$-EtOH (10% $H_2SO_4$ v/v).

$^1$H NMR, $^{19}$F NMR, $^{13}$C NMR and 2D-NMR spectra were recorded using Bruker AVIII 400 and JEOL JNM-ECA500 spectrometer. $^1$H NMR and $^{13}$C NMR chemical shifts were reported in parts per million (ppm) downfield from tetramethylsilane. Coupling constants (*J*) are reported in Hertz (Hz). The residual solvent peak was used as an internal reference: $^1$H NMR ($CDCl_3$ δ 7.26 ppm), $^{13}$C NMR ($CDCl_3$ δ 77.16 ppm), $^1$H NMR (DMSO-$d_6$ δ 2.50 ppm), $^{13}$C NMR (DMSO-$d_6$ δ 39.50 ppm), $^1$H NMR ($CD_3OD$ δ 4.87 ppm), $^{13}$C NMR ($CD_3OD$ δ 49.00 ppm). The following abbreviations were used to explain the multiplicities: s = singlet, d = doublet, t = triplet, q = quartet, m = multiplet, br = broad. Due to residual high-boiling-point solvents, minor peaks are observed at approximately 30 ppm in some $^{13}$C NMR spectra. The IR spectra were recorded using Nicolet iS50 spectrometer. HRMS data was recorded using HRMS Exactive Plus instrument. Optical rotation was measured using MCP 150 instrument.

## 2. Preparation of the oxazolidinone-based glycosyl carbamate donors

### 2.1 Optimization of glycosyl donor synthesis

Procedure: To a solution of bis(trichloromethyl) carbonate (0.33 mmol, 1.0 equiv.) and oxazolidinone (0.5 mmol, 1.5 equiv.) in anhydrous $CH_2Cl_2$ (0.1 M) was added $Et_3N$ (1.2 mmol, 3.6 equiv.) at 0 °C under nitrogen atmosphere. After stirring at 0 °C for 2 h followed by 2 h at room temperature, a solution of compound **S1** (0.33 mmol, 1.0 equiv.) in $CH_2Cl_2$ and base (0.66 mmol, 2.0 equiv.) was added dropwise to the mixture under nitrogen atmosphere. The reaction mixture was stirred at room temperature until completion (monitored by TLC, 4 h), then diluted with $CH_2Cl_2$ (10 mL) and washed with distilled water (15 mL) and brine (15 mL). The organic layer was dried over $Na_2SO_4$, filtered, and concentrated in vacuo. The residue was purified by flash column chromatography using petroleum ether/ethyl acetate (1:1) as eluent on silica gel to afford the glycosyl carbamate donors.

**Supplementary Table S1. Optimization of glycosyl donor synthesis**

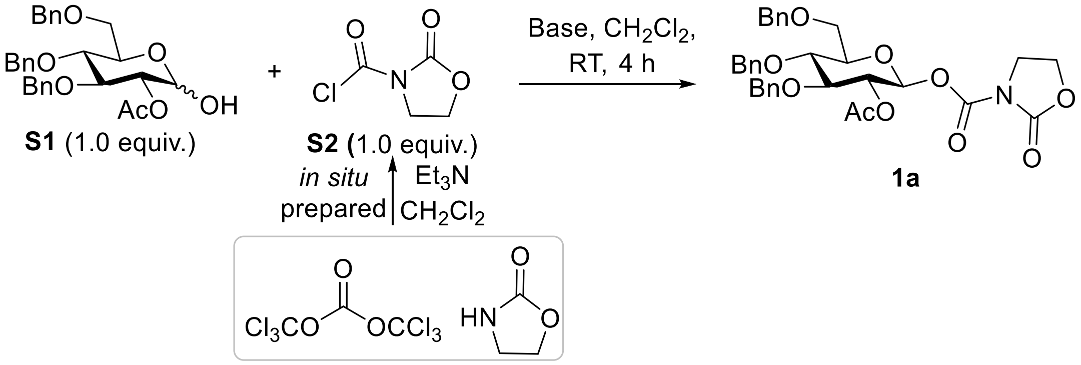


| Entry[a] | Base | Yield% | α/β ratio[b] |
|---|---|---|---|
| 1 | $Et_3N$ | 59 | 1 : 1 |
| 2 | DIPEA | 78 | 1 : 56 |
| 3 | $K_2CO_3$ | 60 | 1 : 20 |
| 4 | NaH | 50 | 1 : 22 |
| 5 | DBU | 55 | 2.6 : 1 |

[a]Reaction condition: **S1** (0.33 mmol, 1.0 equiv.), **S2** (0.33 mmol, 1.0 equiv.), base (0.66 mmol, 2.0 equiv.), $CH_2Cl_2$ (3.3 mL, 0.1 M) at room temperature for 4 h; isolated yields. [b]α/β ratio was determined by crude $^1$H NMR spectrum.

## 2.2 General procedures for glycosyl donor synthesis

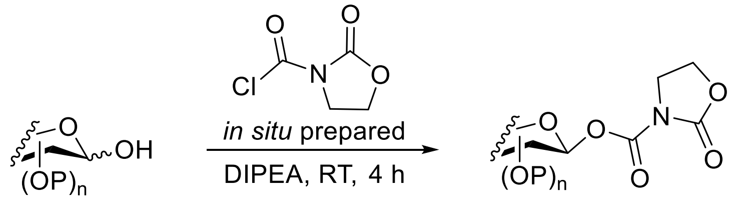


**hemiacetal** **1a-k**

**Supplementary Figure S1. Preparation of the glycosyl carbamate donors 1a-k.**

Hemiacetal was synthesized from glycosyl halides according to the reported literature[1].

**General procedure A:** To a solution of bis(trichloromethyl) carbonate (1.0 equiv.) and oxazolidinone (1.5 equiv.) in anhydrous $CH_2Cl_2$ (0.1 M) was added $Et_3N$ (3.6 equiv.) at 0 °C under nitrogen atmosphere. After stirring at 0 °C for 2 h followed by 2 h at room temperature, a solution of hemiacetal (1.0 equiv.) in $CH_2Cl_2$, DIPEA (2.0 equiv.) was added dropwise to the mixture under nitrogen atmosphere. The reaction mixture was stirred at room temperature until completion (monitored by TLC, 4 h), then diluted with $CH_2Cl_2$ and washed with water and brine. The organic layer was dried over $Na_2SO_4$, filtered, and concentrated in vacuo. The residue was purified by flash column chromatography using petroleum ether/ethyl acetate (1:1) as eluent on silica gel to afford the glycosyl carbamate donors.

**General procedure B:** To a solution of acyl chloride **S2**[2] was added hemiacetal (1.0 equiv.) in $CH_2Cl_2$, DBU (2.0 equiv.) under nitrogen atmosphere. Then the resulting mixture was stirred at room temperature upon completion determined by TLC analysis. Then, the mixture was diluted with $CH_2Cl_2$, and washed with water and brine. The organic layer was dried over $Na_2SO_4$, filtered, and concentrated in vacuo. The residue was purified by flash column chromatography using petroleum ether/ethyl acetate (1:1) as eluent on silica gel to afford the glycosyl carbamate donors.

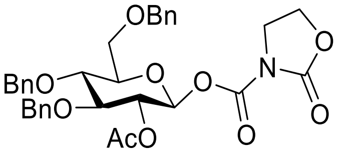


**1a**

**(2*S*,3*R*,4*S*,5*R*,6*R*)-3-Acetoxy-4,5-bis(benzyloxy)-6-((benzyloxy)methyl)tetrahydro-2*H*-pyran-2-yl 2-oxooxazolidine-3-carboxylate**

Compound **1a** was prepared from **S1** (6.2019 g, 12.6 mmol) according to **General procedure A**. The crude product was purified by silica gel column chromatography (petroleum ether/ethyl acetate = 1:1) to afford **1a** (4.9568 g, 65% yield) as a light yellow

solid.

**$^1$H NMR (400 MHz, $CDCl_3$)** δ 7.27 – 7.21 (m, 13H), 7.10 – 7.09 (m, 2H), 5.58 – 5.54 (m, 1H), 5.10 – 5.04 (m, 1H), 4.76 – 4.71 (m, 2H), 4.65 – 4.59 (m, 1H), 4.57 – 4.46 (m, 2H), 4.44 – 4.38 (m, 1H), 4.25 – 4.21 (m, 2H), 3.98 – 3.91 (m, 1H), 3.84 – 3.77 (m, 2H), 3.73 – 3.65 (m, 3H), 3.58 (d, *J* = 7.4 Hz, 1H), 1.89 (s, 3H).

**$^{13}$C NMR (101 MHz, $CDCl_3$)** δ 170.0, 152.2, 148.9, 138.4, 138.2, 138.2, 128.9, 128.9, 128.3, 128.3, 128.2, 128.2, 94.6, 82.8, 77.3, 76.1, 75.5, 75.4, 74.0, 72.5, 68.4, 62.2, 43.7, 21.3.

**$[\alpha]_D^{25}$** = +5.1 (c = 0.47, $CHCl_3$).

**HRMS** (ESI-TOF): calculated for $C_{33}H_{35}NO_{10}Na^+$ [M+Na$^+$]: 628.2153, found 628.2146.

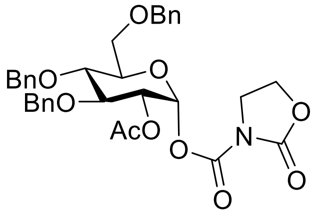


**1a-α**

**(2*R*,3*R*,4*S*,5*R*,6*R*)-3-Acetoxy-4,5-bis(benzyloxy)-6-((benzyloxy)methyl)tetrahydro-2*H*-pyran-2-yl 2-oxooxazolidine-3-carboxylate**

Compound **1a-α** was prepared according to **General procedure B** using hemiacetal (1.6243 g, 3.3 mmol) as starting material. The crude mixture was purified by silica gel column chromatography (petroleum ether/ethyl acetate 1.5:1) to afford **1a-α** (739 mg, 37% yield) as a light yellow oil, and **1a-β** (280 mg, 14% yield) as a light yellow solid.

**$^1$H NMR (400 MHz, $CDCl_3$)** δ 7.32 – 7.24 (m, 13H), 7.17 – 7.15 (m, 2H), 6.35 (d, *J* = 3.5 Hz, 1H), 5.08 – 5.05 (m, 1H), 4.83 – 4.79 (m, 2H), 4.71 (d, *J* = 11.4 Hz, 1H), 4.59 – 4.46 (m, 3H), 4.40 – 4.36 (m, 2H), 4.10 – 3.97 (m, 4H), 3.83 – 3.72 (m, 2H), 3.64 (d, *J* = 11.1 Hz, 1H), 1.95 (s, 3H).

**$^{13}$C NMR (101 MHz, $CDCl_3$)** δ 170.1, 151.3, 149.8, 138.5, 138.1, 137.9, 128.5, 128.5, 128.5, 128.0, 128.0, 127.9, 127.8, 127.8, 127.8, 93.3, 79.7, 75.6, 75.2, 73.9, 73.6, 72.0, 68.1, 61.8, 43.2, 20.8.

**$[\alpha]_D^{25}$** = +6.8 (c = 0.62, $CHCl_3$).

**HRMS** (ESI-TOF): calculated for $C_{33}H_{35}NO_{10}Na^+$ [M+Na$^+$]: 628.2153, found 628.2144.

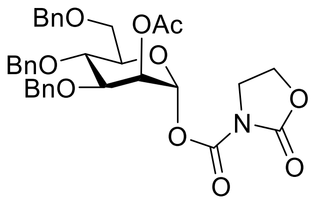

**1b**

**(2*R*,3*S*,4*S*,5*R*,6*R*)-3-Acetoxy-4,5-bis(benzyloxy)-6-((benzyloxy)methyl)tetrahydro-2*H*-pyran-2-yl 2-oxooxazolidine-3-carboxylate**

Compound **1b** was prepared according to **General procedure A** using hemiacetal (1.6243 g, 3.3 mmol) as starting material. The crude product was purified by silica gel column chromatography (petroleum ether/ethyl acetate = 1:1) to afford **1b** (1.3581 g, 68% yield) as a light yellow solid.

**$^{1}$H NMR (400 MHz, CDCl$_3$)** δ 7.33 – 7.23 (m, 13H), 7.16 – 7.14 (m, 2H), 5.76 (s, 1H), 5.62 (d, *J* = 3.3 Hz, 1H), 4.84 (d, *J* = 10.8 Hz, 1H), 4.69 (d, *J* = 11.3 Hz, 1H), 4.62 (d, *J* = 12.0 Hz, 1H), 4.53 – 4.47 (m, 3H), 4.33 – 4.29 (m, 2H), 3.96 – 3.87 (m, 3H), 3.77–3.72 (m, 3H), 3.61–3.57 (m, 1H), 2.20 (s, 3H).

**$^{13}$C NMR (101 MHz, CDCl$_3$)** δ 170.9, 151.3, 148.9, 138.2, 137.4, 128.6, 128.5, 128.5, 128.2, 128.1, 128.0, 127.9, 127.8, 93.2, 79.4, 76.4, 75.2, 73.8, 73.6, 71.8, 68.6, 67.2, 61.8, 43.2, 21.1.

**$[\alpha]_D^{25}$** = -27.2 (c = 0.36, CHCl$_3$).

**HRMS** (ESI-TOF): calculated for $C_{33}H_{35}NO_{10}Na^+$ [M+Na$^+$]: 628.2153, found 628.2149.

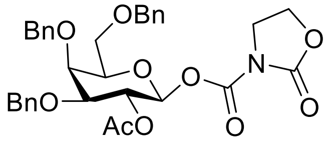

**1c**

**(2*S*,3*R*,4*S*,5*S*,6*R*)-3-Acetoxy-4,5-bis(benzyloxy)-6-((benzyloxy)methyl)tetrahydro-2*H*-pyran-2-yl 2-oxooxazolidine-3-carboxylate**

Compound **1c** was prepared according to **General procedure A** using hemiacetal (1.6243 g, 3.3 mmol) as starting material. The crude product was purified by silica gel column chromatography (petroleum ether/ethyl acetate = 1:1) to afford **1c** (1.2982 g, 65% yield) as a white solid.

**$^{1}$H NMR (400 MHz, CDCl$_3$)** δ 7.34 – 7.24 (m, 15H), 5.57 (d, *J* = 8.1 Hz, 1H), 5.48 – 5.43 (m, 1H), 4.91 (d, *J* = 11.3 Hz, 1H), 4.68 (d, *J* = 12.1 Hz, 1H), 4.60 – 4.52 (m, 2H), 4.48 – 4.40 (m, 2H), 4.29 – 4.25 (m, 2H), 4.01 – 3.96 (m, 2H), 3.88 – 3.82 (m, 1H), 3.75 (t, *J* = 6.4 Hz, 1H), 3.66 – 3.57 (m, 3H), 2.01 (s, 3H).

**$^{13}$C NMR (101 MHz, CDCl$_3$)** δ 169.8, 152.0, 148.5, 138.2, 137.7, 128.6, 128.5,

128.4, 128.0, 128.0, 128.0, 127.9, 127.6, 94.6, 79.7, 74.9, 74.5, 73.6, 72.5, 72.4, 70.3, 67.8, 61.9, 43.4, 21.0.

**[α]$_D$$^{25}$** = +3.33 (c = 0.3, $CHCl_3$).

**HRMS** (ESI-TOF): calculated for $C_{33}H_{35}NO_{10}Na^+$ [M+$Na^+$]: 628.2153, found 628.2136.

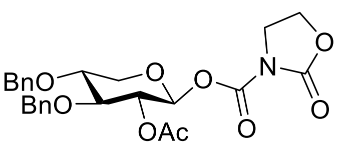


**1d**

**(2*S*,3*R*,4*S*,5*R*)-3-Acetoxy-4,5-bis(benzyloxy)tetrahydro-2*H*-pyran-2-yl 2-oxooxazolidine-3-carboxylate**

Compound **1d** was prepared according to **General procedure A** using hemiacetal (1.2281 g, 3.3 mmol) as starting material. The crude product was purified by silica gel column chromatography (petroleum ether/ethyl acetate = 2:1) to afford **1d** (1.0087 g, 63% yield) as a white solid.

**$^1$H NMR (400 MHz, $CDCl_3$)** δ 7.29 (m, 10H), 5.67 (d, *J* = 6.4 Hz, 1H), 5.03 (t, *J* = 6.8 Hz, 1H), 4.78 (d, *J* = 11.5 Hz, 1H), 4.69 – 4.58 (m, 3H), 4.27 – 4.22 (m, 2H), 4.06 – 4.02 (m, 1H), 3.92 – 3.86 (m, 1H), 3.83 – 3.77 (m, 1H), 3.72 – 3.64 (m, 2H), 3.54 – 3.49 (m, 1H), 1.97 (s, 3H).

**$^{13}$C NMR (101 MHz, $CDCl_3$)** δ 169.8, 151.7, 148.7, 138.0, 137.7, 128.6, 128.5, 128.1, 127.9, 127.9, 94.8, 78.7, 76.1, 74.4, 72.9, 70.3, 63.7, 61.8, 43.3, 20.9.

**[α]$_D$$^{25}$** = -17.9 (c = 0.48, $CHCl_3$).

**HRMS** (ESI-TOF): calculated for $C_{25}H_{27}NO_9Na^+$ [M+$Na^+$]: 508.1578, found 508.1574.

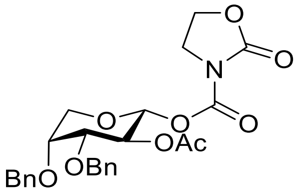


**1e**

**(2*R*,3*S*,4*R*,5*R*)-3-Acetoxy-4,5-bis(benzyloxy)tetrahydro-2*H*-pyran-2-yl 2-oxooxazolidine-3-carboxylate**

Compound **1e** was prepared according to **General procedure A** using hemiacetal (1.2281 g, 3.3 mmol) as starting material. The crude product was purified by silica gel column chromatography (petroleum ether/ethyl acetate = 1:1) to afford **1e** (944.6 mg, 59% yield) as a light yellow solid.

**$^1$H NMR (400 MHz, $CDCl_3$)** δ 7.35 – 7.26 (m, 10H), 5.73 (d, *J* = 3.9 Hz, 1H), 5.26 (t, *J* = 4.7 Hz, 1H), 4.71 (d, *J* = 11.5 Hz, 1H), 4.61 – 4.57 (m, 3H), 4.14 – 4.02 (m, 3H), 3.83 – 3.80 (m, 2H), 3.75 – 3.68 (m, 1H), 3.65 – 3.56 (m, 2H), 2.05 (s, 3H).

**$^{13}$C NMR (101 MHz, $CDCl_3$)** δ 169.4, 151.9, 148.3, 137.8, 137.7, 128.4, 128.3, 128.0, 127.8, 127.6, 93.3, 74.4, 72.5, 71.4, 71.3, 68.7, 61.6, 61.1, 43.0, 20.8.

**$[\alpha]_D^{25}$** = +30.3 (c = 0.84, $CHCl_3$).

**HRMS** (ESI-TOF): calculated for $C_{25}H_{27}NO_9Na^+$ [M+Na$^+$]: 508.1578, found 508.1573.

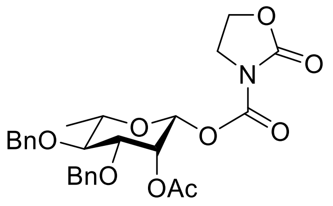


**1f**

**(2*S*,3*R*,4*R*,5*S*,6*S*)-3-Acetoxy-4,5-bis(benzyloxy)-6-methyltetrahydro-2*H*-pyran-2-yl 2-oxooxazolidine-3-carboxylate**

Compound **1f** was prepared according to **General procedure A** using hemiacetal[3] (1.3272 g, 3.3 mmol) as starting material. The crude product was purified by silica gel column chromatography (petroleum ether/ethyl acetate = 1:1) to afford **1f** (988.4 mg, 60% yield) as a white solid.

**$^1$H NMR (400 MHz, $CDCl_3$)** δ 7.35 – 7.26 (m, 10H), 5.76 (d, *J* = 1.2 Hz, 1H), 5.66 – 5.64 (m, 1H), 4.93 (d, *J* = 10.9 Hz, 1H), 4.72 (d, *J* = 11.4 Hz, 1H), 4.64 (d, *J* = 10.9 Hz, 1H), 4.54 (d, *J* = 11.4 Hz, 1H), 4.37 – 4.33 (m, 2H), 4.03 – 3.92 (m, 2H), 3.73 – 3.70 (m, 1H), 3.56 – 3.46 (m, 2H), 2.23 (s, 3H), 1.38 (d, *J* = 5.8 Hz, 3H).

**$^{13}$C NMR (101 MHz, $CDCl_3$)** δ 170.9, 151.2, 149.0, 138.2, 137.4, 128.7, 128.6, 128.3, 128.1, 128.0, 93.0, 79.4, 79.2, 75.6, 72.9, 71.8, 67.4, 61.8, 43.2, 21.1, 17.9.

**$[\alpha]_D^{25}$** = +1.4 (c = 0.25, $CHCl_3$).

**HRMS** (ESI-TOF): calculated for $C_{26}H_{29}NO_9Na^+$ [M+Na$^+$]: 522.1735, found 522.1725.

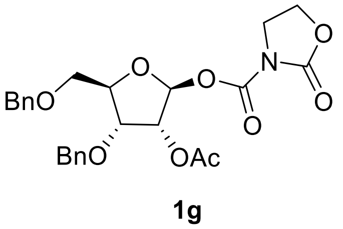


**1g**

**(2*S*,3*R*,4*R*,5*R*)-3-Acetoxy-4-(benzyloxy)-5-((benzyloxy)methyl)tetrahydrofuran-2-**

**yl 2-oxooxazolidine-3-carboxylate**

Compound **1g** was prepared according to **General procedure A** using hemiacetal[4] (1.2281 g, 3.3 mmol) as starting material. The crude product was purified by silica gel column chromatography (petroleum ether/ethyl acetate = 2:1) to afford **1g** (1.0887 g, 68% yield) as a white solid.

**$^1$H NMR (400 MHz, CDCl$_3$)** δ 7.38 – 7.24 (m, 10H), 6.20 (s, 1H), 5.40 (d, *J* = 4.3 Hz, 1H), 4.65 (d, *J* = 11.5 Hz, 1H), 4.50 – 4.45 (m, 3H), 4.42– 4.39 (m, 1H), 4.30 – 4.28 (m, 1H), 4.12 – 4.06 (m, 1H), 4.01 – 3.95 (m, 1H), 3.77 – 3.74 (m, 1H), 3.70 – 3.53 (m, 3H), 2.16 (s, 3H).

**$^{13}$C NMR (101 MHz, CDCl$_3$)** δ 169.9, 151.4, 149.0, 138.2, 137.4, 128.6, 128.4, 128.2, 128.2, 127.8, 127.7, 100.5, 82.0, 76.1, 73.4, 73.4, 69.2, 61.5, 42.9, 20.8.

**$[\alpha]_D^{25}$** = +36.8 (c = 0.50, CHCl$_3$).

**HRMS** (ESI-TOF): calculated for $C_{25}H_{27}NO_9Na^+$ [M+Na$^+$]: 508.1578, found 508.1575.

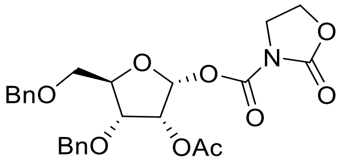


**1g-α**

**(2*R*,3*R*,4*R*,5*R*)-3-Acetoxy-4-(benzyloxy)-5-((benzyloxy)methyl)tetrahydrofuran-2-yl 2-oxooxazolidine-3-carboxylate**

Compound **1g-α** was prepared according to **General procedure B** using hemiacetal (0.3722 g, 1 mmol) as starting material. The crude product was purified by silica gel column chromatography (petroleum ether/ethyl acetate = 2:1) to afford **1g-α** (237.8 mg, 49% yield, α/β = 4:1) as a syrup.

**$^1$H NMR (400 MHz, CDCl$_3$)** δ 7.37 – 7.26 (m, 10H), 6.44 (d, *J* = 4.3 Hz, 1H), 5.17 – 5.15 (m, 1H), 4.60 – 4.55 (m, 2H), 4.50 – 4.47 (m, 2H), 4.43 – 4.40 (m, 1H), 4.29 – 4.18 (m, 3H), 3.92 – 3.82 (m, 2H), 3.58 – 3.48 (m, 2H), 2.14 (s, 3H).

**$^{13}$C NMR (101 MHz, CDCl$_3$)** δ 170.2, 151.8, 149.4, 137.9, 137.8, 128.6, 128.5, 128.1, 128.1, 128.0, 127.8, 97.1, 84.5, 75.7, 73.7, 73.3, 72.0, 69.3, 61.7, 43.4, 20.8.

**$[\alpha]_D^{25}$** = +8.5 (c = 0.34, CHCl$_3$).

**HRMS** (ESI-TOF): calculated for $C_{25}H_{27}NO_9Na^+$ [M+Na$^+$]: 508.1578, found 508.1570.

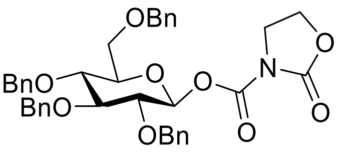

**1h**

**(2*S*,3*R*,4*S*,5*R*,6*R*)-3,4,5-Tris(benzyloxy)-6-((benzyloxy)methyl)tetrahydro-2*H*-pyran-2-yl 2-oxooxazolidine-3-carboxylate**

Compound **1h** was prepared according to **General procedure A** using hemiacetal (1.7828 g, 3.3 mmol) as starting material. Purification of crude product by recrystallization using petroleum ether and dichloromethane to afford **1h** (1.7677 g, 82% yield) as a white solid.

**$^{1}$H NMR (400 MHz, $CDCl_3$)** δ 7.36 – 7.30 (m, 18H), 7.19 – 7.17 (m, 2H), 5.69 (d, *J* = 7.5 Hz, 1H), 4.94 (d, *J* = 10.9 Hz, 1H), 4.88 – 4.84 (m, 4H), 4.64 (d, *J* = 12.0 Hz, 1H), 4.58 (d, *J* = 10.9 Hz, 1H), 4.51 (d, *J* = 12.0 Hz, 1H), 4.28 – 4.23 (m, 2H), 3.92 – 3.86 (m, 1H), 3.82 – 3.64 (m, 7H).

**$^{13}$C NMR (101 MHz, $CDCl_3$)** δ 151.3, 149.5, 138.4, 138.4, 138.1, 137.9, 128.5, 128.5, 128.4, 128.0, 127.9, 127.9, 127.8, 127.8, 127.8, 127.7, 96.4, 84.6, 80.9, 77.0, 75.6, 75.6, 75.0, 74.8, 73.6, 68.3, 61.6, 43.1.

**$[\alpha]_D^{25}$** = +2.3 (c = 0.13, $CHCl_3$).

**HRMS** (ESI-TOF): calculated for $C_{38}H_{39}NO_9Na^+$ [M+Na$^+$]: 676.2517, found 676.2505.

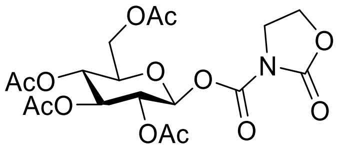

**1i**

**(2*R*,3*R*,4*S*,5*R*,6*S*)-2-(acetoxymethyl)-6-((2-oxooxazolidine-3-carbonyl)oxy)tetrahydro-2*H*-pyran-3,4,5-triyl triacetate**

Compound **1i** was prepared according to **General procedure A** using hemiacetal (1.1487 g, 3.3 mmol) as starting material. The crude product was purified by silica gel column chromatography (petroleum ether/ethyl acetate = 1:2) to afford **1i** (715.2 mg, 47% yield) as a white solid.

**$^{1}$H NMR (400 MHz, $CDCl_3$)** δ 5.72 (d, *J* = 7.7 Hz, 1H), 5.29 – 5.22 (m, 2H), 5.14 (t, *J* = 9.5 Hz, 1H), 4.32 – 4.25 (m, 1H), 4.13 – 4.06 (m, 2H), 4.00 – 3.84 (m, 2H), 3.72 – 3.53 (m, 2H), 2.08 (s, 3H), 2.04 (s, 3H), 2.03 (s, 3H), 2.02 (s, 3H).

**$^{13}$C NMR (101 MHz, $CDCl_3$)** δ 170.7, 170.2, 169.8, 169.5, 169.3, 150.6, 94.3, 77.4, 77.2, 73.0, 72.6, 69.9, 67.8, 61.5, 20.9, 20.8, 20.8, 20.7.

**$[\alpha]_D^{25}$** = -6.9 (c = 0.42, $CHCl_3$).

**HRMS** (ESI-TOF): calculated for $C_{18}H_{23}NO_{13}Na^+$ [M+Na$^+$]: 484.1062, found 484.1054.

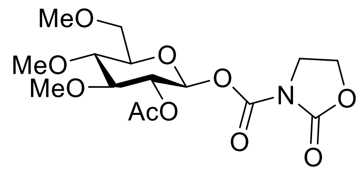

**1j**

**(2*S*,3*R*,4*S*,5*R*,6*R*)-3-Acetoxy-4,5-dimethoxy-6-(methoxymethyl)tetrahydro-2*H*-pyran-2-yl 2-oxooxazolidine-3-carboxylate**

Compound **1l** was prepared according to **General procedure A** using hemiacetal (0.5282 g, 2 mmol) as starting material. The crude product was purified by silica gel column chromatography (petroleum ether/ethyl acetate = 1:1) to afford **1j** (452.6 mg, 60% yield) as a white solid.

**$^1$H NMR (400 MHz, CDCl$_3$)** δ 5.55 (d, *J* = 8.1 Hz, 1H), 4.99 (t, *J* = 8.2 Hz, 1H), 4.35 – 4.31 (m, 2H), 4.04 (q, *J* = 8.7 Hz, 1H), 3.88 (q, *J* = 8.8 Hz, 1H), 3.64 – 3.59 (m, 2H), 3.54 (s, 3H), 3.52 (s, 3H), 3.48 – 3.45 (m, 1H), 3.41 – 3.34 (m, 5H), 2.08 (s, 3H).

**$^{13}$C NMR (101 MHz, CDCl$_3$)** δ 169.7, 151.9, 148.6, 94.2, 84.2, 78.4, 75.6, 72.0, 70.3, 61.9, 60.7, 60.6, 59.4, 43.4, 21.0.

**$[\alpha]_D^{25}$** = +4.5 (c = 0.20, $CHCl_3$).

**HRMS** (ESI-TOF): calculated for $C_{15}H_{23}NO_{10}Na^+$ [M+Na$^+$]: 400.1214, found 400.1210.

**(2*S*,3*R*,4*S*,5*R*,6*R*)-3-Acetoxy-4,5-dimethoxy-6-(methoxymethyl)tetrahydro-2*H*-pyran-2-yl-2-d 2-oxooxazolidine-3-carboxylate (*d*-1j)**

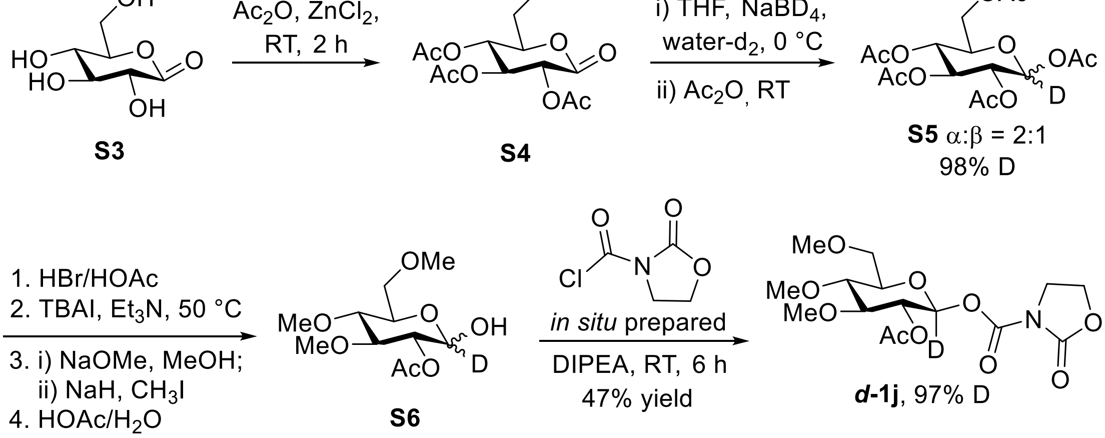


**Supplementary Figure S2. Synthesis of the glycosyl donor *d*-1j.**

**S3** D-Glucono-1,5-lactone (2.1366 g, 12 mmol) was added to a stirred solution of $Ac_2O$ (10 mL) containing $ZnCl_2$ (1 g). After 2 h the reaction mixture was poured onto

crushed ice (10.0000 g) and this mixture was stirred for 30 min. The resulting solution was extracted with $CH_2Cl_2$ (3 x 10 mL) and the organic layer was washed successively with $H_2O$ (10 mL), saturated $NaHCO_3$ (10 mL), saturated NaCl (10 mL) and dried over anhydrous $Na_2SO_4$. The filtered solution was concentrated to yield a thick syrup. The crude product was purified by silica gel column chromatography (petroleum ether/ethyl acetate = 2:1) to afford **S4** (3.6962 g, 89% yield) as a colorless syrup.

A cooled solution of $NaBD_4$ (200.0 mg, 4.8 mmol) in $D_2O$ (1 mL, 0 °C) was added dropwise to a solution of Compound **S4** (3.6962 g, 10.7 mmol) in THF (30 mL) at 0 °C and the resulting mixture was stirred for 2 h at 0 °C. Following dropwise addition of cooled acetic anhydride (5 mL, 0 °C) to the reaction mixture the solution was stirred overnight at ambient temperature. The reaction mixture was poured into cold saturated $NaHCO_3$ (10 mL) and stirred for 30 min. The resulting solution was extracted with $CH_2Cl_2$ (3 x 250 mL) and the organic solution was washed successively with $H_2O$ (250 mL), saturated NaCl (250 mL) and dried over anhydrous $Na_2SO_4$. The filtered solution was concentrated. The crude product was purified by silica gel column chromatography (petroleum ether/ethyl acetate = 1:1) to afford **S5** (3.1806 g, 76% yield, 98% D) as a white solid. Compound **S5** was synthesized following the reported literature[6].

Compound **S6** was synthesized from **S5** according to the reported literature[1].

Compound ***d*-1j** was prepared from **S6** (530.3 mg, 2 mmol) according to **General procedure A**. The crude product was purified by silica gel column chromatography (petroleum ether/ethyl acetate = 1:1) to afford ***d*-1j** (452.6 mg, 60% yield, 97% D) as a white solid.

**$^1$H NMR (400 MHz, $CDCl_3$)** δ 5.55 (d, $J$ = 8.1 Hz, 0.03H, 97% D), 5.00 (d, $J$ = 8.9 Hz, 1H), 4.33 (t, $J$ = 8.0 Hz, 2H), 4.07 – 4.01 (m, 1H), 3.91 – 3.85 (m, 1H), 3.64 – 3.59 (m, 2H), 3.54 (s, 3H), 3.53 (s, 3H), 3.48 – 3.45 (m, 1H), 3.41 – 3.34 (m, 5H), 2.08 (s, 3H).

**$^{13}$C NMR (101 MHz, $CDCl_3$)** δ 169.8, 151.9, 148.6, 94.1 (t, $J_{C,D}$ = 25.3 Hz, C-1), 84.2, 78.4, 75.6, 71.9, 70.3, 61.9, 60.7, 60.6, 59.4, 43.4, 21.1.

**$[\alpha]_D^{25}$** = +4.8 (c = 0.21, $CHCl_3$).

**HRMS** (ESI-TOF): calculated for $C_{15}H_{22}DNO_{10}Na^+$ [M+$Na^+$]: 402.1355, found 402.1304.

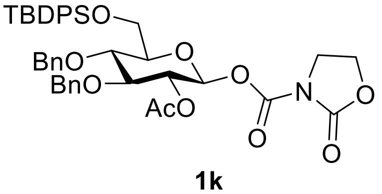


**1k**

**(2*S*,3*R*,4*S*,5*R*,6*R*)-3-Acetoxy-4,5-bis(benzyloxy)-6-(((tert-butyldiphenylsilyl)oxy)methyl)tetrahydro-2H-pyran-2-yl 2-oxooxazolidine-3-carboxylate**

Compound **1k** was prepared according to **General procedure A** with 4.0 equiv. DIPEA using hemiacetal[5] (1.2808 g, 2 mmol) as starting material. The crude product was purified by silica gel column chromatography (petroleum ether/ethyl acetate = 3:1) to afford **1k** (979.2 mg, 65% yield) as a light yellow solid.

**$^1$H NMR (400 MHz, $CDCl_3$)** δ 7.68 (dd, *J* = 10.9, 7.5 Hz, 4H), 7.44 – 7.41 (m, 2H), 7.37 – 7.31 (m, 12H), 7.22 – 7.20 (m, 2H), 5.68 (d, *J* = 7.8 Hz, 1H), 5.15 (t, *J* = 8.4 Hz, 1H), 4.90 – 4.84 (m, 2H), 4.76 – 4.71 (m, 2H), 4.36 – 4.33 (m, 2H), 4.02 – 3.91 (m, 5H), 3.79 (t, *J* = 8.9 Hz, 1H), 3.56 (d, *J* = 9.6 Hz, 1H), 1.99 (s, 3H), 1.08 (s, 9H).

**$^{13}$C NMR (101 MHz, $CDCl_3$)** δ 169.8, 151.8, 148.5, 138.0, 137.9, 135.9, 135.7, 133.4, 133.1, 129.9, 129.8, 128.6, 128.6, 128.0, 128.0, 128.0, 127.9, 127.8, 127.7, 94.3, 82.4, 77.0, 76.5, 75.3, 75.2, 72.2, 62.1, 61.8, 43.4, 26.9, 21.0, 19.5.

**$[\alpha]_D^{25}$** = -157.0 (c = 0.50, $CHCl_3$).

**HRMS** (ESI-TOF): calculated for $C_{42}H_{47}NO_{10}SiNa^+$ [M+Na$^+$]: 776.2861, found 776.2850.

## 3. Synthesis of Pyridinium Catalyst

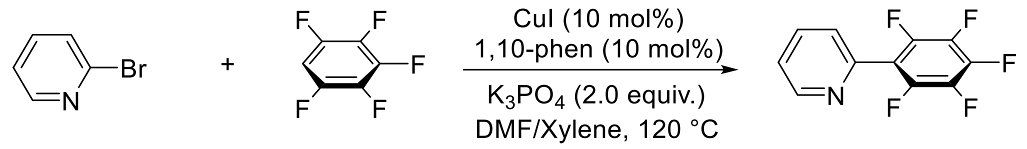


**S7** (10 mmol) **S8** (2.0 equiv.) **S9** (85%, 2.0827 g)

**Supplementary Figure S3. Synthesis of 2-Pentafluorophenylpyridine.**

2-Pentafluorophenylpyridine was synthesized via a copper-catalyzed coupling between 2-bromopyridine and pentafluorobenzene (10 mol% CuI), affording the product in 85% yield and gram-scale quantities according to the reported literature[7].

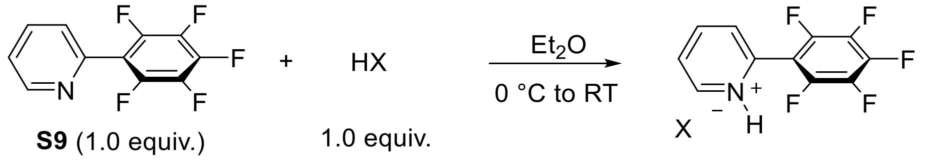


**S9** (1.0 equiv.) 1.0 equiv.

**Supplementary Figure S4. Synthesis of 2-Pentafluorophenyl pyridinium salt.**

**General procedure C.** To a solution of the 2-Pentafluorophenylpyridine (2 mmol, 1.0 equiv.) in dry $Et_2O$ (8 mL) was added a solution of corresponding acid HX (X = $BF_4$, Br, OTf) (2 mmol, 1.0 equiv.) at 0 °C. Upon completion of addition, the mixture was allowed to stir at room temperature for 10 min and form a precipitation. The precipitation was collected by filtration and washed twice with $Et_2O$, and further dried under vacuum to give the product.

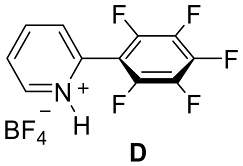

**D**

The title compound was synthesized according to general procedure **C** using 2-pentafluorophenylpyridine (2.0 mmol, 490.0 mg), dry $Et_2O$ (8 mL) and $HBF_4$ ether solution (54 wt%, 0.54 mL, 2 mmol, 1.0 equiv.) as reaction reagent. The precipitation was collected by filtration and washed twice with $Et_2O$, and further dried under vacuum to give the product **D** as a white solid (599.6 mg, 90%). The pyridinium salt was kept in nitrogen atmosphere.

**$^1$H NMR (400 MHz, DMSO-*d*$_6$)** δ 12.25 (s, 1H), 8.87 (d, *J* = 5.2 Hz, 1H), 8.28 (t, *J* = 8.0 Hz, 1H), 7.87 (d, *J* = 8.0 Hz, 1H), 7.77 (t, *J* = 6.5 Hz, 1H).

**$^{13}$C NMR (101 MHz, DMSO-*d*$_6$)** δ 148.3, 146.0-143.1 (m), 143.5, 141.7, 141.8-140.6 (m), 139.1-136.4 (m), 128.1, 126.4, 112.9-112.4 (m).

**$^{19}$F NMR (376 MHz, DMSO-*d*$_6$)** δ -142.7 (dd, *J* = 23.7, 7.7 Hz), -148.5, -152.7 (t,

*J* = 21.8 Hz), -162.3 (td, *J* = 22.9, 7.6 Hz).

**ESI-HRMS:** Calculated for $C_{11}H_5F_5N$ $(M-BF_4^-)^+$: 246.0337, Found: 246.0336.

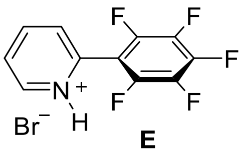

**E**

The title compound was synthesized according to general procedure **C** using 2-pentafluorophenylpyridine (2.0 mmol, 490.0 mg), dry $Et_2O$ (8 mL) and HBr aqueous solution (48 wt%, 230 μL, 2.0 mmol) as reaction reagent. Then the solvent was removed and the residue was recrystallized in $CH_2Cl_2/Et_2O$ to give the product **E** as a white solid (578.4 mg, 89%). The pyridinium salt was kept in nitrogen atmosphere.

**$^1$H NMR (400 MHz, DMSO-*d*$_6$)** δ 8.80 (d, *J* = 5.3 Hz, 1H), 8.20 (s, 1H), 8.09 (td, *J* = 7.8, 1.8 Hz, 1H), 7.73 (d, *J* = 7.9 Hz, 1H), 7.61 (dd, *J* = 7.7, 4.9 Hz, 1H).

**$^{13}$C NMR (101 MHz, DMSO-*d*$_6$)** δ 149.6, 145.2, 145.3-142.6 (m), 142.2-139.18 (m), 138.3, 138.5-135.8 (m), 126.5, 124.8, 114.7-114.2 (m).

**$^{19}$F NMR (376 MHz, DMSO-*d*$_6$)** δ -143.5 (dd, *J* = 24.0, 7.9 Hz), -154.1 (t, *J* = 22.2 Hz), -162.3 (td, *J* = 23.7, 7.8 Hz).

**ESI-HRMS:** Calculated for $C_{11}H_5F_5N$ $(M-Br^-)^+$: 246.0337, Found: 246.0334.

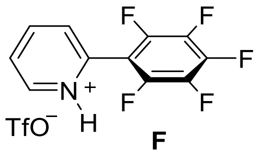

**F**

The title compound was synthesized according to general procedure **C** using 2-pentafluorophenylpyridine (2.0 mmol, 490.0 mg), dry $Et_2O$ (8 mL) and HOTf (0.18 mL, 2 mmol) as reaction reagent. The precipitation was collected by filtration and washed twice with $Et_2O$, and further dried under vacuum to give the product **F** as a white solid (750.5 mg, 95%). The final pyridinium salt was kept in nitrogen atmosphere.

**$^1$H NMR (400 MHz, DMSO-*d*$_6$)** δ 10.21 (s, 1H), 8.80 (d, *J* = 5.0 Hz, 1H), 8.10 (td, *J* = 7.8, 2.0 Hz, 1H), 7.74 (d, *J* = 7.9 Hz, 1H), 7.62 (dd, *J* = 7.7, 5.1 Hz, 1H).

**$^{13}$C NMR (101 MHz, DMSO-*d*$_6$)** δ 149.6, 145.2, 145.5-142.6 (m), 142.3-139.4 (m), 139.7, 138.8-136.0 (m), 126.6, 124.9, 120.8 (q, *J* = 322.3 Hz), 115.3-113.9 (m).

**$^{19}$F NMR (376 MHz, DMSO-*d*$_6$)** δ -77.6 – -78.3 (m), -142.0 – -144.4 (m), -150.7 – -154.9 (m), -162.3 – -162.3 (m).

**ESI-HRMS:** Calculated for $C_{11}H_5F_5N$ $(M-TfO^-)^+$: 246.0337, Found: 246.0335.

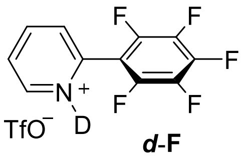

***d*-F**

The title compound was synthesized according to general procedure **C** using 2-pentafluorophenylpyridine (2.0 mmol, 490.0 mg), dry $Et_2O$ (8 mL) and DOTf (0.18 mL, 2 mmol) as reaction reagent. The precipitation was collected by filtration and washed twice with $Et_2O$, and further dried under vacuum to give the product ***d*-F** as a white solid (728.6 mg, 92%). The final pyridinium salt was kept in nitrogen atmosphere.

**$^{1}$H NMR (400 MHz, DMSO-*d*$_6$)** δ 8.81 (d, *J* = 5.0 Hz, 1H), 8.12 (t, *J* = 7.8 Hz, 1H), 7.76 (d, *J* = 7.9 Hz, 1H), 7.64 (dd, *J* = 7.7, 5.0 Hz, 1H).

**$^{13}$C NMR (101 MHz, DMSO-*d*$_6$)** δ 149.5, 145.6-142.9 (m), 145.0, 143.1-142.1 (m), 142.9-139.7 (m), 139.0, 138.8-136.0 (m), 126.9, 125.2, 120.9 (q, *J* = 322.1 Hz), 114.3-114.0 (m).

**$^{19}$F NMR (376 MHz, DMSO-*d*$_6$)** δ -77.9, -143.4 (dd, *J* =16.0 Hz, 8.0 Hz), -153.7 – -153.8 (m), -162.2 – -162.3 (m).

**ESI-HRMS:** Calculated for $C_{11}H_4DF_5N$ $(M-TfO^-)^+$: 247.0399, Found: 247.0361.

## 4. The glycosyl acceptors in this work (2a-2g, 2l-2x, 2ad-2au)

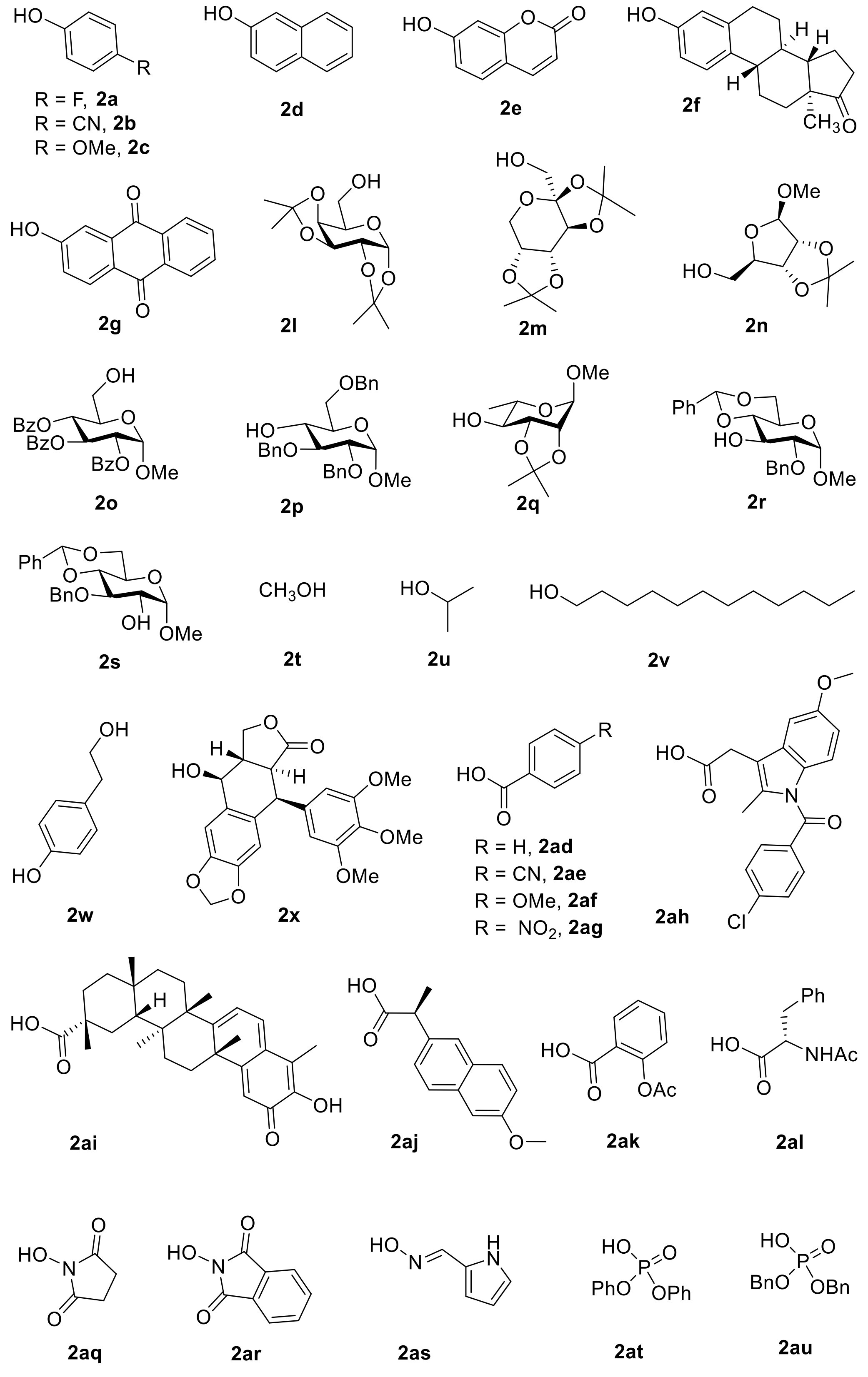


**Supplementary Figure S5.** The acceptors **2a**-2**n**, **2t-2x** and **2ad-2au** are commercially available. The acceptors **2o-2s** were synthesized following literature procedures[8-10].

# 5. *O*-Glycosylation using oxazolidinone-based glycosyl carbamate donors

## 5.1 Optimization of *O*-glycosylation

Procedure: In a glove box filled with nitrogen, to an oven-dried 5 mL tube equipped with a stirring bar were added glycosyl donor **1a** (0.075 mmol, 1.5 equiv.), acceptor **2a** (0.05 mmol, 1.0 equiv.), **Cat** (20 mol%) and solvent (1 mL, 0.05 M). The suspension was stirred at room temperature for 10 h. Upon completion, the yield was determined by $^{19}F$ NMR using trifluoromethoxybenzene or $^{1}H$ NMR using 1,3,5-trimethoxybenzene as an internal standard.

**Supplementary Table S2. Optimization of *O*-glycosylation Conditions.**

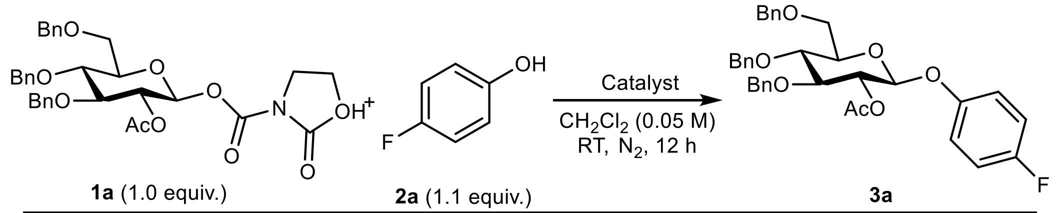


| Entry | Catalyst | Solvent | Yield% | α/β ratio[a] |
|---|---|---|---|---|
| 1[b] | 20 mol% TMSOTf | $CH_2Cl_2$ | trace | -- |
| 2 | 20 mol% TfOH | $CH_2Cl_2$ | 53 | 3 : 1 |
| 3 | 20 mol% TsOH·$H_2O$ | $CH_2Cl_2$ | 45 | 1 : 1 |
| 4[b] | 20 mol% $BF_3$·$Et_2O$ | $CH_2Cl_2$ | 50 | 3 : 1 |
| 5 | 20 mol% $SnCl_4$ | $CH_2Cl_2$ | 52 | 1.6 : 1 |
| 6 | 20 mol% **A** | $CH_2Cl_2$ | 17 | 1 : 4.5 |
| 7 | 20 mol% **B** | $CH_2Cl_2$ | no reaction | -- |
| 8 | 20 mol% **C** | $CH_2Cl_2$ | no reaction | -- |
| 9 | 20 mol% **D** | $CH_2Cl_2$ | 71 | < 1 : 20 |
| 10 | 20 mol% **E** | $CH_2Cl_2$ | 20 | 1 : 2.7 |
| 11 | 20 mol% **D** | $CH_3CN$ or DMF | no reaction | -- |
| 12[c] | 5 mol% **D** | $CH_2Cl_2$ | 96 | < 1 : 20 |
| 13[d] | 20 mol% **D** | $CH_2Cl_2$ | 13 | 1.7 : 1 |
| 14[e] | 5 mol% **D** | $CH_2Cl_2$ | 36 | 1.8 : 1 |
| 15[f] | 5 mol% **D** | $CH_2Cl_2$ | no reaction | -- |
| 16[g] | 5 mol% **D** | $CH_2Cl_2$ | no reaction | -- |
| 17 | none | $CH_2Cl_2$ | no reaction | -- |

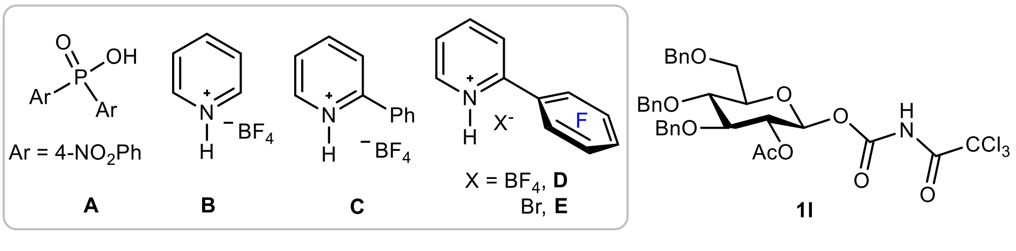


[a]Reaction conditions: **1a** (0.05 mmol, 1.0 equiv.), **2a** (0.055 mmol, 1.1 equiv.), catalyst (0.01 mmol, 20 mol%), $CH_2Cl_2$ (1 mL) at room temperature for 12 h, yields were determined by $^{19}F$ NMR using trifluoromethoxybenzene as an internal standard; α/β ratio was determined by crude $^{1}H$ NMR spectrum. [b]0 °C; [c]**1a** (0.075 mmol), 93% of oxazolidinone was recovered; [d]**1a-α** was used instead of **1a**; [e]**1h** was used instead of **1a**, 40°C; [f]**1i** was used instead of **1a**; [g]**1l** was used instead of **1a**.

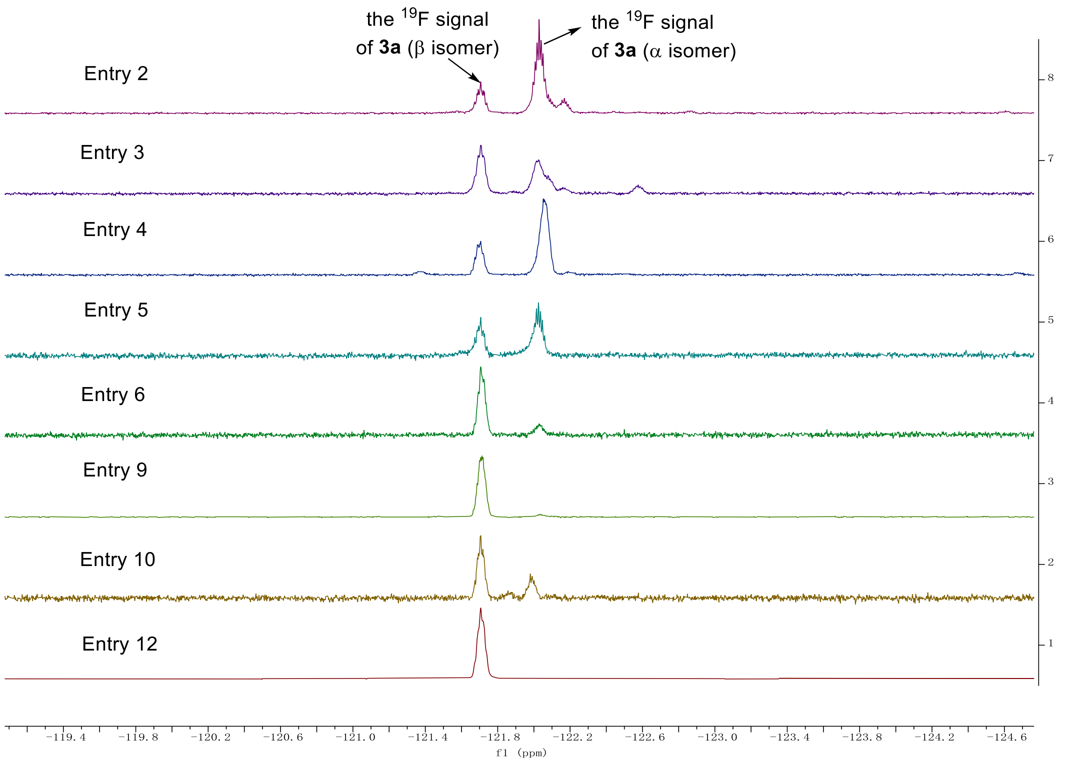


**Supplementary Figure S6. The crude $^{19}$F NMR (376 MHz, $CDCl_3$) spectra of the reaction mixture (Table S2, Entries 2-6, 9-10, 12)**

To confirm the chemical shift of the $^{19}$F signal of **3a-β**, compound **3a (α and β mixture)** was synthesized and characterized by $^{1}$H and $^{19}$F NMR spectroscopy.

## Synthesis of 3a (α and β mixture)

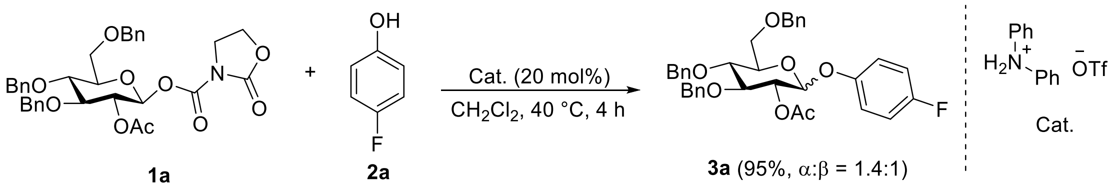


To an oven-dried vial was added glycosyl donor **1a** (45.4 mg, 0.075 mmol, 1.5 equiv.), acceptor **2a** (5.6 mg, 0.05 mmol, 1.0 equiv.), cat. (3.2 mg, 20 mol%) and anhydrous $CH_2Cl_2$ (1 mL, 0.05 M) under nitrogen atmosphere. The reaction mixture was stirred at 40 °C for 4 h and then purified by column chromatography on silica gel with petroleum ether/ethyl acetate (9:1) as eluent to afford **3a (α:β = 1.4:1)** as a white solid (27.8 mg, 95% yield).

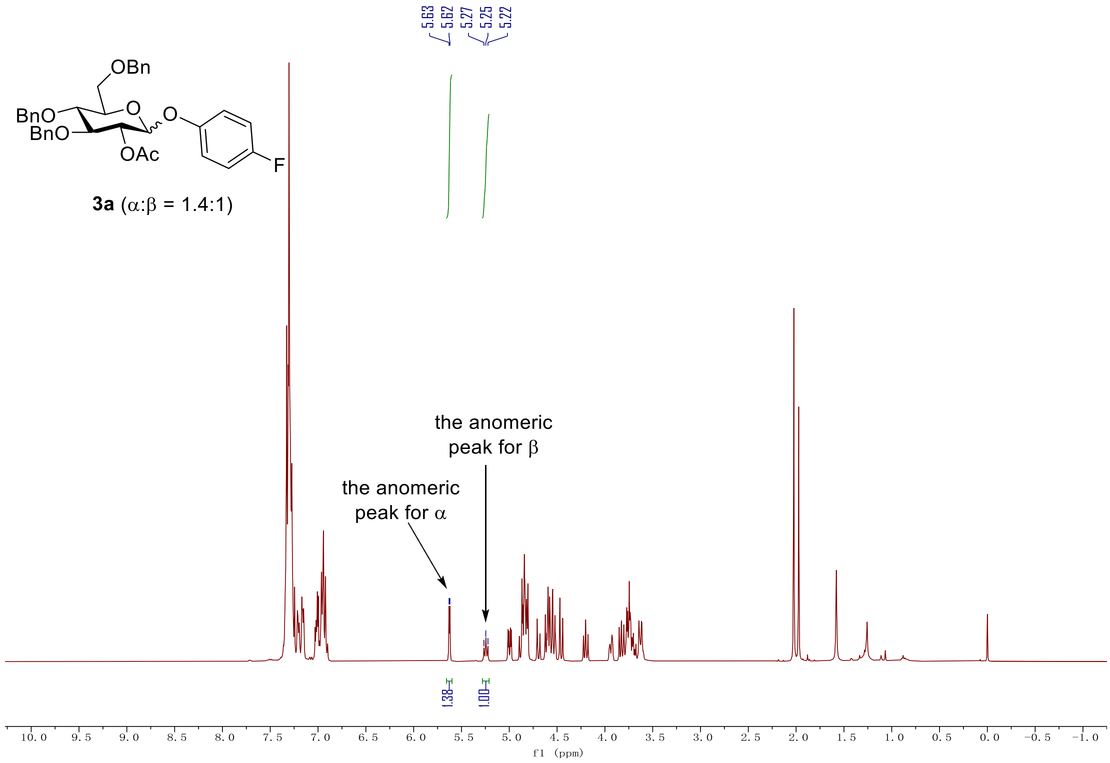


**Supplementary Figure S7. $^{1}$H NMR (400 MHz, $CDCl_3$) Spectra for compound 3a (α and β mixture)**

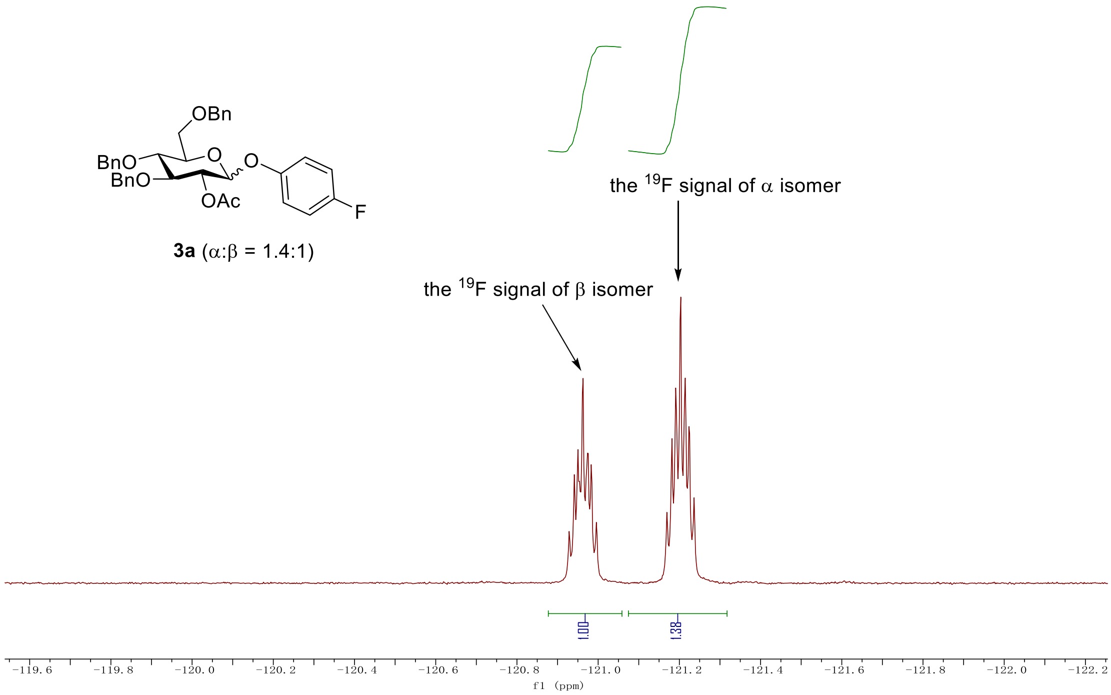


**Supplementary Figure S8. $^{19}$F NMR (376 MHz, $CDCl_3$) Spectra for compound 3a (α and β mixture)**

To determine the $^{19}$F NMR signal of **3av**, compound **3av (in Table S2, Entry 14)** was isolated and characterized by $^{1}$H and $^{19}$F NMR spectroscopy.

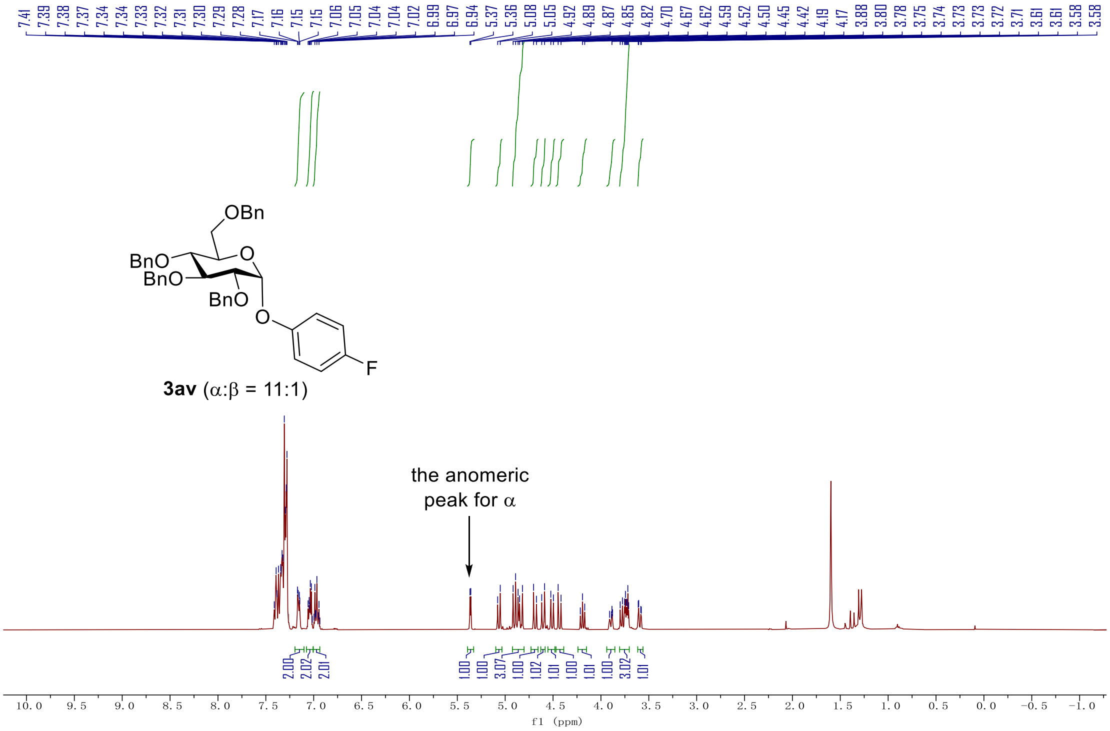


**Supplementary Figure S9. $^{1}$H NMR (400 MHz, $CDCl_3$) Spectra for isolated compound 3av**

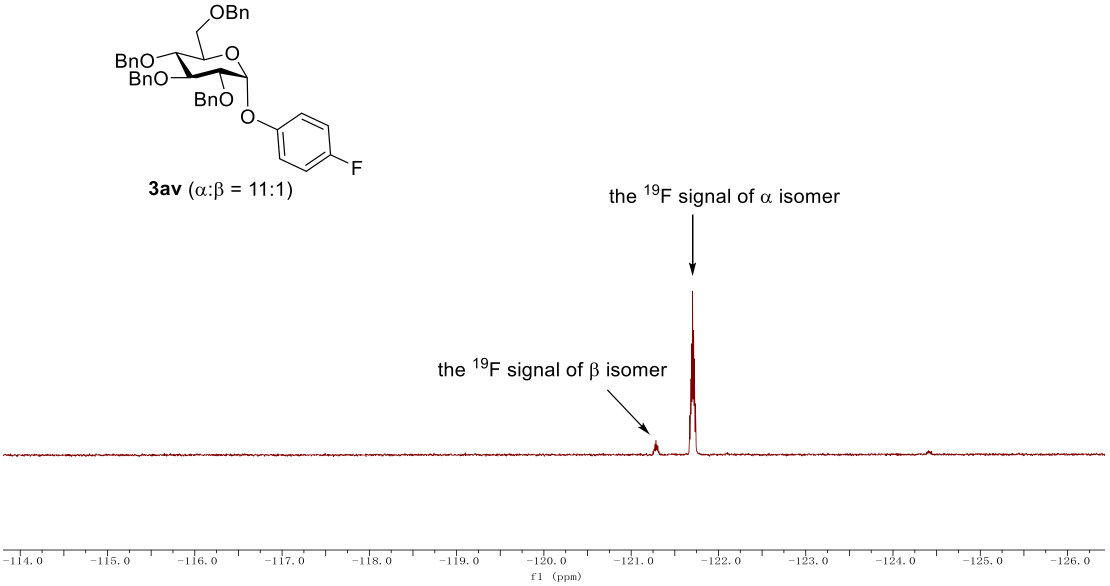


**Supplementary Figure S10. $^{19}$F NMR (376 MHz, $CDCl_3$) Spectra for isolated compound 3av**

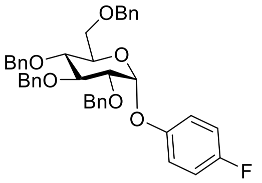


**3av** (α:β = 1.8:1)

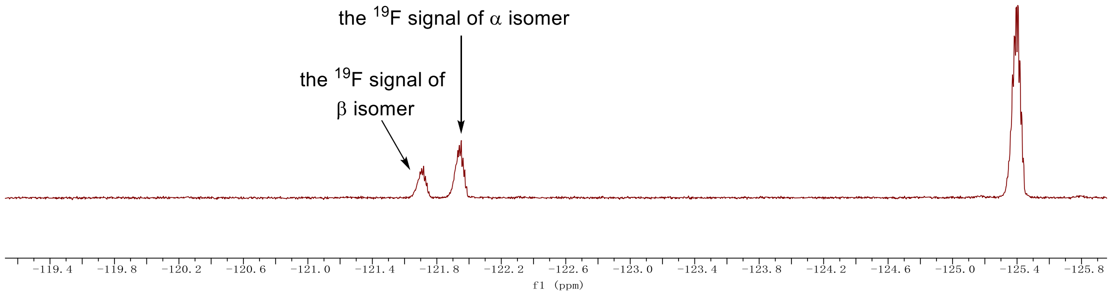


**Supplementary Figure S11. The crude $^{19}F$ NMR (376 MHz, $CDCl_3$) Spectra of the reaction mixture (Table S2, Entry 14)**

The glycosyl donor **1l** was synthesized following literature procedure[11].

## 5.2 General procedure for *O*-glycosylation

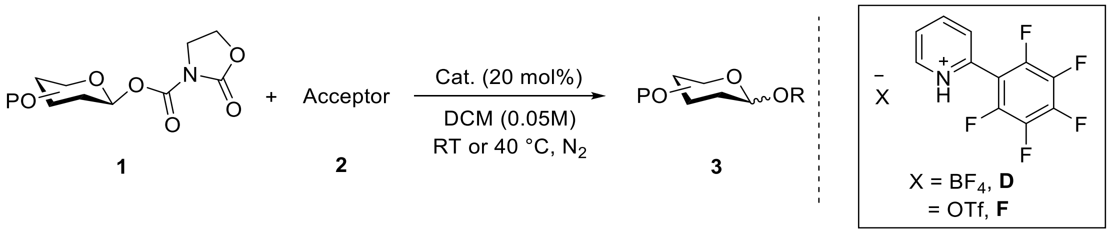


**Supplementary Figure S12. Glycosylation of oxazolidinone-based glycosyl carbamate donors with nucleophilic acceptors.**

**General procedure D.** To an oven-dried vial was added glycosyl donor **1** (0.075 mmol, 1.5 eq.), acceptor (0.05 mmol, 1.0 equiv.), **D** or **F** (5 or 20 mol%) and anhydrous $CH_2Cl_2$ (1 mL, 0.05 M) under nitrogen atmosphere. The suspension was stirred at room temperature or 40 °C for 12 h. The resulting residue was purified by silica gel column chromatography to afford the glycosylated products.

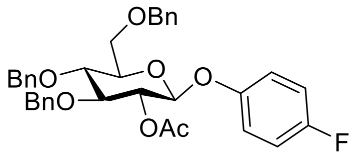

**3a**

**(2*S*,3*R*,4*S*,5*R*,6*R*)-4,5-Bis(benzyloxy)-6-((benzyloxy)methyl)-2-(4-fluorophenoxy)tetrahydro-2*H*-pyran-3-yl acetate**

The title compound was synthesized according to **general procedure D** using glycosyl donor **1a** (45.4 mg, 0.075 mmol) and acceptor **2a** (5.6 mg, 0.05 mmol) as reactants and **D** (0.8 mg, 5 mol%) as the catalyst. The reaction mixture was stirred at room temperature for 12 h and then purified by column chromatography on silica gel with petroleum ether/ethyl acetate (9:1) as eluent to afford **3a** as a colorless syrup (28.1 mg, 96% yield).

**$^1$H NMR (400 MHz, CDCl$_3$)** δ 7.28 – 7.15 (m, 15H), 6.94 – 6.85 (m, 4H), 5.20 (t, *J* = 8.3 Hz, 1H), 4.82 – 4.76 (m, 3H), 4.65 (d, *J* = 11.4 Hz, 1H), 4.56 – 4.47 (m, 3H), 3.76 – 3.63 (m, 4H), 3.59–3.56 (m, 1H), 1.93 (s, 3H).

**$^{13}$C NMR (101 MHz, CDCl$_3$)** δ 169.6, 158.7 (d, *J* = 240.6 Hz), 153.5 (d, *J* = 2.3 Hz), 138.1 (d, *J* = 3.2 Hz), 137.9, 128.6, 128.5, 128.2, 128.1, 128.0, 128.0, 127.8, 127.8, 118.6 (d, *J* = 8.1 Hz), 116.1, 115.9, 100.2, 82.9, 78.0, 75.5, 75.3, 75.2, 73.6, 73.1, 68.8, 21.0.

**$^{19}$F NMR (376 MHz, CDCl$_3$)** δ -120.93.

**$[\alpha]_D^{25}$** = +7.619 (c = 0.21, CHCl$_3$).

**HRMS** (ESI-TOF): calculated for $C_{35}H_{35}FO_7Na^+$ [M+Na$^+$]: 609.2259, found 609.2249.

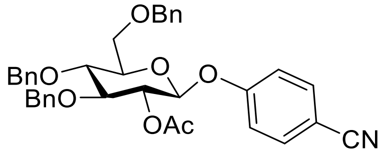

**3b**

**(2*S*,3*R*,4*S*,5*R*,6*R*)-4,5-Bis(benzyloxy)-6-((benzyloxy)methyl)-2-(4-cyanophenoxy)tetrahydro-2*H*-pyran-3-yl acetate**

The title compound was synthesized according to **general procedure D** using glycosyl donor **1a** (45.4 mg, 0.075 mmol) and acceptor **2b** (6.0 mg, 0.05 mmol) as reactants and **D** (0.8 mg, 5 mol%) as the catalyst. The reaction mixture was stirred at room temperature for 12 h and then purified by column chromatography on silica gel with petroleum ether/ethyl acetate (7:1) as eluent to afford **3b** as a white solid (25.2 mg,

85% yield).

**$^1$H NMR (400 MHz, CDCl$_3$)** δ 7.50 (d, *J* = 8.4 Hz, 2H), 7.32 – 7.16 (m, 15H), 7.01 (d, *J* = 8.4 Hz, 2H), 5.25 (t, *J* = 8.1 Hz, 1H), 4.98 (d, *J* = 7.8 Hz, 1H), 4.81 – 4.77 (m, 2H), 4.67 (d, *J* = 11.4 Hz, 1H), 4.56 – 4.45 (m, 3H), 3.76 – 3.70 (m, 3H), 3.65 – 3.61 (m, 2H), 1.92 (s, 3H).

**$^{13}$C NMR (101 MHz, CDCl$_3$)** δ 169.8, 160.6, 138.3, 138.3, 138.1, 134.4, 129.0, 128.9, 128.5, 128.4, 128.3, 128.2, 128.1, 119.3, 117.7, 106.5, 98.9, 83.1, 78.1, 76.1, 75.6, 75.6, 74.0, 73.1, 69.0, 21.3.

**$[\alpha]_D^{25}$** = -28.0 (c = 0.40, CHCl$_3$).

**HRMS** (ESI-TOF): calculated for $C_{36}H_{35}NO_7Na^+$ [M+Na$^+$]: 616.2306, found 616.2298.

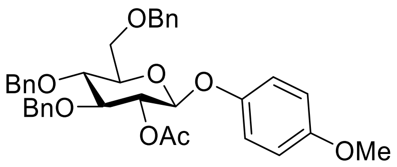


**3c**

**(2*S*,3*R*,4*S*,5*R*,6*R*)-4,5-Bis(benzyloxy)-6-((benzyloxy)methyl)-2-(4-methoxyphenoxy)tetrahydro-2*H*-pyran-3-yl acetate**

The title compound was synthesized according to **general procedure D** using glycosyl donor **1a** (45.4 mg, 0.075 mmol) and acceptor **2c** (6.2 mg, 0.05 mmol) as reactants and **D** (3.3 mg, 20 mol%) as the catalyst. The reaction mixture was stirred at 40 °C for 12 h and then purified by column chromatography on silica gel with petroleum ether/ethyl acetate (5:1) as eluent to afford **3c** as a white solid (28.7 mg, 96% yield).

**$^1$H NMR (400 MHz, CDCl$_3$)** δ 7.27 – 7.15 (m, 15H), 6.91 (d, *J* = 8.9 Hz, 2H), 6.73 (d, *J* = 9.0 Hz, 2H), 5.19 (t, *J* = 8.5 Hz, 1H), 4.78 – 4.75 (m, 3H), 4.65 (d, *J* = 11.4 Hz, 1H), 4.57 – 4.48 (m, 3H), 3.76 – 3.64 (m, 7H), 3.56 – 3.55 (m, 1H), 1.93 (s, 3H).

**$^{13}$C NMR (101 MHz, CDCl$_3$)** δ 169.3, 155.2, 151.2, 137.9, 137.9, 137.6, 128.3, 128.1, 127.8, 127.7, 127.7, 127.6, 127.5, 127.4, 118.3, 114.3, 100.3, 82.7, 77.7, 75.2, 74.9, 73.3, 72.9, 68.5, 55.4, 20.7.

**$[\alpha]_D^{25}$** = +9.3 (c = 0.48, CHCl$_3$).

**HRMS** (ESI-TOF): calculated for $C_{36}H_{38}O_8Na^+$ [M+Na$^+$]: 621.2459, found 621.2450.

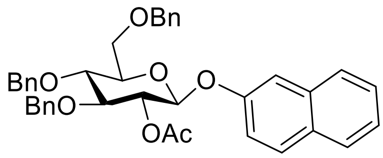


**3d**

**(2*S*,3*R*,4*S*,5*R*,6*R*)-4,5-Bis(benzyloxy)-6-((benzyloxy)methyl)-2-(naphthalen-2-yloxy)tetrahydro-2*H*-pyran-3-yl acetate**

The title compound was synthesized according to **general procedure D** using glycosyl donor **1a** (45.4 mg, 0.075 mmol) and acceptor **2d** (7.2 mg, 0.05 mmol) as reactants and **D** (3.3 mg, 20 mol%) as the catalyst. The reaction mixture was stirred at 40 °C for 12 h and then purified by column chromatography on silica gel with petroleum ether/ethyl acetate (8:1) as eluent to afford **3d** as a white solid (26.0 mg, 84% yield).

**$^1$H NMR (400 MHz, CDCl$_3$)** δ 7.82 – 7.76 (m, 2H), 7.68 (d, *J* = 8.0 Hz, 1H), 7.47 – 7.22 (m, 19H), 5.40 – 5.35 (m, 1H), 5.13 (d, *J* = 7.8 Hz, 1H), 4.90 – 4.87 (m, 2H), 4.76 (d, *J* = 11.4 Hz, 1H), 4.66 – 4.57 (m, 3H), 3.89 – 3.75 (m, 5H), 2.02 (s, 3H).

**$^{13}$C NMR (101 MHz, CDCl$_3$)** δ 169.7, 155.2, 138.2, 138.2, 138.0, 134.4, 130.1, 129.6, 128.6, 128.5, 128.2, 128.1, 128.0, 128.0, 127.9, 127.7, 127.4, 126.5, 124.5, 119.2, 111.5, 99.7, 83.0, 78.1, 75.7, 75.3, 75.2, 73.7, 73.2, 69.0, 21.0.

**$[\alpha]_D^{25}$** = -31.4 (c = 0.28, CHCl$_3$).

**HRMS** (ESI-TOF): calculated for $C_{39}H_{38}O_7Na^+$ [M+Na$^+$]: 641.2510, found 641.2509.

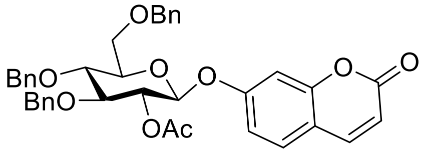


**3e**

**(2*S*,3*R*,4*S*,5*R*,6*R*)-4,5-Bis(benzyloxy)-6-((benzyloxy)methyl)-2-((2-oxo-2*H*-chromen-7-yl)oxy)tetrahydro-2*H*-pyran-3-yl acetate**

The title compound was synthesized according to **general procedure D** using glycosyl donor **1a** (45.4 mg, 0.075 mmol) and acceptor **2e** (8.1 mg, 0.05 mmol) as reactants and **D** (3.3 mg, 20 mol%) as the catalyst. The reaction mixture was stirred at 40 °C for 12 h and then purified by column chromatography on silica gel with petroleum ether/ethyl acetate (6:1) as eluent to afford **3e** as a white solid (28.6 mg, 90% yield).

**$^1$H NMR (400 MHz, CDCl$_3$)** δ 7.64 (d, *J* = 9.5 Hz, 1H), 7.36 – 7.26 (m, 14H), 7.19 (d, *J* = 6.8 Hz, 2H), 7.02 – 6.86 (m, 2H), 6.30 (d, *J* = 9.5 Hz, 1H), 5.30 (t, *J* = 8.4 Hz, 1H), 5.02 (d, *J* = 7.8 Hz, 1H), 4.85 – 4.80 (m, 2H), 4.70 (d, *J* = 11.4 Hz, 1H), 4.62 – 4.57 (m, 2H), 4.51 (d, *J* = 12.0 Hz, 1H), 3.86 – 3.73 (m, 4H), 3.67 (d, *J* = 8.3 Hz, 1H), 1.96 (s, 3H).

**$^{13}$C NMR (101 MHz, CDCl$_3$)** δ 169.6, 161.1, 160.1, 155.5, 143.3, 138.0, 137.9, 137.8, 128.9, 128.6, 128.5, 128.2, 128.1, 128.1, 128.0, 127.9, 127.8, 114.4, 114.3, 114.2, 104.1, 98.9, 82.7, 77.6, 75.7, 75.3, 75.3, 73.7, 72.7, 68.4, 21.0.

**$[\alpha]_D^{25}$** = +51.1 (c = 0.17, CHCl$_3$).

**HRMS** (ESI-TOF): calculated for $C_{38}H_{36}O_9Na^+$ [M+Na$^+$]: 659.2252, found 659.2244.

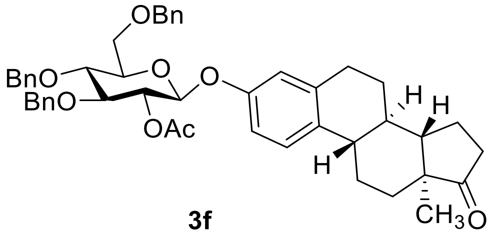


**3f**

**(2*S*,3*R*,4*S*,5*R*,6*R*)-4,5-Bis(benzyloxy)-6-((benzyloxy)methyl)-2-(((8R,9S,13S,14S)-13-methyl-17-oxo-7,8,9,11,12,13,14,15,16,17-decahydro-6*H*-cyclopenta[a]phenanthren-3-yl)oxy)tetrahydro-2*H*-pyran-3-yl acetate**

The title compound was synthesized according to **general procedure D** using glycosyl donor **1a** (45.4 mg, 0.075 mmol) and acceptor **2f** (13.5 mg, 0.05 mmol) as reactants and **D** (3.3 mg, 20 mol%) as the catalyst. The reaction mixture was stirred at 40 °C for 12 h and then purified by column chromatography on silica gel with petroleum ether/ethyl acetate (3:1) as eluent to afford **3f** as a white solid (35.7 mg, 96% yield).

**$^{1}$H NMR (400 MHz, CDCl$_3$)** δ 7.28 – 7.12 (m, 16H), 6.78 – 6.75 (m, 1H), 6.71 –6.70 (m, 1H), 5.23 – 5.19 (m, 1H), 4.88 – 4.85 (m, 1H), 4.80 – 4.76 (m, 2H), 4.68 – 4.64 (m, 1H), 4.58 – 4.48 (m, 3H), 3.78 – 3.61 (m, 5H), 2.80 – 2.76 (m, 2H), 2.49 – 2.42 (m, 1H), 2.34 – 2.33 (m, 1H), 2.23 – 2.20 (m, 1H), 2.14 – 1.98 (m, 2H), 1.93 – 1.90 (m, 5H), 1.61 – 1.33 (m, 6H), 0.86 (s, 3H).

**$^{13}$C NMR (101 MHz, CDCl$_3$)** δ 169.6, 155.5, 138.2, 138.2, 138.0, 137.9, 134.3, 128.6, 128.4, 128.1, 128.0, 128.0, 127.9, 127.8, 127.7, 126.5, 117.1, 114.5, 99.6, 83.0, 78.0, 75.6, 75.2, 75.2, 73.7, 73.1, 68.9, 50.5, 48.1, 44.2, 38.4, 36.0, 31.7, 29.7, 26.6, 26.0, 21.7, 21.0, 14.0.

**$[\alpha]_D^{25}$** = +65.5 (c = 0.18, CHCl$_3$).

**HRMS** (ESI-TOF): calculated for $C_{47}H_{52}O_8Na^+$ [M+Na$^+$]: 767.3554, found 767.3542.

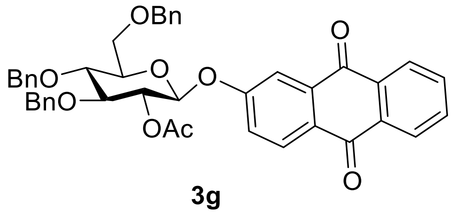


**3g**

**(2*S*,3*R*,4*S*,5*R*,6*R*)-4,5-Bis(benzyloxy)-6-((benzyloxy)methyl)-2-((9,10-dioxo-8a,9,10,10a-tetrahydroanthracen-2-yl)oxy)tetrahydro-2*H*-pyran-3-yl acetate**

The title compound was synthesized according to **general procedure D** using glycosyl donor **1a** (45.4 mg, 0.075 mmol) and acceptor **2g** (11.3 mg, 0.05 mmol) as reactants and **D** (3.3 mg, 20 mol%) as the catalyst. The reaction mixture was stirred at 40 °C for 12 h and then purified by column chromatography on silica gel with petroleum ether/ethyl acetate (7:1) as eluent to afford **3g** as a white solid (32.9 mg, 94% yield).

**$^1$H NMR (400 MHz, $CDCl_3$)** δ 8.27 – 8.19 (m, 3H), 7.79 – 7.74 (m, 3H), 7.33 – 7.15 (m, 16H), 5.30 (t, *J* = 8.3 Hz, 1H), 5.16 (d, *J* = 7.7 Hz, 1H), 4.81 – 4.77 (m, 2H), 4.67 (d, *J* = 11.4 Hz, 1H), 4.57 – 4.54 (m, 2H), 4.45 (d, *J* = 12.1 Hz, 1H), 3.84 – 3.69 (m, 5H), 1.93 (s, 3H).

**$^{13}$C NMR (101 MHz, $CDCl_3$)** δ 182.6, 181.9, 169.2, 161.4, 137.8, 137.7, 137.6, 135.3, 134.0, 133.7, 133.4, 129.7, 128.3, 128.3, 128.2, 127.8, 127.8, 127.7, 127.7, 127.6, 127.5, 127.0, 122.5, 113.3, 98.2, 82.5, 77.4, 75.5, 75.0, 74.9, 73.4, 72.5, 68.2, 20.7.

**$[\alpha]_D^{25}$** = -6.1 (c = 0.18, $CHCl_3$).

**HRMS** (ESI-TOF): calculated for $C_{43}H_{38}O_9Na^+$ [M+Na$^+$]: 721.2408, found 721.2397.

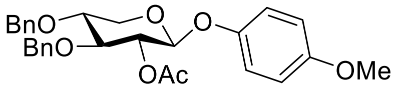


**3h**

**(2*S*,3*R*,4*S*,5*R*)-4,5-Bis(benzyloxy)-2-(4-methoxyphenoxy)tetrahydro-2*H*-pyran-3-yl acetate**

The title compound was synthesized according to **general procedure D** using glycosyl donor **1d** (36.4 mg, 0.075 mmol) and acceptor **2c** (6.2 mg, 0.05 mmol) as reactants and **F** (4.0 mg, 20 mol%) as the catalyst. The reaction mixture was stirred at 40 °C for 12 h and then purified by column chromatography on silica gel with petroleum ether/ethyl acetate (10:1) as eluent to afford **3h** as a white solid (22.2 mg, 93% yield).

**$^1$H NMR (400 MHz, $CDCl_3$)** δ 7.37 – 7.29 (m, 10H), 6.94 – 6.91 (m, 2H), 6.83 – 6.79 (m, 2H), 5.16 (t, *J* = 8.7 Hz, 1H), 4.87 – 4.84 (m, 2H), 4.74 – 4.71 (m, 2H), 4.63 (d, *J* = 11.7 Hz, 1H), 4.07 – 4.03 (m, 1H), 3.78 – 3.72 (m, 4H), 3.67 (t, *J* = 8.4 Hz, 1H), 3.39 – 3.34 (m, 1H), 2.01 (s, 3H).

**$^{13}$C NMR (101 MHz, CDCl$_3$)** δ 169.7, 155.6, 151.2, 138.4, 138.0, 128.7, 128.5, 128.1, 128.0, 128.0, 127.9, 118.6, 114.7, 100.8, 80.6, 77.4, 74.6, 73.3, 72.2, 63.6, 55.8, 21.0.

**[α]$_D$$^{25}$** = +5.2 (c = 0.50, CHCl$_3$).

**HRMS** (ESI-TOF): calculated for $C_{28}H_{30}O_7Na^+$ [M+Na$^+$]: 501.1884, found 501.1875.

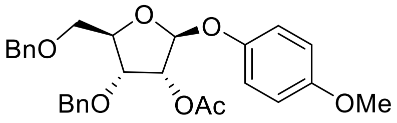


**3i**

**(2*S*,3*R*,4*R*,5*R*)-4-(benzyloxy)-5-((benzyloxy)methyl)-2-(4-methoxyphenoxy)tetrahydrofuran-3-yl acetate**

The title compound was synthesized according to **general procedure D** using glycosyl donor **1g** or **1g-α** (36.2 mg, 0.075 mmol) and acceptor **2c** (6.2 mg, 0.05 mmol) as reactants and **D** (3.3 mg, 20 mol%) as the catalyst. The reaction mixture was stirred at 40 °C for 12 h and then purified by column chromatography on silica gel with petroleum ether/ethyl acetate (10:1) as eluent to afford **3i** as a white solid (23.7 mg, 99% yield).

**$^1$H NMR (400 MHz, CDCl$_3$)** δ 7.35 – 7.24 (m, 10H), 6.99 – 6.95 (m, 2H), 6.85 – 6.81 (d, 2H), 5.57 (s, 1H), 5.47 (d, *J* = 4.0 Hz, 1H), 4.66 (d, *J* = 11.4 Hz, 1H), 4.53 – 4.50 (m, 3H), 4.37 – 4.30 (m, 2H), 3.79 (s, 3H), 3.68 – 3.65 (m, 1H), 3.60 – 3.56 (m, 1H), 2.17 (s, 3H).

**$^{13}$C NMR (101 MHz, CDCl$_3$)** δ 170.2, 155.1, 150.5, 138.3, 137.6, 128.6, 128.4, 128.1, 127.7, 127.6, 118.0, 114.7, 104.5, 81.4, 77.7, 77.5, 77.2, 76.8, 74.6, 73.4, 73.3, 70.8, 55.8, 21.0.

**[α]$_D$$^{25}$** = -25.7 (c = 0.50, CHCl$_3$).

**HRMS** (ESI-TOF): calculated for $C_{28}H_{30}O_7Na^+$ [M+Na$^+$]: 501.1884, found 501.1880.

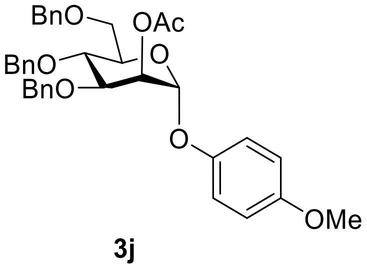


**3j**

**(2*R*,3*S*,4*S*,5*R*,6*R*)-4,5-Bis(benzyloxy)-6-((benzyloxy)methyl)-2-(4-methoxyphenoxy)tetrahydro-2*H*-pyran-3-yl acetate**

The title compound was synthesized according to **general procedure D** using glycosyl donor **1b** (45.4 mg, 0.075 mmol) and acceptor **2c** (6.2 mg, 0.05 mmol) as reactants and **F** (4.0 mg, 20 mol%) as the catalyst. The reaction mixture was stirred at 40 °C for 36 h and then purified by column chromatography on silica gel with petroleum ether/ethyl acetate (7:1) as eluent to afford **3j** as a white solid (23.0 mg, 77% yield).

**$^1$H NMR (400 MHz, $CDCl_3$)** δ 7.36 – 7.22 (m, 13H), 7.16 (d, *J* = 7.5 Hz, 2H), 6.98 (d, *J* = 8.6 Hz, 2H), 6.78 (d, *J* = 8.5 Hz, 2H), 5.54 (s, 1H), 5.46 (s, 1H), 4.88 (d, *J* = 10.7 Hz, 1H), 4.76 (d, *J* = 11.0 Hz, 1H), 4.66 – 4.59 (m, 2H), 4.50 – 4.42 (m, 2H), 4.19 – 4.16 (m, 1H), 4.02 – 3.92 (m, 2H), 3.82 – 3.78 (m, 1H), 3.74 (s, 3H), 3.66 (d, *J* = 10.9 Hz, 1H), 2.17 (s, 3H).

**$^{13}$C NMR (101 MHz, $CDCl_3$)** δ 170.6, 155.2, 150.1, 138.4, 138.3, 138.0, 128.6, 128.5, 128.4, 128.2, 128.0, 127.9, 127.9, 127.8, 127.7, 117.9, 114.7, 97.0, 78.2, 75.4, 74.3, 73.5, 72.1, 72.0, 68.8, 68.8, 55.7, 21.2.

**$[\alpha]_D^{25}$** = +26.3 (c = 0.60, $CHCl_3$).

**HRMS** (ESI-TOF): calculated for $C_{36}H_{38}O_8Na^+$ [M+Na$^+$]: 621.2459, found 621.2452.

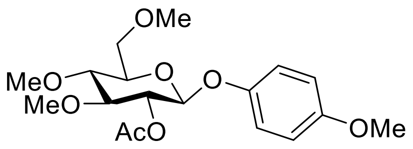


**3k**

**(2*S*,3*R*,4*S*,5*R*,6*R*)-4,5-dimethoxy-6-(methoxymethyl)-2-(4-methoxyphenoxy)tetrahydro-2*H*-pyran-3-yl acetate**

The title compound was synthesized according to **general procedure D** using glycosyl donor **1l** (28.4 mg, 0.075 mmol) and acceptor **2c** (6.2 mg, 0.05 mmol) as reactants and **D** (3.3 mg, 20 mol%) as the catalyst. The reaction mixture was stirred at 40 °C for 12 h and then purified by column chromatography on silica gel with petroleum ether/ethyl acetate (4:1) as eluent to afford **3k** as a colorless syrup (17.8 mg, 96% yield).

**$^1$H NMR (400 MHz, $CDCl_3$)** δ 6.93 (d, *J* = 8.8 Hz, 2H), 6.80 (d, *J* = 9.1 Hz, 2H), 5.10 (t, *J* = 8.3 Hz, 1H), 4.79 (d, *J* = 7.9 Hz, 1H), 3.76 (s, 3H), 3.69 – 3.66 (m, 1H), 3.62 – 3.59 (m, 1H), 3.56 (s, 3H), 3.55 (s, 3H), 3.42 – 3.40 (m, 4H), 3.37 – 3.35 (m, 2H), 2.11 (s, 3H).

**$^{13}$C NMR (101 MHz, $CDCl_3$)** δ 169.6, 155.5, 151.6, 118.5, 114.6, 100.5, 84.7, 79.2, 75.3, 73.0, 71.3, 60.6, 60.3, 59.6, 55.8, 21.1.

**$[\alpha]_D^{25}$** = -3.9 (c = 0.18, $CHCl_3$).

**HRMS** (ESI-TOF): calculated for $C_{18}H_{26}O_8Na^+$ [M+Na$^+$]: 365.1571, found 365.1555.

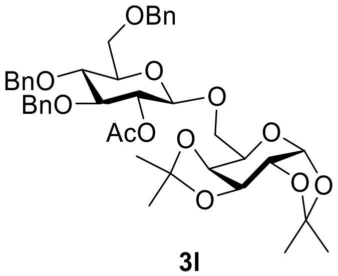


**3l**

**(2*R*,3*R*,4*S*,5*R*,6*R*)-4,5-Bis(benzyloxy)-6-((benzyloxy)methyl)-2-(((3aR,5R,5aS,8aS,8bR)-2,2,7,7-tetramethyltetrahydro-5*H*-bis([1,3]dioxolo)[4,5-b:4',5'-d]pyran-5-yl)methoxy)tetrahydro-2*H*-pyran-3-yl acetate**

The title compound was synthesized according to **general procedure D** using glycosyl donor **1a** (45.4 mg, 0.075 mmol) and acceptor **2l** (13.0 mg, 0.05 mmol) as reactants and **D** (3.3 mg, 20 mol%) as the catalyst. The reaction mixture was stirred at 40 °C for 12 h and then purified by column chromatography on silica gel with petroleum ether/ethyl acetate (5:1) as eluent to afford **3l** as a colorless syrup (35.2 mg, 96% yield).

**$^1$H NMR (400 MHz, CDCl$_3$)** δ 7.31 – 7.25 (m, 13H), 7.15 – 7.13 (m, 2H), 5.46 (d, *J* = 4.9 Hz, 1H), 4.97 (t, *J* = 8.4 Hz, 1H), 4.77– 4.73 (m, 2H), 4.66 – 4.50 (m, 5H), 4.41 (d, *J* = 8.0 Hz, 1H), 4.25 – 4.24 (m, 1H), 4.16 – 4.14 (m, 1H), 4.04 – 4.01 (m, 1H), 3.89 (d, *J* = 5.4 Hz, 1H), 3.70 – 3.57 (m, 5H), 3.44 (d, *J* = 8.7 Hz, 1H), 1.98 (s, 3H), 1.47 (s, 3H), 1.39 (s, 3H), 1.28 (s, 3H), 1.27 (s, 3H).

**$^{13}$C NMR (101 MHz, CDCl$_3$)** δ 170.2, 138.7, 138.6, 138.4, 128.9, 128.8, 128.8, 128.4, 128.3, 128.3, 128.2, 128.1, 128.0, 109.8, 109.1, 102.3, 96.7, 83.3, 78.4, 75.6, 75.5, 75.4, 74.0, 73.5, 71.8, 71.1, 71.0, 69.9, 69.1, 68.3, 26.5, 26.4, 25.5, 24.8, 21.4.
**$[\alpha]_D^{25}$** = -16.6 (c = 0.50, $CHCl_3$).

**HRMS** (ESI-TOF): calculated for $C_{41}H_{50}O_{12}Na^+$ [M+Na$^+$]: 757.3194, found 757.3182.

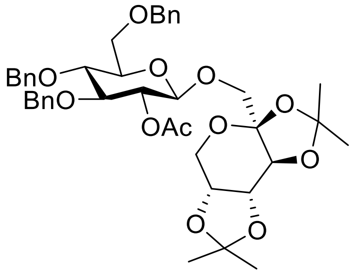


**3m**

**(2*R*,3*R*,4*S*,5*R*,6*R*)-4,5-Bis(benzyloxy)-6-((benzyloxy)methyl)-2-(((3a*S*,5a*S*,8a*R*,8b*S*)-2,2,7,7-tetramethyltetrahydro-3aH-bis([1,3]dioxolo)[4,5-**

**b:4',5'-d]pyran-3a-yl)methoxy)tetrahydro-2*H*-pyran-3-yl acetate**

The title compound was synthesized according to **general procedure D** using glycosyl donor **1a** (45.4 mg, 0.075 mmol) and acceptor **2m** (13.0 mg, 0.05 mmol) as reactants and **D** (3.3 mg, 20 mol%) as the catalyst. The reaction mixture was stirred at 40 °C for 12 h and then purified by column chromatography on silica gel with petroleum ether/ethyl acetate (5:1) as eluent to afford **3m** as a colorless syrup (34.5 mg, 94% yield).

**$^1$H NMR (400 MHz, CDCl$_3$)** δ 7.33 – 7.24 (m, 13H), 7.20 – 7.17 (m, 2H), 5.03 (t, *J* = 8.6 Hz, 1H), 4.80 – 4.75 (m, 2H), 4.65 – 4.45 (m, 7H), 4.20 (d, *J* = 7.9 Hz, 1H), 3.93 – 3.89 (m, 2H), 3.76 – 3.61 (m, 6H), 3.49 – 3.46 (m, 1H), 1.91 (s, 3H), 1.50 (s, 3H), 1.41 (s, 3H), 1.34 (s, 3H), 1.29 (s, 3H).

**$^{13}$C NMR (101 MHz, CDCl$_3$)** δ 169.9, 138.7, 138.6, 138.4, 128.9, 128.8, 128.5, 128.3, 128.3, 128.2, 128.1, 128.0, 109.4, 109.1, 102.5, 100.9, 83.6, 78.4, 75.9, 75.5, 75.4, 74.2, 73.4, 71.5, 70.6, 70.2, 69.7, 69.2, 61.6, 27.0, 26.4, 25.9, 24.5, 21.4.

**$[\alpha]_D^{25}$** = +19.3 (c = 0.44, CHCl$_3$).

**HRMS** (ESI-TOF): calculated for $C_{41}H_{50}O_{12}Na^+$ [M+Na$^+$]: 757.3194, found 757.3181.

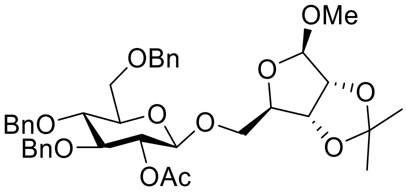


**3n**

**(2*R*,3*R*,4*S*,5*R*,6*R*)-4,5-Bis(benzyloxy)-6-((benzyloxy)methyl)-2-(((3a*R*,4*R*,6*R*,6a*R*)-6-methoxy-2,2-dimethyltetrahydrofuro[3,4-d][1,3]dioxol-4-yl)methoxy)tetrahydro-2*H*-pyran-3-yl acetate**

The title compound was synthesized according to **general procedure D** using glycosyl donor **1a** (45.4 mg, 0.075 mmol) and acceptor **2n** (10.2 mg, 0.05 mmol) as reactants and **D** (3.3 mg, 20 mol%) as the catalyst. The reaction mixture was stirred at 40 °C for 12 h and then purified by column chromatography on silica gel with petroleum ether/ethyl acetate (5:1) as eluent to afford **3n** as a colorless syrup (30.9 mg, 91% yield).

**$^1$H NMR (400 MHz, CDCl$_3$)** δ 7.32 – 7.24 (m, 13H), 7.16 – 7.14 (m, 2H), 4.98 (t, *J* = 8.5 Hz, 1H), 4.91 (s, 1H), 4.78 – 4.75 (m, 2H), 4.66 – 4.59 (m, 3H), 4.54 – 4.51 (m, 3H), 4.38 (d, *J* = 8.0 Hz, 1H), 4.26 – 4.22 (m, 1H), 3.78 – 3.55 (m, 6H), 3.47 – 3.44 (m, 1H), 3.26 (s, 3H), 1.96 (s, 3H), 1.44 (s, 3H), 1.26 (s, 3H).

**$^{13}$C NMR (101 MHz, CDCl$_3$)** δ 170.1, 138.6, 138.5, 138.3, 128.9, 128.8, 128.5, 128.4, 128.3, 128.2, 128.1, 112.7, 109.9, 101.2, 85.5, 85.1, 83.4, 82.4, 78.3, 75.7, 75.5, 74.0, 73.4, 69.8, 69.1, 55.2, 26.9, 25.3, 21.3.

**[α]D25** = -23.8 (c = 0.13, $CHCl_3$).

**HRMS** (ESI-TOF): calculated for $C_{38}H_{46}O_{11}Na^+$ [M+$Na^+$]: 701.2932, found 701.2919.

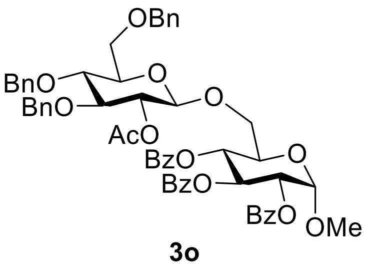


**3o**

**(2*R*,3*R*,4*S*,5*R*,6*S*)-2-((((2*R*,3*R*,4*S*,5*R*,6*R*)-3-Acetoxy-4,5-bis(benzyloxy)-6-((benzyloxy)methyl)tetrahydro-2*H*-pyran-2-yl)oxy)methyl)-6-methoxytetrahydro-2*H*-pyran-3,4,5-triyl tribenzoate**

The title compound was synthesized according to **general procedure D** using glycosyl donor **1a** (45.4 mg, 0.075 mmol) and acceptor **2o** (25.3 mg, 0.05 mmol) as reactants and **F** (4.0 mg, 20 mol%) as the catalyst. The reaction mixture was stirred at 40 °C for 12 h and then purified by column chromatography on silica gel with petroleum ether/ethyl acetate (5:1) as eluent to afford **3o** as a colorless syrup (45.6 mg, 93% yield).

**$^1$H NMR (400 MHz, $CDCl_3$)** δ 7.98 – 7.91 (m, 4H), 7.85 (d, *J* = 7.7 Hz, 2H), 7.49 (t, *J* = 7.5 Hz, 2H), 7.42 – 7.22 (m, 20H), 7.17 – 7.15 (m, 2H), 6.14 (t, *J* = 9.7 Hz, 1H), 5.42 (t, *J* = 9.9 Hz, 1H), 5.25 – 5.19 (m, 2H), 5.04 (t, *J* = 8.3 Hz, 1H), 4.80 – 4.76 (m, 2H), 4.68 (d, *J* = 11.4 Hz, 1H), 4.55 – 4.52 (m, 2H), 4.46 – 4.41 (m, 2H), 4.28 – 4.24 (m, 1H), 4.07 (d, *J* = 10.8 Hz, 1H), 3.69 – 3.62 (m, 5H), 3.47 – 3.44 (m, 4H), 2.01 (s, 3H).

**$^{13}$C NMR (101 MHz, $CDCl_3$)** δ 170.0, 166.3, 166.2, 165.8, 138.6, 138.4, 138.3, 133.8, 133.8, 133.5, 130.3, 130.3, 130.1, 129.7, 129.5, 129.3, 128.8, 128.8, 128.7, 128.4, 128.3, 128.2, 128.2, 128.1, 128.0, 101.8, 97.1, 83.3, 78.2, 75.6, 75.5, 75.4, 73.8, 73.5, 72.6, 71.0, 69.9, 69.1, 68.9, 68.8, 55.8, 21.4.

**[α]D25** = +28.4 (c = 0.33, $CHCl_3$).

**HRMS** (ESI-TOF): calculated for $C_{57}H_{56}O_{15}Na^+$ [M+$Na^+$]: 1003.3511, found 1003.3494.

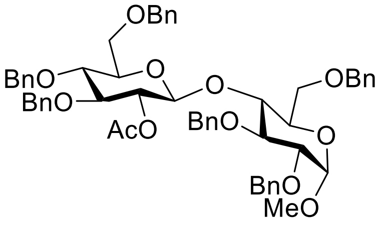


**3p**

**(2*S*,3*R*,4*S*,5*R*,6*R*)-4,5-Bis(benzyloxy)-6-((benzyloxy)methyl)-2-(((2*R*,3*R*,4*S*,5*R*,6*S*)-4,5-bis(benzyloxy)-2-((benzyloxy)methyl)-6-methoxytetrahydro-2*H*-pyran-3-yl)oxy)tetrahydro-2*H*-pyran-3-yl acetate**

The title compound was synthesized according to **general procedure D** using glycosyl donor **1a** (45.4 mg, 0.075 mmol) and acceptor **2p** (23.2 mg, 0.05 mmol) as reactants and **F** (4.0 mg, 20 mol%) as the catalyst. The reaction mixture was stirred at 40 °C for 12 h and then purified by column chromatography on silica gel with petroleum ether/ethyl acetate (5:1) as eluent to afford **3p** as a colorless syrup (46.4 mg, 99% yield).

**$^1$H NMR (400 MHz, $CDCl_3$)** δ 7.37 – 7.16 (m, 30H), 5.05 (d, *J* = 11.5 Hz, 1H), 4.94 (t, *J* = 8.8 Hz, 1H), 4.79 – 4.67 (m, 5H), 4.61 – 4.32 (m, 8H), 3.90 – 3.83 (m, 2H), 3.74 – 3.73 (m, 1H), 3.69 – 3.57 (m, 4H), 3.51 – 3.42 (m, 3H), 3.35 (s, 3H), 3.32 – 3.28 (m, 1H), 1.87 (s, 3H).

**$^{13}$C NMR (101 MHz, $CDCl_3$)** δ 169.7, 140.0, 138.8, 138.8, 138.6, 138.4, 138.3, 129.0, 128.9, 128.8, 128.8, 128.7, 128.7, 128.6, 128.5, 128.5, 128.3, 128.3, 128.2, 128.2, 128.2, 128.1, 128.1, 128.0, 127.8, 127.5, 100.7, 98.8, 83.5, 80.6, 79.4, 78.5, 77.4, 75.7, 75.7, 75.4, 75.3, 74.1, 74.1, 74.0, 73.8, 70.3, 69.2, 68.3, 55.8, 21.4.

**$[\alpha]_D^{25}$** = +16.4 (c = 0.25, $CHCl_3$).

**HRMS** (ESI-TOF): calculated for $C_{57}H_{62}O_{12}Na^+$ [M+Na$^+$]: 961.4133, found 961.4120.

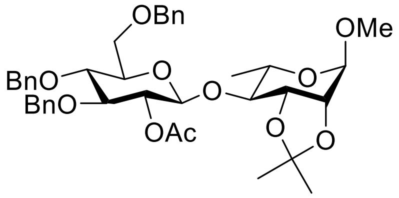


**3q**

**(2*S*,3*R*,4*S*,5*R*,6*R*)-4,5-Bis(benzyloxy)-6-((benzyloxy)methyl)-2-(((3a*R*,4*R*,6*R*,7*R*,7a*S*)-4-methoxy-2,2,6-trimethyltetrahydro-4*H*-[1,3]dioxolo[4,5-c]pyran-7-yl)oxy)tetrahydro-2*H*-pyran-3-yl acetate**

The title compound was synthesized according to **general procedure D** using glycosyl donor **1a** (45.4 mg, 0.075 mmol) and acceptor **2q** (10.9 mg, 0.05 mmol) as reactants and **F** (4.0 mg, 20 mol%) as the catalyst. The reaction mixture was stirred at 40 °C for 12 h and then purified by column chromatography on silica gel with petroleum ether/ethyl acetate (5:1) as eluent to afford **3q** as a white solid (30.8 mg, 89% yield).

**$^1$H NMR (400 MHz, $CDCl_3$)** δ 7.27 – 7.14 (m, 15H), 4.90 (t, *J* = 8.3 Hz, 1H), 4.78 – 4.72 (m, 4H), 4.63 – 4.47 (m, 4H), 4.00 (s, 2H), 3.69 – 3.61 (m, 4H), 3.55 – 3.49 (m, 2H), 3.39 – 3.37 (m, 1H), 3.29 (s, 3H), 1.93 (s, 3H), 1.40 (s, 3H), 1.26 – 1.24 (m, 6H).

**$^{13}$C NMR (101 MHz, CDCl$_3$)** δ 170.2, 138.8, 138.7, 138.5, 128.9, 128.8, 128.8, 128.4, 128.2, 128.2, 128.1, 128.0, 109.7, 100.3, 98.3, 83.5, 79.4, 78.8, 78.5, 76.4, 75.7, 75.4, 74.0, 69.1, 64.5, 55.2, 28.4, 26.9, 21.5, 18.1.

**[α]$_D^{25}$** = -8.5 (c = 0.35, CHCl$_3$).

**HRMS** (ESI-TOF): calculated for $C_{39}H_{48}O_{11}Na^+$ [M+Na$^+$]: 715.3089, found 715.3075.

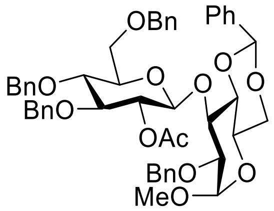


**3r**

**(2*S*,3*R*,4*S*,5*R*,6*R*)-4,5-Bis(benzyloxy)-2-(((2*R*,4a*R*,6*S*,7*R*,8*S*,8a*R*)-7-(benzyloxy)-6-methoxy-2-phenylhexahydropyrano[3,2-d][1,3]dioxin-8-yl)oxy)-6-((benzyloxy)methyl)tetrahydro-2*H*-pyran-3-yl acetate**

The title compound was synthesized according to **general procedure D** using glycosyl donor **1a** (45.4 mg, 0.075 mmol) and acceptor **2r** (18.6 mg, 0.05 mmol) as reactants and **D** (3.3 mg, 20 mol%) as the catalyst. The reaction mixture was stirred at 40 °C for 12 h and then purified by column chromatography on silica gel with petroleum ether/ethyl acetate (5:1) as eluent to afford **3r** as a colorless syrup (39.0 mg, 92% yield).

**$^1$H NMR (400 MHz, CDCl$_3$)** δ 7.38 – 7.26 (m, 23H), 7.14 – 7.12 (m, 2H), 5.40 (s, 1H), 5.06 (t, $J$ = 8.8 Hz, 1H), 4.79 – 4.70 (m, 4H), 4.63 (d, $J$ = 11.3 Hz, 1H), 4.55 – 4.41 (m, 5H), 4.20 – 4.16 (m, 2H), 3.77 – 3.72 (m, 2H), 3.67 – 3.55 (m, 3H), 3.52 – 3.47 (m, 2H), 3.43 – 3.41 (m, 1H), 3.35 (s, 3H), 3.21 – 3.17 (m, 1H), 1.92 (s, 3H).

**$^{13}$C NMR (101 MHz, CDCl$_3$)** δ 170.2, 138.7, 138.7, 138.7, 138.4, 137.8, 129.4, 128.9, 128.9, 128.7, 128.6, 128.6, 128.5, 128.4, 128.3, 128.1, 128.0, 126.6, 102.0, 101.2, 99.4, 83.6, 81.3, 79.4, 78.4, 78.0, 77.7, 75.4, 75.4, 75.4, 74.5, 74.2, 73.9, 69.5, 68.7, 62.5, 55.8, 21.5.

**[α]$_D^{25}$** = -6.0 (c = 0.20, CHCl$_3$).

**HRMS** (ESI-TOF): calculated for $C_{50}H_{54}O_{12}Na^+$ [M+Na$^+$]: 869.3507, found 869.3490.

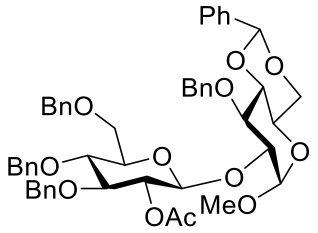

**3s**

**(2*R*,3*S*,4*R*,5*S*,6*S*)-4,5-Bis(benzyloxy)-2-(((2S,6S,7R,8S)-8-(benzyloxy)-6-methoxy-2-phenylhexahydropyrano[3,2-d][1,3]dioxin-7-yl)oxy)-6-((benzyloxy)methyl)tetrahydro-2*H*-pyran-3-yl acetate**

The title compound was synthesized according to **general procedure D** using glycosyl donor **1a** (45.4 mg, 0.075 mmol) and acceptor **2s** (18.6 mg, 0.05 mmol) as reactants and **D** (3.3 mg, 20 mol%) as the catalyst. The reaction mixture was stirred at 40 °C for 12 h and then purified by column chromatography on silica gel with petroleum ether/ethyl acetate (5:1) as eluent to afford **3s** as a white solid (39.8 mg, 94% yield).

**$^{1}$H NMR (400 MHz, CDCl$_3$)** δ 7.46 – 7.44 (m, 2H), 7.36 – 7.26 (m, 20H), 7.19 – 7.17 (m, 3H), 5.54 (s, 1H), 5.10 (t, *J* = 8.5 Hz, 1H), 4.92 (d, *J* = 3.6 Hz, 1H), 4.81 – 4.77 (m, 3H), 4.71 – 4.64 (m, 3H), 4.59 – 4.50 (m, 3H), 4.30 – 4.26 m, 1H), 3.98 (t, *J* = 9.4 Hz, 1H), 3.88 – 3.82 (m, 1H), 3.75 – 3.56 (m, 7H), 3.50 – 3.48 (m, 1H), 3.39 (s, 3H), 1.75 (s, 3H).

**$^{13}$C NMR (101 MHz, CDCl$_3$)** δ 169.9, 139.1, 138.5, 138.4, 138.2, 137.8, 129.4, 128.9, 128.9, 128.9, 128.8, 128.7, 128.6, 128.4, 128.4, 128.2, 128.2, 128.2, 128.0, 126.5, 102.6, 101.8, 100.8, 83.6, 82.7, 80.5, 78.4, 78.0, 77.7, 75.5, 75.5, 75.4, 75.3, 75.3, 73.9, 73.5, 69.6, 69.4, 62.7, 55.9, 21.3.

**$[\alpha]_D^{25}$** = +12.4 (c = 0.25, CHCl$_3$).

**HRMS** (ESI-TOF): calculated for $C_{50}H_{54}O_{12}Na^+$ [M+Na$^+$]: 869.3507, found 869.3494.

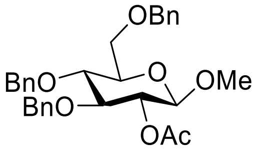

**3t**

**(2*R*,3*R*,4*S*,5*R*,6*R*)-4,5-Bis(benzyloxy)-6-((benzyloxy)methyl)-2-methoxytetrahydro-2*H*-pyran-3-yl acetate**

The title compound was synthesized according to **general procedure D** using glycosyl donor **1a** (45.4 mg, 0.075 mmol) and acceptor **2t** (2.03 μL, 0.05 mmol) as reactants and **F** (4.0 mg, 20 mol%) as the catalyst. The reaction mixture was stirred at 40 °C for 12 h and then purified by column chromatography on silica gel with petroleum

ether/ethyl acetate (7:1) as eluent to afford **3t** as a colorless syrup (23.5 mg, 93% yield).

**$^{1}$H NMR (400 MHz, CDCl$_3$)** δ 7.34 – 7.25 (m, 13H), 7.19 – 7.17 (m, 2H), 5.00 (t, *J* = 8.3 Hz, 1H), 4.81 – 4.77 (m, 2H), 4.68 – 4.62 (m, 2H), 4.58 – 4.54 (m, 2H), 4.29 (d, *J* = 7.9 Hz, 1H), 3.78 – 3.64 (m, 4H), 3.51 – 3.48 (m, 4H), 1.97 (s, 3H).

**$^{13}$C NMR (101 MHz, CDCl$_3$)** δ 169.7, 138.3, 138.3, 138.1, 128.6, 128.5, 128.2, 128.0, 127.9, 127.9, 127.8, 101.9, 83.2, 78.2, 75.4, 75.2, 73.7, 73.2, 68.9, 56.7, 21.1.

**$[\alpha]_D^{25}$** = +2.6 (c = 0.43, $CHCl_3$).

**HRMS** (ESI-TOF): calculated for $C_{30}H_{34}O_7Na^+$ [M+Na$^+$]: 529.2197, found 529.2188.

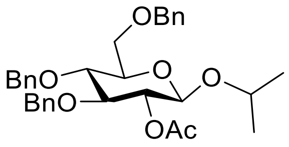


**3u**

**(2*R*,3*R*,4S,5R,6R)-4,5-bis(benzyloxy)-6-((benzyloxy)methyl)-2-isopropoxytetrahydro-2*H*-pyran-3-yl acetate**

The title compound was synthesized according to **general procedure D** using glycosyl donor **1a** (45.4 mg, 0.075 mmol) and acceptor **2u** (3.82 μL, 0.05 mmol) as reactants and **D** (0.8 mg, 5 mol%) as the catalyst. The reaction mixture was stirred at room temperature for 12 h and then purified by column chromatography on silica gel with petroleum ether/ethyl acetate (9:1) as eluent to afford **3u** as a colorless syrup (22.4 mg, 84% yield).

**$^{1}$H NMR (400 MHz, CDCl$_3$)** δ 7.32 – 7.16 (m, 15H), 4.94 – 4.90 (m, 1H), 4.78 – 4.75 (m, 2H), 4.66 – 4.53 (m, 4H), 4.37 (d, *J* = 8.0 Hz, 1H), 3.90 – 3.84 (m, 1H), 3.74 – 3.59 (m, 4H), 3.47 – 3.44 (m, 1H), 1.93 (s, 3H), 1.21 (d, *J* = 6.2 Hz, 3H), 1.09 (d, *J* = 6.1 Hz, 3H).

**$^{13}$C NMR (101 MHz, CDCl$_3$)** δ 169.6, 138.4, 138.1, 128.6, 128.6, 128.5, 128.2, 128.0, 128.0, 127.8, 127.7, 100.1, 83.2, 78.3, 75.3, 75.2, 75.1, 73.6, 73.6, 72.6, 69.1, 23.6, 22.2, 21.0.

**$[\alpha]_D^{25}$** = +6.1 (c = 0.33, $CHCl_3$).

**HRMS** (ESI-TOF): calculated for $C_{32}H_{38}O_7Na^+$ [M+Na$^+$]: 557.2510, found 557.2499.

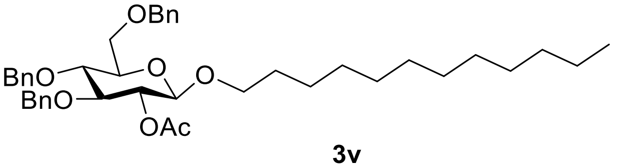


**3v**

**(2*R*,3*R*,4*S*,5*R*,6*R*)-4,5-Bis(benzyloxy)-6-((benzyloxy)methyl)-2-(dodecyloxy)tetrahydro-2*H*-pyran-3-yl acetate**

The title compound was synthesized according to **general procedure D** using glycosyl donor **1a** (45.4 mg, 0.075 mmol) and acceptor **2v** (9.3 mg, 0.05 mmol) as reactants and **D** (3.3 mg, 20 mol%) as the catalyst. The reaction mixture was stirred at 40 °C for 12 h and then purified by column chromatography on silica gel with petroleum ether/ethyl acetate (13:1) as eluent to afford **3v** as a colorless syrup (26.4 mg, 80% yield).

**$^1$H NMR (400 MHz, CDCl$_3$)** δ 7.32 – 7.15 (m, 15H), 4.96 (t, *J* = 8.4 Hz, 1H), 4.78 – 4.75 (m, 2H), 4.66 – 4.52 (m, 4H), 4.32 (d, *J* = 8.0 Hz, 1H), 3.87 – 3.81 (m, 1H), 3.74 – 3.61 (m, 4H), 3.48 – 3.38 (m, 2H), 1.94 (s, 3H), 1.55 – 1.50 (m, 2H), 1.23 (s, 18H), 0.86 (t, *J* = 6.7 Hz, 3H).

**$^{13}$C NMR (101 MHz, CDCl$_3$)** δ 169.9, 138.7, 138.6, 138.4, 128.9, 128.8, 128.5, 128.3, 128.2, 128.2, 128.1, 128.0, 101.5, 83.5, 78.6, 75.7, 75.5, 75.4, 74.0, 73.7, 70.2, 69.3, 32.4, 30.1, 30.1, 30.0, 29.8, 29.8, 26.4, 23.1, 21.3, 14.6.

**$[\alpha]_D^{25}$** = +4.1 (c = 0.17, CHCl$_3$).

**HRMS** (ESI-TOF): calculated for $C_{41}H_{56}O_7Na^+$ [M+Na$^+$]: 683.3918, found 683.3906.

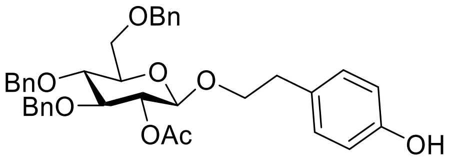


**3w**

**(2*R*,3*R*,4*S*,5*R*,6*R*)-4,5-Bis(benzyloxy)-6-((benzyloxy)methyl)-2-(4-hydroxyphenethoxy)tetrahydro-2*H*-pyran-3-yl acetate**

The title compound was synthesized according to **general procedure D** using glycosyl donor **1a** (45.4 mg, 0.075 mmol) and acceptor **2w** (6.9 mg, 0.05 mmol) as reactants and **D** (3.3 mg, 20 mol%) as the catalyst. The reaction mixture was stirred at 40 °C for 12 h and then purified by column chromatography on silica gel with petroleum ether/ethyl acetate (3:1) as eluent to afford **3w** as a colorless syrup (26.0 mg, 85% yield).

**$^1$H NMR (400 MHz, DMSO-*d*$_6$)** δ 9.18 (s, 1H), 7.33 – 7.21 (m, 15H), 6.98 – 6.96 (m, 2H), 6.65 (d, *J* = 7.3 Hz, 2H), 4.72 – 4.69 (m, 3H), 4.59 – 4.48 (m, 5H), 3.87 – 3.84 (m, 1H), 3.64 – 3.49 (m, 6H), 2.67 (s, 2H), 1.87 (s, 3H).

**$^{13}$C NMR (101 MHz, DMSO-*d*$_6$)** δ 169.5, 156.0, 138.7, 138.7, 138.5, 130.1, 129.1, 128.7, 128.3, 128.1, 128.1, 128.0, 127.9, 115.4, 100.1, 82.4, 78.4, 74.6, 74.5, 74.4, 73.1, 72.7, 70.3, 69.0, 34.9, 21.0.

**$[\alpha]_D^{25}$** = +6.2 (c = 0.29, CHCl$_3$).

**HRMS** (ESI-TOF): calculated for $C_{37}H_{40}O_8Na^+$ [M+Na$^+$]: 635.2615, found 635.2614.

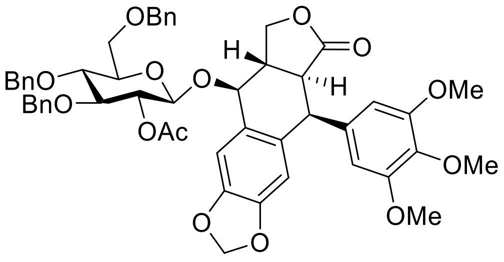

**3x**

**(2*R*,3*R*,4*S*,5*R*,6*R*)-4,5-Bis(benzyloxy)-6-((benzyloxy)methyl)-2-(((5*S*,5a*S*,8a*S*,9*S*)-8-oxo-9-(3,4,5-trimethoxyphenyl)-5,5a,6,8,8a,9-hexahydrofuro[3',4':6,7]naphtho[2,3-d][1,3]dioxol-5-yl)oxy)tetrahydro-2*H*-pyran-3-yl acetate**

The title compound was synthesized according to **general procedure D** using glycosyl donor **1a** (45.4 mg, 0.075 mmol) and acceptor **2x** (20.7 mg, 0.05 mmol) as reactants and **F** (4.0 mg, 20 mol%) as the catalyst. The reaction mixture was stirred at 40 °C for 12 h and then purified by column chromatography on silica gel with petroleum ether/ethyl acetate (3:1) as eluent to afford **3x** as a white solid (44.0 mg, 99% yield).

**$^1$H NMR (400 MHz, $CDCl_3$)** δ 7.30 – 7.20 (m, 13H), 7.17 – 7.15 (m, 3H), 6.45 (s, 1H), 6.31 (s, 2H), 5.95 – 5.92 (m, 2H), 5.02 (t, *J* = 8.4 Hz, 1H), 4.84 (d, *J* = 9.6 Hz, 1H), 4.77 – 4.74 (m, 2H), 4.61 (d, *J* = 11.5 Hz, 1H), 4.55 – 4.45 (m, 5H), 4.40 (d, *J* = 8.0 Hz, 1H), 4.02 (t, *J* = 9.5 Hz, 1H), 3.73 – 3.70 (m, 9H), 3.65 – 3.57 (m, 4H), 3.44 – 3.41 (m, 1H), 2.89 – 2.78 (m, 1H), 2.75 – 2.70 (m, 1H), 1.89 (s, 3H).

**$^{13}$C NMR (101 MHz, $CDCl_3$)** δ 173.8, 169.0, 152.4, 147.7, 147.3, 137.8, 137.7, 137.5, 137.0, 137.0, 135.0, 131.8, 129.8, 128.3, 128.2, 127.8, 127.8, 127.6, 127.6, 127.5, 127.4, 109.2, 108.0, 101.2, 99.0, 82.8, 78.8, 77.7, 75.0, 74.9, 74.8, 73.3, 72.9, 71.1, 68.6, 60.5, 56.1, 45.3, 43.7, 38.6, 20.7.

**$[\alpha]_D^{25}$** = -23.6 (c = 0.25, $CHCl_3$).

**HRMS** (ESI-TOF): calculated for $C_{51}H_{52}O_{14}Na^+$ [M+Na$^+$]: 911.3249, found 911.3235.

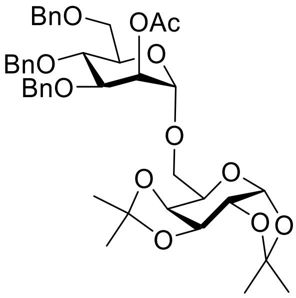


**3y**

**(2*R*,3*S*,4*S*,5*R*,6*R*)-4,5-Bis(benzyloxy)-6-((benzyloxy)methyl)-2-(((3aR,5R,5aS,8aS,8bR)-2,2,7,7-tetramethyltetrahydro-5*H*-bis([1,3]dioxolo)[4,5-b:4',5'-d]pyran-5-yl)methoxy)tetrahydro-2*H*-pyran-3-yl acetate**

The title compound was synthesized according to **general procedure D** using glycosyl donor **1b** (45.4 mg, 0.075 mmol) and acceptor **2l** (13.0 mg, 0.05 mmol) as reactants and **F** (4.0 mg, 20 mol%) as the catalyst. The reaction mixture was stirred at 40 °C for 12 h and then purified by column chromatography on silica gel with petroleum ether/ethyl acetate (7:1) as eluent to afford **3y** as a colorless syrup (32.7 mg, 89% yield).

**$^{1}$H NMR (400 MHz, CDCl$_3$)** δ 7.36 – 7.25 (m, 13H), 7.15 – 7.14 (m, 2H), 5.50 (d, *J* = 5.0 Hz, 1H), 5.40 – 5.39 (m, 1H), 4.90 (d, *J* = 1.8 Hz, 1H), 4.83 (d, *J* = 10.7 Hz, 1H), 4.70 (d, *J* = 11.4 Hz, 2H), 4.61 – 4.59 (m, 1H), 4.54 – 4.45 (m, 3H), 4.31 – 4.29 (m, 1H), 4.22 – 4.20 (m, 1H), 4.00 – 3.90 (m, 3H), 3.83 – 3.75 (m, 3H), 3.71 – 3.67 (m, 2H), 2.14 (s, 3H), 1.51 (s, 3H), 1.42 (s, 3H), 1.34 (s, 3H), 1.32 (s, 3H).

**$^{13}$C NMR (101 MHz, CDCl$_3$)** δ 170.9, 138.9, 138.7, 138.5, 128.8, 128.8, 128.5, 128.4, 128.3, 128.2, 128.1, 128.0, 109.8, 109.1, 98.4, 96.7, 78.6, 75.6, 74.7, 73.9, 72.3, 72.0, 71.3, 71.1, 71.1, 69.2, 69.2, 66.6, 66.5, 26.6, 26.4, 25.4, 25.0, 21.6.

**$[\alpha]_D^{25}$** = -1.7 (c = 0.17, CHCl$_3$).

**HRMS** (ESI-TOF): calculated for $C_{41}H_{50}O_{12}Na^+$ [M+Na$^+$]: 757.3194, found 757.3176.

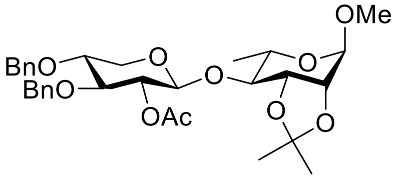


**3z**

**(2*S*,3*R*,4*S*,5*R*)-4,5-Bis(benzyloxy)-2-(((3a*R*,4*R*,6*S*,7*S*,7a*R*)-4-methoxy-2,2,6-trimethyltetrahydro-4*H*-[1,3]dioxolo[4,5-c]pyran-7-yl)oxy)tetrahydro-2*H*-pyran-3-yl acetate**

The title compound was synthesized according to **general procedure D** using

glycosyl donor **1d** (36.4 mg, 0.075 mmol) and acceptor **2q** (10.9 mg, 0.05 mmol) as reactants and **F** (4.0 mg, 20 mol%) as the catalyst. The reaction mixture was stirred at 40 °C for 12 h and then purified by column chromatography on silica gel with petroleum ether/ethyl acetate (10:1) as eluent to afford **3z** as a colorless syrup (26.6 mg, 93% yield).

**$^1$H NMR (400 MHz, CDCl$_3$)** δ 7.27 – 7.21 (m, 10H), 4.84 – 4.75 (m, 4H), 4.65 – 4.63 (m, 2H), 4.56 (d, *J* = 11.8 Hz, 1H), 4.00 – 3.92 (m, 3H), 3.65 – 3.44 (m, 4H), 3.29 (s, 3H), 3.20 (d, *J* = 10.9 Hz, 1H), 1.95 (s, 3H), 1.46 (s, 3H), 1.28 (s, 3H), 1.18 (d, *J* = 5.8 Hz, 3H).

**$^{13}$C NMR (101 MHz, CDCl$_3$)** δ 169.6, 138.2, 137.8, 128.3, 128.2, 127.7, 127.6, 127.6, 127.4, 109.1, 99.7, 97.6, 80.9, 78.3, 78.2, 77.5, 75.8, 74.3, 72.9, 72.5, 63.7, 63.3, 54.6, 27.7, 26.2, 20.8, 17.3.

**$[\alpha]_D^{25}$** = -11.5 (c = 0.59, CHCl$_3$).

**HRMS** (ESI-TOF): calculated for $C_{31}H_{40}O_{10}Na^+$ [M+Na$^+$]: 595.2514, found 595.2502.

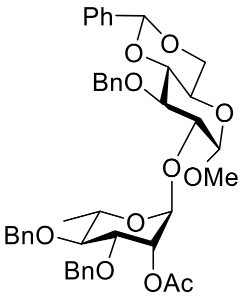


**3aa**

**(2*S*,3*R*,4*R*,5*S*,6*S*)-4,5-Bis(benzyloxy)-2-(((2*R*,4a*R*,6*S*,7*R*,8*S*,8a*R*)-8-(benzyloxy)-6-methoxy-2-phenylhexahydropyrano[3,2-d][1,3]dioxin-7-yl)oxy)-6-methyltetrahydro-2*H*-pyran-3-yl acetate**

The title compound was synthesized according to **general procedure D** using glycosyl donor **1f** (25 mg, 0.075 mmol) and acceptor **2s** (18.6 mg, 0.05 mmol) as reactants and **F** (4.0 mg, 20 mol%) as the catalyst stirring at 50 °C. The reaction mixture was stirred at 50 °C for 12 h and then purified by column chromatography on silica gel with petroleum ether/ethyl acetate (7:1) as eluent to afford **3aa** as a colorless syrup (28.1 mg, 76% yield).

**$^1$H NMR (400 MHz, CDCl$_3$)** δ 7.53 – 7.50 (m, 2H), 7.44 – 7.26 (m, 18H), 5.60 (s, 2H), 5.06 (s, 1H), 4.94 (d, *J* = 10.8 Hz, 1H), 4.87 (d, *J* = 11.2 Hz, 1H), 4.81 – 4.73 (m, 3H), 4.64 (d, *J* = 10.8 Hz, 1H), 4.54 (d, *J* = 11.0 Hz, 1H), 4.33 – 4.30 (m, 1H), 4.03 – 3.98 (m, 2H), 3.91 – 3.73 (m, 4H), 3.65 (t, *J* = 9.2 Hz, 1H), 3.46 (t, *J* = 9.5 Hz, 1H), 3.43 (s, 3H), 2.15 (s, 3H), 1.35 (d, *J* = 6.3 Hz, 3H).

**$^{13}$C NMR (101 MHz, $CDCl_3$)** δ 170.1, 138.6, 138.5, 138.2, 137.5, 129.1, 128.5, 128.5, 128.4, 128.3, 128.2, 128.2, 127.9, 127.8, 126.2, 101.5, 100.5, 99.9, 82.5, 80.0, 79.3, 78.2, 77.9, 75.6, 75.4, 72.1, 69.2, 69.0, 68.5, 62.4, 55.4, 21.2, 18.2.

**$[\alpha]_D^{25}$** = +11.0 (c = 0.19, $CHCl_3$).

**HRMS** (ESI-TOF): calculated for $C_{43}H_{48}O_{11}Na^+$ [M+Na$^+$]: 763.3089, found 763.3076.

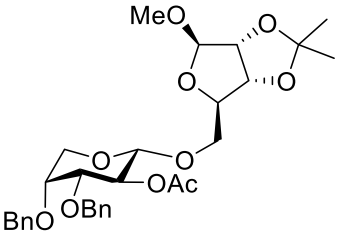


**3ab**

**(2*S*,3*S*,4*R*,5*R*)-4,5-Bis(benzyloxy)-2-(((3a*R*,4*R*,6*R*,6a*R*)-6-methoxy-2,2-dimethyltetrahydrofuro[3,4-d][1,3]dioxol-4-yl)methoxy)tetrahydro-2*H*-pyran-3-yl acetate**

The title compound was synthesized according to **general procedure D** using glycosyl donor **1e** (72.7 mg, 0.15 mmol) and acceptor **2n** (10.2 mg, 0.05 mmol) as reactants and **F** (4.0 mg, 20 mol%) as the catalyst. The reaction mixture was stirred at 40 °C for 12 h and then purified by column chromatography on silica gel with petroleum ether/ethyl acetate (5:1) as eluent to afford **3ab** as a colorless syrup (21.0 mg, 75% yield).

**$^{1}$H NMR (400 MHz, $CDCl_3$)** δ 7.28 – 7.18 (m, 10H), 5.22 – 5.19 (m, 1H), 4.86 (s, 1H), 4.60 – 4.51 (m, 5H), 4.46 (d, *J* = 5.9 Hz, 1H), 4.35 (d, *J* = 5.0 Hz, 1H), 4.28 – 4.25 (m, 1H), 4.03 – 3.99 (m, 1H), 3.77 – 3.73 (m, 1H), 3.68 – 3.65 (m, 1H), 3.55 – 3.52 (m, 1H), 3.38 – 3.30 (m, 2H), 3.21 (s, 3H), 1.98 (s, 3H), 1.39 (s, 3H), 1.21 (s, 3H).

**$^{13}$C NMR (101 MHz, $CDCl_3$)** δ 169.7, 138.2, 138.2, 128.5, 128.4, 127.9, 127.8, 127.8, 127.7, 112.4, 109.5, 100.4, 85.3, 84.9, 82.1, 76.1, 71.7, 71.7, 71.3, 70.1, 69.6, 61.2, 55.0, 26.6, 25.1, 21.0.

**$[\alpha]_D^{25}$** = -2.2 (c = 0.18, $CHCl_3$).

**HRMS** (ESI-TOF): calculated for $C_{30}H_{38}O_{10}Na^+$ [M+Na$^+$]: 581.2357, found 581.2350.

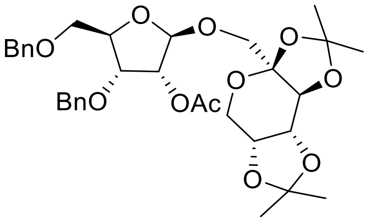

**3ac**

**(2*R*,3*R*,4*R*,5*R*)-4-(benzyloxy)-5-((benzyloxy)methyl)-2-(((3a*S*,5a*S*,8a*R*,8b*S*)-2,2,7,7-tetramethyltetrahydro-3aH-bis([1,3]dioxolo)[4,5-b:4',5'-d]pyran-3a-yl)methoxy)tetrahydrofuran-3-yl acetate**

The title compound was synthesized according to **general procedure D** using glycosyl donor **1g** (36.4 mg, 0.075 mmol) and acceptor **2m** (13.0 mg, 0.05 mmol) as reactants and **F** (4.0 mg, 20 mol%) as the catalyst. The reaction mixture was stirred at 40 °C for 12 h and then purified by column chromatography on silica gel with petroleum ether/ethyl acetate (5:1) as eluent to afford **3ac** as a colorless syrup (30.4 mg, 99% yield).

**$^1$H NMR (400 MHz, CDCl$_3$)** δ 7.35 – 7.24 (m, 10H), 5.30 (d, *J* = 4.4 Hz, 1H), 5.10 (s, 1H), 4.60 – 4.51 (m, 4H), 4.41 (d, *J* = 11.5 Hz, 1H), 4.26 – 4.14 (m, 4H), 3.88 (d, *J* = 12.0 Hz, 1H), 3.73 – 3.70 (m, 2H), 3.64 – 3.61 (m, 2H), 3.54 – 3.50 (m, 1H), 2.11 (s, 3H), 1.52 (s, 3H), 1.42 (s, 3H), 1.34 (s, 3H), 1.31 (s, 3H).

**$^{13}$C NMR (101 MHz, CDCl$_3$)** δ 170.1, 138.4, 137.6, 128.5, 128.5, 128.1, 128.1, 127.7, 127.6, 109.1, 108.7, 105.8, 102.5, 80.7, 78.3, 74.0, 73.3, 73.2, 71.4, 71.1, 70.3, 70.1, 68.5, 61.3, 26.7, 26.1, 25.5, 24.2, 21.0.

**$[\alpha]_D^{25}$** = -6.3 (c = 0.30, CHCl$_3$).

**HRMS** (ESI-TOF): calculated for $C_{33}H_{42}O_{11}Na^+$ [M+Na$^+$]: 637.2619, found 637.2607.

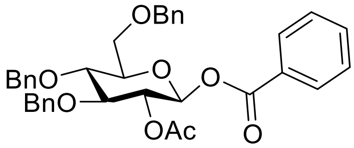

**3ad**

**(2*S*,3*R*,4*S*,5*R*,6*R*)-3-Acetoxy-4,5-bis(benzyloxy)-6-((benzyloxy)methyl)tetrahydro-2*H*-pyran-2-yl benzoate**

The title compound was synthesized according to **general procedure D** using glycosyl donor **1a** (45.4 mg, 0.075 mmol) and acceptor **2ad** (6.1 mg, 0.05 mmol) as reactants and **D** (3.3 mg, 20 mol%) as the catalyst. The reaction mixture was stirred at 40 °C for 12 h and then purified by column chromatography on silica gel with petroleum ether/ethyl acetate (6:1) as eluent to afford **3ad** as a white solid (26.5 mg, 89% yield).

**$^1$H NMR (400 MHz, CDCl$_3$)** δ 8.06 (d, *J* = 7.7 Hz, 2H), 7.59 (t, *J* = 7.4 Hz, 1H), 7.45 (t, *J* = 7.7 Hz, 2H), 7.34 – 7.26 (m, 13H), 7.20 – 7.17 (m, 2H), 5.82 (d, *J* = 8.2 Hz,

1H), 5.34 (t, $J$ = 8.8 Hz, 1H), 4.87 – 4.81 (m, 2H), 4.73 (d, $J$ = 11.5 Hz, 1H), 4.65 – 4.57 (m, 2H), 4.50 (d, $J$ = 12.1 Hz, 1H), 3.92 (t, $J$ = 9.3 Hz, 1H), 3.84 – 3.75 (m, 3H), 3.73 – 3.69 (m, 1H), 1.89 (s, 3H).

**$^{13}$C NMR (101 MHz, CDCl$_3$)** δ 169.6, 164.7, 164.1, 138.2, 138.0, 132.4, 128.6, 128.6, 128.5, 128.1, 128.0, 128.0, 127.9, 127.9, 127.8, 121.3, 113.9, 92.8, 82.9, 77.5, 75.9, 75.2, 75.2, 72.3, 68.2, 55.6, 20.9.

**$[\alpha]_D^{25}$** = +0.6 (c = 0.13, CHCl$_3$).

**HRMS** (ESI-TOF): calculated for $C_{36}H_{36}O_8Na^+$ [M+Na$^+$]: 619.2302, found 619.2286.

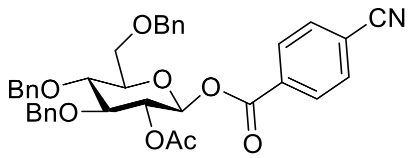

**3ae**

**(2*S*,3*R*,4*S*,5*R*,6*R*)-3-Acetoxy-4,5-bis(benzyloxy)-6-((benzyloxy)methyl)tetrahydro-2*H*-pyran-2-yl 4-cyanobenzoate**

The title compound was synthesized according to **general procedure D** using glycosyl donor **1a** (45.4 mg, 0.075 mmol) and acceptor **2ae** (7.4 mg, 0.05 mmol) as reactants and **D** (3.3 mg, 20 mol%) as the catalyst. The reaction mixture was stirred at 40 °C for 12 h and then purified by column chromatography on silica gel with petroleum ether/ethyl acetate (6:1) as eluent to afford **3ae** as a white solid (28.3 mg, 91% yield).

**$^1$H NMR (400 MHz, CDCl$_3$)** δ 8.14 (d, $J$ = 8.1 Hz, 2H), 7.74 (d, $J$ = 8.1 Hz, 2H), 7.37 – 7.26 (m, 13H), 7.19 – 7.17 (m, 2H), 5.82 (d, $J$ = 8.0 Hz, 1H), 5.32 (t, $J$ = 8.6 Hz, 1H), 4.87 – 4.81 (m, 2H), 4.73 (d, $J$ = 11.5 Hz, 1H), 4.64 – 4.56 (m, 2H), 4.49 (d, $J$ = 12.0 Hz, 1H), 3.91 (t, $J$ = 9.3 Hz, 1H), 3.82 (d, $J$ = 9.0 Hz, 1H), 3.78 – 3.75 (m, 2H), 3.71 (d, $J$ = 9.7 Hz, 1H), 1.89 (s, 3H).

**$^{13}$C NMR (101 MHz, CDCl$_3$)** δ 169.6, 163.5, 138.1, 137.8, 137.8, 132.8, 132.4, 130.7, 128.6, 128.6, 128.5, 128.1, 128.0, 128.0, 127.9, 127.9, 117.9, 117.2, 93.5, 82.6, 77.3, 76.0, 75.2, 75.2, 73.7, 72.2, 68.1, 20.8.

**$[\alpha]_D^{25}$** = -271.6 (c = 0.28 CHCl$_3$).

**HRMS** (ESI-TOF): calculated for $C_{37}H_{35}NO_8Na^+$ [M+Na$^+$]: 644.2255, found 644.2241.

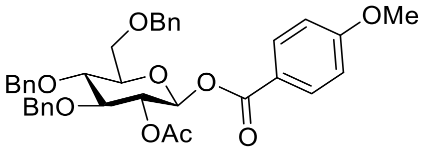


**3af**

**(2*S*,3*R*,4*S*,5*R*,6*R*)-3-Acetoxy-4,5-bis(benzyloxy)-6-((benzyloxy)methyl)tetrahydro-2*H*-pyran-2-yl 4-methoxybenzoate**

The title compound was synthesized according to **general procedure D** using glycosyl donor **1a** (45.4 mg, 0.075 mmol) and acceptor **2af** (7.6 mg, 0.05 mmol) as reactants and **D** (3.3 mg, 20 mol%) as the catalyst. The reaction mixture was stirred at 40 °C for 12 h and then purified by column chromatography on silica gel with petroleum ether/ethyl acetate (5:1) as eluent to afford **3af** as a white solid (28.8 mg, 92% yield).

**$^{1}$H NMR (400 MHz, CDCl$_3$)** δ 8.01 – 7.98 (m, 2H), 7.34 – 7.24 (m, 13H), 7.18 – 7.15 (m, 2H), 6.92 – 6.90 (m, 2H), 5.79 – 5.76 (m, 1H), 5.30 (t, *J* = 8.8 Hz, 1H), 4.85 – 4.79 (m, 2H), 4.71 (d, *J* = 11.3 Hz, 1H), 4.64 – 4.56 (m, 2H), 4.49 (d, *J* = 12.1 Hz, 1H), 3.90 (d, *J* = 9.3 Hz, 1H), 3.86 (s, 3H), 3.81 – 3.74 (m, 3H), 3.68 (d, *J* = 8.9 Hz, 1H), 1.87 (s, 3H).

**$^{13}$C NMR (101 MHz, CDCl$_3$)** δ 170.0, 165.1, 164.4, 138.6, 138.3, 132.8, 128.9, 128.9, 128.8, 128.4, 128.4, 128.3, 128.2, 128.2, 128.1, 121.6, 114.2, 93.1, 83.2, 77.8, 76.3, 75.5, 75.5, 74.0, 72.6, 68.5, 55.9, 21.2.

**$[\alpha]_D^{25}$** = -7.6 (c = 0.25, CHCl$_3$).

**HRMS** (ESI-TOF): calculated for $C_{37}H_{38}O_9Na^+$ [M+Na$^+$]: 649.2408, found 649.2395.

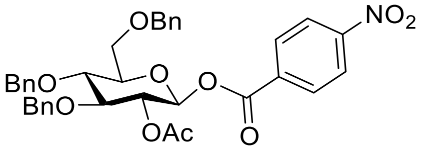


**3ag**

**(2*S*,3*R*,4*S*,5*R*,6*R*)-3-Acetoxy-4,5-bis(benzyloxy)-6-((benzyloxy)methyl)tetrahydro-2*H*-pyran-2-yl 4-nitrobenzoate**

The title compound was synthesized according to **general procedure D** using glycosyl donor **1a** (45.4 mg, 0.075 mmol) and acceptor **2ag** (8.4 mg, 0.05 mmol) as reactants and **D** (3.3 mg, 20 mol%) as the catalyst. The reaction mixture was stirred at 40 °C for 12 h and then purified by column chromatography on silica gel with petroleum ether/ethyl acetate (5:1) as eluent to afford **3ag** as a white solid (29.2 mg, 91% yield).

**$^{1}$H NMR (400 MHz, CDCl$_3$)** δ 8.28 – 8.27 (m, 2H), 8.23 – 8.20 (m, 2H), 7.37 – 7.25 (m, 13H), 7.20 – 7.17 (m, 2H), 5.85 – 5.83 (m, 1H), 5.36 – 5.31 (m, 1H), 4.89 –

4.81 (m, 2H), 4.76 – 4.72 (m, 1H), 4.64 – 4.57 (m, 2H), 4.52 – 4.48 (m, 1H), 3.95 – 3.90 (m, 1H), 3.87 – 3.77 (m, 3H), 3.75 – 3.70 (m, 1H), 1.90 (s, 3H).

**$^{13}$C NMR (101 MHz, CDCl$_3$)** δ 169.6, 163.2, 151.0, 138.1, 137.8, 137.8, 134.4, 131.4, 128.6, 128.6, 128.5, 128.1, 128.1, 128.0, 127.9, 127.9, 123.8, 93.5, 82.6, 77.2, 76.0, 75.3, 75.2, 73.7, 72.2, 68.0, 20.8.

**$[\alpha]_D^{25}$** = -5.2 (c = 0.19, CHCl$_3$).

**HRMS** (ESI-TOF): calculated for $C_{36}H_{35}NO_{10}Na^+$ [M+Na$^+$]: 664.2153, found 664.2137.

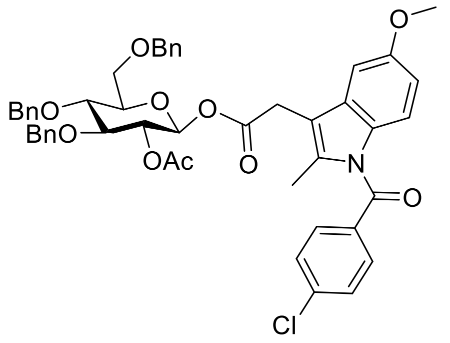


**3ah**

**(2*S*,3*R*,4*S*,5*R*,6*R*)-3-Acetoxy-4,5-bis(benzyloxy)-6-((benzyloxy)methyl)tetrahydro-2*H*-pyran-2-yl 2-(1-(4-chlorobenzoyl)-5-methoxy-2-methyl-1*H*-indol-3-yl)acetate**

The title compound was synthesized according to **general procedure D** using glycosyl donor **1a** (45.4 mg, 0.075 mmol) and acceptor **2ah** (17.9 mg, 0.05 mmol) as reactants and **D** (3.3 mg, 20 mol%) as the catalyst. The reaction mixture was stirred at 40 °C for 12 h and then purified by column chromatography on silica gel with petroleum ether/ethyl acetate (5:1) as eluent to afford **3ah** as a white solid (41.1 mg, 99% yield).

**$^{1}$H NMR (400 MHz, CDCl$_3$)** δ 7.73 (d, *J* = 8.1 Hz, 2H), 7.50 (d, *J* = 8.1 Hz, 2H), 7.37 – 7.26 (m, 13H), 7.20 – 7.18 (m, 2H), 7.03 – 6.97 (m, 2H), 6.74 – 6.71 (m, 1H), 5.65 (d, *J* = 8.2 Hz, 1H), 5.12 (t, *J* = 8.8 Hz, 1H), 4.82 – 4.79 (m, 2H), 4.67 – 4.64 (m, 2H), 4.59 – 4.52 (m, 2H), 3.87 (s, 3H), 3.82 – 3.63 (m, 7H), 2.37 (s, 3H), 1.51 (s, 3H).

**$^{13}$C NMR (126 MHz, CDCl$_3$)** δ 169.3, 169.2, 168.4, 156.2, 139.2, 138.0, 137.8, 137.8, 136.2, 134.0, 131.2, 130.8, 130.3, 129.2, 128.5, 128.5, 128.0, 128.0, 127.9, 127.8, 115.1, 112.0, 111.6, 101.0, 92.7, 82.6, 77.3, 75.9, 75.2, 75.1, 73.6, 71.8, 68.0, 55.7, 30.5, 20.1, 13.5.

**$[\alpha]_D^{25}$** = -7.7 (c = 0.20, CHCl$_3$).

**HRMS** (ESI-TOF): calculated for $C_{48}H_{46}ClNO_{10}Na^+$ [M+Na$^+$]: 854.2702, found 854.2679.

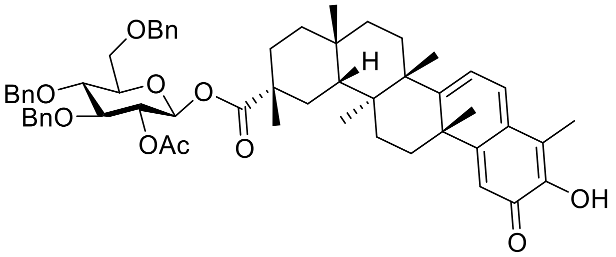

**3ai**

**(2*S*,3*R*,4*S*,5*R*,6*R*)-3-Acetoxy-4,5-bis(benzyloxy)-6-((benzyloxy)methyl)tetrahydro-2*H*-pyran-2-yl (2*R*,4a*S*,6a*S*,12b*R*,14a*S*,14b*R*)-10-hydroxy-2,4a,6a,9,12b,14a-hexamethyl-11-oxo-1,2,3,4,4a,5,6,6a,11,12b,13,14,14a,14b-tetradecahydropicene-2-carboxylate**

The title compound was synthesized according to **general procedure D** using glycosyl donor **1a** (45.4 mg, 0.075 mmol) and acceptor **2ai** (22.5 mg, 0.05 mmol) as reactants and **D** (3.3 mg, 20 mol%) as the catalyst. The reaction mixture was stirred at 40 °C for 12 h and then purified by column chromatography on silica gel with petroleum ether/ethyl acetate (5:1) as eluent to afford **3ai** as a white solid (17.1 mg, 37% yield, α/β = 1:5.6).

**$^{1}$H NMR (400 MHz, CDCl$_3$)** δ 7.67 – 7.63 (m, 1H), 7.42 – 7.40 (m, 1H), 7.35 – 7.20 (m, 16H), 6.25 (s, 1H), 5.31 (t, *J* = 8.6 Hz, 1H), 5.18 (d, *J* = 7.7 Hz, 1H), 4.88 – 4.76 (m, 3H), 4.61 – 4.57 (m, 2H), 4.51 (d, *J* = 12.0 Hz, 1H), 3.98 (t, *J* = 9.3 Hz, 1H), 3.82 – 3.67 (m, 4H), 2.96 – 2.88 (m, 1H), 2.77 – 2.69 (m, 1H), 2.52 (s, 3H), 2.41 (s, 3H), 2.27 – 2.21 (m, 1H), 2.02 (s, 3H), 2.02 – 1.89 (m, 2H), 1.77 – 1.71 (m, 1H), 1.65 – 1.60 (m, 2H), 1.54 – 1.48 (m, 2H), 1.46 – 1.40 (m, 2H), 1.37 – 1.33 (m, 2H), 1.28 – 1.24 (m, 3H), 1.16 (s, 3H), 1.09 (s, 3H), 1.05 (s, 3H).

**$^{13}$C NMR (101 MHz, CDCl$_3$)** δ 176.9, 171.1, 145.2, 142.7, 138.3, 138.1, 138.0, 135.3, 130.2, 129.7, 128.6, 128.5, 128.5, 128.0, 128.0, 127.9, 127.7, 127.0, 121.2, 117.3, 104.9, 100.7, 99.6, 82.3, 77.7, 75.6, 75.5, 75.2, 73.8, 68.7, 44.4, 42.1, 41.2, 38.9, 38.7, 36.4, 35.4, 35.2, 31.9, 31.5, 29.8, 25.9, 25.3, 24.5, 23.9, 22.4, 22.1, 21.2, 20.0, 11.1.

**$[\alpha]_D^{25}$** = -32.5 (c = 0.24, CHCl$_3$).

**HRMS** (ESI-TOF): calculated for $C_{58}H_{68}O_{10}Na^+$ [M+Na$^+$]: 947.4705, found 947.4677.

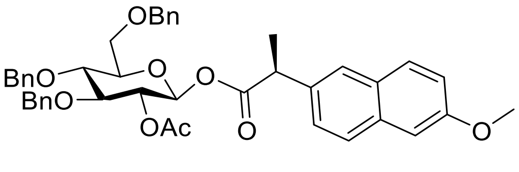

**3aj**

**(2*S*,3*R*,4*S*,5*R*,6*R*)-3-Acetoxy-4,5-bis(benzyloxy)-6-((benzyloxy)methyl)tetrahydro-2*H*-pyran-2-yl (*S*)-2-(6-methoxynaphthalen-2-yl)propanoate**

The title compound was synthesized according to **general procedure D** using glycosyl donor **1a** (45.4 mg, 0.075 mmol) and acceptor **2aj** (11.5 mg, 0.05 mmol) as reactants and **D** (3.3 mg, 20 mol%) as the catalyst. The reaction mixture was stirred at 40 °C for 12 h and then purified by column chromatography on silica gel with petroleum ether/ethyl acetate (5:1) as eluent to afford **3aj** as a white solid (34.9 mg, 99% yield).

**$^1$H NMR (400 MHz, $CDCl_3$)** δ 7.71 – 7.66 (m, 3H), 7.41 (d, *J* = 8.4 Hz, 1H), 7.33 – 7.26 (m, 13H), 7.18 – 7.12 (m, 3H), 7.08 (d, *J* = 2.5 Hz, 1H), 5.63 (d, *J* = 8.1 Hz, 1H), 5.15 (t, *J* = 8.6 Hz, 1H), 4.80 – 4.74 (m, 2H), 4.65 (d, *J* = 11.5 Hz, 1H), 4.54 – 4.51 (m, 2H), 4.41 (d, *J* = 12.1 Hz, 1H), 3.93 – 3.87 (m, 4H), 3.77 (t, *J* = 9.2 Hz, 1H), 3.71 – 3.66 (m, 3H), 3.58 (d, *J* = 9.6 Hz, 1H), 1.70 (s, 3H), 1.61 (d, *J* = 7.2 Hz, 3H).

**$^{13}$C NMR (101 MHz, $CDCl_3$)** δ 173.1, 169.4, 157.8, 138.2, 138.1, 138.0, 134.5, 133.9, 129.5, 129.0, 128.6, 128.5, 128.4, 128.1, 128.0, 127.9, 127.9, 127.7, 127.2, 126.5, 126.4, 119.1, 105.7, 92.5, 82.8, 76.1, 75.1, 75.1, 73.6, 72.0, 68.2, 55.4, 45.6, 20.7, 18.2.

**$[\alpha]_D^{25}$** = -2.7 (c = 0.25, $CHCl_3$).

**HRMS** (ESI-TOF): calculated for $C_{43}H_{44}O_9Na^+$ [M+Na$^+$]: 727.2878, found 727.2857.

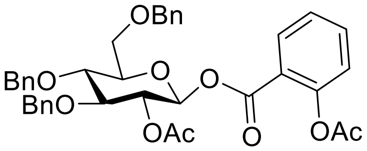


**3ak**

**(2*S*,3*R*,4*S*,5*R*,6*R*)-3-Aacetoxy-4,5-bis(benzyloxy)-6-((benzyloxy)methyl)tetrahydro-2*H*-pyran-2-yl 2-acetoxybenzoate**

The title compound was synthesized according to **general procedure D** using glycosyl donor **1a** (45.4 mg, 0.075 mmol) and acceptor **2ak** (9.0 mg, 0.05 mmol) as reactants and **D** (3.3 mg, 20 mol%) as the catalyst. The reaction mixture was stirred at 40 °C for 12 h and then purified by column chromatography on silica gel with petroleum ether/ethyl acetate (6:1) as eluent to afford **3ak** as a white solid (31.4 mg, 96% yield).

**$^1$H NMR (400 MHz, $CDCl_3$)** δ 8.06 (d, *J* = 7.7 Hz, 1H), 7.60 (t, *J* = 7.9 Hz, 1H), 7.34 – 7.26 (m, 13H), 7.21 – 7.16 (m, 2H), 7.13 (d, *J* = 8.1 Hz, 2H), 5.79 (d, *J* = 8.2 Hz, 1H), 5.28 (t, *J* = 8.7 Hz, 1H), 4.86 – 4.81 (m, 2H), 4.72 (d, *J* = 11.5 Hz, 1H), 4.65 – 4.59 (m, 2H), 4.52 (d, *J* = 12.1 Hz, 1H), 3.90 (t, *J* = 9.3 Hz, 1H), 3.82 – 3.77 (m, 3H), 3.69 (d, *J* = 9.6 Hz, 1H), 2.36 (s, 3H), 1.91 (s, 3H).

**$^{13}$C NMR (101 MHz, $CDCl_3$)** δ 169.7, 169.7, 162.3, 151.6, 138.1, 138.0, 137.9,

134.8, 132.4, 128.6, 128.6, 128.5, 128.1, 128.0, 128.0, 127.9, 127.8, 126.4, 124.1, 121.8, 92.7, 82.8, 76.1, 75.3, 75.2, 73.7, 72.2, 68.2, 21.1, 20.9.

**[α]D^25** = -31.1 (c = 0.25, $CHCl_3$).

**HRMS** (ESI-TOF): calculated for $C_{38}H_{38}O_{10}Na^+$ [M+Na+]: 677.2357, found 677.2336.

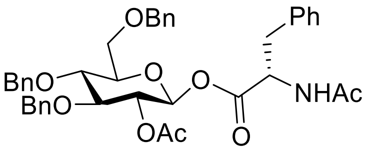


**3al**

**(2*S*,3*R*,4*S*,5*R*,6*R*)-3-Acetoxy-4,5-bis(benzyloxy)-6-((benzyloxy)methyl)tetrahydro-2*H*-pyran-2-yl acetyl-L-phenylalaninate**

The title compound was synthesized according to **general procedure D** using glycosyl donor **1a** (45.4 mg, 0.075 mmol) and acceptor **2al** (10.4 mg, 0.05 mmol) as reactants and **D** (3.3 mg, 20 mol%) as the catalyst. The reaction mixture was stirred at 40 °C for 12 h and then purified by column chromatography on silica gel with petroleum ether/ethyl acetate (5:1) as eluent to afford **3al** as a colorless syrup (28.3 mg, 83% yield).

**$^1$H NMR (400 MHz, CDCl$_3$)** δ 7.33 – 7.21 (m, 16H), 7.20 – 7.15 (m, 4H), 5.83 (d, *J* = 7.7 Hz, 1H), 5.63 (d, *J* = 8.2 Hz, 1H), 5.15 (t, *J* = 8.7 Hz, 1H), 4.85 – 4.79 (m, 3H), 4.70 – 4.62 (m, 2H), 4.58 – 4.53 (m, 2H), 3.85 (t, *J* = 9.4 Hz, 1H), 3.78 – 3.71 (m, 3H), 3.64 (d, *J* = 9.4 Hz, 1H), 3.17 – 3.16 (m, 2H), 1.96 (s, 3H), 1.90 (s, 3H).

**$^{13}$C NMR (101 MHz, CDCl$_3$)** δ 170.0, 169.9, 138.1, 137.9, 137.9, 135.7, 129.7, 128.7, 128.6, 128.6, 128.1, 128.0, 128.0, 127.9, 127.9, 127.2, 93.1, 82.7, 77.3, 76.0, 75.3, 75.2, 73.8, 72.0, 68.3, 53.2, 36.9, 23.1, 20.9.

**[α]D^25** = -311.1 (c = 0.20, $CHCl_3$).

**HRMS** (ESI-TOF): calculated for $C_{40}H_{43}NO_9Na^+$ [M+Na+]: 704.2830, found 704.2808.

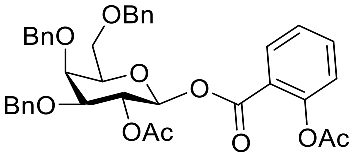


**3am**

The title compound was synthesized according to **general procedure D** using glycosyl donor **1c** (45.4 mg, 0.075 mmol) and acceptor **2ak** (9.0 mg, 0.05 mmol) as reactants and **D** (3.3 mg, 20 mol%) as the catalyst. The reaction mixture was stirred at

40 °C for 12 h and then purified by column chromatography on silica gel with petroleum ether/ethyl acetate (6:1) as eluent to afford **3am** as a white solid (31.4 mg, 96% yield).

**$^{1}$H NMR (400 MHz, CDCl$_3$)** δ 8.06 (dd, *J* = 8.0, 1.7 Hz, 1H), 7.60 – 7.53 (m, 1H), 7.38 – 7.26 (m, 16H), 7.08 (d, *J* = 8.1 Hz, 1H), 5.74 (d, *J* = 8.2 Hz, 1H), 5.62 (dd, *J* = 10.1, 8.2 Hz, 1H), 4.96 (d, *J* = 11.4 Hz, 1H), 4.70 (d, *J* = 12.2 Hz, 1H), 4.62 (d, *J* = 11.4 Hz, 1H), 4.55 (d, *J* = 12.2 Hz, 1H), 4.48 – 4.40 (m, 2H), 4.05 (d, *J* = 2.8 Hz, 1H), 3.79 (t, *J* = 6.6 Hz, 1H), 3.69 – 3.60 (m, 3H), 2.32 (s, 3H), 1.97 (s, 3H).

**$^{13}$C NMR (101 MHz, CDCl$_3$)** δ 169.9, 169.8, 162.4, 151.5, 138.4, 137.8, 137.8, 134.8, 132.6, 128.6, 128.6, 128.4, 128.4, 128.2, 128.0, 127.8, 127.7, 126.4, 124.0, 121.7, 93.1, 80.1, 74.8, 74.6, 73.7, 72.5, 72.3, 70.3, 68.0, 21.1, 21.0.

**$[\alpha]_D^{25}$** = -33.6 (c = 0.36, CHCl$_3$).

**HRMS** (ESI-TOF): calculated for $C_{38}H_{38}O_{10}Na^+$ [M+Na$^+$]: 677.2357, found 677.2341.

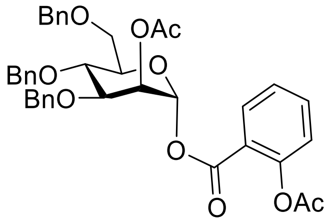


**3an**

**(2*R*,3*S*,4*S*,5*R*,6*R*)-3-Acetoxy-4,5-bis(benzyloxy)-6-((benzyloxy)methyl)tetrahydro-2*H*-pyran-2-yl 2-acetoxybenzoate**

The title compound was synthesized according to **general procedure D** using glycosyl donor **1b** (45.4 mg, 0.075 mmol) and acceptor **2ak** (9.0 mg, 0.05 mmol) as reactants and **D** (3.3 mg, 20 mol%) as the catalyst. The reaction mixture was stirred at 40 °C for 12 h and then purified by column chromatography on silica gel with petroleum ether/ethyl acetate (6:1) as eluent to afford **3an** as a white solid (17.3 mg, 53% yield).

**$^{1}$H NMR (400 MHz, CDCl$_3$)** δ 7.76 (d, *J* = 7.8 Hz, 1H), 7.55 (t, *J* = 7.9 Hz, 1H), 7.32 – 7.20 (m, 14H), 7.15 – 7.07 (m, 3H), 6.26 (s, 1H), 5.44 (s, 1H), 4.84 (d, *J* = 10.7 Hz, 1H), 4.74 – 4.64 (m, 2H), 4.56 – 4.45 (m, 3H), 4.01 (d, *J* = 8.3 Hz, 2H), 3.88 – 3.87 (m, 1H), 3.82 – 3.78 (m, 1H), 3.66 (d, *J* = 11.0 Hz, 1H), 2.29 (s, 3H), 2.15 (s, 3H).

**$^{13}$C NMR (101 MHz, CDCl$_3$)** δ 170.2, 169.7, 161.6, 151.4, 138.3, 138.2, 137.7, 134.6, 131.4, 128.6, 128.5, 128.4, 128.1, 128.0, 128.0, 127.9, 127.7, 126.1, 124.2, 122.4, 92.1, 77.6, 75.6, 74.4, 73.8, 73.7, 72.1, 68.6, 67.7, 21.1, 21.1.

**$[\alpha]_D^{25}$** = +1.0 (c = 0.28, CHCl$_3$).

**HRMS** (ESI-TOF): calculated for $C_{38}H_{38}O_{10}Na^+$ [M+Na$^+$]: 677.2357, found 677.2339.

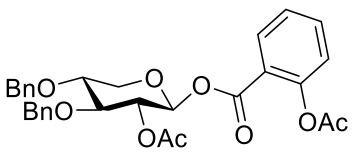

**3ao**

**(2*S*,3*R*,4*S*,5*R*)-3-Acetoxy-4,5-bis(benzyloxy)tetrahydro-2*H*-pyran-2-yl 2-acetoxybenzoate**

The title compound was synthesized according to **general procedure D** using glycosyl donor **1d** (36.4 mg, 0.075 mmol) and acceptor **2ak** (9.0 mg, 0.05 mmol) as reactants and **D** (3.3 mg, 20 mol%) as the catalyst. The reaction mixture was stirred at 40 °C for 12 h and then purified by column chromatography on silica gel with petroleum ether/ethyl acetate (6:1) as eluent to afford **3ao** as a white solid (26.4 mg, 99% yield).

**$^1$H NMR (400 MHz, $CDCl_3$)** δ 7.99 (d, $J$ = 7.8 Hz, 1H), 7.57 (t, $J$ = 7.8 Hz, 1H), 7.37 – 7.23 (m, 11H), 7.10 (d, $J$ = 8.1 Hz, 1H), 5.81 (d, $J$ = 6.8 Hz, 1H), 5.16 (t, $J$ = 7.2 Hz, 1H), 4.83 (d, $J$ = 11.6 Hz, 1H), 4.85 – 4.63 (m, 3H), 4.09 – 4.05 (m, 1H), 3.74 – 3.72 (m, 2H), 3.55 – 3.50 (m, 1H), 2.36 (s, 3H), 1.95 (s, 3H).

**$^{13}$C NMR (101 MHz, $CDCl_3$)** δ 169.8, 169.8, 162.3, 151.6, 138.1, 137.9, 134.8, 132.3, 128.7, 128.6, 128.2, 128.0, 128.0, 127.9, 126.4, 124.1, 121.8, 93.0, 79.6, 76.7, 74.6, 73.2, 70.7, 64.0, 21.1, 20.9.

**$[\alpha]_D^{25}$** = -7.3 (c = 0.15, $CHCl_3$).

**HRMS** (ESI-TOF): calculated for $C_{30}H_{30}O_9Na^+$ [M+Na$^+$]: 557.1782, found 557.1770.

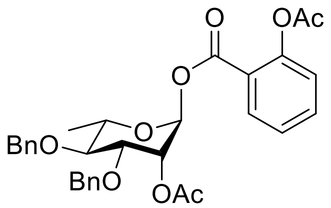

**3ap**

**(2*S*,3*R*,4*R*,5*S*,6*S*)-3-Acetoxy-4,5-bis(benzyloxy)-6-methyltetrahydro-2*H*-pyran-2-yl 2-acetoxybenzoate**

The title compound was synthesized according to **general procedure D** using glycosyl donor **1f** (25.0 mg, 0.075 mmol) and acceptor **2ak** (9.0 mg, 0.05 mmol) as reactants and **D** (3.3 mg, 20 mol%) as the catalyst. The reaction mixture was stirred at 40 °C for 12 h and then purified by column chromatography on silica gel with petroleum ether/ethyl acetate (6:1) as eluent to afford **3ap** as a white solid (18.9 mg, 69% yield).

$^{1}$**H NMR (400 MHz, CDCl$_3$)** δ 7.91 (d, *J* = 8.9 Hz, 2H), 7.38 – 7.30 (m, 10H), 6.92 (d, *J* = 8.9 Hz, 2H), 6.23 (d, *J* = 2.0 Hz, 1H), 5.49 – 5.48 (m, 1H), 4.96 (d, *J* = 10.6 Hz, 1H), 4.78 (d, *J* = 11.2 Hz, 1H), 4.67 (d, *J* = 10.7 Hz, 1H), 4.60 (d, *J* = 11.2 Hz, 1H), 4.06 – 4.03 (m, 1H), 3.93 – 3.88 (m, 1H), 3.88 (s, 3H), 3.56 (t, *J* = 9.5 Hz, 1H), 2.21 (s, 3H), 1.35 (d, *J* = 6.2 Hz, 3H).

$^{13}$**C NMR (101 MHz, CDCl$_3$)** δ 170.2, 164.1, 163.9, 138.3, 137.7, 132.2, 128.6, 128.5, 128.3, 128.1, 128.0, 121.5, 114.0, 91.5, 79.7, 77.6, 75.9, 72.0, 70.4, 68.2, 55.6, 21.1, 18.2.

**[α]$_D$$^{25}$** = -376.8 (c = 0.25, CHCl$_3$).

**HRMS** (ESI-TOF): calculated for $C_{31}H_{32}O_9Na^+$ [M+Na$^+$]: 571.1939, found 571.1908.

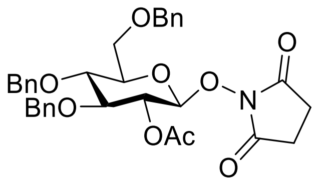


**3aq**

**(2*S*,3*R*,4*S*,5*R*,6*R*)-4,5-Bis(benzyloxy)-6-((benzyloxy)methyl)-2-((2,5-dioxopyrrolidin-1-yl)oxy)tetrahydro-2*H*-pyran-3-yl acetate**

The title compound was synthesized according to **general procedure D** using glycosyl donor **1a** (45.4 mg, 0.075 mmol) and acceptor **2aq** (5.8 mg, 0.05 mmol) as reactants and **F** (4.0 mg, 20 mol%) as the catalyst. The reaction mixture was stirred at 40 °C for 12 h and then purified by column chromatography on silica gel with petroleum ether/ethyl acetate (6:1) as eluent to afford **3aq** as a colorless syrup (20.6 mg, 70% yield).

$^{1}$**H NMR (400 MHz, CDCl$_3$)** δ 7.34 – 7.26 (m, 13H), 7.17 – 7.14 (m, 2H), 5.45 (d, *J* = 4.0 Hz, 1H), 4.98 – 4.95 (m, 1H), 4.85 – 4.77 (m, 3H), 4.64 – 4.61 (m, 2H), 4.54 (d, *J* = 10.8 Hz, 1H), 4.44 (d, *J* = 12.0 Hz, 1H), 4.09 (t, *J* = 9.7 Hz, 1H), 3.89 – 3.82 (m, 2H), 3.65 – 3.62 (m, 1H), 2.68 (s, 4H), 2.14 (s, 3H).

$^{13}$**C NMR (101 MHz, CDCl$_3$)** δ 170.9, 138.9, 138.5, 138.3, 128.9, 128.8, 128.4, 128.3, 128.2, 128.2, 128.2, 128.1, 102.1, 79.8, 77.4, 75.9, 75.7, 73.9, 72.9, 72.8, 68.3, 25.9, 21.2.

**[α]$_D$$^{25}$** = +56.3 (c = 0.30, CHCl$_3$).

**HRMS** (ESI-TOF): calculated for $C_{33}H_{35}NO_9Na^+$ [M+Na$^+$]: 612.2204, found 612.2197.

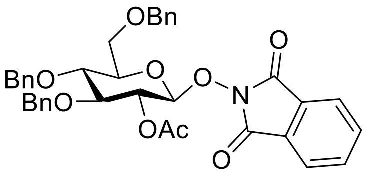

**3ar**

**(2*S*,3*R*,4*S*,5*R*,6*R*)-4,5-Bis(benzyloxy)-6-((benzyloxy)methyl)-2-((1,3-dioxoisoindolin-2-yl)oxy)tetrahydro-2*H*-pyran-3-yl acetate**

The title compound was synthesized according to **general procedure D** using glycosyl donor **1a** (45.4 mg, 0.075 mmol) and acceptor **2ar** (8.2 mg, 0.05 mmol) as reactants and **F** (4.0 mg, 20 mol%) as the catalyst. The reaction mixture was stirred at 40 °C for 12 h and then purified by column chromatography on silica gel with petroleum ether/ethyl acetate (7:1) as eluent to afford **3ar** as a white solid (24.2 mg, 76% yield).

**$^1$H NMR (400 MHz, CDCl$_3$)** δ 7.83 – 7.79 (m, 2H), 7.77 – 7.74 (m, 2H), 7.35 – 7.25 (m, 13H), 7.20 – 7.18 (m, 2H), 5.55 (d, *J* = 4.0 Hz, 1H), 5.01 (dd, *J* = 10.4, 4.0 Hz, 1H), 4.89 – 4.81 (m, 3H), 4.75 – 4.71 (m, 1H), 4.65 (d, *J* = 12.0 Hz, 1H), 4.58 (d, *J* = 10.8 Hz, 1H), 4.46 (d, *J* = 12.1 Hz, 1H), 4.15 (t, *J* = 9.7 Hz, 1H), 3.97 – 3.88 (m, 2H), 3.74 – 3.70 (m, 1H), 2.21 (s, 3H).

**$^{13}$C NMR (101 MHz, CDCl$_3$)** δ 170.7, 163.2, 138.6, 138.3, 138.1, 134.7, 129.0, 128.5, 128.5, 128.5, 128.0, 127.9, 127.8, 127.8, 127.8, 123.8, 102.7, 79.6, 77.4, 75.6, 75.4, 73.7, 72.7, 72.6, 68.1, 20.9.

**$[\alpha]_D^{25}$** = -22.4 (c = 0.25, CHCl$_3$).

**HRMS** (ESI-TOF): calculated for $C_{37}H_{35}NO_9Na^+$ [M+Na$^+$]: 660.2204, found 660.2194.

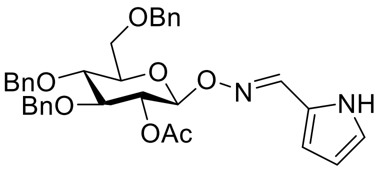

**3as**

**(2*S*,3*R*,4*S*,5*R*,6*R*)-2-((((E)-(1H-pyrrol-2-yl)methylene)amino)oxy)-4,5-bis(benzyloxy)-6-((benzyloxy)methyl)tetrahydro-2*H*-pyran-3-yl acetate**

The title compound was synthesized according to **general procedure D** using glycosyl donor **1a** (45.4 mg, 0.075 mmol) and acceptor **2as** (5.5 mg, 0.05 mmol) as reactants and **F** (8.0 mg, 40 mol%) as the catalyst. The reaction mixture was stirred at 40 °C for 12 h and then purified by column chromatography on silica gel with petroleum ether/ethyl acetate (7:1) as eluent to afford **3as** as a white solid (18.7 mg, 64% yield).

**$^1$H NMR (400 MHz, CDCl$_3$)** δ 10.20 (s, 1H), 7.35 – 7.26 (m, 14H), 7.18 – 7.16 (m, 2H), 6.97 (d, *J* = 2.0 Hz, 1H), 6.49 (s, 1H), 6.22 (d, *J* = 3.0 Hz, 1H), 5.20 (t, *J* = 8.5 Hz, 1H), 5.11 (d, *J* = 8.2 Hz, 1H), 4.88 – 4.76 (m, 3H), 4.66 (d, *J* = 12.1 Hz, 1H), 4.59 – 4.51 (m, 2H), 3.92 – 3.82 (m, 4H), 3.64 (d, *J* = 9.5 Hz, 1H), 1.96 (s, 3H).

**$^{13}$C NMR (101 MHz, CDCl$_3$)** δ 171.6, 140.4, 138.4, 138.1, 138.1, 128.6, 128.6, 128.5, 128.1, 128.0, 128.0, 127.8, 123.5, 123.4, 116.5, 109.2, 102.4, 82.9, 77.7, 75.4, 75.2, 73.7, 72.9, 68.2, 21.2.

**$[\alpha]_D^{25}$** = -31.9 (c = 0.21, CHCl$_3$).

**HRMS** (ESI-TOF): calculated for $C_{34}H_{36}N_2O_7Na^+$ [M+Na$^+$]: 607.2415, found 607.2402.

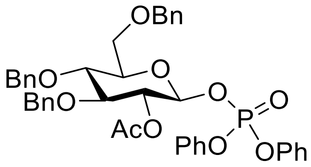


**3at**

**(2*S*,3*R*,4*S*,5*R*,6*R*)-4,5-Bis(benzyloxy)-6-((benzyloxy)methyl)-2-((diphenoxyphosphoryl)oxy)tetrahydro-2*H*-pyran-3-yl acetate**

The title compound was synthesized according to **general procedure D** using glycosyl donor **1a** (45.4 mg, 0.075 mmol) and acceptor **2at** (12.5 mg, 0.05 mmol) as reactants and **F** (4.0 mg, 20 mol%) as the catalyst. The reaction mixture was stirred at 40 °C for 12 h and then purified by column chromatography on silica gel with petroleum ether/ethyl acetate (5:1) as eluent to afford **3at** as a white solid (35.5 mg, 98% yield).

The title compound **3at** (24.6 mg, 68% yield) also can obtain according to **general procedure D** using glycosyl donor **1a** (45.4 mg, 0.075 mmol) and acceptor **2at** (12.5 mg, 0.05 mmol) as reactants and stirring at 40 °C for 36 h in the unpresence of catalyst.

**$^1$H NMR (500 MHz, CDCl$_3$)** δ 7.33 – 7.24 (m, 17H), 7.21 – 7.12 (m, 8H), 6.05 – 6.03 (m, 1H), 4.96 – 4.92 (m, 1H), 4.79 – 4.76 (m, 2H), 4.67 (d, *J* = 11.4 Hz, 1H), 4.57 – 4.50 (m, 2H), 4.44 (d, *J* = 12.1 Hz, 1H), 3.97 – 3.93 (m, 1H), 3.88 – 3.84 (m, 1H), 3.82 – 3.78 (m, 1H), 3.67 – 3.65 (m, 1H), 3.42 – 3.40 (m, 1H), 1.80 (s, 3H).

**$^{13}$C NMR (126 MHz, CDCl$_3$)** δ 170.2, 150.5, 138.4, 138.0, 137.9, 130.0, 129.9, 128.6, 128.6, 128.1, 128.0, 128.0, 127.9, 127.8, 125.6, 120.4, 120.4, 120.2, 120.2, 96.3, 96.3, 79.4, 77.4, 77.4, 77.2, 76.9, 75.6, 75.4, 73.6, 73.1, 72.5, 72.4, 67.6, 20.7.

**$^{31}$P NMR (162 MHz, CDCl$_3$)** δ -10.46.

**$[\alpha]_D^{25}$** = +23.0 (c = 0.57, CHCl$_3$).

**HRMS** (ESI-TOF): calculated for $C_{41}H_{41}O_{10}PNa^+$ [M+Na$^+$]: 747.2330, found 747.2322.

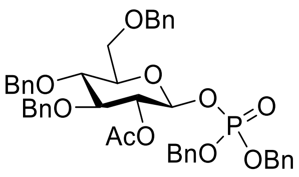

**3au**

**(2*S*,3*R*,4*S*,5*R*,6*R*)-4,5-Bis(benzyloxy)-6-((benzyloxy)methyl)-2-((bis(benzyloxy)phosphoryl)oxy)tetrahydro-2*H*-pyran-3-yl acetate**

The title compound was synthesized according to **general procedure D** using glycosyl donor **1a** (45.4 mg, 0.075 mmol) and acceptor **2au** (13.9 mg, 0.05 mmol) as reactants and **F** (4.0 mg, 20 mol%) as the catalyst. The reaction mixture was stirred at 40 °C for 12 h and then purified by column chromatography on silica gel with petroleum ether/ethyl acetate (5:1) as eluent to afford **3au** as a colorless syrup (32.0 mg, 85% yield).

**$^1$H NMR (400 MHz, $CDCl_3$)** δ 7.26 – 7.18 (m, 23H), 7.10 – 7.08 (m, 2H), 5.83 – 5.81 (m, 1H), 4.98 – 4.92 (m, 4H), 4.88 – 4.84 (m, 1H), 4.75 – 4.71 (m, 2H), 4.63 (d, *J* = 11.5 Hz, 1H), 4.50 – 4.44 (m, 2H), 4.36 (d, *J* = 12.1 Hz, 1H), 3.92 – 3.87 (m, 2H), 3.71 (t, *J* = 9.5 Hz, 1H), 3.62 – 3.58 (m, 1H), 3.44 (d, *J* = 10.7 Hz, 1H), 1.77 (s, 3H).

**$^{13}$C NMR (101 MHz, $CDCl_3$)** δ 170.1, 138.4, 138.1, 138.0, 135.8, 135.7, 135.7, 128.7, 128.7, 128.5, 128.0, 128.0, 128.0, 127.9, 127.8, 127.7, 95.1, 95.0, 79.5, 75.5, 75.3, 73.6, 72.9, 72.6, 72.5, 69.6, 69.5, 68.0, 20.7.

**$^{31}$P NMR (162 MHz, $CDCl_3$)** δ -2.57.

**$[\alpha]_D^{25}$** = +51.5 (c = 0.66, $CHCl_3$).

**HRMS** (ESI-TOF): calculated for $C_{43}H_{45}O_{10}PNa^+$ [M+Na$^+$]: 775.2643, found 775.2634.

### 5.3 Procedure for gram-scale synthesis

In a glove box filled with nitrogen, to an oven-dried 25 mL tube equipped with a stirring bar were added **1a** (0.9078 g, 1.5 mmol, 1.5 equiv.), acceptor **2c** (124.2 mg, 1.0 mmol, 1.0 equiv.), **D** (66.0 mg, 20 mol%), and anhydrous $CH_2Cl_2$ (7 mL, 0.14 M). The reaction mixture was stirred at 40 °C for 12 h and then purified by column chromatography on silica gel with petroleum ether/ethyl acetate (5:1) as eluent to afford **3c** as a white solid (580.2 mg, 97% yield).

## 6. Comparison of glycosyl donor reactivities

Procedure: In a glove box filled with nitrogen, to an oven-dried 10 mL tube equipped with a stirring bar were added glycosyl donor, acceptor, promoter and solvent.

The suspension was stirred at room temperature or 40 °C. Upon completion, the yield and α/β ratio were determined by crude $^{1}$H NMR spectrum using 1,3,5-trimethoxybenzene as an internal standard.

**Supplementary Table S3. Exploration of glycosyl donor reactivity under varied conditions.**

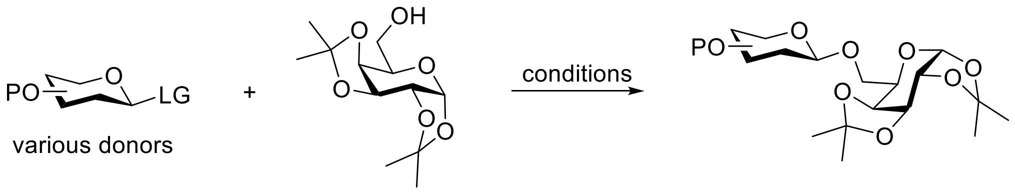


| Donor | Conditions | Conversion of donor (%) | Yield (%) |
|---|---|---|---|
| 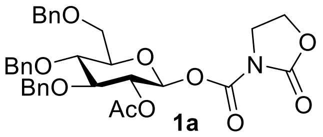 **1a** | NIS/TMSOTf, 3Å MS, 0 °C to RT, 12 h | trace | trace |
| | $Ph_3PAuOTf$, 4Å MS, RT, 10 h | 0 | 0 |
| 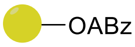 (**5**) | Cat. **F**, $CH_2Cl_2$, 40°C, 10 h | 0 | 0 |
| 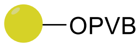 (**6**) | Cat. **F**, $CH_2Cl_2$, 40°C, 10 h | 0 | 0 |
| 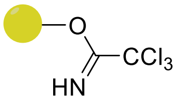 (**7**) | Cat. **F**, $CH_2Cl_2$, 40°C, 10 h | 100 | 72 (β) |
| 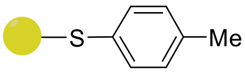 (**8**) | Cat. **F**, $CH_2Cl_2$, 40°C, 10 h | 0 | 0 |

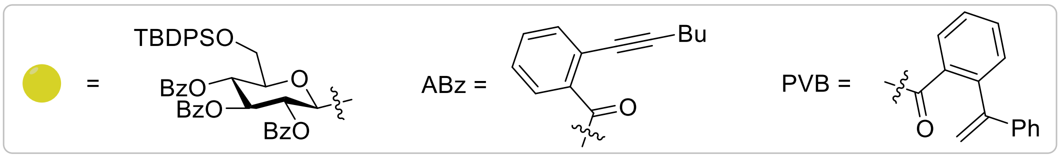


**Method A**: A solution of glycosyl donor **1a** (36.3 mg, 0.06 mmol, 1.2 equiv.) and acceptor **2l** (13.0 mg, 0.05 mmol, 1.0 equiv.) in anhydrous $CH_2Cl_2$ (1.5 mL, 0.033 M) was stirred at room temperature for 30 min under nitrogen atmosphere in the presence of activated 3 Å MS (150 mg, 3.0 g/mmol). The reaction vessel was cooled to 0 °C, whereupon *N*-Iodosuccinimide (NIS, 16.9 mg, 0.075 mmol, 1.5 equiv.) and trimethylsilyl trifluoromethanesulfonate (TMSOTf, 2.6 μL, 0.015 mmol, 0.3 equiv.) were added sequentially. The resulting mixture was allowed to warm gradually to room temperature and stirred for 12 h. As detailed in **Supplementary Table S3**, glycosyl donor **1a** exhibited constrained reactivity under Xiao's glycosylation conditions.

**Method B**: A solution of glycosyl donor **1a** (36.3 mg, 0.06 mmol, 1.2 equiv.) and acceptor **2l** (13.0 mg, 0.05 mmol, 1.0 equiv.) in anhydrous $CH_2Cl_2$ (1.5 mL, 0.033 M) was stirred at room temperature for 30 min under nitrogen atmosphere in the presence of activated 4 Å MS (150.0 mg, 3.0 g/mmol). Then to which $PPh_3AuOTf$ (6.1 mg, 20

mol%) was added to the mixture slowly. The resulting mixture was stirred at room temperature for 10 h. As detailed in **Supplementary Table S3**, glycosyl donor **1a** was completely unreactive under Yu's glycosylation conditions.

**Method C**: A solution of glycosyl donor **5**[12], **6**[13], **7**[14], **8**[15] (0.075 mmol, 1.5 equiv.), acceptor **2l** (0.05 mmol, 1.0 equiv.) and **F** (4.0 mg, 20 mol%) in anhydrous $CH_2Cl_2$ (1 mL, 0.05 M) was stirred at 40 °C for 12 h under nitrogen atmosphere. As detailed in **Supplementary Table S3**, only glycosyl donor **7** demonstrated reactivity (72%, β), while the other donors **5**, **6** and **8** remained completely inactive under Our's glycosylation conditions.

## 7. Synthesis of oligosaccharides 10, 14, 15, 19, 20

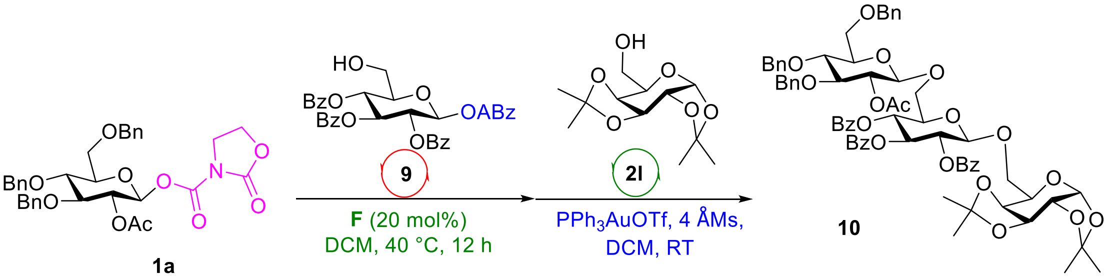


**Supplementary Figure S13. Orthogonal strategy for synthesis of trisaccharide 10.**

A suspension of glucosyl donor **1a** (45.4 mg, 0.075 mmol), glucosyl acceptor **9**[12] (34.9 mg, 0.05 mmol), and **F** (4.0 mg, 0.01 mmol) in anhydrous $CH_2Cl_2$ (1.3 mL) was stirred at 40 °C for 12 h under nitrogen atmosphere. Activated 4Å MS (150.0 mg) and glycosyl acceptor **2l** (11.7 mg, 0.045 mmol) were added, and the mixture was stirred at room temperature for 15 min. Then to which $PPh_3AuOTf$ (10.9 mg, 0.018 mmol, 40 mol%) was added. The resulting mixture was stirred at room temperature for another 3 h until TLC-analysis indicated the reaction to be complete. The filtrates were concentrated under vacuum to give a residue, which was purified by column chromatography on silica gel with petroleum ether/ethyl acetate (4:1) as eluent to afford **10** (35.4 mg, 65% yield) as a syrup.

**$^1$H NMR (400 MHz, $CDCl_3$)** δ 8.09 (d, $J$ = 7.6 Hz, 2H), 7.93 (d, $J$ = 7.8 Hz, 2H), 7.75 – 7.72 (m, 2H), 7.58 (q, $J$ = 8.0 Hz, 2H), 7.49 – 7.39 (m, 6H), 7.27 – 7.22 (m, 14H), 7.14 – 7.12 (m, 2H), 6.01 (d, $J$ = 5.3 Hz, 1H), 5.66 (d, $J$ = 3.0 Hz, 1H), 5.46 (d, $J$ = 5.0 Hz, 1H), 5.35 – 5.27 (m, 2H), 5.01 – 4.97 (m, 1H), 4.79 – 4.71 (m, 3H), 4.63 (d, $J$ = 11.4 Hz, 1H), 4.55 – 4.45 (m, 4H), 4.37 (d, $J$ = 8.0 Hz, 1H), 4.25 – 4.23 (m, 1H), 4.16 – 4.14 (m, 1H), 4.05 – 4.02 (m, 1H), 3.98 – 3.94 (m, 1H), 3.88 –3.85 (m, 1H), 3.68 – 3.60 (m, 4H), 3.52 – 3.42 (m, 3H), 1.85 (s, 3H), 1.47 (s, 3H), 1.35 (s, 3H), 1.24 (s, 3H),

1.23 (s, 3H).

**$^{13}$C NMR (101 MHz, $CDCl_3$)** δ 169.7, 165.3, 164.9, 138.3, 138.2, 138.0, 135.0, 133.7, 133.6, 130.3, 130.0, 128.6, 128.5, 128.5, 128.4, 128.2, 128.0, 127.9, 126.7, 121.1, 109.3, 108.6, 101.7, 97.7, 96.4, 83.0, 78.1, 75.3, 75.2, 73.6, 73.0, 72.4, 71.0, 70.7, 70.6, 69.6, 68.9, 68.4, 68.2, 66.7, 63.0, 36.1, 32.1, 29.5, 27.4, 26.2, 26.1, 25.7, 25.0, 24.5, 22.8, 20.8, 14.3.

**$[\alpha]_D^{25}$** = -226.5 (c = 0.2, $CHCl_3$).

**HRMS** (ESI-TOF): calculated for $C_{68}H_{72}O_{20}Na^+$ [M+Na$^+$]: 1231.4509, found 1231.4505.

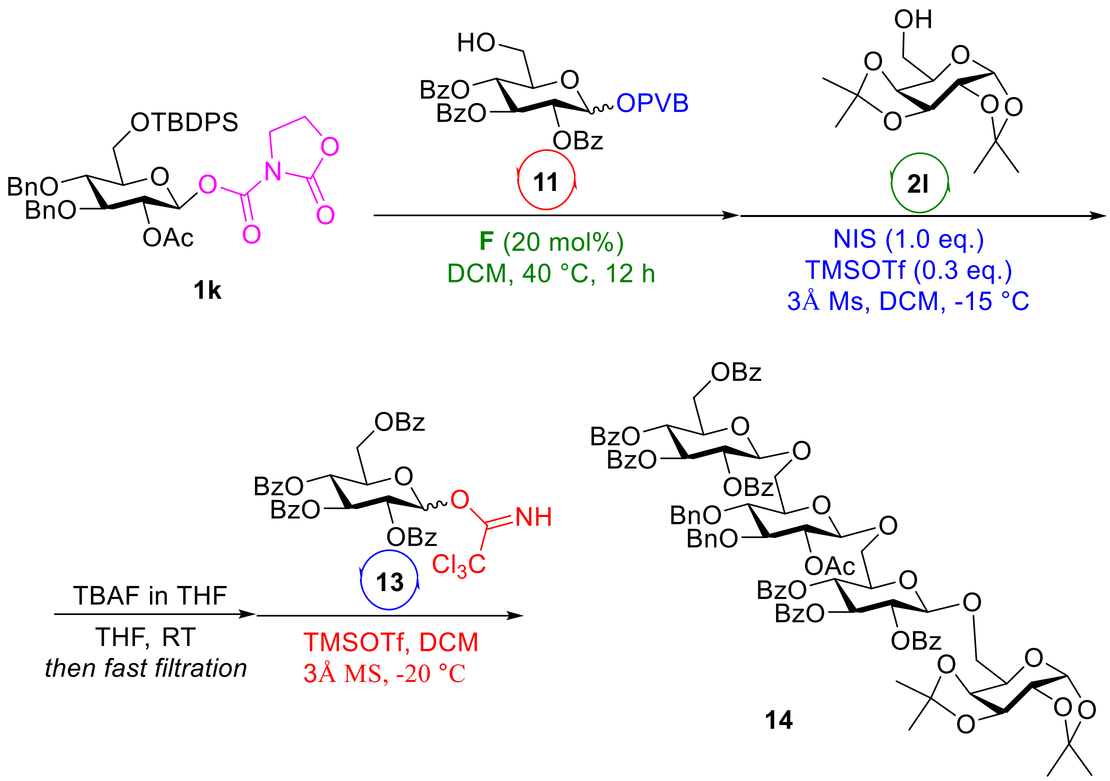


**Supplementary Figure S14. Orthogonal strategy for synthesis of 14.**

A suspension of glucosyl donor **1k** (56.5 mg, 0.075 mmol), glucosyl acceptor **11**[13] (34.9 mg, 0.05 mmol), and **F** (4.0 mg, 0.01 mmol) in anhydrous $CH_2Cl_2$ (1 mL) was stirred at 40 °C for 12 h. Then the activated 3Å MS (135.0 mg) was added and stirred at room temperature for 15 min. Then the reaction mixture was cooled to -15°C, to which glycosyl acceptor **2l** (11.7 mg, 0.045 mmol) and NIS (15.2 mg, 0.0675 mmol, 1.5 equiv.) and TMSOTf (2.4 μL, 0.0135 mmol, 0.3 equiv.) were added successively. The resulting mixture was stirred at -15 °C for another 3 h until TLC-analysis indicated the reaction to be complete, and concentrated in vacuo.

The crude product mentioned above dissolved in THF (1 mL), was added TBAF in THF (1 mol/L, 0.06 mmol, 1.5 equiv.). The resulting mixture was stirred at room

temperature until TLC-analysis indicated the reaction to be complete. Then, the mixture was eluted by fast filtration with EtOAc as eluent and concentrated in vacuo to afford the crude product.

A suspension of the crude product mentioned above, glucosyl trichloroacetimidate **13**[14] (25.9 mg, 0.035 mmol), and activated 3Å MS (100 mg) in anhydrous $CH_2Cl_2$ (0.8 mL) was stirred at room temperature for 15 min and was then cooled to -20°C. TMSOTf (60 μL, 10 μL in 1 mL $CH_2Cl_2$) was added to the mixture dropwise. After being stirred at -20°C for 0.5 h, the reaction mixture quenched with $Et_3N$, filtered to remove 3Å MS and concentrated in vacuo. The reaction mixture was purified by column chromatography on silica gel with petroleum ether/ethyl acetate (4:1) as eluent to afford **14** (25.2 mg, 42% yield) as a syrup.

**$^1$H NMR (400 MHz, CDCl$_3$)** δ 8.06 (d, *J* = 7.8 Hz, 2H), 7.96 – 7.77 (m, 9H), 7.74 (d, *J* = 7.0 Hz, 3H), 7.64 – 6.87 (m, 31H), 5.93 (d, *J* = 5.2 Hz, 1H), 5.81 (t, *J* = 9.6 Hz, 1H), 5.71 (s, 1H), 5.54 – 5.41 (m, 2H), 5.37 – 5.32 (m, 2H), 4.95 – 4.70 (m, 5H), 4.64 – 4.48 (m, 4H), 4.41 – 4.33 (m, 2H), 4.17 – 4.04 (m, 4H), 3.95 (d, *J* = 10.8 Hz, 2H), 3.84 (t, *J* = 6.2 Hz, 1H), 3.76 – 3.71 (m, 2H), 3.62 – 3.58 (m, 2H), 3.53 – 3.47 (m, 2H), 3.36 (d, *J* = 11.0 Hz, 1H), 2.03 (s, 3H), 1.37 (s, 3H), 1.26 (s, 3H), 1.19 (s, 3H), 1.18 (s, 3H).

**$^{13}$C NMR (101 MHz, CDCl$_3$)** δ 169.7, 166.2, 165.9, 165.5, 165.3, 165.2, 164.7, 138.3, 137.9, 135.2, 133.7, 133.6, 133.3, 133.1, 130.2, 130.1, 130.1, 130.0, 129.9, 129.8, 129.6, 129.3, 129.2, 129.1, 129.0, 128.7, 128.6, 128.5, 128.5, 128.4, 128.4, 128.3, 127.9, 127.9, 127.8, 126.6, 121.4, 109.3, 108.5, 101.4, 101.2, 97.7, 96.3, 83.2, 77.9, 77.4, 75.1, 75.0, 74.5, 74.2, 73.2, 73.2, 72.6, 71.9, 70.9, 70.6, 69.9, 69.5, 68.6, 68.5, 68.1, 67.7, 67.1, 64.2, 63.2, 29.9, 29.5, 27.4, 26.1, 25.9, 25.0, 24.3, 21.1.

**$[\alpha]_D^{25}$** = -5.8 (c = 0.19, $CHCl_3$).

**HRMS** (ESI-TOF): calculated for $C_{95}H_{92}O_{29}Na^+$ [M+Na$^+$]: 1720.5650, found 1720.5649.

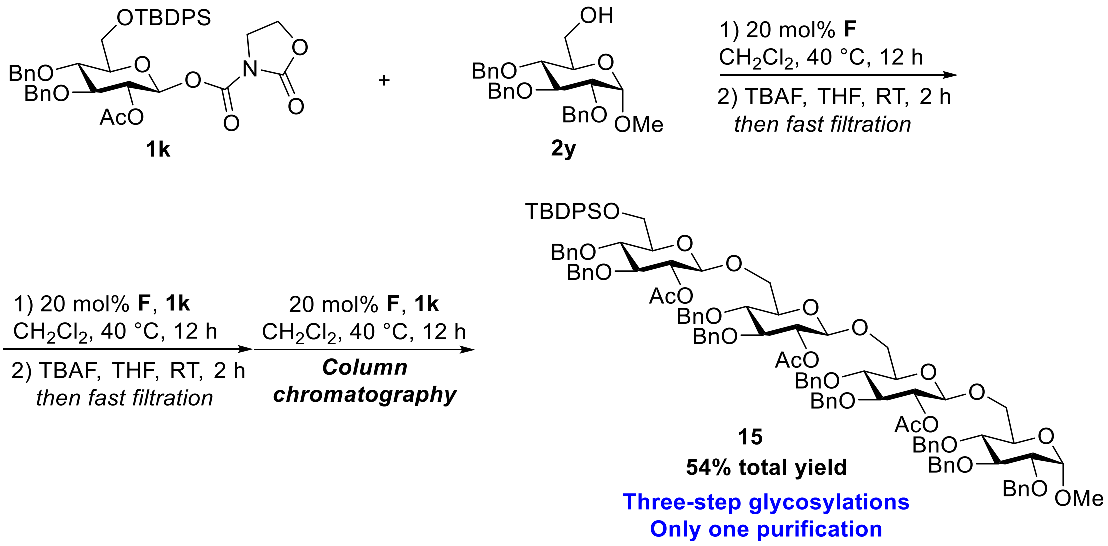


**Supplementary Figure S15. Iterative strategy for synthesis of tetrasaccharide 15.**

The glycosyl donor **1k** (113.0 mg, 0.15 mmol), acceptor **2y** (46.4 mg, 0.1 mmol) and **F** (8.0 mg, 0.02 mmol) were dissolved in anhydrous $CH_2Cl_2$ (2 mL, 0.05 M) under nitrogen atmosphere. After being stirred at 40 °C for 12 h, the solvent was removed under reduced pressure. Then the crude product was dissolved in THF (3.0 mL) under Air atmosphere and TBAF in THF (1 mol/L) (0.15 mL, 0.15 mmol) was added to the mixture and stirred at room temperature for 2 h. After the reaction was completed, the solvent was concentrated under reduced pressure. The resulting was eluted by fast filtration with EtOAc as eluent and concentrated in vacuo to afford the disaccharide crude product.

A mixture of glycosyl donor **1k** (90.4 mg, 0.12 mmol), the disaccharide crude product and **F** (6.3 mg, 0.016 mmol) in anhydrous $CH_2Cl_2$ (1.6 mL) was stirred at 40 °C for 12 h under nitrogen atmosphere. The solvent was removed under reduced pressure. Then the crude product was dissolved in THF (2.5 mL) under Air atmosphere and TBAF in THF (1 mol/L) (0.12 mL, 0.12 mmol) was added to the mixture and stirred at room temperature for 2 h. After the reaction was completed, the solvent was concentrated under reduced pressure. The resulting residue was eluted by fast filtration with EtOAc as eluent to afford the trisaccharide crude product.

The glycosyl donor **1k** (72.3 mg, 0.096 mmol), the trisaccharide crude product and **F** (5.1 mg, 0.0128 mmol) were dissolved in anhydrous $CH_2Cl_2$ (1.3 mL, 0.05 M) under nitrogen atmosphere and stirred at 40 °C for 12 h. The reaction mixture was purified by column chromatography on silica gel with petroleum ether/ethyl acetate (3:1) as eluent to afford **15** (96.4 mg, 54% total yield) as a white solid.

**$^1$H NMR (400 MHz, CDCl₃)** δ 7.74 – 7.71 (m, 2H), 7.66 (d, J = 7.2 Hz, 2H), 7.40 – 7.15 (m, 51H), 5.06 – 4.94 (m, 4H), 4.84 – 4.66 (m, 10H), 4.63 – 4.49 (m, 9H), 4.38

– 4.35 (m, 2H), 4.13 – 4.05 (m, 3H), 3.96 – 3.84 (m, 4H), 3.71 – 3.54 (m, 7H), 3.52 – 3.39 (m, 6H), 3.29 – 3.25 (m, 4H), 1.89 (s, 3H), 1.86 (s, 3H), 1.83 (s, 3H), 1.02 (s, 9H).

**$^{13}$C NMR (101 MHz, $CDCl_3$)** δ 169.4, 169.2, 138.9, 138.4, 138.3, 138.2, 138.1, 138.1, 137.9, 137.7, 136.0, 135.6, 133.7, 133.0, 129.8, 128.6, 128.5, 128.4, 128.3, 128.1, 128.0, 128.0, 128.0, 128.0, 127.9, 127.9, 127.8, 127.8, 127.6, 101.2, 101.0, 100.9, 98.3, 83.2, 83.1, 83.1, 82.0, 79.9, 78.2, 78.2, 77.7, 77.6, 77.5, 77.4, 77.2, 76.8, 76.1, 75.8, 75.7, 75.2, 75.2, 75.0, 74.9, 73.6, 73.1, 73.0, 69.9, 67.7, 67.6, 62.5, 55.4, 26.9, 21.2, 21.1, 21.0, 19.4.

**$[\alpha]_D^{25}$** = -814.8 (c = 0.27, $CHCl_3$).

**HRMS** (ESI-TOF): calculated for $C_{110}H_{122}O_{24}SiNa^+$ [M+Na$^+$]: 1878.8021, found 1878.7988.

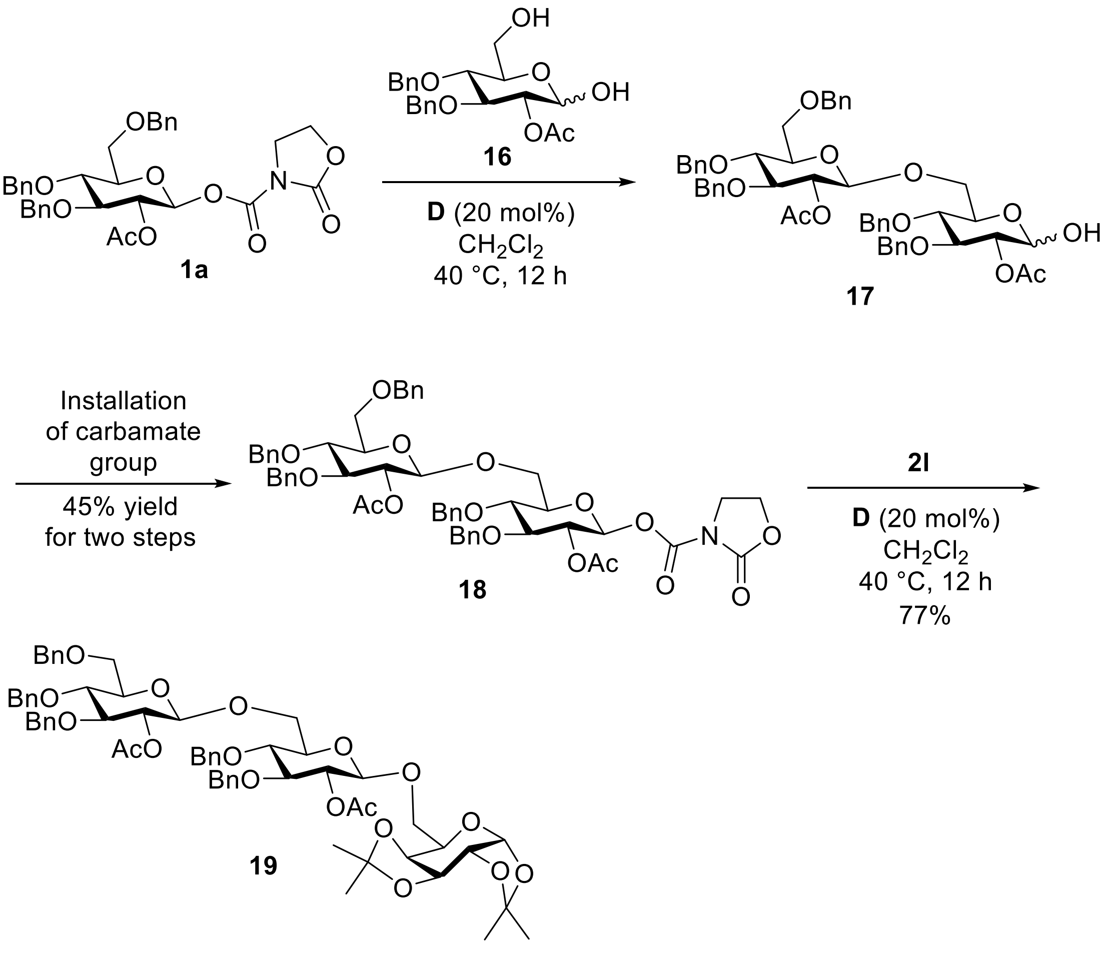


**Supplementary Figure S16. Latent-active strategy for synthesis of 19.**

A suspension of glucosyl donor **1a** (290.5 mg, 0.48 mmol), acceptor **16** (128.7 mg, 0.32 mmol) and **D** (25.4 mg, 0.064 mmol) in anhydrous $CH_2Cl_2$ (6.4 mL, 0.05 M) was stirred at 40 °C for 12 h under nitrogen atmosphere. Then the progress of the reaction was monitored by TLC. Upon completion, the reaction mixture was concentrated in vacuo to obtained crude product **17** as a syrup.

Compound **18** was prepared from crude product **17** according to **General procedure A**. The reaction mixture was purified by column chromatography on silica gel with petroleum ether/ethyl acetate (1:1) as eluent to afford **18** (142.5 mg, 45% yield for two steps) as a white solid.

**$^1$H NMR (400 MHz, CDCl$_3$)** δ 7.33 – 7.26 (m, 23H), 7.19 – 7.17 (m, 2H), 5.62 (d, $J$ = 7.8 Hz, 1H), 5.11 (t, $J$ = 7.8 Hz, 1H), 5.04 (t, $J$ = 8.5 Hz, 1H), 4.81 – 4.77 (m, 4H), 4.69 – 4.53 (m, 6H), 4.42 (d, $J$ = 8.0 Hz, 1H), 4.28 – 4.23 (m, 2H), 4.14 (d, $J$ = 11.1 Hz, 1H), 4.02 – 3.95 (m, 1H), 3.87 – 3.80 (m, 1H), 3.76 – 3.62 (m, 8H), 3.47 (t, $J$ = 7.3 Hz, 1H), 1.94 (s, 3H), 1.89 (s, 3H).

**$^{13}$C NMR (101 MHz, CDCl$_3$)** δ 169.7, 169.6, 151.9, 148.5, 138.2, 138.1, 138.0, 137.9, 128.7, 128.7, 128.6, 128.6, 128.5, 128.2, 128.2, 128.1, 128.1, 128.0, 128.0, 128.0, 127.9, 127.9, 127.9, 127.8, 100.9, 94.2, 83.1, 82.4, 78.1, 77.5, 77.4, 77.2, 77.1, 76.9, 75.5, 75.3, 75.2, 75.1, 75.0, 73.6, 73.1, 72.1, 68.7, 67.2, 61.9, 43.4, 21.1, 21.0.

**$[\alpha]_D^{25}$** = +6.7 (c = 0.21, CHCl$_3$).

**HRMS** (ESI-TOF): calculated for $C_{55}H_{59}NO_{16}Na^+$ [M+Na$^+$]: 1012.3726, found 1012.3703.

A suspension of glycosyl donor **18** (59.4 mg, 0.06 mmol), acceptor **2l** (16.1 mg, 0.04 mmol) and **D** (2.7 mg, 0.008 mmol) in anhydrous $CH_2Cl_2$ (0.8 mL, 0.05 M) was stirred at 40 °C for 12 h under nitrogen atmosphere. Then the progress of the reaction was monitored by TLC. Upon completion, the reaction mixture was concentrated in vacuo. The reaction mixture was purified by column chromatography on silica gel with petroleum ether/ethyl acetate (4:1) as eluent to afford **19** (34.4 mg, 77%) as a white solid.

**$^1$H NMR (400 MHz, CDCl$_3$)** δ 7.32 – 7.26 (m, 23H), 7.17 – 7.15 (m, 2H), 5.47 (d, $J$ = 4.9 Hz, 1H), 5.05 – 4.95 (m, 2H), 4.80 – 4.74 (m, 4H), 4.68 – 4.51 (m, 8H), 4.39 (d, $J$ = 8.0 Hz, 1H), 4.27 – 4.25 (m, 1H), 4.15 (d, $J$ = 8.1 Hz, 1H), 4.11 – 4.02 (m, 2H), 3.91 – 3.88 (m, 1H), 3.73 – 3.62 (m, 6H), 3.60 – 3.54 (m, 1H), 3.51 – 3.46 (m, 3H), 2.00 (s, 3H), 1.96 (s, 3H), 1.49 (s, 3H), 1.38 (s, 3H), 1.30 (s, 3H), 1.23 (s, 3H).

**$^{13}$C NMR (101 MHz, CDCl$_3$)** δ 169.8, 169.4, 138.2, 137.9, 137.8, 128.5, 128.4, 128.4, 128.4, 128.0, 128.0, 127.9, 127.9, 127.8, 127.8, 127.7, 127.6, 109.3, 108.7, 101.8, 101.1, 96.2, 83.2, 82.7, 78.1, 77.9, 77.2, 75.3, 75.1, 75.0, 75.0, 73.5, 73.0, 73.0, 71.2, 70.6, 70.6, 69.6, 68.7, 67.8, 67.7, 60.4, 50.9, 26.1, 26.0, 25.1, 24.2, 21.0, 20.9, 14.2.

**$[\alpha]_D^{25}$** = +2.3 (c = 0.57, CHCl$_3$).

**HRMS** (ESI-TOF): calculated for $C_{63}H_{74}O_{18}Na^+$ [M+Na$^+$]: 1141.4767, found 1141.4763.

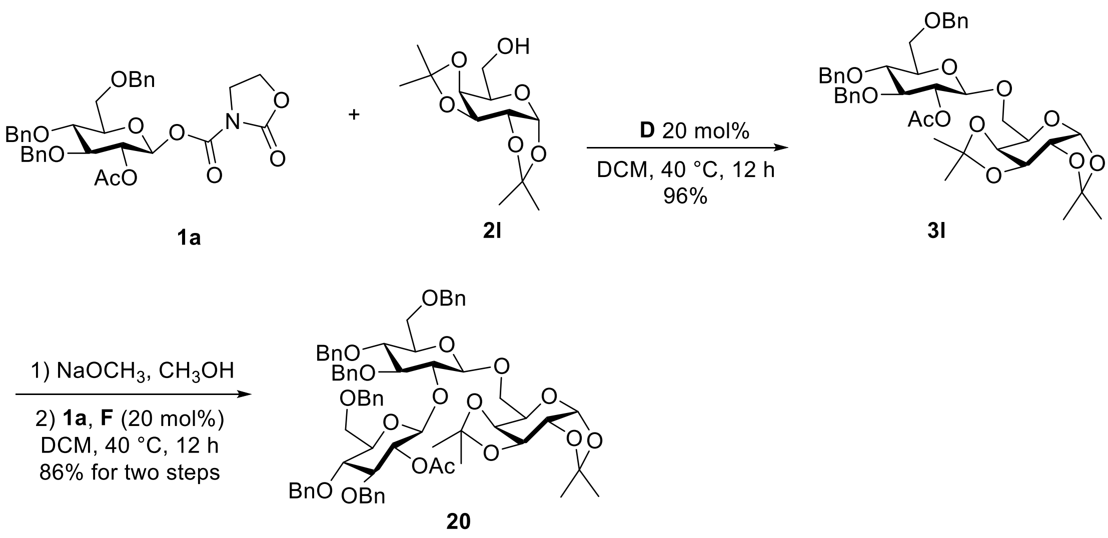


**Supplementary Figure S17. Sequential glycosylation for synthesis of 20.**

A suspension of glucosyl donor **1a** (45.4 mg, 0.075 mmol), acceptor **2l** (13.0 mg, 0.05 mmol) and **D** (3.3 mg, 0.01 mmol) in anhydrous $CH_2Cl_2$ (1.0 mL, 0.05 M) was stirred at 40 °C for 12 h under nitrogen atmosphere. Then the progress of the reaction was monitored by TLC. Upon completion, the reaction mixture was concentrated in vacuo. The residue was purified by flash column chromatography on silica gel with petroleum ether/ethyl acetate (5:1) as eluent to obtained **3l** as a colorless syrup (35.2 mg, 96% yield).

To a solution of disaccharide **3l** (35.2 mg, 0.048 mmol) in $CH_3OH$ (2 mL) was added $NaOCH_3$ (2.6 mg, 1.0 equiv.). The resulting mixture was stirred at room temperature until TLC-analysis indicated the reaction to be complete. Then, the mixture was diluted with $CH_2Cl_2$, and washed with water and brine. The organic layer was dried over $Na_2SO_4$, filtered, and concentrated in vacuo to afford the crude product of disaccharide receptor. Then the crude product of the disaccharide receptor was dissolved in anhydrous $CH_2Cl_2$ (1 mL) under a nitrogen atmosphere. Glucosyl donor **1a** (43.6 mg, 0.072 mmol) and catalyst **F** (3.9 mg, 0.01 mmol) were added, and the resulting mixture was stirred at 40 °C for 12 h. After completion, the reaction mixture was concentrated under reduced pressure, and the residue was purified by column chromatography on silica gel (petroleum ether/ethyl acetate = 4:1) to afford compound **20** (48.2 mg, 86% yield for two steps) as a syrup.

**$^1$H NMR (400 MHz, $CDCl_3$)** δ 7.35 – 7.23 (m, 26H), 7.18 – 7.15 (m, 2H), 7.10 – 7.08 (m, 2H), 5.48 (d, $J$ = 5.0 Hz, 1H), 5.01 (t, $J$ = 8.8 Hz, 1H), 4.91 (d, $J$ = 8.1 Hz, 1H), 4.85 (d, $J$ = 11.3 Hz, 1H), 4.77 – 4.66 (m, 5H), 4.62 – 4.48 (m, 8H), 4.27 – 4.26 (m, 1H), 4.20 – 4.17 (m, 1H), 4.03 – 3.979 (m, 2H), 3.82 – 3.58 (m, 11H), 3.43 – 3.40 (m, 1H), 1.73 (s, 3H), 1.53 (s, 3H), 1.38 (s, 3H), 1.31 (s, 3H), 1.27 (s, 3H).

**$^{13}$C NMR (101 MHz, $CDCl_3$)** δ 169.6, 138.9, 138.5, 138.4, 138.3, 138.2, 138.2,

128.5, 128.5, 128.4, 128.4, 128.3, 128.0, 127.9, 127.9, 127.8, 127.7, 127.7, 127.6, 127.6, 127.5, 109.2, 108.7, 102.1, 100.0, 96.4, 84.7, 83.3, 80.9, 78.1, 78.0, 77.4, 75.4, 75.0, 74.9, 74.9, 74.8, 74.6, 73.9, 73.6, 73.5, 71.5, 70.8, 70.5, 68.9, 68.7, 67.7, 26.3, 26.2, 25.1, 24.5, 21.0.

**[α]D$^{25}$** = +11.5 (c = 0.5, $CHCl_3$).

**HRMS** (ESI-TOF): calculated for $C_{68}H_{78}O_{17}Na^+$ [M+Na$^+$]: 1189.5131, found 1189.5117.

# 8. Mechanistic studies and proposed mechanism

## 8.1 Analysis and recovery of by-products in glycosylation reactions

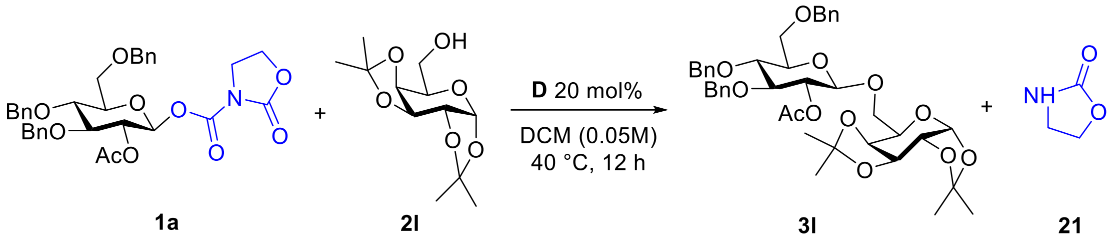


**Supplementary Figure S18. oxazolidinone recovery experiment.**

To an oven-dried vial was added glycosyl donor **1a** (90.8 mg, 0.15 mmol, 1.5 equiv.), acceptor **2l** (0.1 mmol, 1.0 equiv.), **D** (6.6 mg, 20 mol%) and dry $CH_2Cl_2$ (2 mL, 0.05 M) under nitrogen atmosphere. The suspension was stirred at 40 °C for 12 h. The reaction mixture was purified by column chromatography on silica gel with petroleum ether/ethyl acetate (2:1) as eluent to afford the glycosylated products **3l** as a colorless syrup (70.4 mg, 96% yield) and with ethyl acetate as eluent to afford by-product oxazolidinone **21** as a white solid (8.1 mg, 93% yield):

**$^1$H NMR (400 MHz, $CDCl_3$)** δ 6.02 (s, 1H), 4.47 – 4.39 (m, 2H), 3.65 – 3.58 (m, 2H).

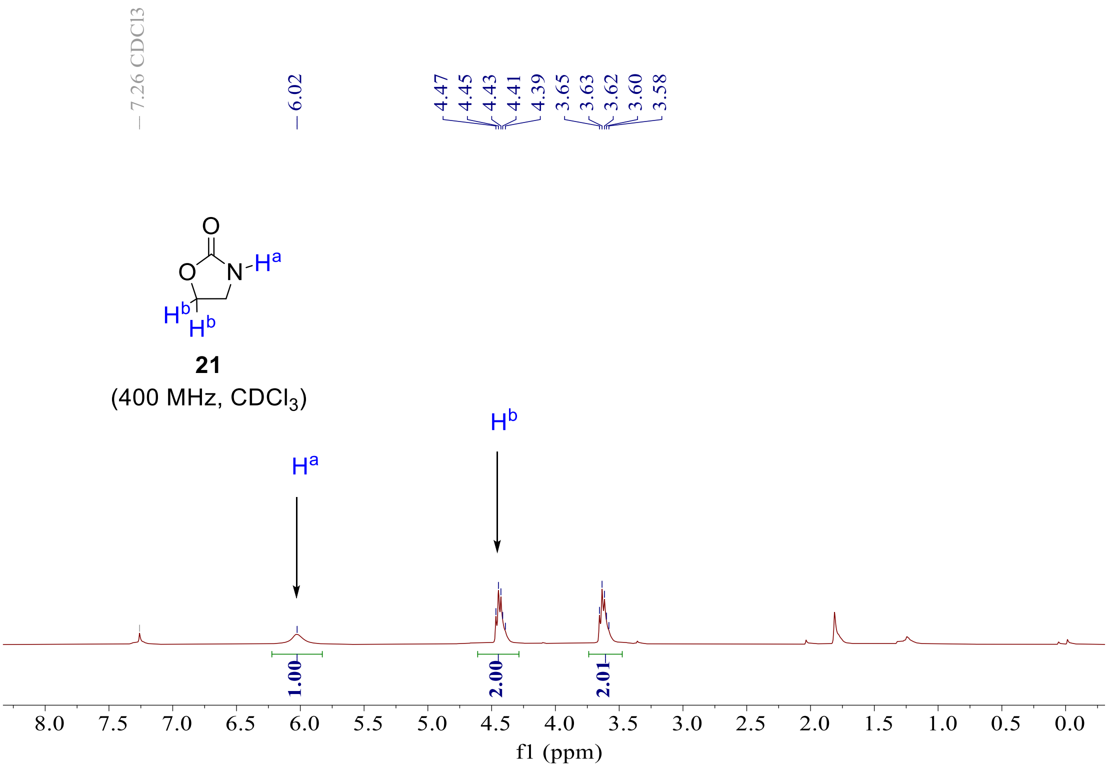


**Supplementary Figure S19. $^{1}$H NMR spectra for recycled oxazolidinone 21.**

## 8.2 Analysis of by-products in glycosylation reactions using deuterated catalyst

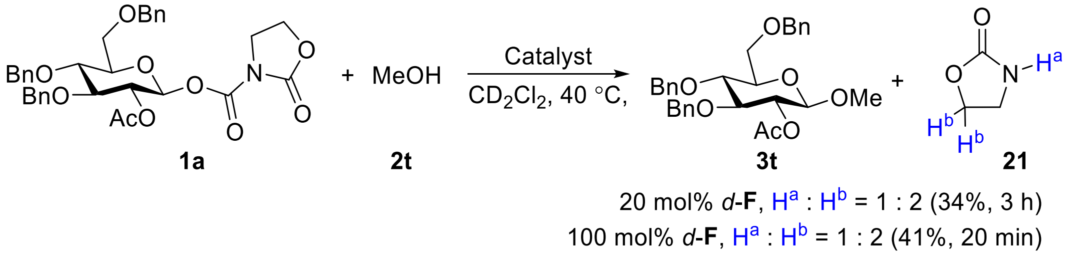


To an NMR tube was added glycosyl donor **1a** (0.0375 mmol, 1.5 equiv.), acceptor **2t** (0.025 mmol, 1.0 equiv.), ***d*-F** (20 or 100 mol%) and $CD_2Cl_2$ (0.5 mL, 0.05 M) under air atmosphere. The reaction mixture was placed at 40 °C water bath and determined the yield of glycosylated product **3t** and $H^a/H^b$ ratio of oxazolidinone by $^{1}$H NMR using 1,3,5-trimethylbenzene as an internal standard. As shown in **Supplementary Figure S13** and **Supplementary Figure S14** $H^a/H^b$ ratio of oxazolidinone is 1/2 when 20 mol% ***d*-F** or 100 mol% ***d*-F** was used in glycosylation. It is indicated $H^a$ of oxazolidinone **21** originate from receptors rather than catalysts.

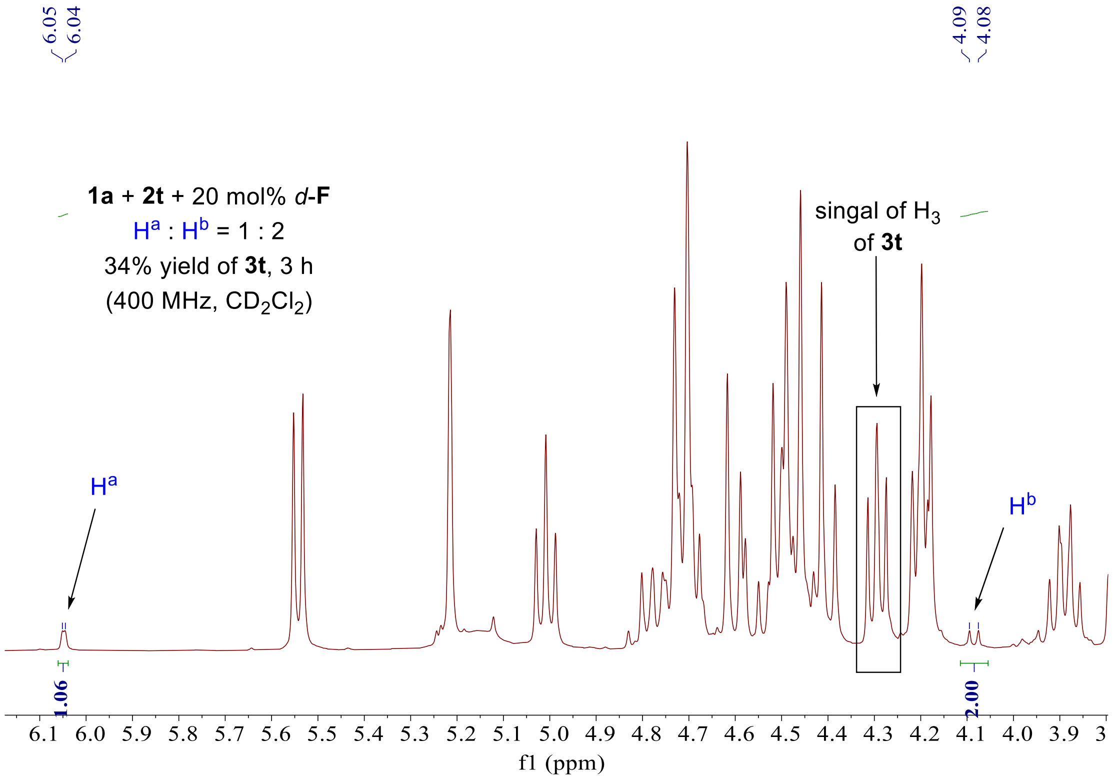


**Supplementary Figure S20. $^1$H NMR spectroscopic analysis of glycosylation with 20 mol% *d*-F**

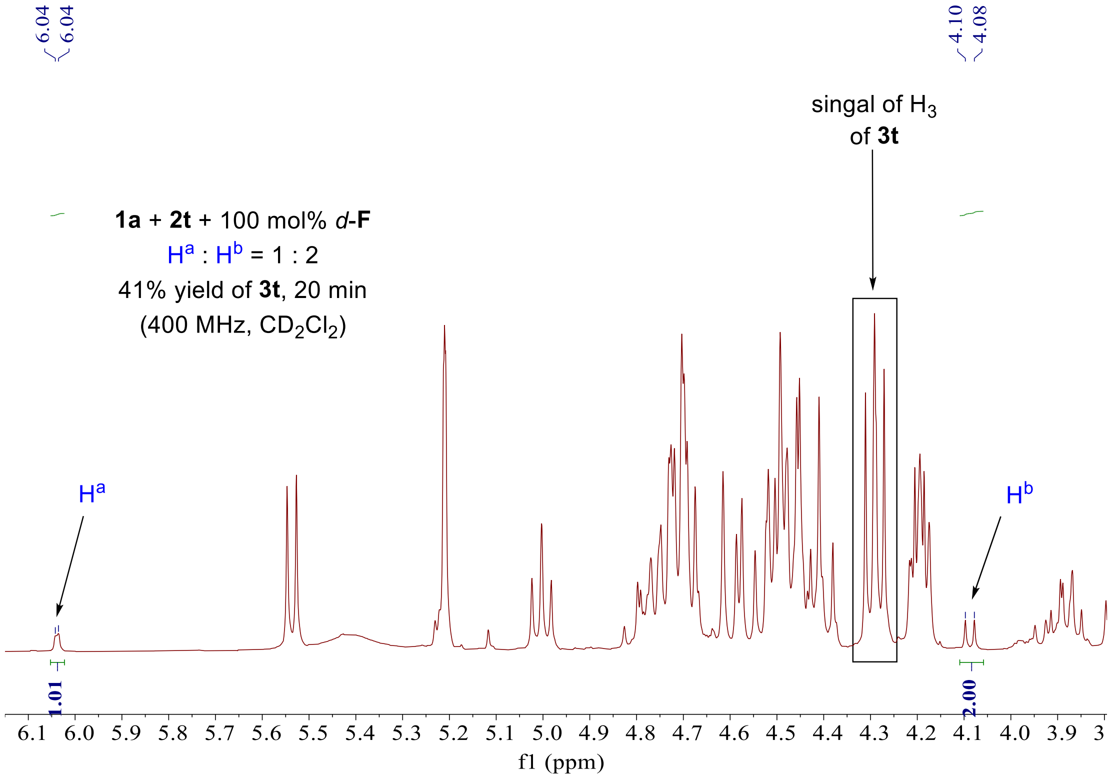


**Supplementary Figure S21. $^1$H NMR spectroscopic analysis of glycosylation with 100 mol% *d*-F.**

### 8.3 NMR titration study: catalyst-phenol interaction analysis

To an NMR tube was added **F** (7.9 mg, 0.02 mmol) and $CD_2Cl_2$ (0.5 mL, 0.04 M) under Air atmosphere. The mixture was determined the singal of **F** by $^1$H NMR (**Supplementary Figure S16**, entry 1).

Then to which acceptor **2a** (2.2 mg, 0.02 mmol, 1.0 equiv.) was added and determined the singal of **F** and **2a** by $^1$H NMR (**Supplementary Figure S16**, entry 2).

Then to which acceptor **2a** (8.9 mg, 0.08 mmol, 4.0 equiv.) was added and determined the singal of **F** and **2a** by $^1$H NMR (**Supplementary Figure S16**, entry 3).

Then to which acceptor **2a** (33.3 mg, 0.3 mmol, 15.0 equiv.) was added and determined the singal of **F** and **2a** by $^1$H NMR (**Supplementary Figure S16**, entry 4).

As shown in **Supplementary Figure S16**, significant upfield shift were observed for the protons of catalyst **F**, accompanied by the disappearance of the phenolic hydroxyl proton signal.

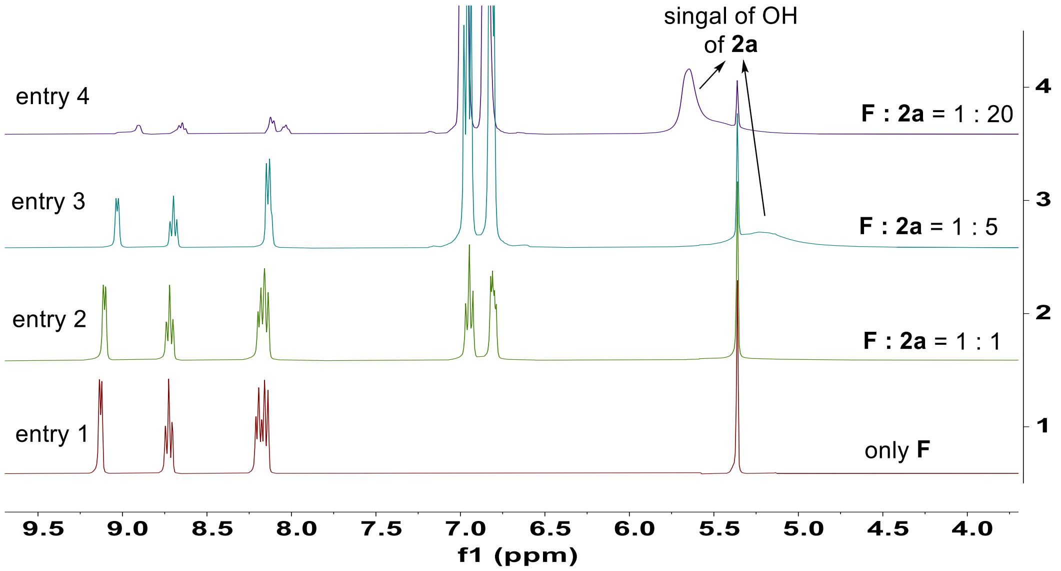


**Supplementary Figure S22. $^1$H NMR spectroscopic analysis of catalyst-phenol interactions.**

## 8.4 Kinetic Isotope Effect (KIE) analysis of glycosylation with MeOH and MeOD

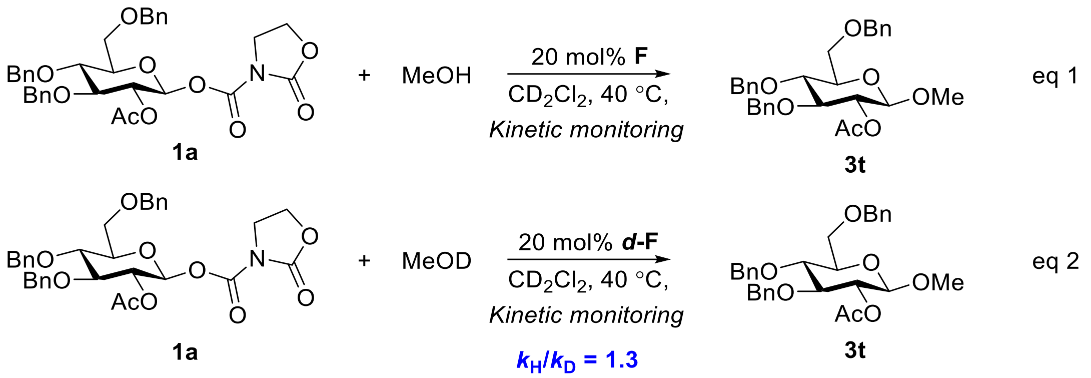


To an NMR tube was added glycosyl donor **1a** (22.7 mg, 0.0375 mmol, 1.5 equiv.), acceptor MeOH or MeOD (0.025 mmol, 1.0 equiv.), **F** or ***d*-F** (20 mol%) and $CD_2Cl_2$ (0.5 mL, 0.05 M) under Air atmosphere. The reaction mixture was placed at 40 °C water bath and determined the yield of glycosylated product **3t** by $^1$H NMR using 1,3,5-trimethylbenzene as an internal standard.

The glycosylation of **1a** to **3s** were detected by different reaction time.

**Supplementary Table S4. The data of conversion of 1a to 3t.**

| Time (min) | Yield of **3t** for eq 1 (%) | Conc of **3t** for eq 1 (mM) | Yield of **3t** for eq 2 (%) | Conc of **3t** for eq 2 (mM) |
|---|---|---|---|---|
| 0 | 0 | 0 | 0 | 0 |
| 5 | 3.7 | 1.9 | 4.4 | 2.2 |
| 10 | 5.5 | 2.8 | 5.3 | 2.7 |
| 30 | 13.1 | 6.6 | 11.3 | 5.7 |
| 60 | 25.5 | 12.8 | 19.8 | 9.9 |
| 120 | 44.8 | 22.4 | 34.1 | 17.1 |
| 180 | 57.8 | 28.9 | 43.7 | 21.9 |
| 240 | 67.4 | 33.7 | 53.3 | 26.7 |
| 300 | 71.6 | 35.8 | 56.9 | 28.5 |
| 420 | 76.5 | 38.3 | 65.9 | 33.0 |
| 540 | 77.8 | 38.9 | 74.7 | 37.4 |

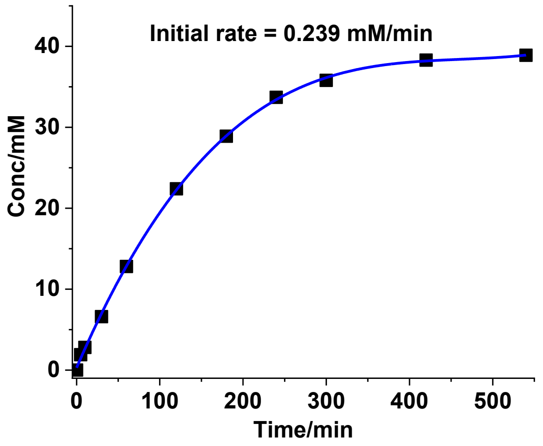


| | |
|---|---|
| Equation | $y$ = Intercept + B1*$x$ + B2*$x^2$ + B3*$x^3$ |
| Intercept | 0.28523 ± 0.17655 |
| B1 | 0.23867 ± 0.00433 |
| B2 | $-5.06993\times10^{-4} \pm 2.18069\times10^{-5}$ |
| B3 | $3.65743\times10^{-7} \pm 2.75936\times10^{-8}$ |
| Residual sum of squares | 0.68883 |
| $R^2$ (COD) | 0.99972 |
| Adjusted $R^2$ | 0.9996 |

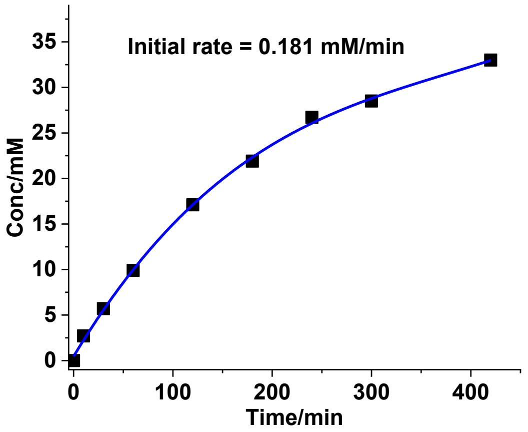


| | |
|---|---|
| Equation | $y$ = Intercept + B1*$x$ + B2*$x^2$ + B3*$x^3$ |
| Intercept | 0.46705 ± 0.33313 |
| B1 | 0.18074 ± 0.00977 |
| B2 | $-3.92045\times10^{-4} \pm 5.99691\times10^{-5}$ |
| B3 | $3.47624\times10^{-7} \pm 9.43361\times10^{-8}$ |
| Residual sum of squares | 1.12835 |
| $R^2$ (COD) | 0.99904 |
| Adjusted $R^2$ | 0.99846 |

$K_H/K_D = 0.239/0.181 = 1.3$

**Supplementary Figure S23. Kinetic isotope effect analysis of glycosylation with MeOH and MeOD.**

## 8.5 Competition experiment between glycosyl donors 1j and *d*-1j

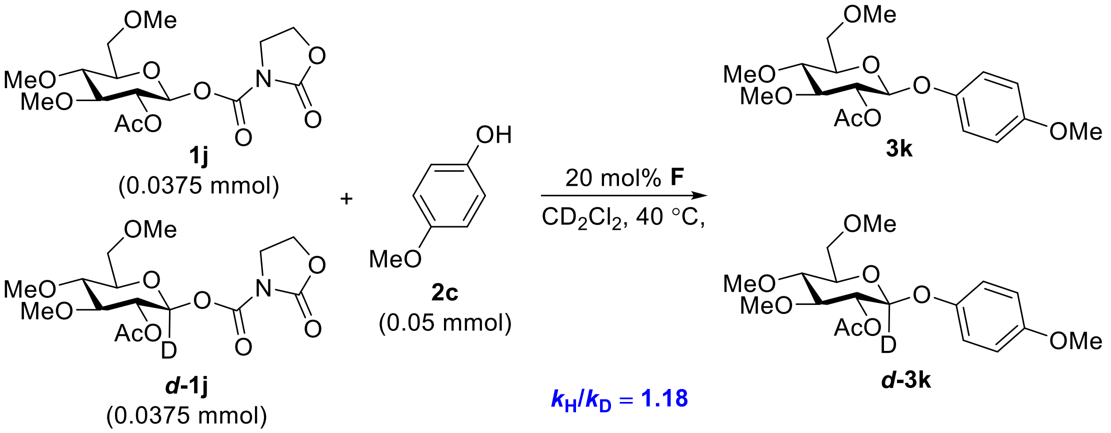


To an NMR tube was added glycosyl donor **1j** (14.1 mg, 0.0375 mmol, 1.5 equiv.), donor ***d*-1j** (97%D, 14.6 mg, 0.0375 mmol, 1.5 equiv.), $CD_2Cl_2$ (1 mL, 0.05 M), and determined the **1j**/***d*-1j** ratio by $^1$H NMR. Then to which acceptor **2c** (6.2 mg, 0.05 mmol, 1.0 equiv.), **F** (4.0 mg, 20 mol%) under Air atmosphere. The reaction mixture was placed at 40 °C water bath and determined the yield of glycosylated product **3k** and ***d*-3k** by $^1$H NMR using 1,3,5-trimethylbenzene as an internal standard.

KIE determinations from the reaction mixture were carried out three times at different time point, yielding values of 1.38, 1.05, and 1.11, respectively. The average KIE from these three measurements was calculated to be 1.18. Subsequently, the reaction product was isolated and analyzed by $^1$H NMR spectroscopy. $^1$H NMR integration of the isolated product yielded a KIE of 1.13. These two independently determined KIE values are in close agreement.

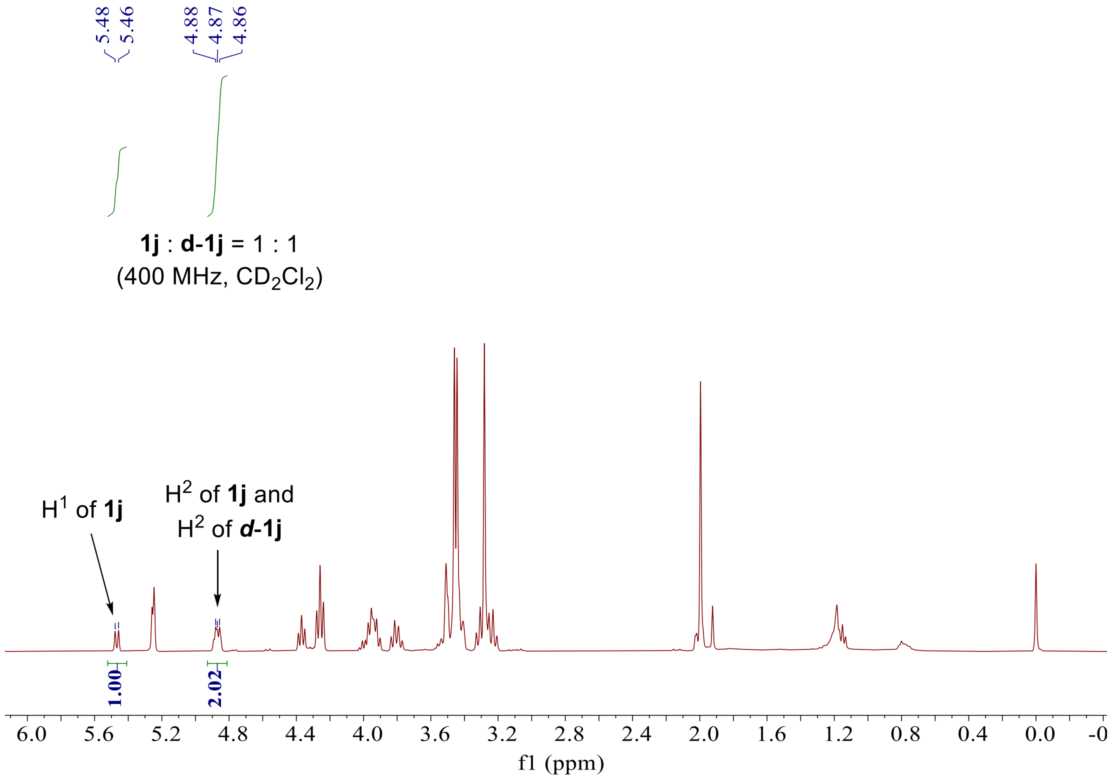


**Supplementary Figure S24.** **$^1$H NMR spectra analysis of 1j and *d*-1j in $CD_2Cl_2$.**

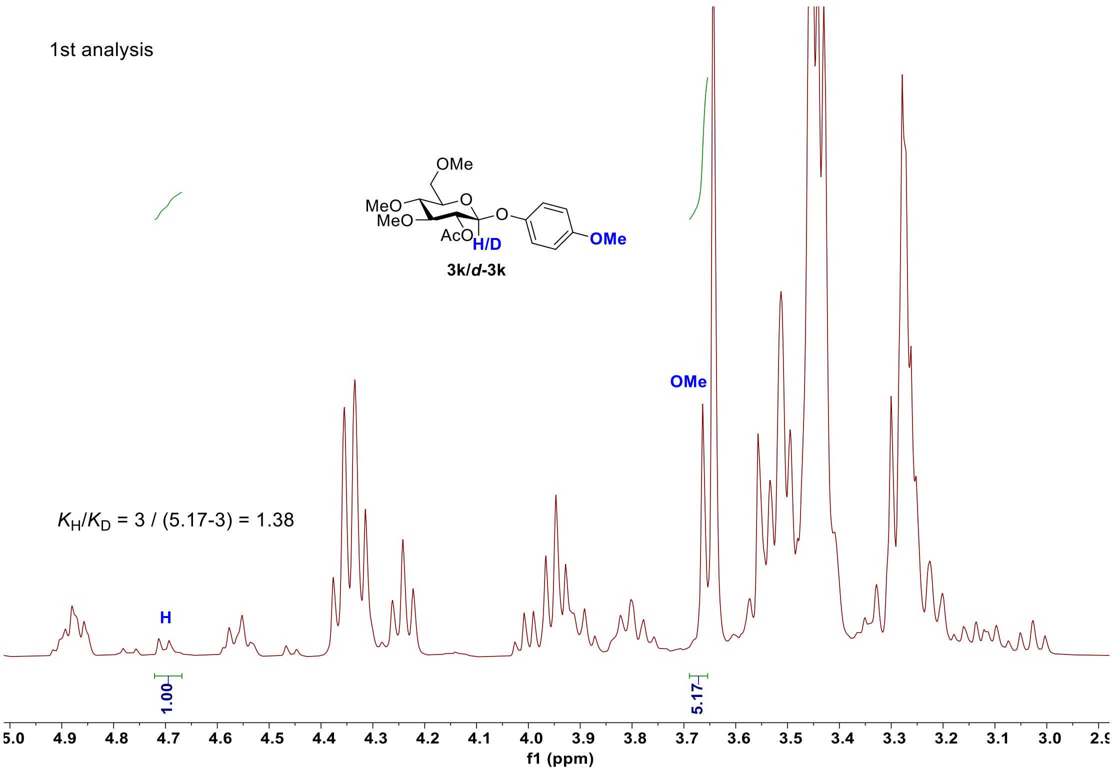

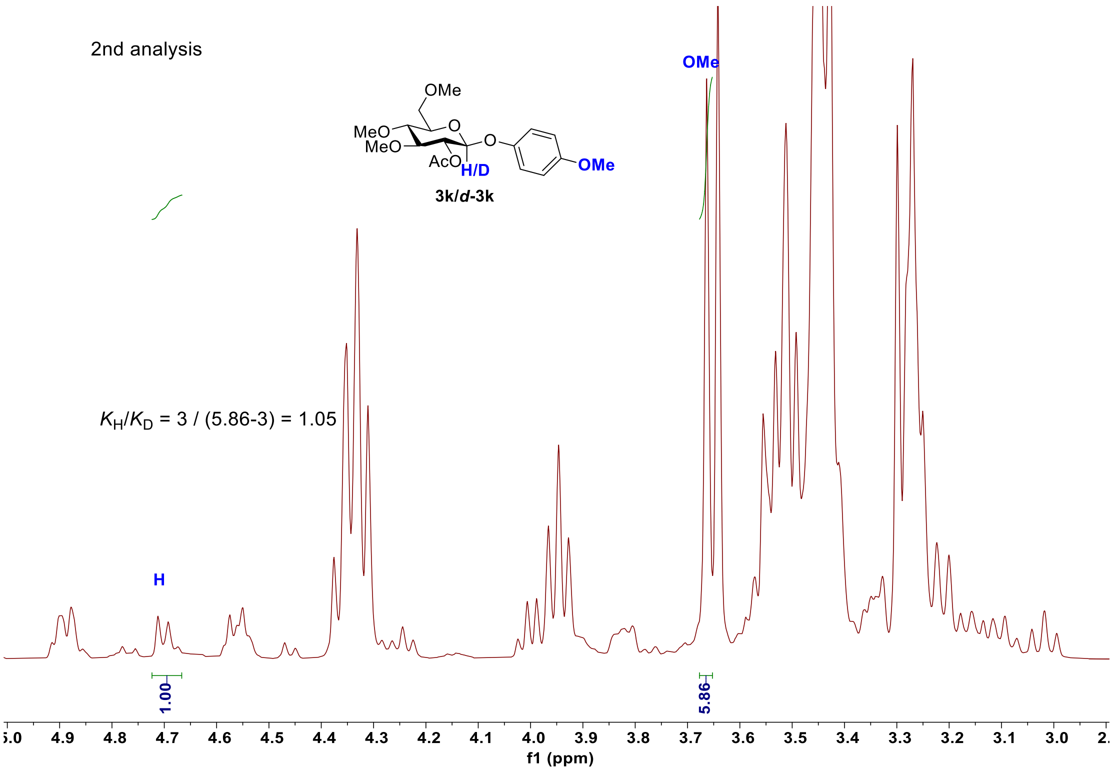


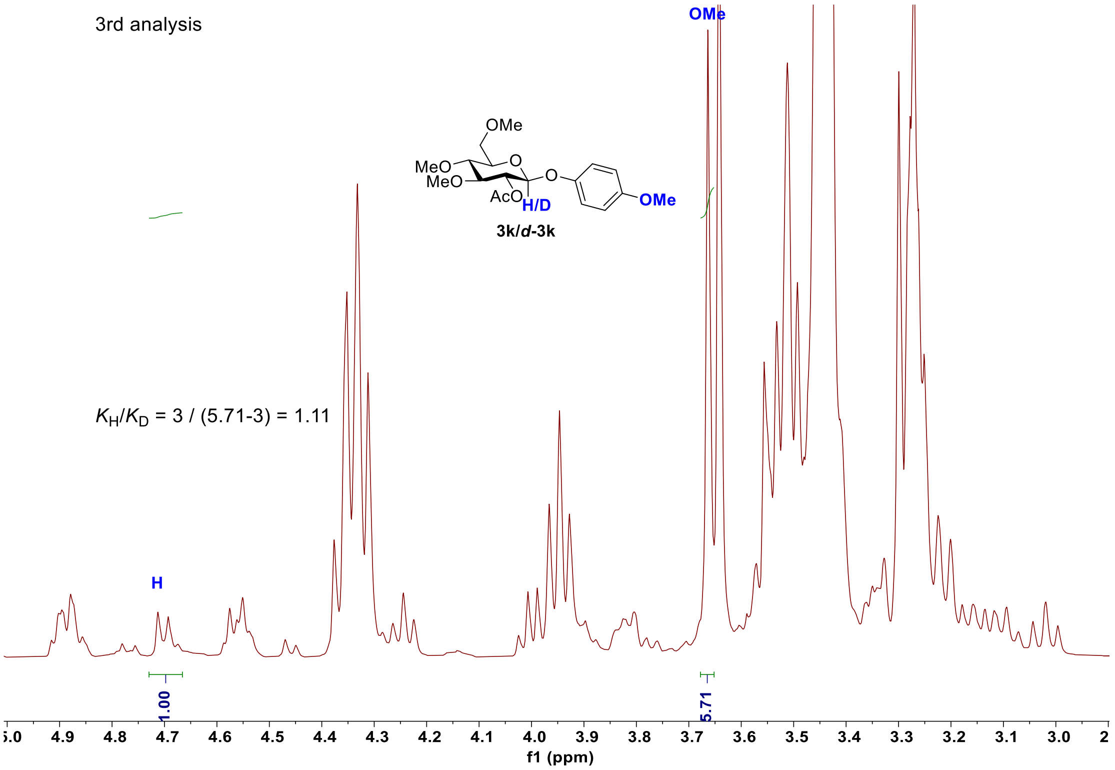


**Supplementary Figure S25. Crude $^{1}$H NMR spectra analysis of competition experiment.**

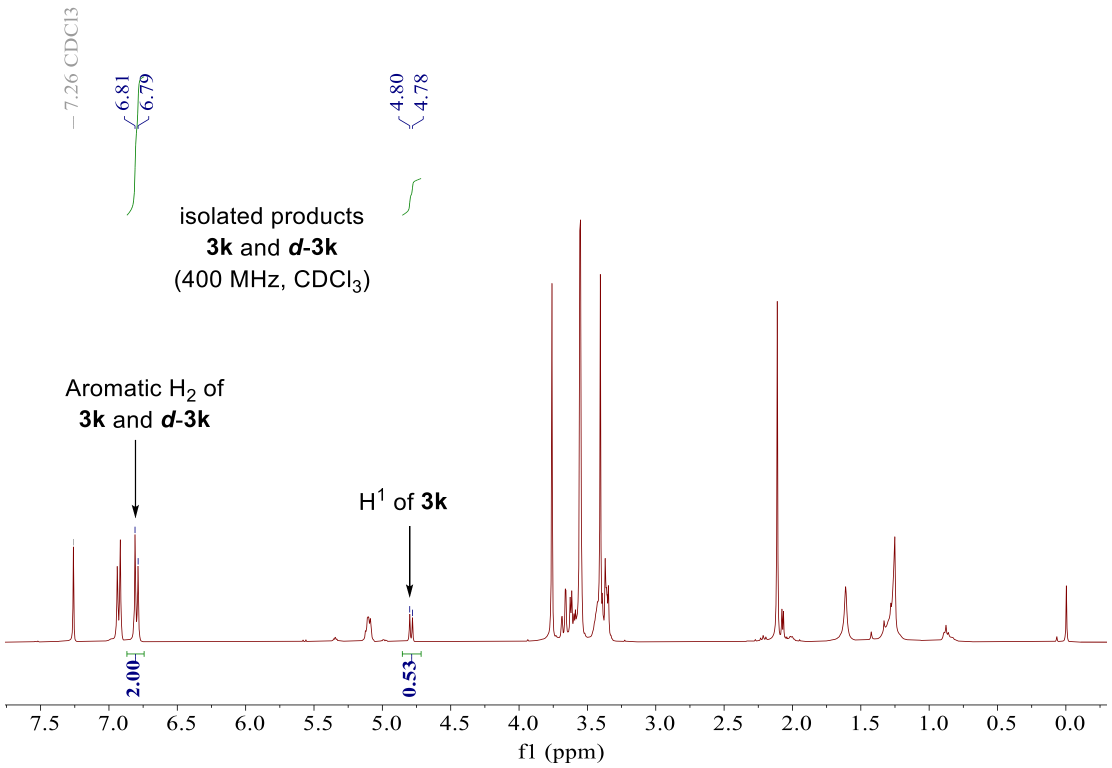


Yield of **3k** : Yield of ***d*-3k** = 0.53 : (1-0.53), $K_{H/D}$ =1.13

**Supplementary Figure S26. $^{1}$H NMR spectra analysis of isolated products 3k and *d*-3k.**

## 8.6 Study on the stability of glycosyl carbamate donor 1a

The glycosyl carbamate donors **1a** are stable and stay inert at room temperature for at least three months.

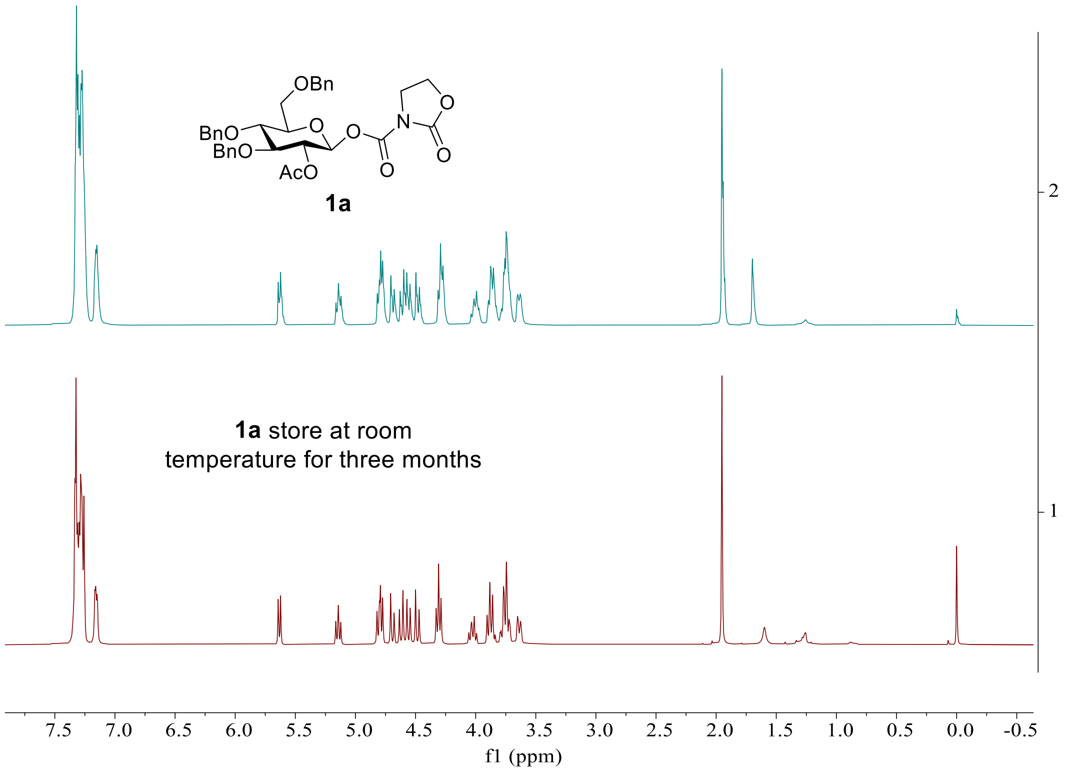


**Supplementary Figure S27. $^1$H NMR spectra analysis of 1a store for three months.**

### 8.7 Proposed mechanism

Based on experimental evidence and literature precedents[16,17], a plausible mechanism is proposed. The reaction starts with the formation of **Int 1**; which subsequently activates the glycosyl donor **1**, leading to the generation of the oxocarbenium (**Int 2**) with concomitant release of $CO_2$ and oxazolidinone **21**. In this process, the hydroxyl proton of **Int 1** is proposed to interact with the carbonyl groups of the glycosyl donor **1**, while the neighboring participation of the 2-OAc group facilitates the departure of the anomeric leaving group, thereby promoting the efficient formation of the oxocarbenium **Int 2**. Concurrently, **Int 1** is converted into **Int 3**. Finally, **Int 2** is intercepted either by the glycosyl acceptor **2** (path 1) or **Int 3** (path 2) to afford the product **3**.

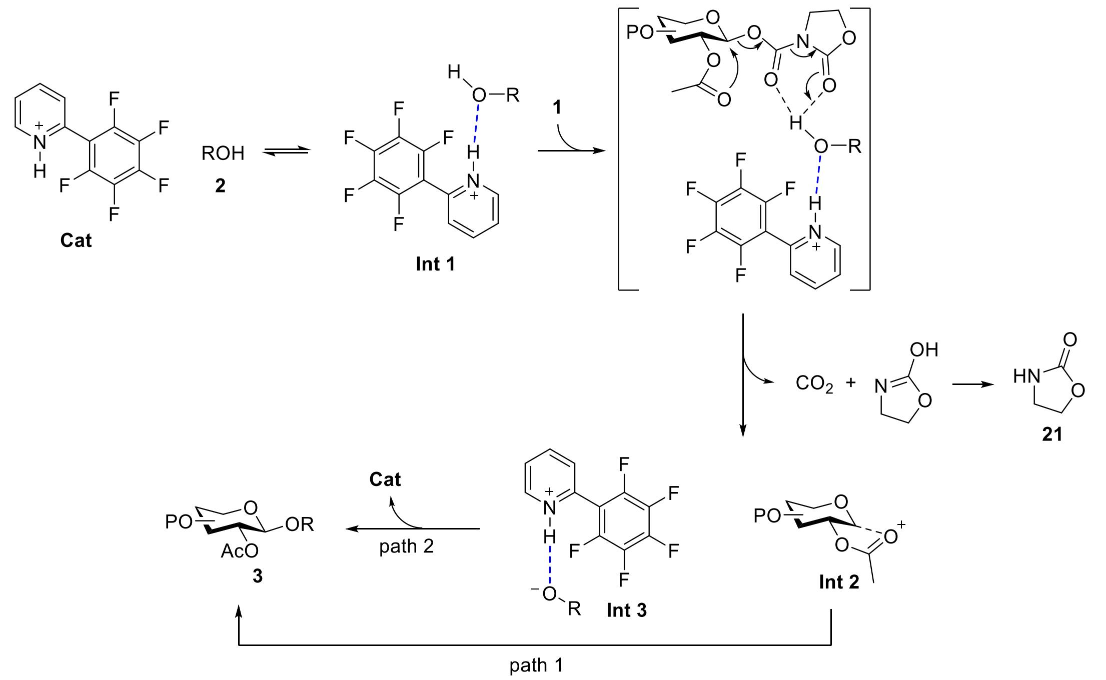


**Supplementary Figure S28. Proposed mechanism.**

## 9. X-ray crystal structure data of compound 1a

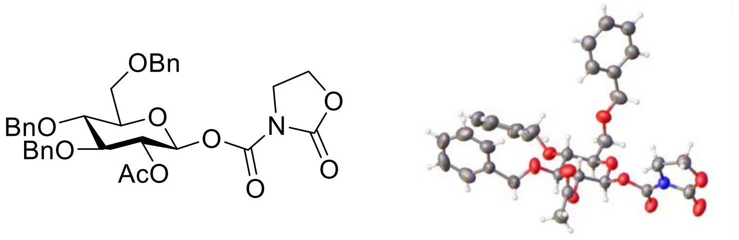


**1a** **CCDC 2433567**

**Supplementary Figure S29. X-ray crystal structure data of 1a.**

Single crystals suitable for X-ray studies were grown by slow evaporation of a

solution of compound **1a** in a mixture of petroleum ether and $CH_2Cl_2$ at room temperature.

The preparation of crystal of **1a**: compound **1a** (120 mg) was dissolved in $CH_2Cl_2$ (1.0 mL) at room temperature. Petroleum ether (5.0 mL) was dropped carefully to the mixture. Then, the flask was capped with thin film. Finally, a needle crystal was obtained after 7 days.

**Supplementary Table S5. Crystal data and structure refinement details for compound 1a.**

| Compound | **1a** |
|---|---|
| Identification code | CCDC 2433567 |
| Empirical formula | $C_{33}H_{35}NO_{10}$ |
| Formula weight | 605.62 |
| Temperature (K) | 296(2) |
| Crystal system | monoclinic |
| Space group | $Pca2_1$ |
| a (Å) | 11.3842(9) |
| b (Å) | 6.3603(5) |
| c (Å) | 22.1715(15) |
| α (°) | 90 |
| β (°) | 103.391(2) |
| γ (°) | 90 |
| Volume (Å3) | 1561.7(2) |
| Z | 2 |
| Dc(Mg cm$^{-3}$) | 1.288 |
| μ (mm$^{-1}$) | 0.096 |
| F(000) | 640.0 |
| 2θ range for data collection (°) | 4.51 to 52.95 |
| Index ranges | $-14 \leq h \leq 14$, $-7 \leq k \leq 7$, $-26 \leq l \leq 27$ |
| Reflections collected | 27694 |
| Independent reflections | 6343 [$R_{int}$ = 0.1057, $R_{sigma}$ = 0.0854] |
| Goodness-of-fit on $F^2$ | 1.133 |
| Final R indexes [I>=2σ (I)] | $R_1$ = 0.0745 $\omega R_2$ = 0.1902 |
| R indices (all data) | $R_1$ = 0.1387 $\omega R_2$ = 0.2647 |
| Largest diff. peak and hole [eÅ$^{-3}$] | 0.50/-0.34 |

# 10. NMR Spectra

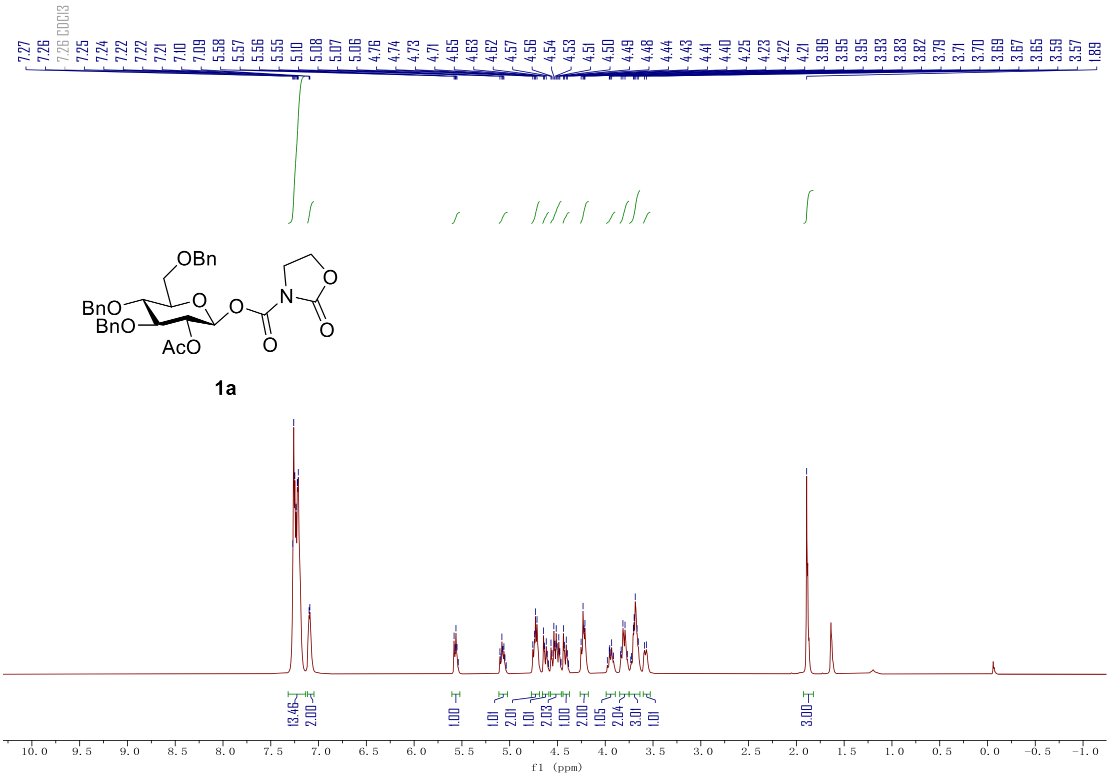

Supplementary Figure S30. $^{1}H$ NMR (400 MHz, $CDCl_3$) Spectra for compound 1a

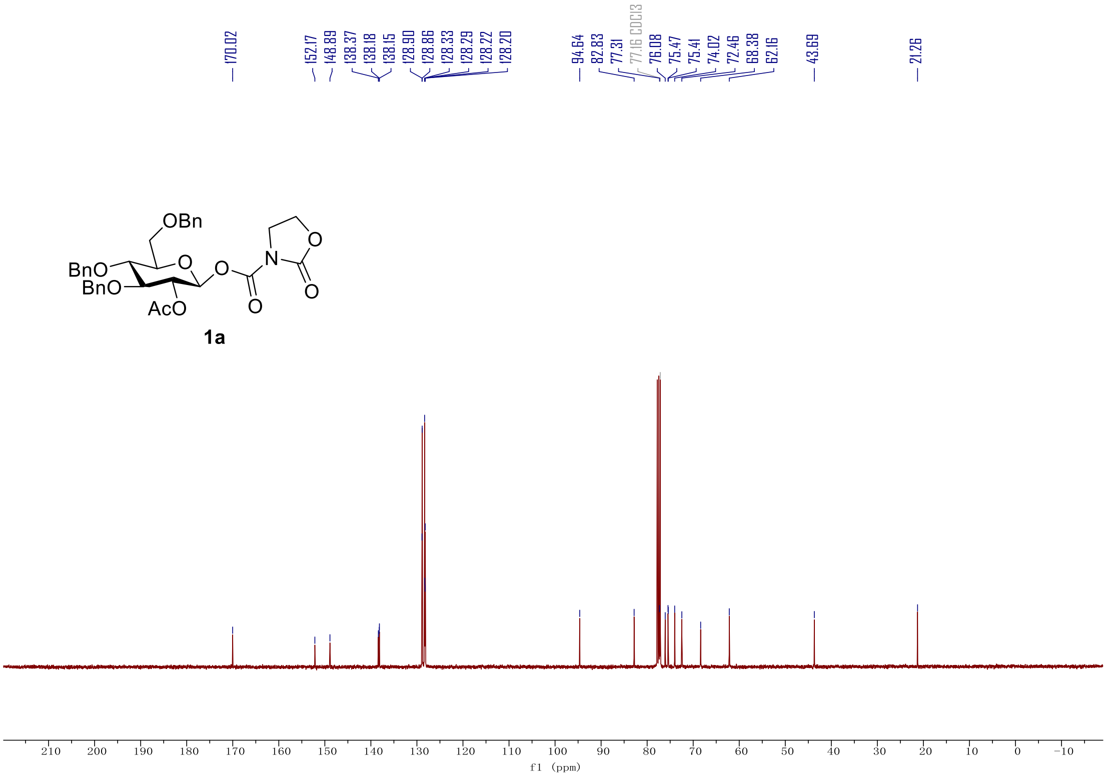

Supplementary Figure S31. $^{13}C$ NMR (101 MHz, $CDCl_3$) Spectra for compound 1a

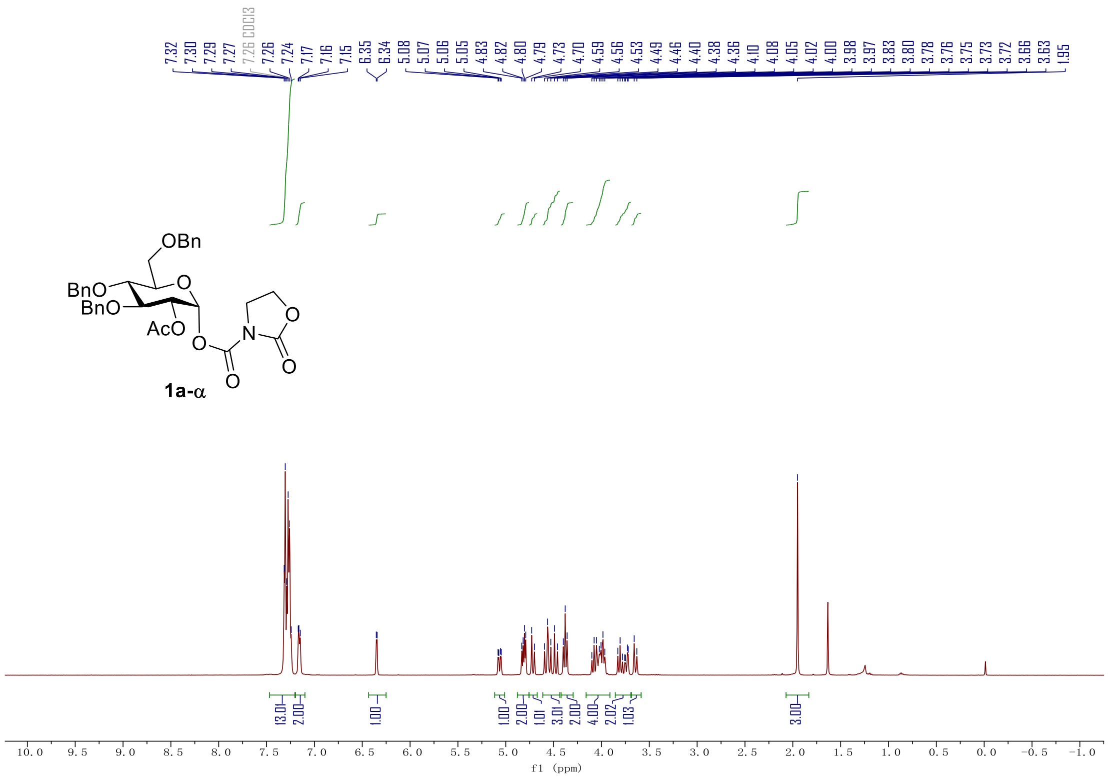


Supplementary Figure S32. $^{1}$H NMR (400 MHz, $CDCl_3$) Spectra for compound 1a-α

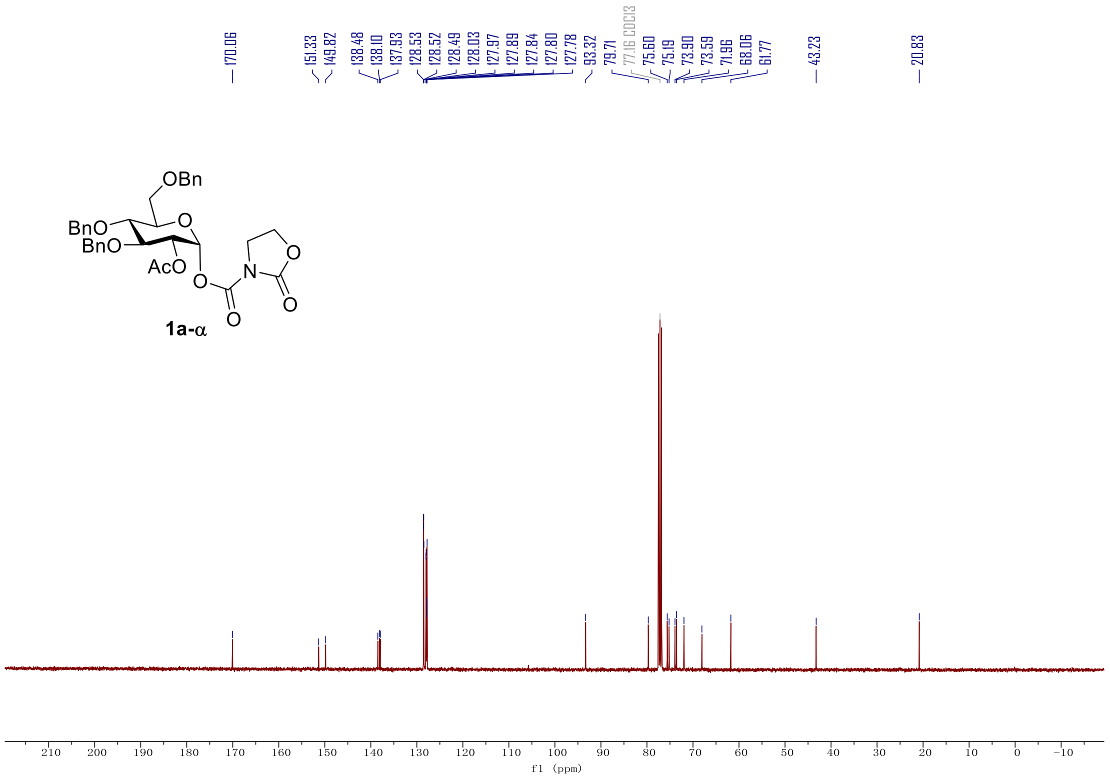


Supplementary Figure S33. $^{13}$C NMR (101 MHz, $CDCl_3$) Spectra for compound 1a-α

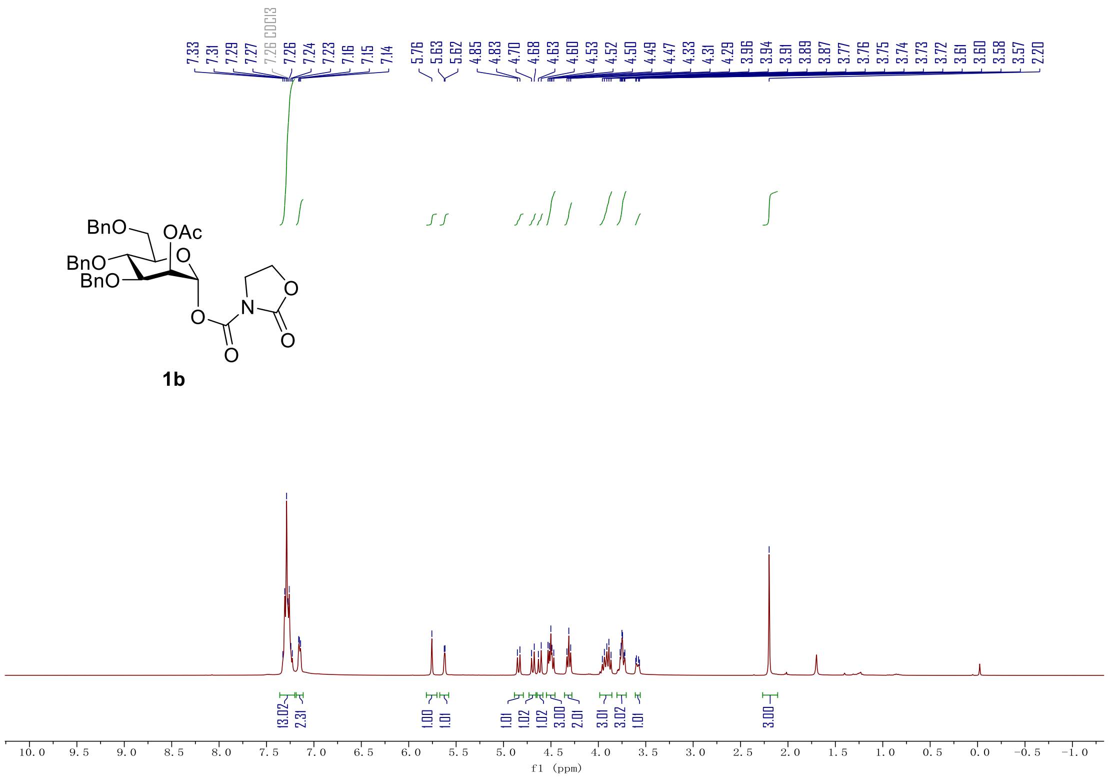


**Supplementary Figure S34. $^{1}$H NMR (400 MHz, $CDCl_3$) Spectra for compound 1b**

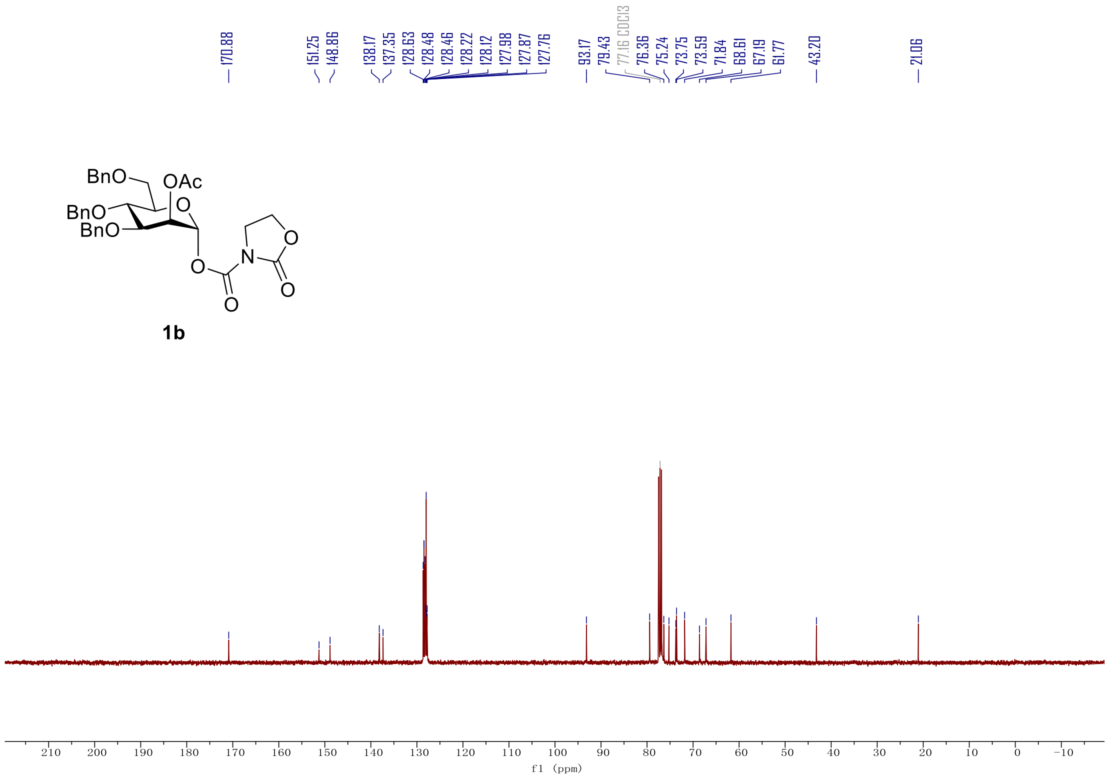


**Supplementary Figure S35. $^{13}$C NMR (101 MHz, $CDCl_3$) Spectra for compound 1b**

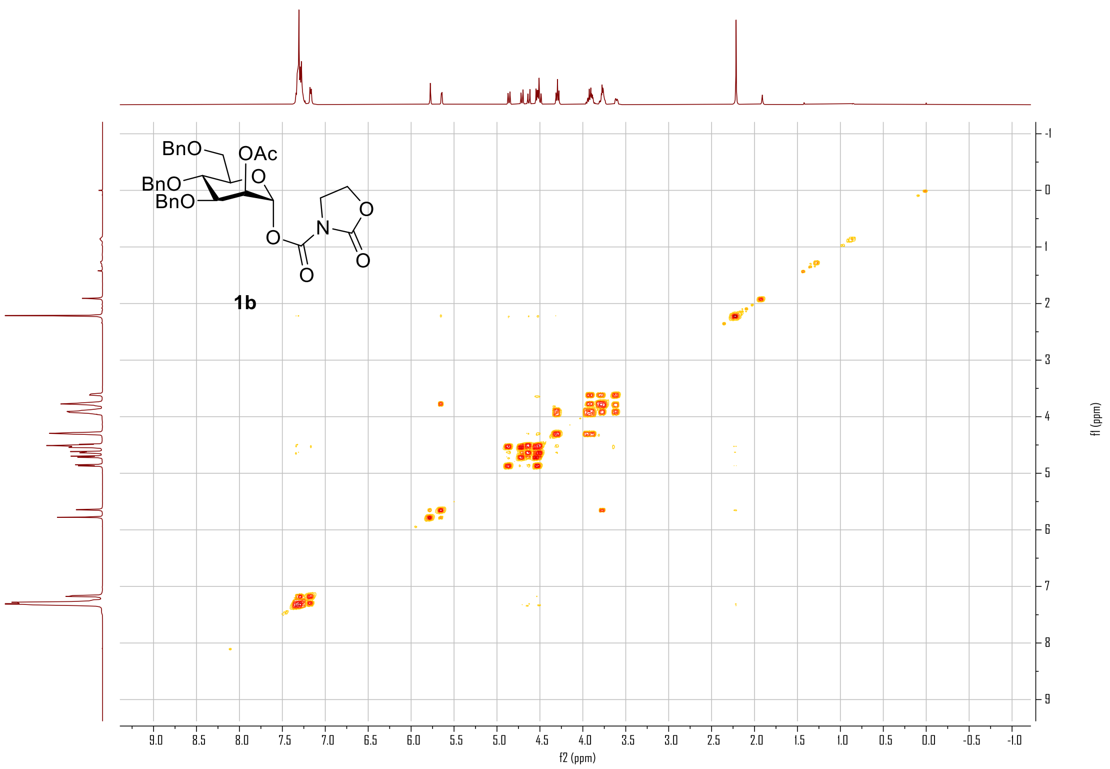


**Supplementary Figure S36. COSY (500 MHz, $CDCl_3$) Spectrum for compound 1b**

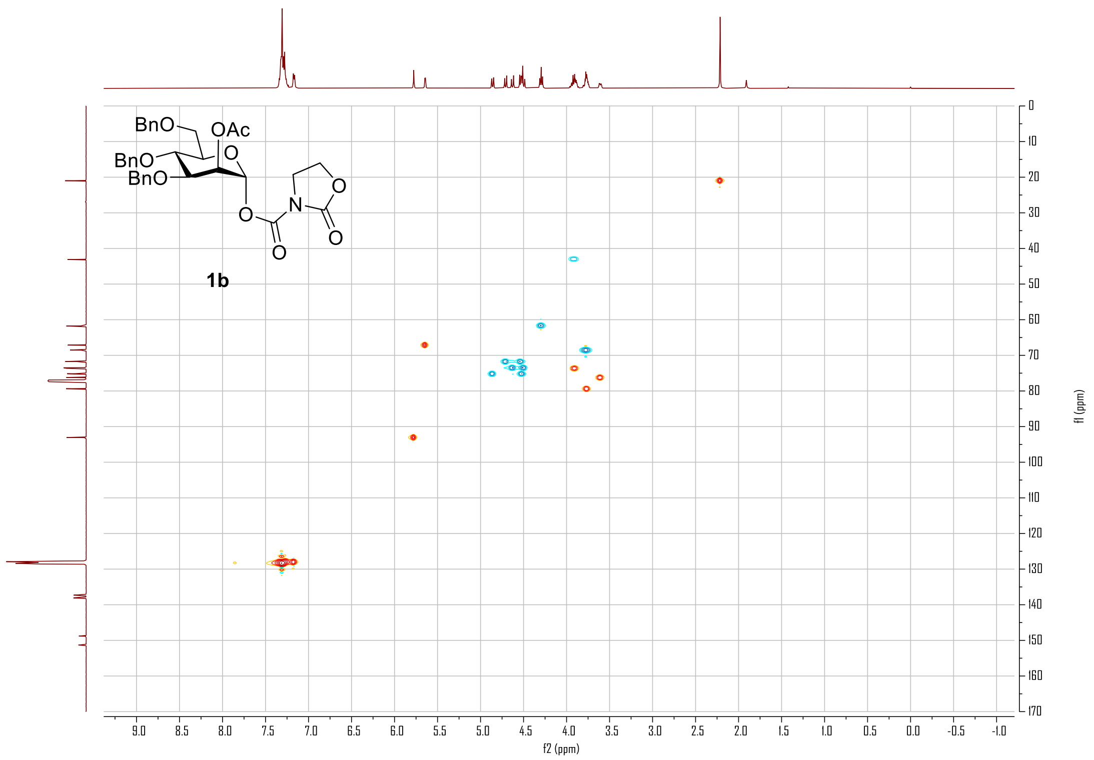


**Supplementary Figure S37. HSQC (500 MHz, $CDCl_3$) Spectrum for compound 1b**

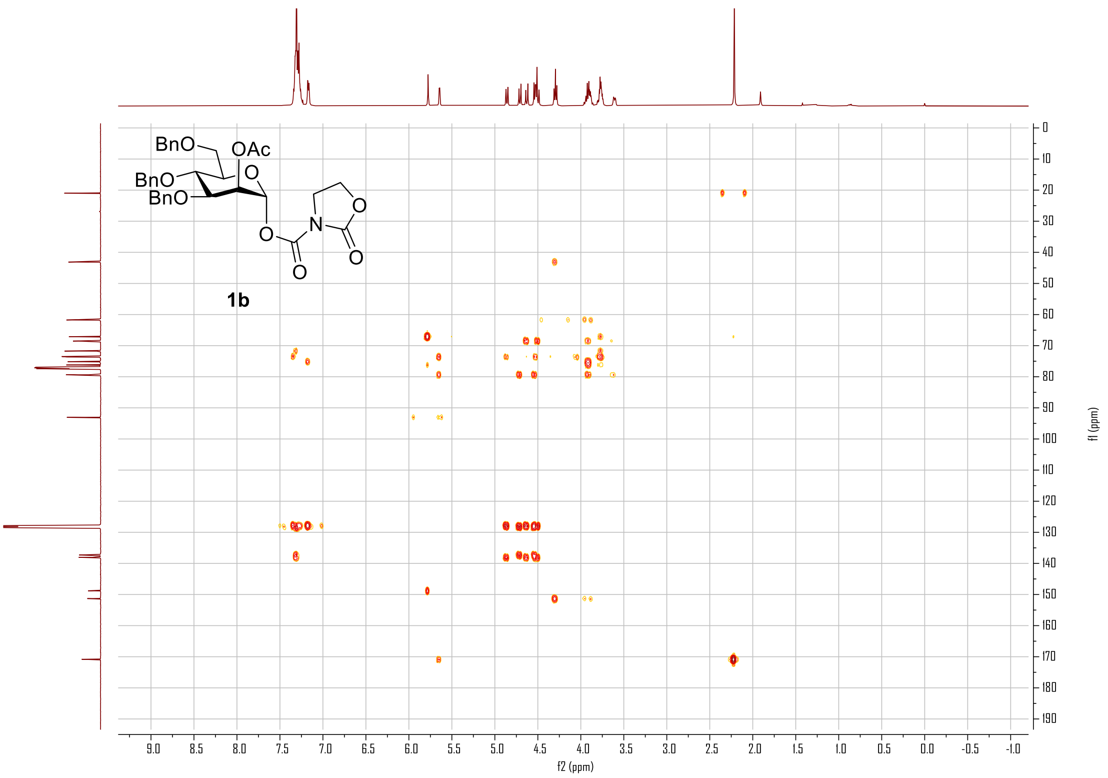


**Supplementary Figure S38. HMBC (500 MHz, $CDCl_3$) Spectrum for compound 1b**

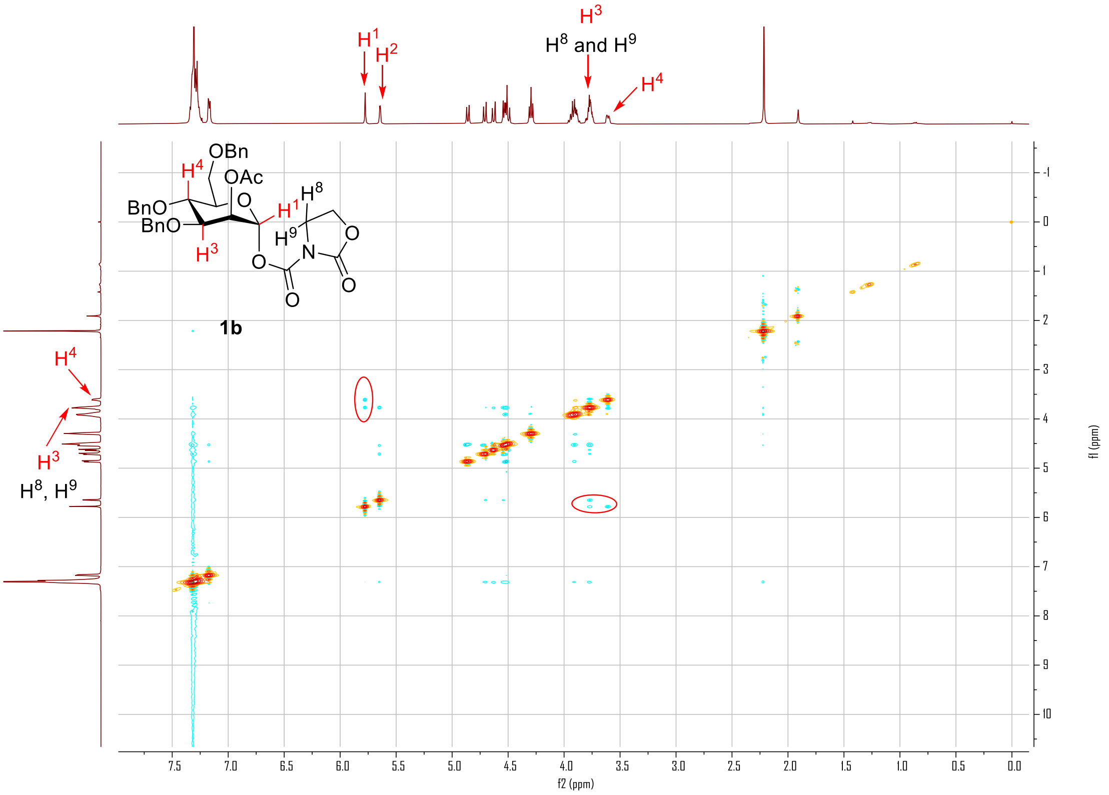


**Supplementary Figure S39. NOESY (500 MHz, $CDCl_3$) Spectrum for compound 1b**

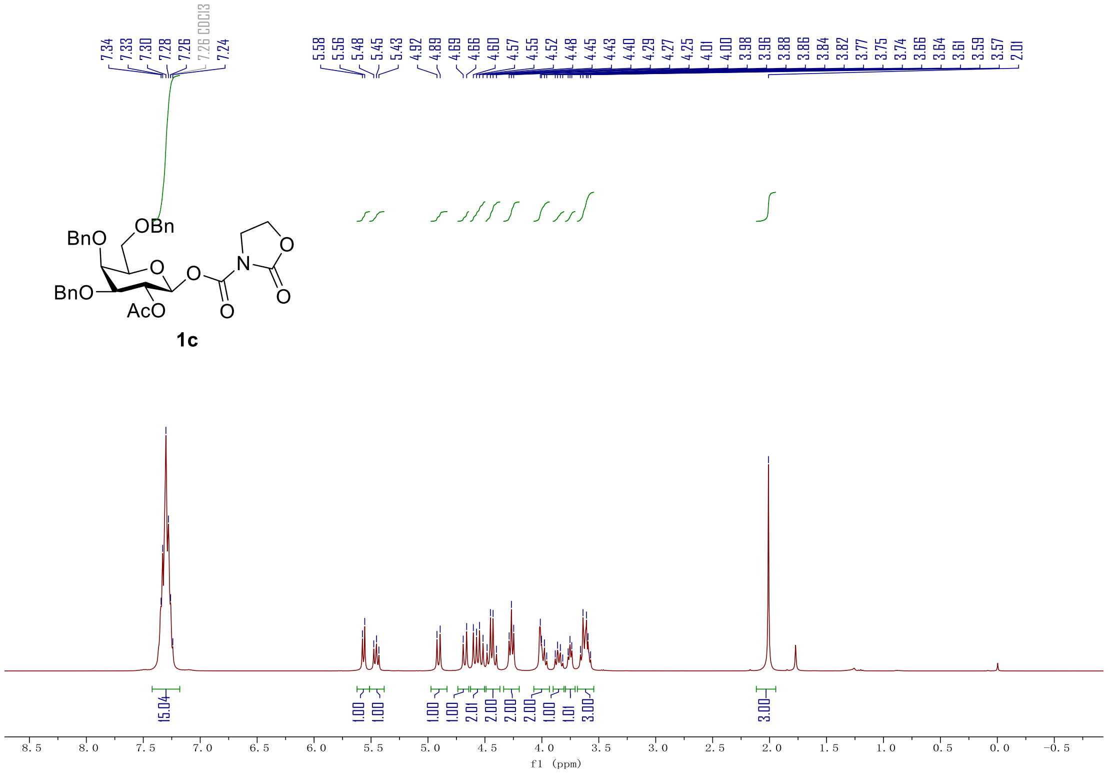


**Supplementary Figure S40.** $^{1}$H NMR (400 MHz, $CDCl_3$) Spectra for compound 1c

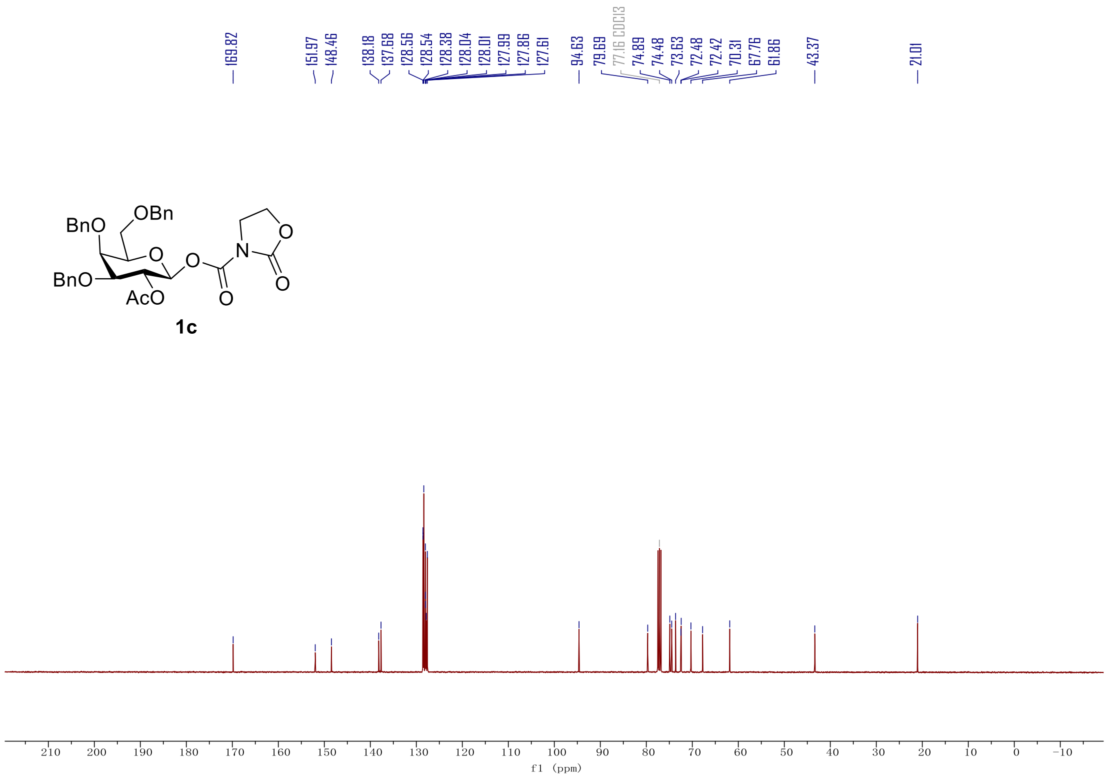


**Supplementary Figure S41.** $^{13}$C NMR (101 MHz, $CDCl_3$) Spectra for compound 1c

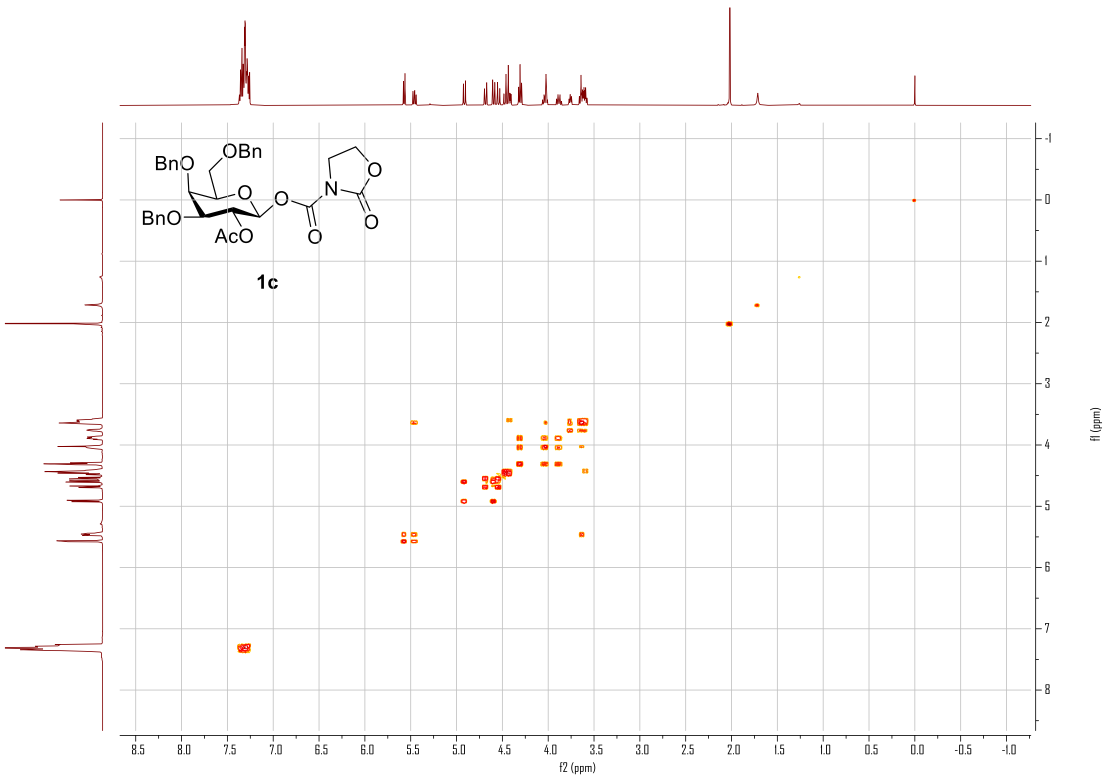


**Supplementary Figure S42. COSY (500 MHz, $CDCl_3$) Spectrum for compound 1c**

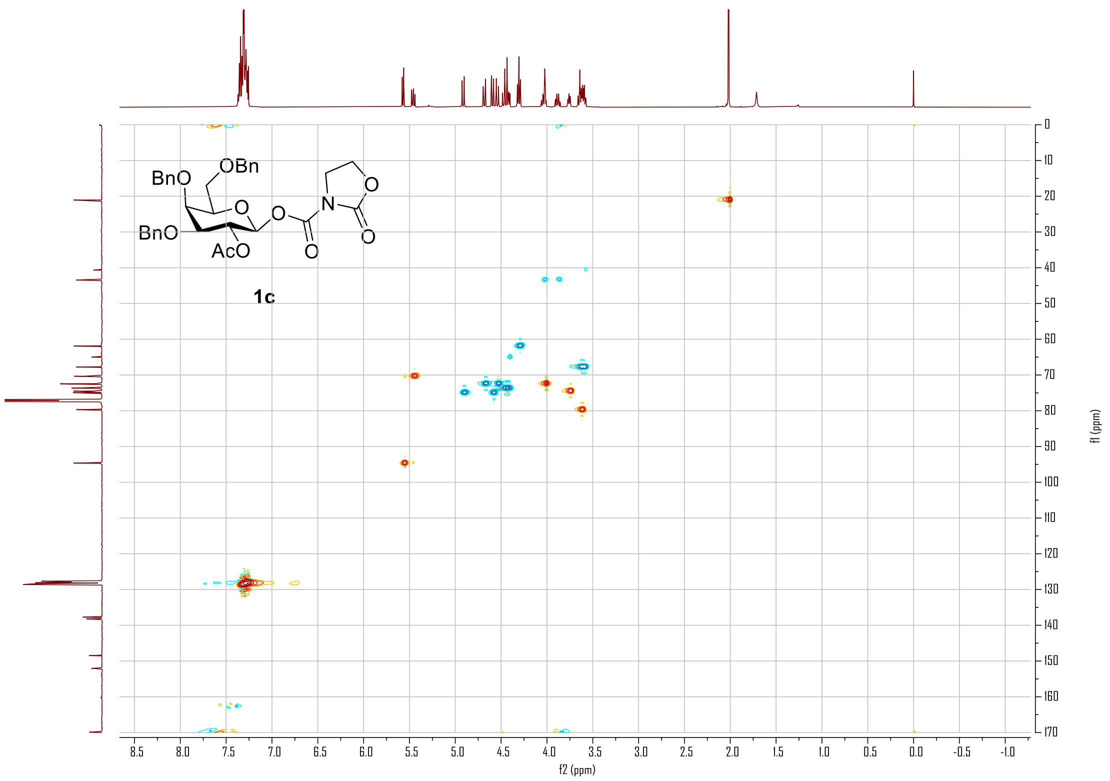


**Supplementary Figure S43. HSQC (500 MHz, $CDCl_3$) Spectrum for compound 1c**

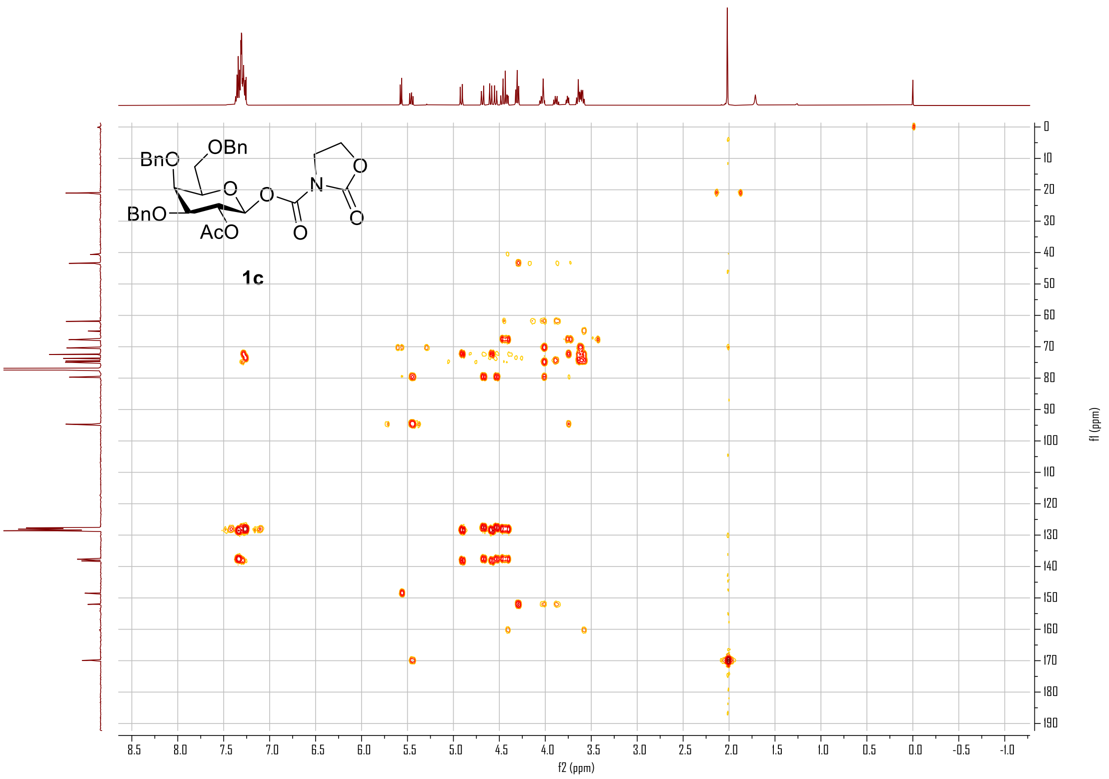


**Supplementary Figure S44. HMBC (500 MHz, $CDCl_3$) Spectrum for compound 1c**

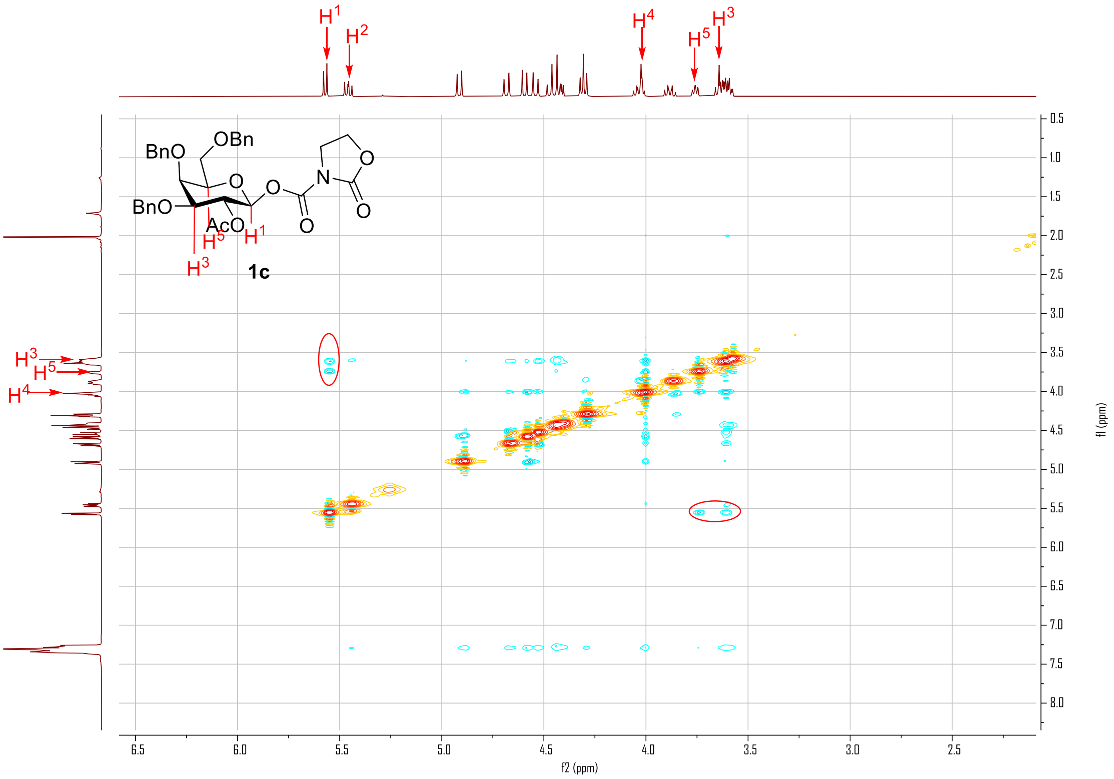


**Supplementary Figure S45. NOESY (500 MHz, $CDCl_3$) Spectrum for compound 1c**

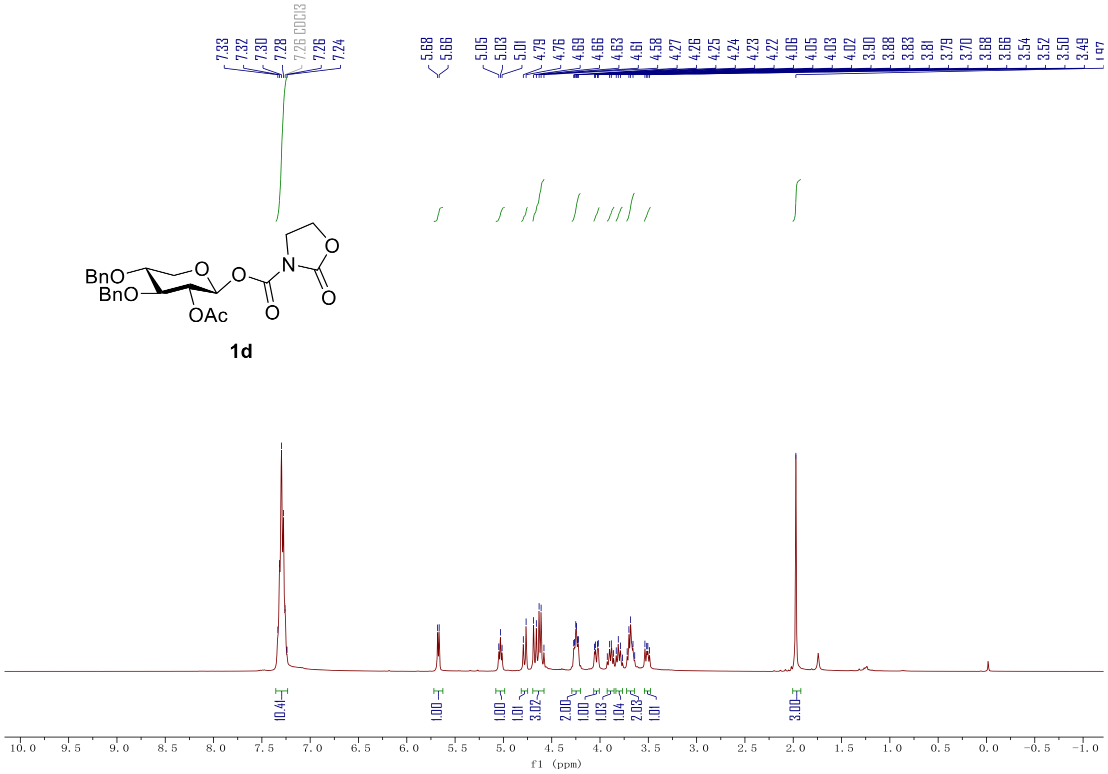


**Supplementary Figure S46. $^{1}H$ NMR (400 MHz, $CDCl_3$) Spectra for compound 1d**

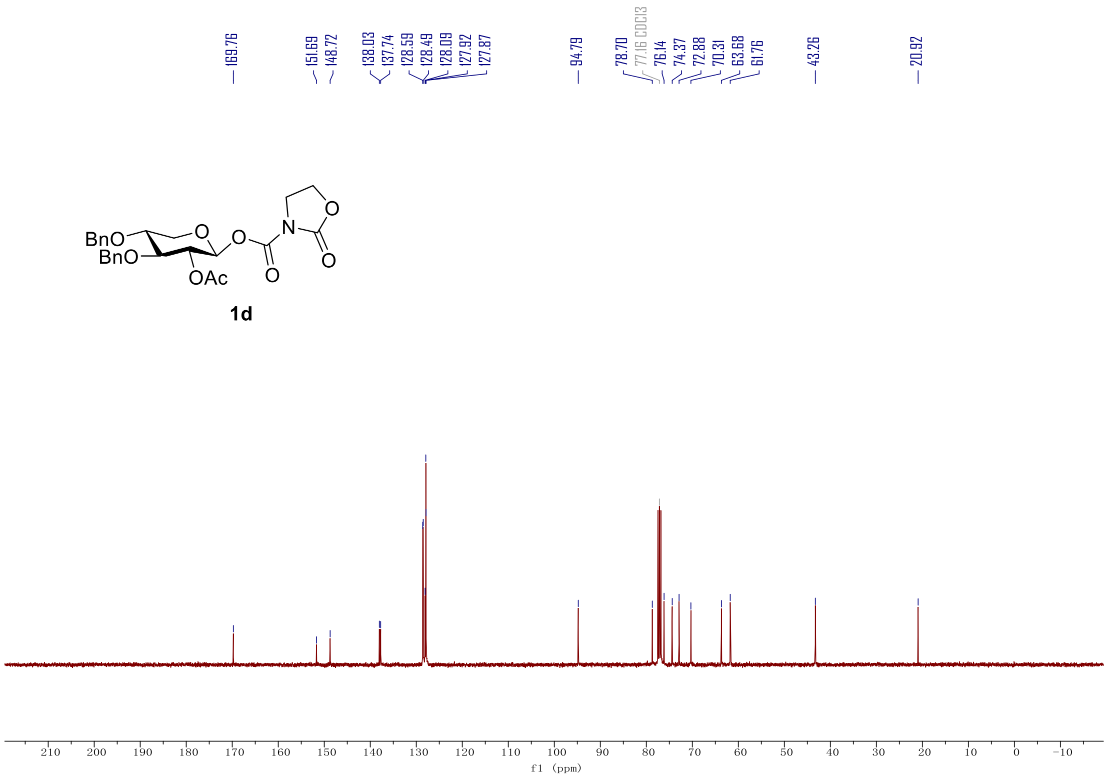


**Supplementary Figure S47. $^{13}C$ NMR (101 MHz, $CDCl_3$) Spectra for compound 1d**

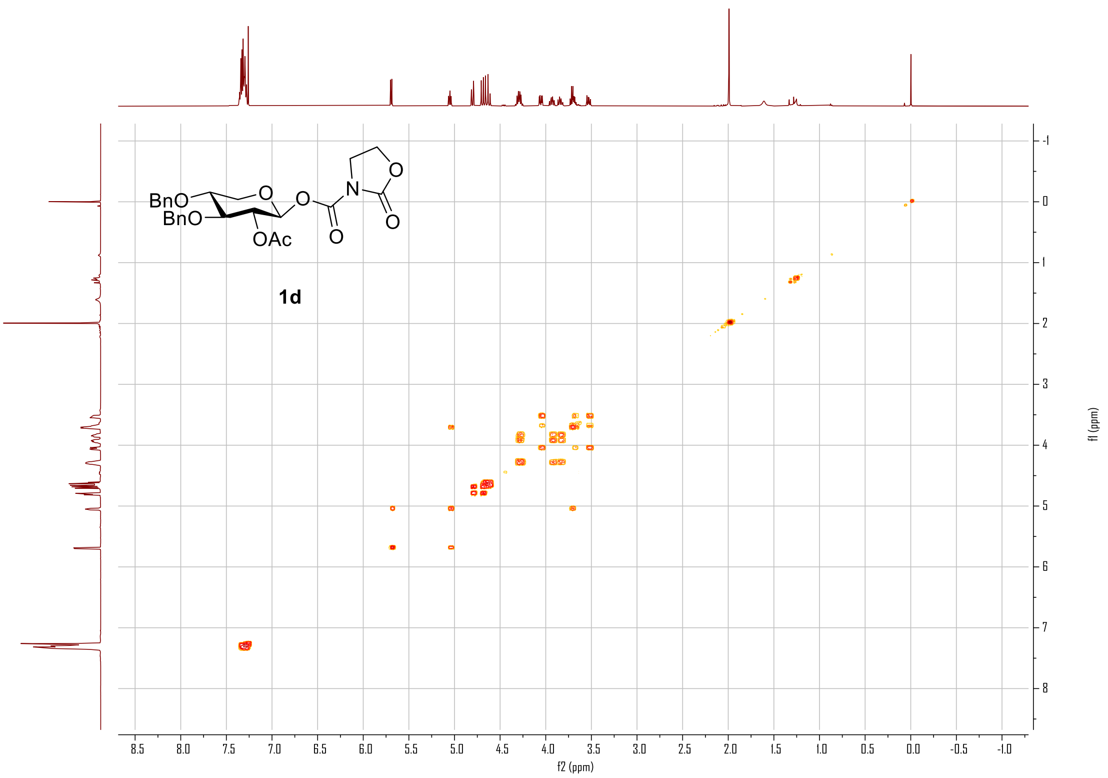


**Supplementary Figure S48. COSY (500 MHz, $CDCl_3$) Spectrum for compound 1d**

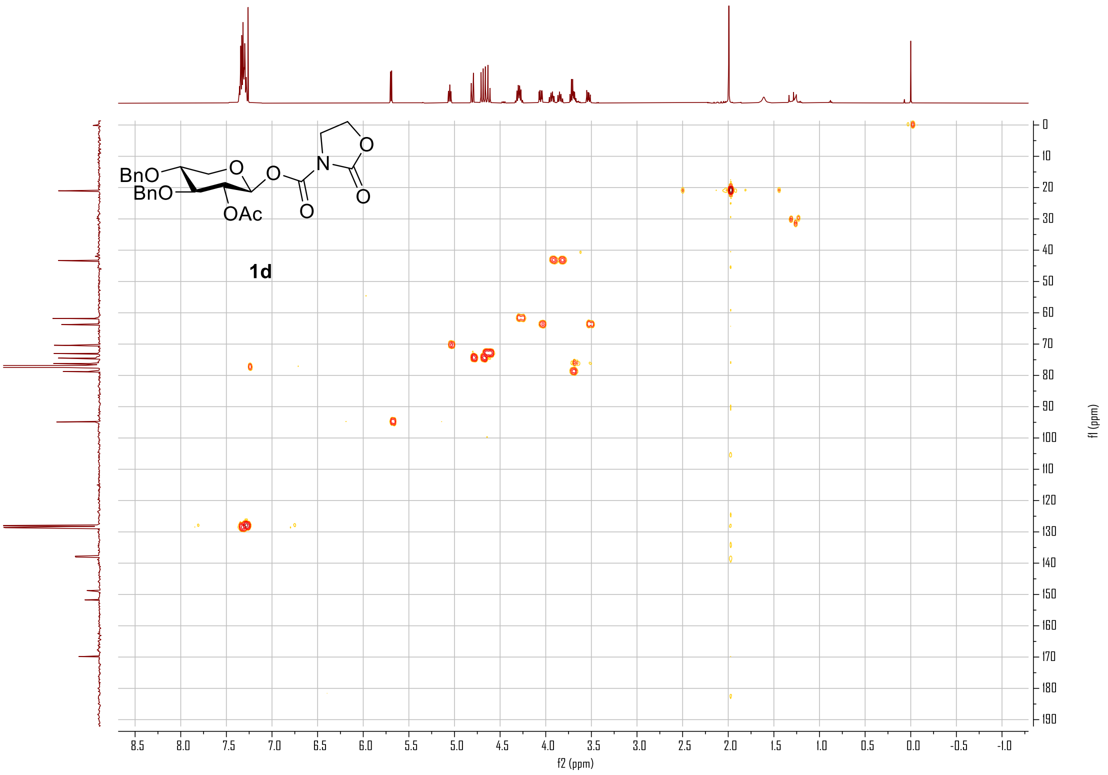


**Supplementary Figure S49. HMQC (500 MHz, $CDCl_3$) Spectrum for compound 1d**

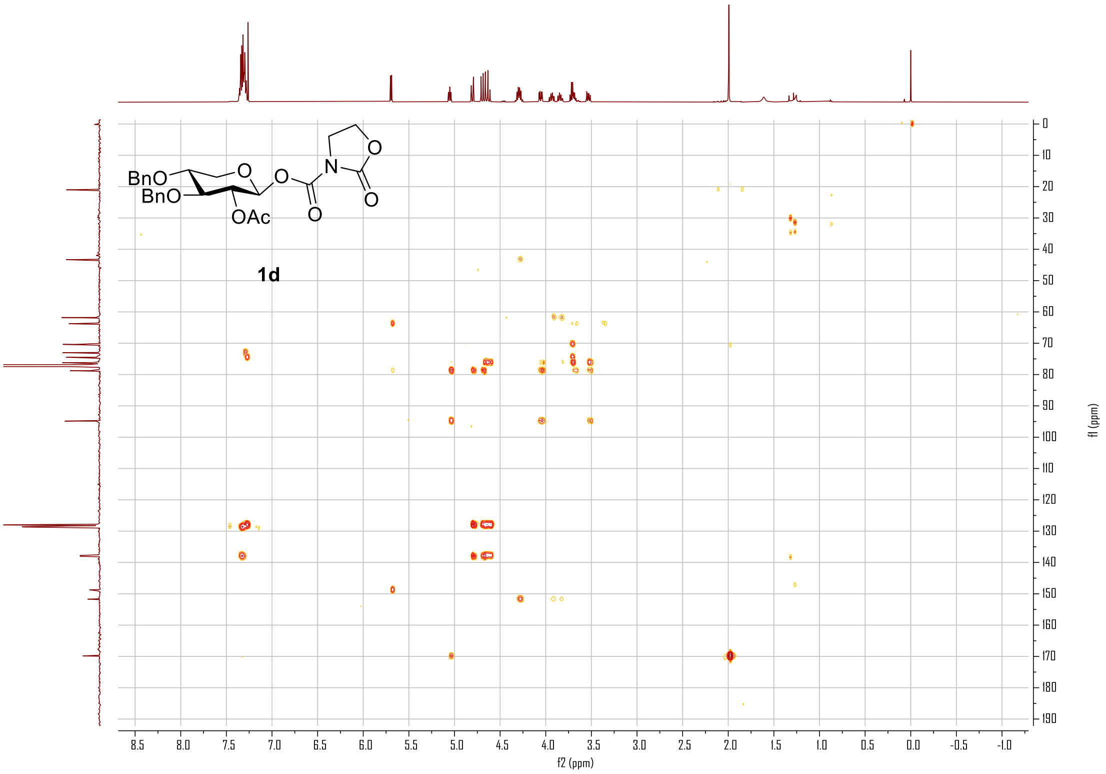


**Supplementary Figure S50. HMBC (500 MHz, $CDCl_3$) Spectrum for compound 1d**

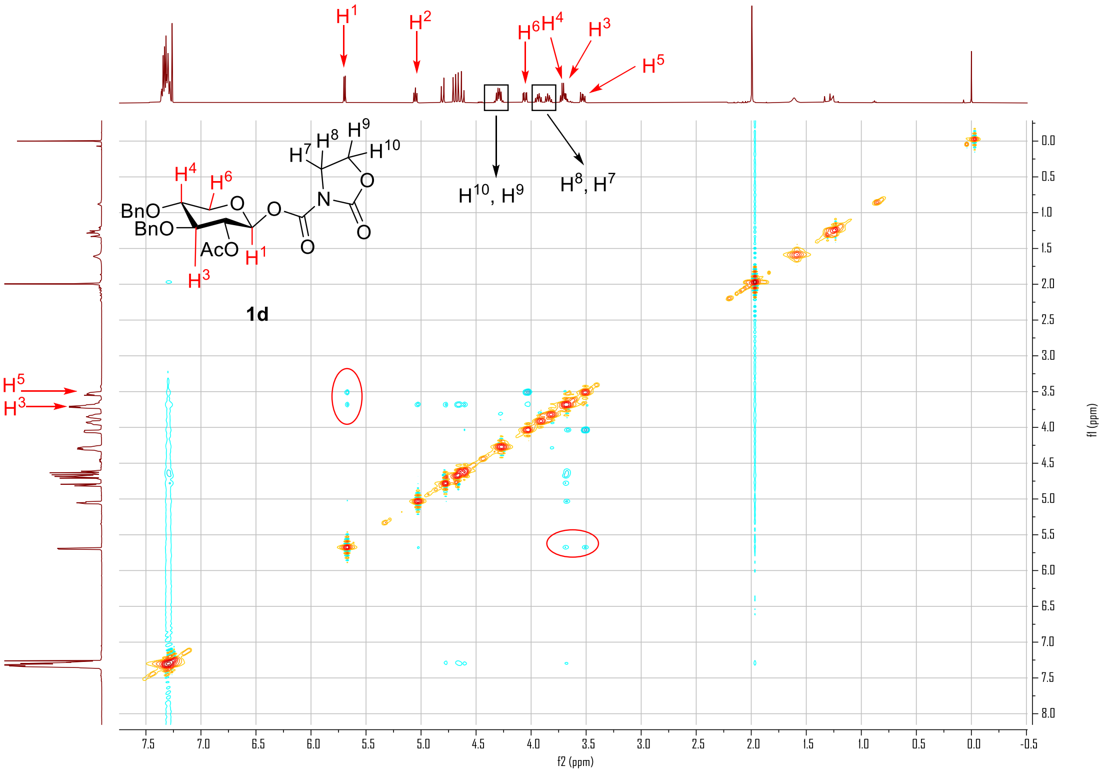


**Supplementary Figure S51. NOESY (500 MHz, $CDCl_3$) Spectrum for compound 1d**

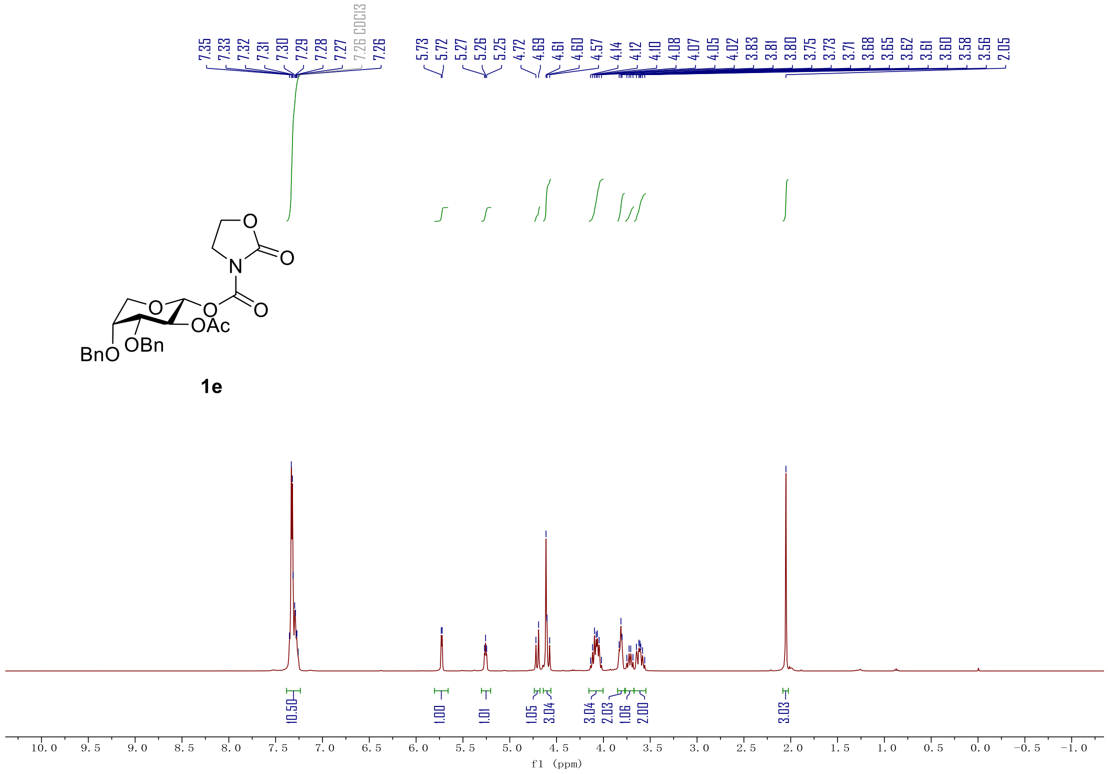


**Supplementary Figure S52. $^{1}H$ NMR (400 MHz, $CDCl_3$) Spectra for compound 1e**

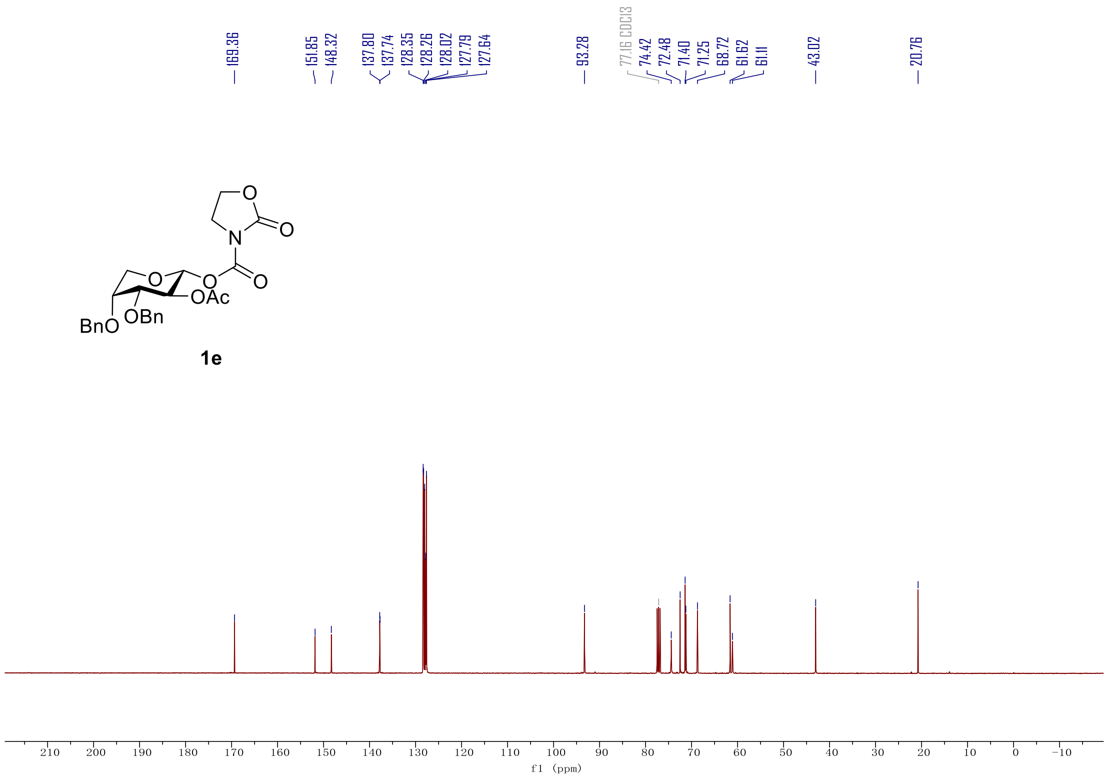


**Supplementary Figure S53. $^{13}C$ NMR (101 MHz, $CDCl_3$) Spectra for compound 1e**

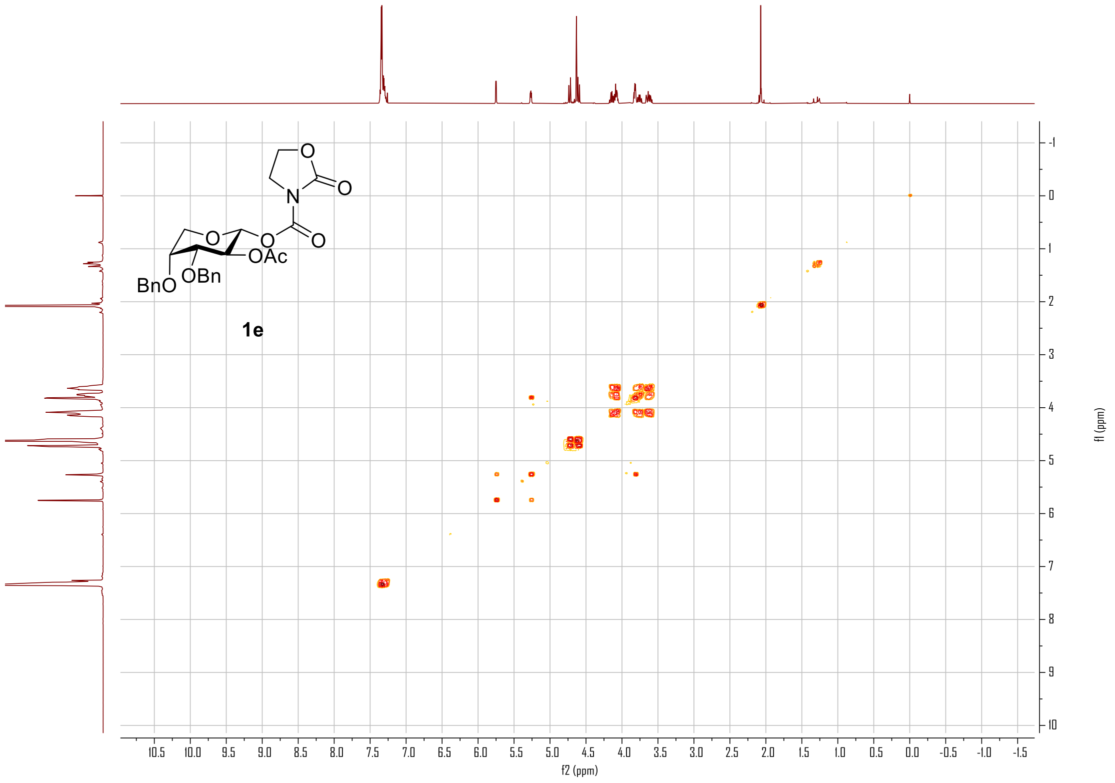


**Supplementary Figure S54. COSY (500 MHz, CDCl₃) Spectrum for compound 1e**

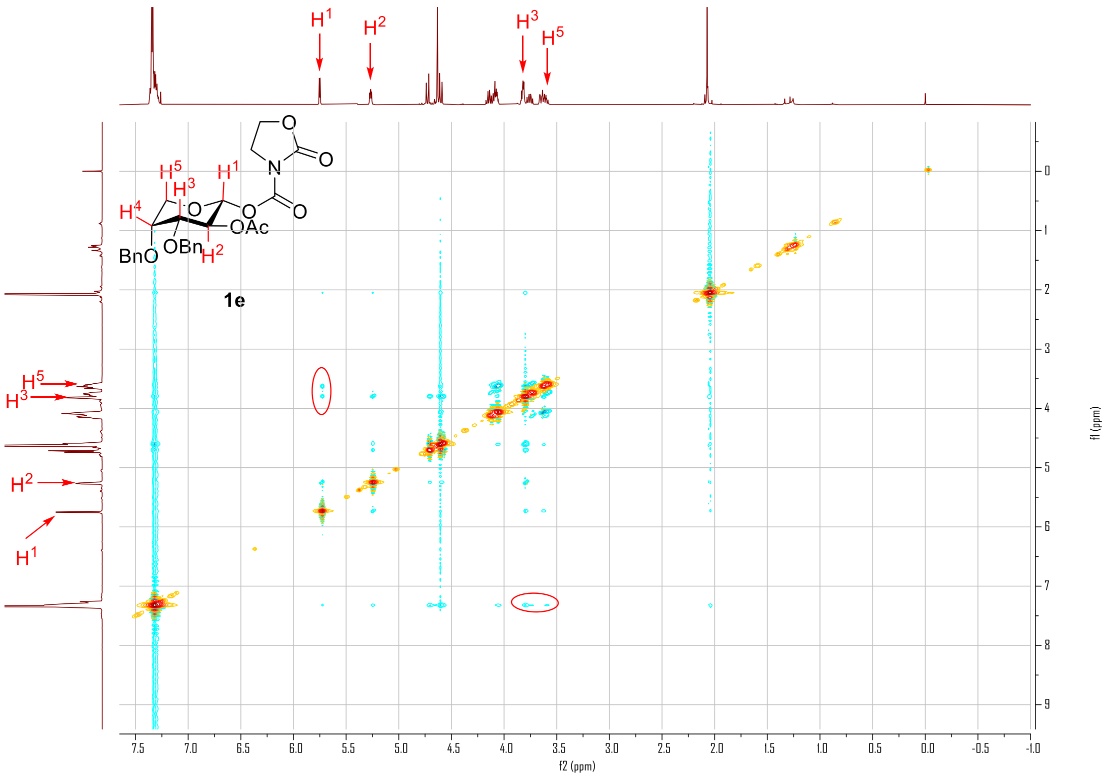


**Supplementary Figure S55. NOESY (500 MHz, CDCl₃) Spectrum for compound 1e**

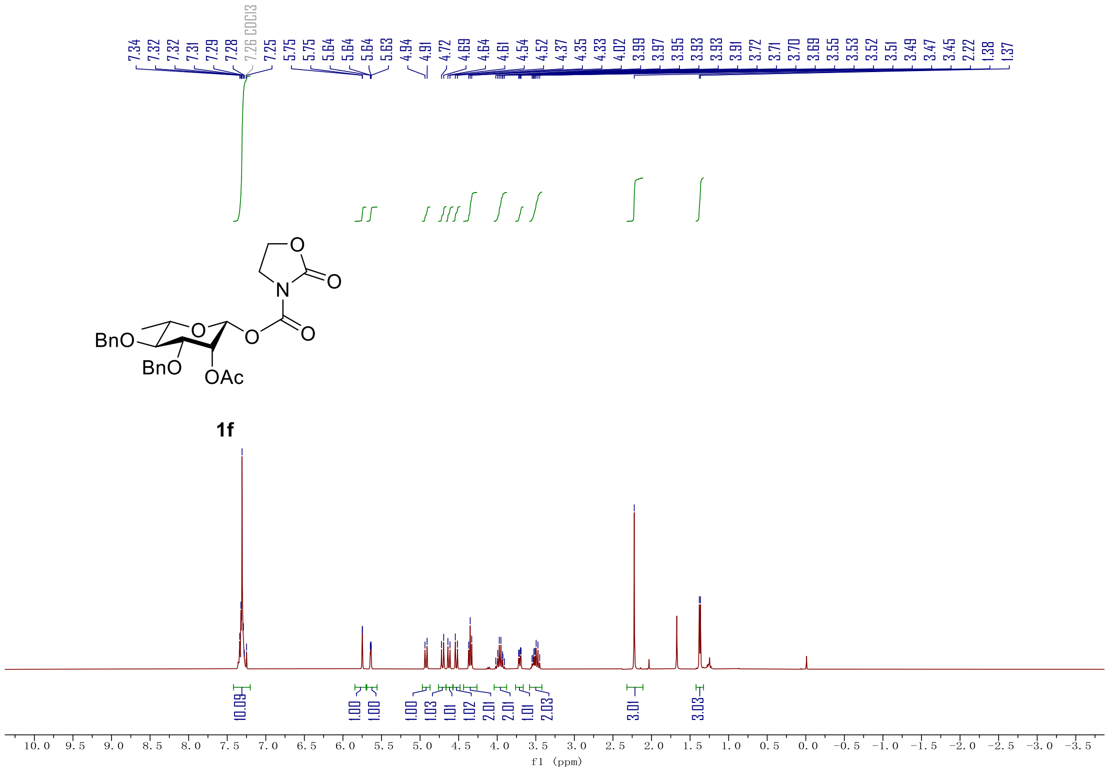

**Supplementary Figure S56. $^{1}$H NMR (400 MHz, CDCl$_3$) Spectra for compound 1f**

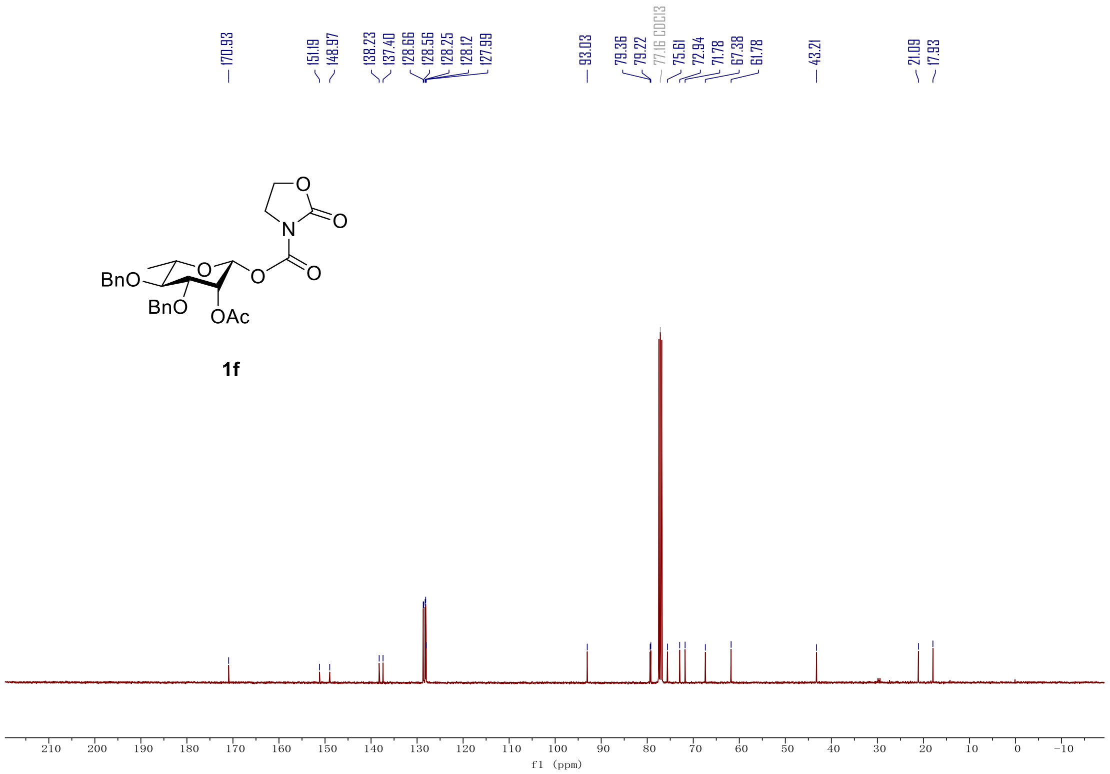

**Supplementary Figure S57. $^{13}$C NMR (101 MHz, CDCl$_3$) Spectra for compound 1f**

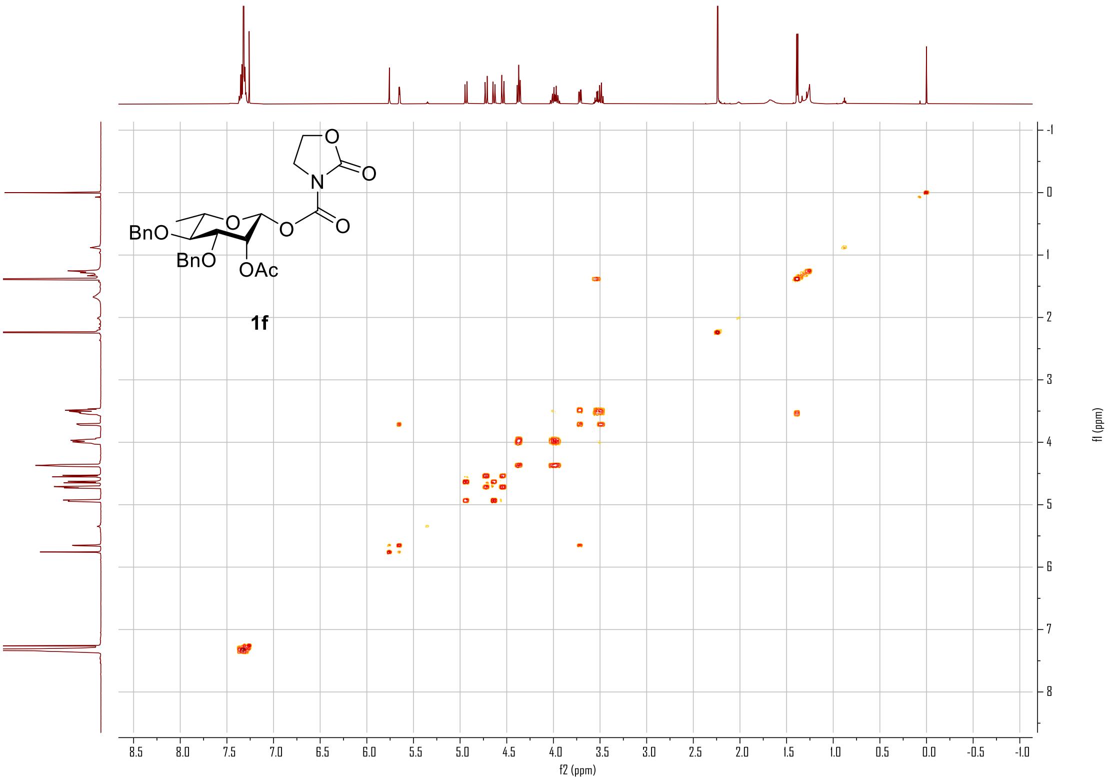


**Supplementary Figure S58. COSY (500 MHz, $CDCl_3$) Spectrum for compound 1f**

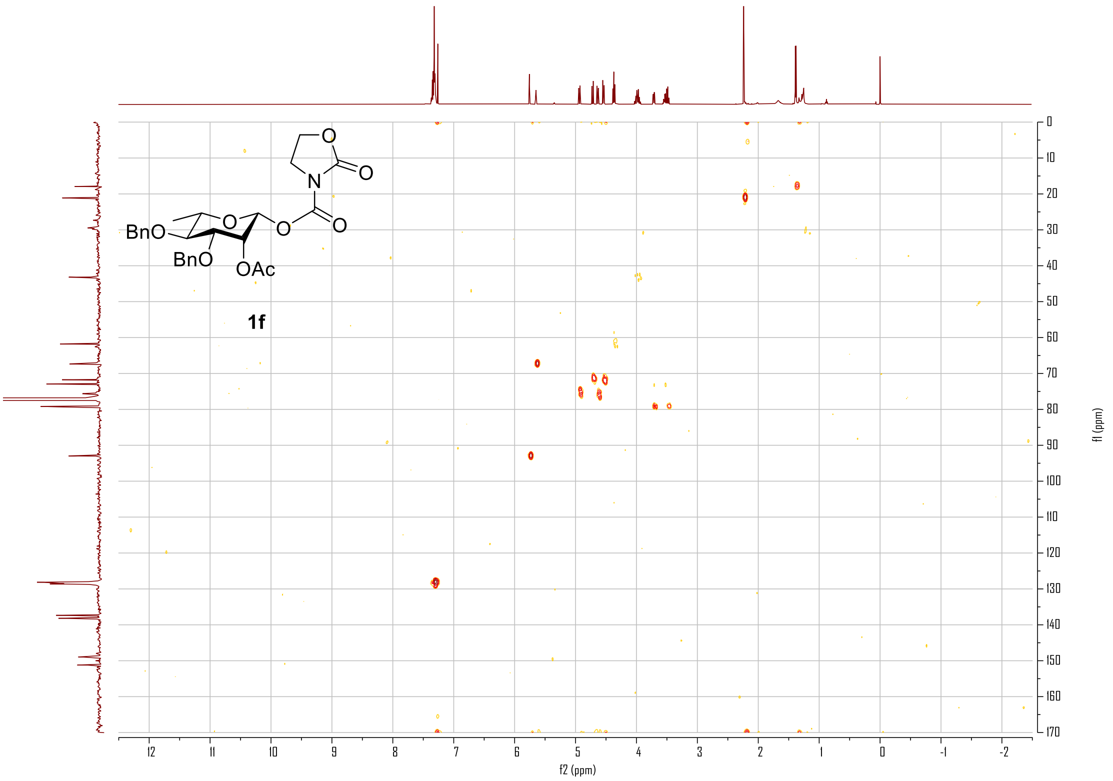


**Supplementary Figure S59. HMQC (500 MHz, $CDCl_3$) Spectrum for compound 1f**

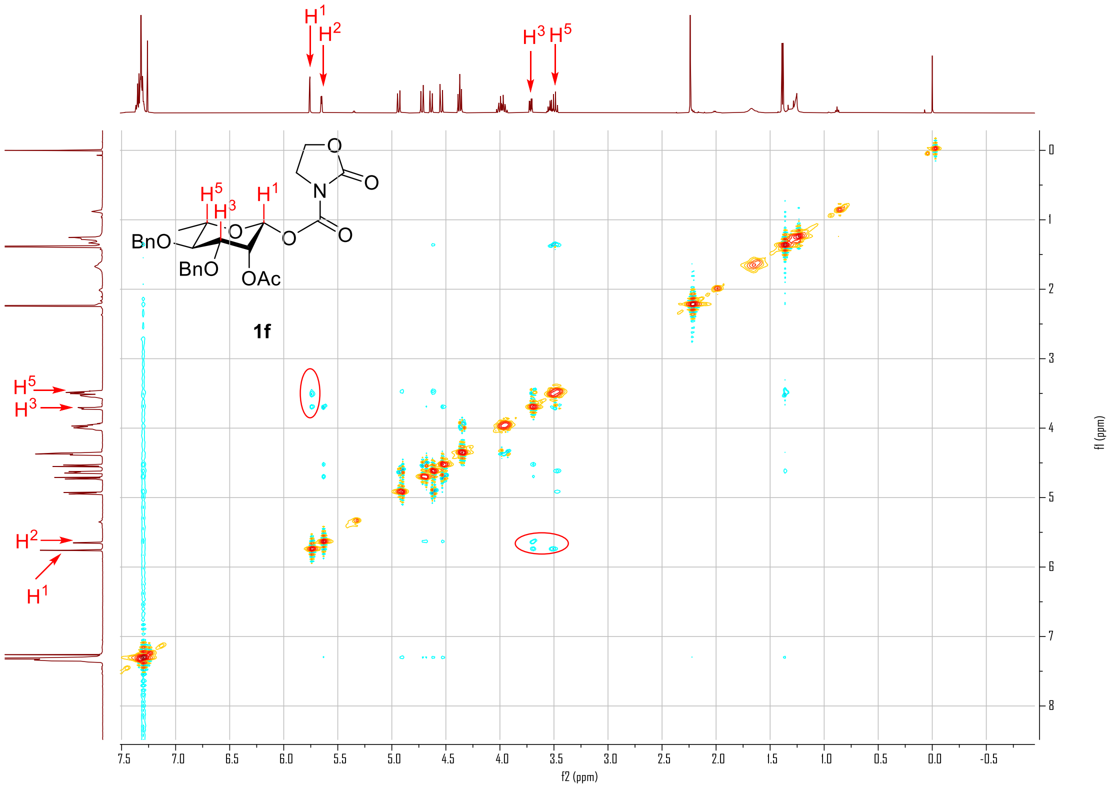


**Supplementary Figure S60. NOESY (500 MHz, CDCl₃) Spectrum for compound 1f**

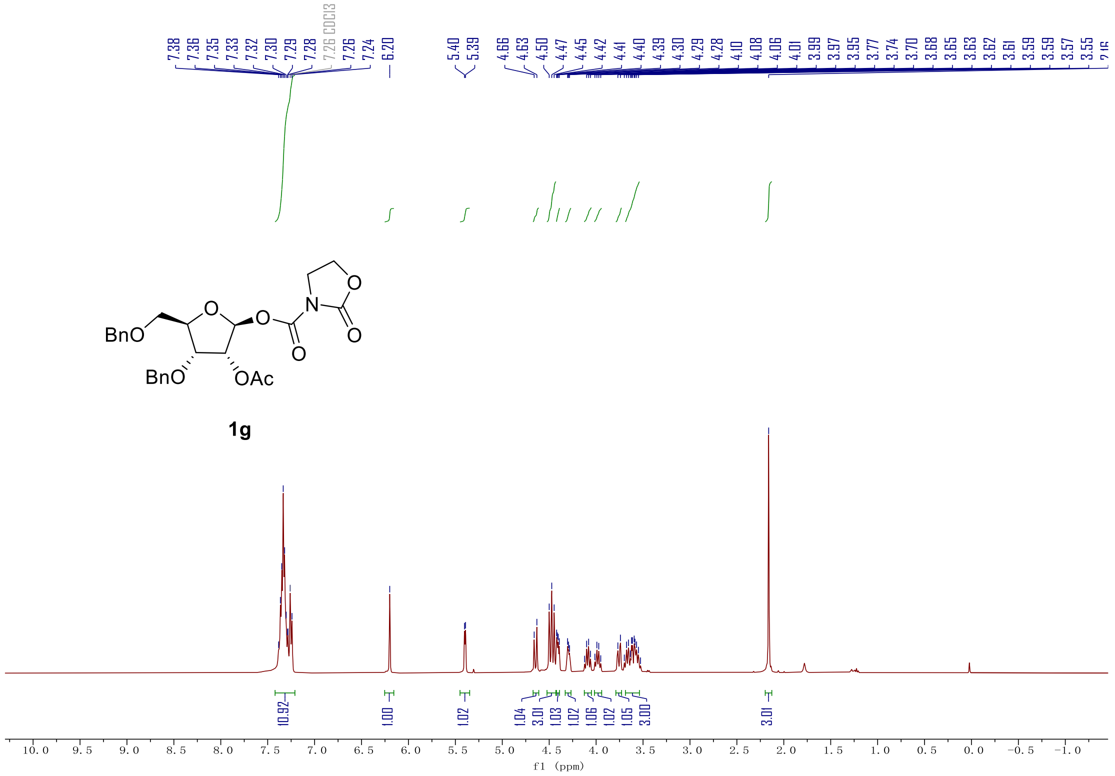


**Supplementary Figure S61. $^1$H NMR (400 MHz, CDCl₃) Spectra for compound 1g**

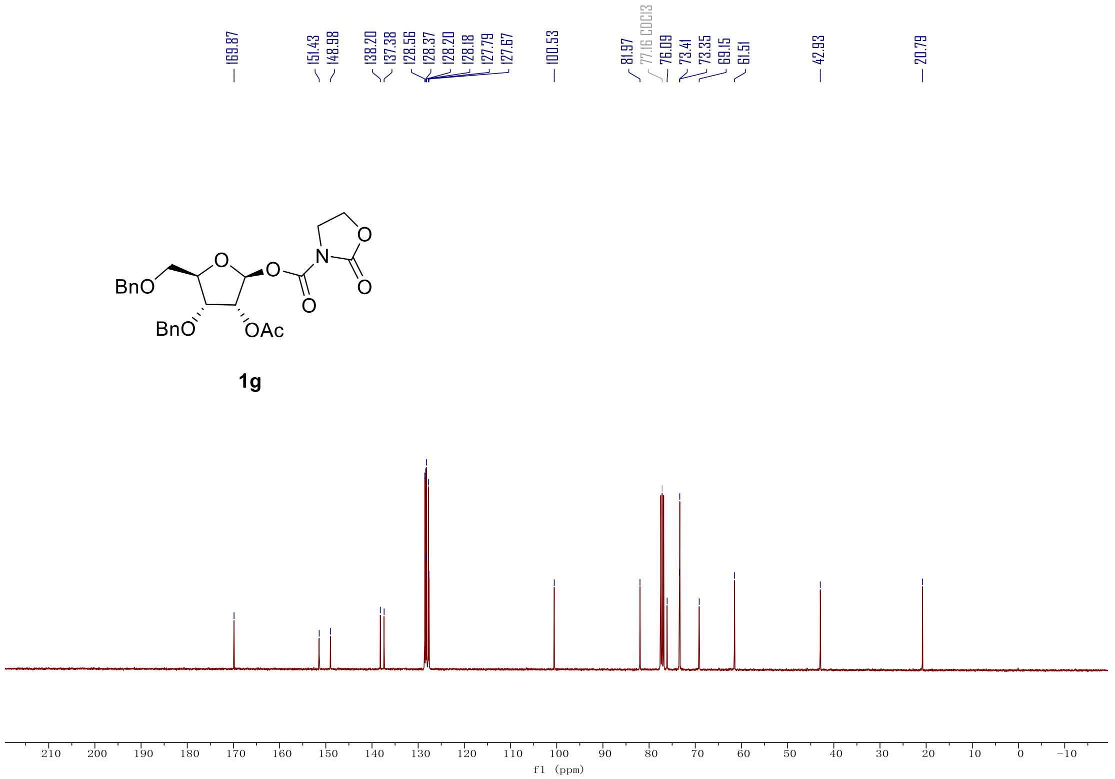


**Supplementary Figure S62.** $^{13}C$ **NMR (101 MHz,** $CDCl_3$**) Spectra for compound 1g**

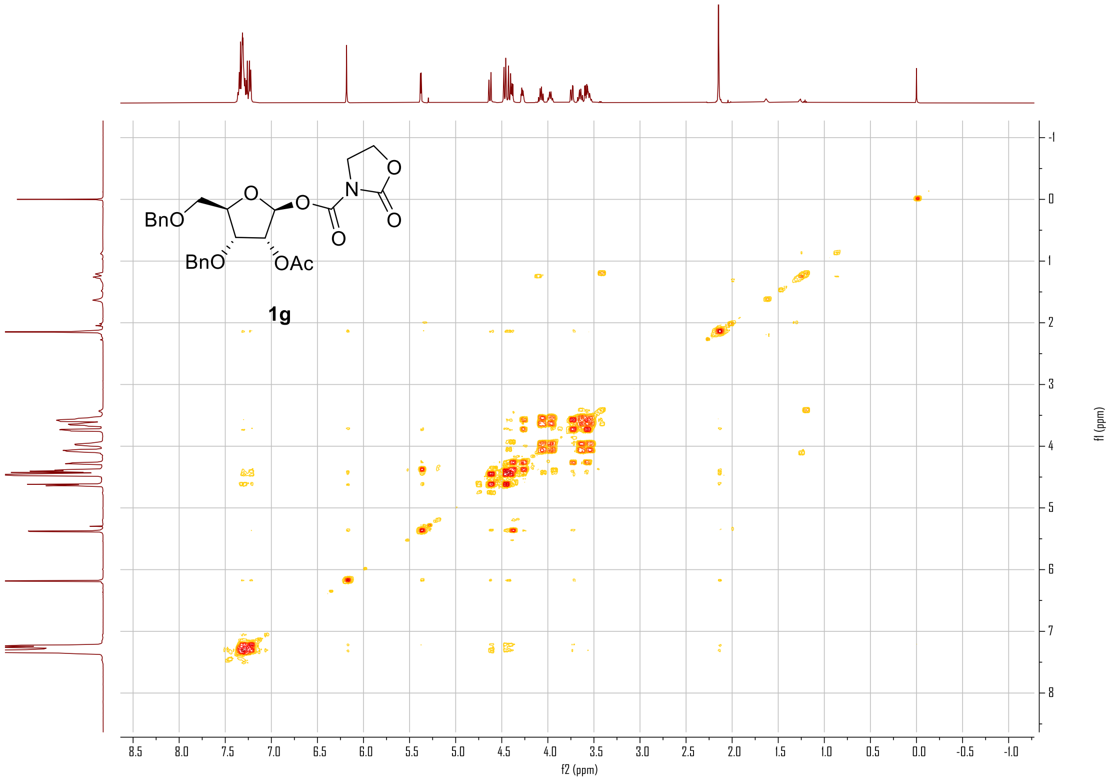


**Supplementary Figure S63. COSY (500 MHz,** $CDCl_3$**) Spectrum for compound 1g**

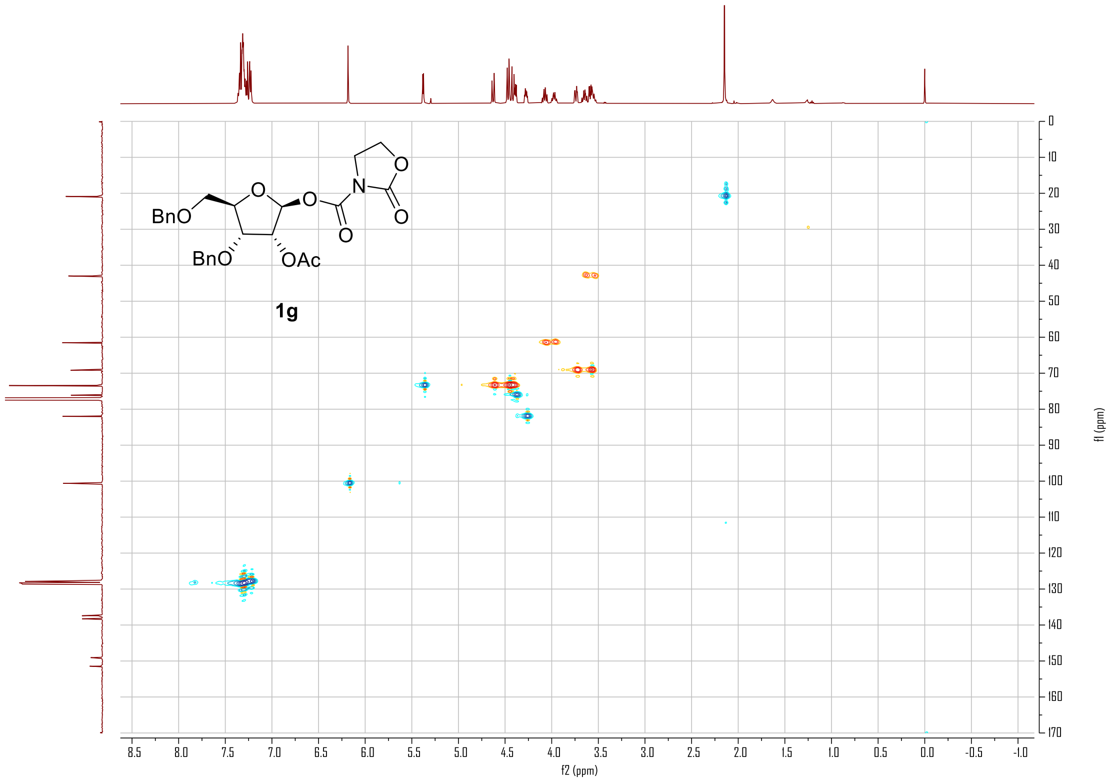


**Supplementary Figure S64. HSQC (500 MHz, $CDCl_3$) Spectrum for compound 1g**

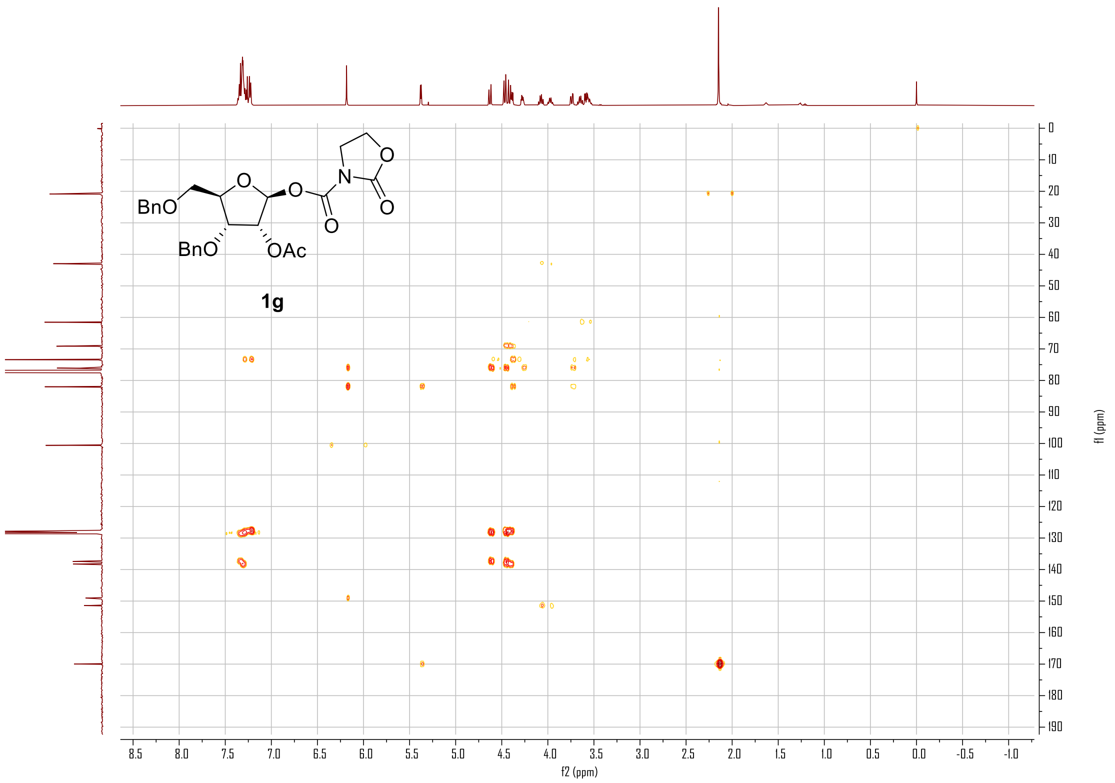


**Supplementary Figure S65. HMBC (500 MHz, $CDCl_3$) Spectrum for compound 1g**

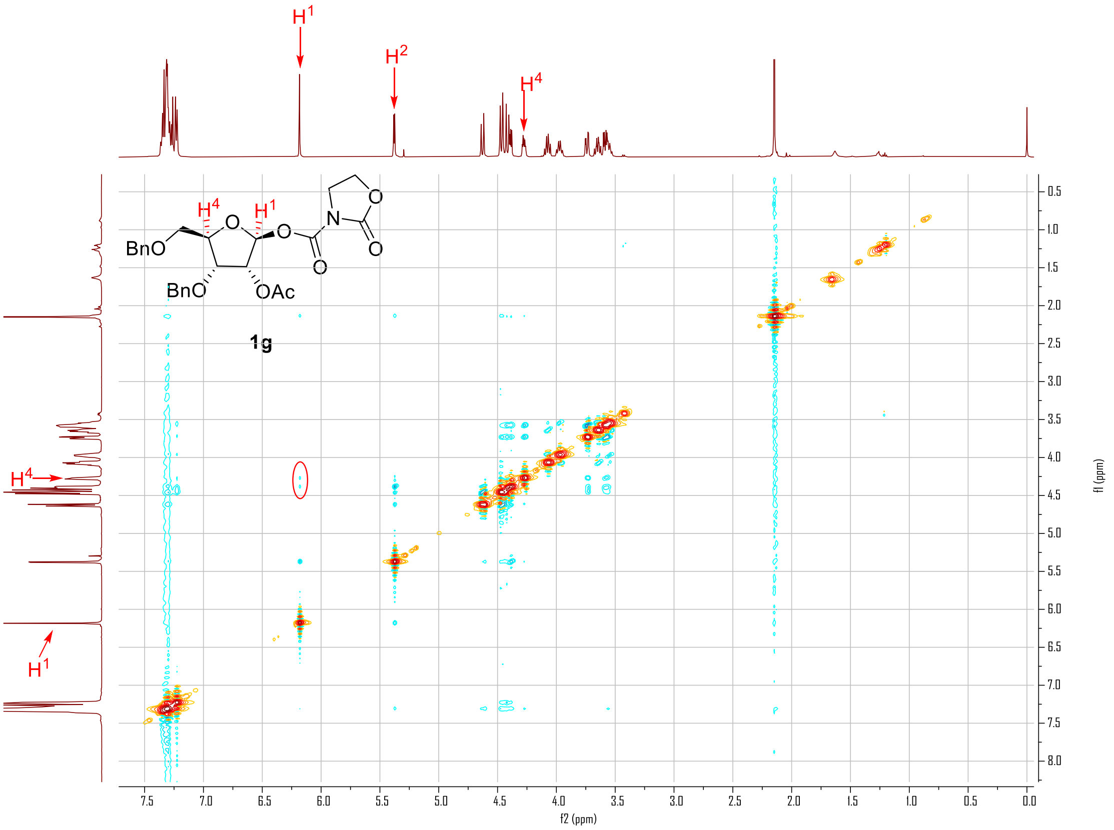


**Supplementary Figure S66. NOESY (500 MHz, $CDCl_3$) Spectrum for compound 1g**

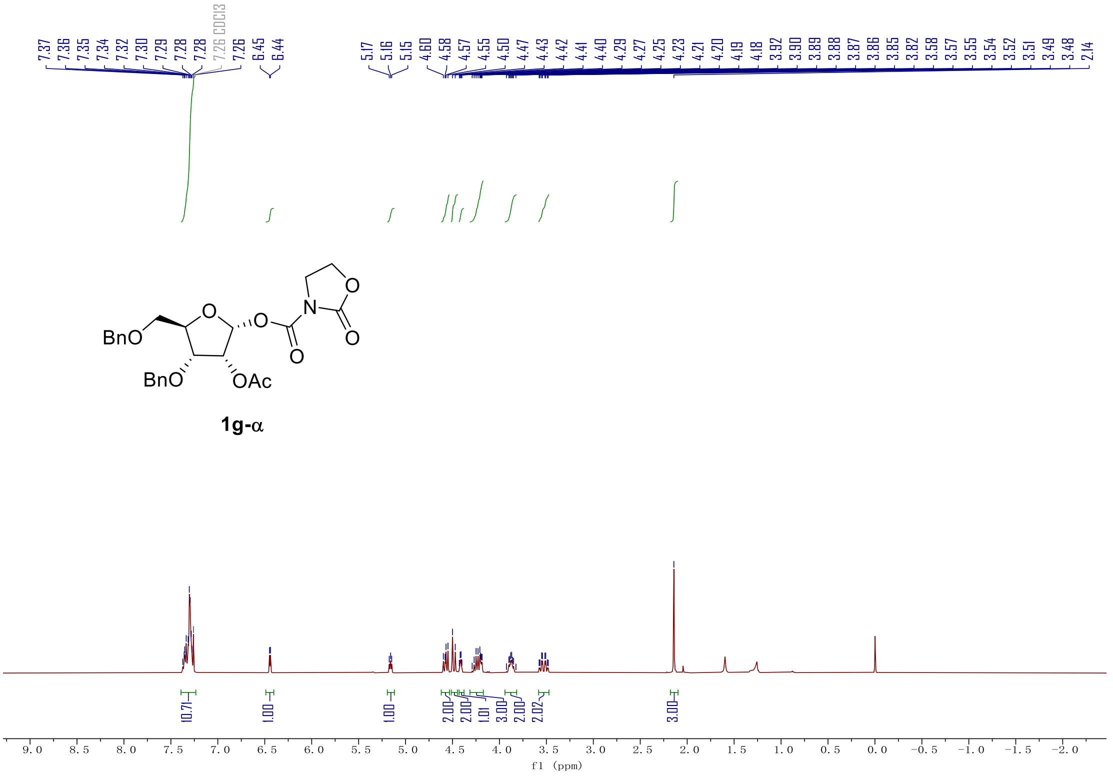


**Supplementary Figure S67. $^1$H NMR (400 MHz, $CDCl_3$) Spectra for compound 1g-α**

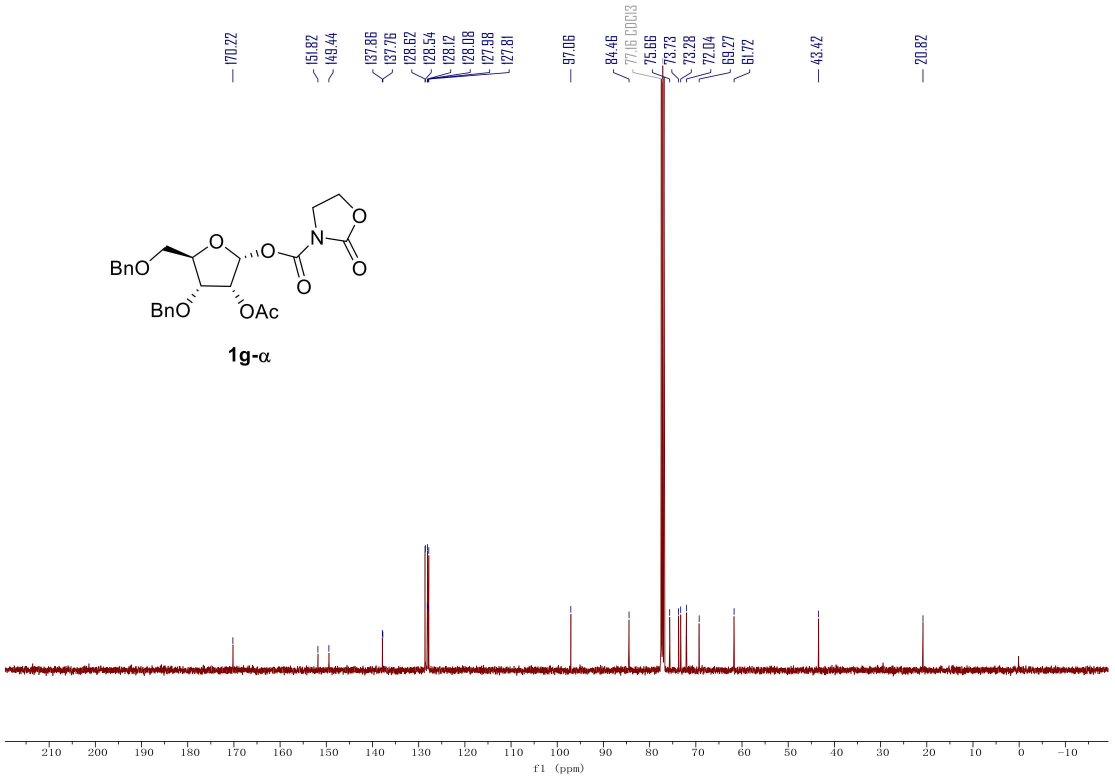

Supplementary Figure S68. $^{13}C$ NMR (101 MHz, $CDCl_3$) Spectra for compound 1g-α

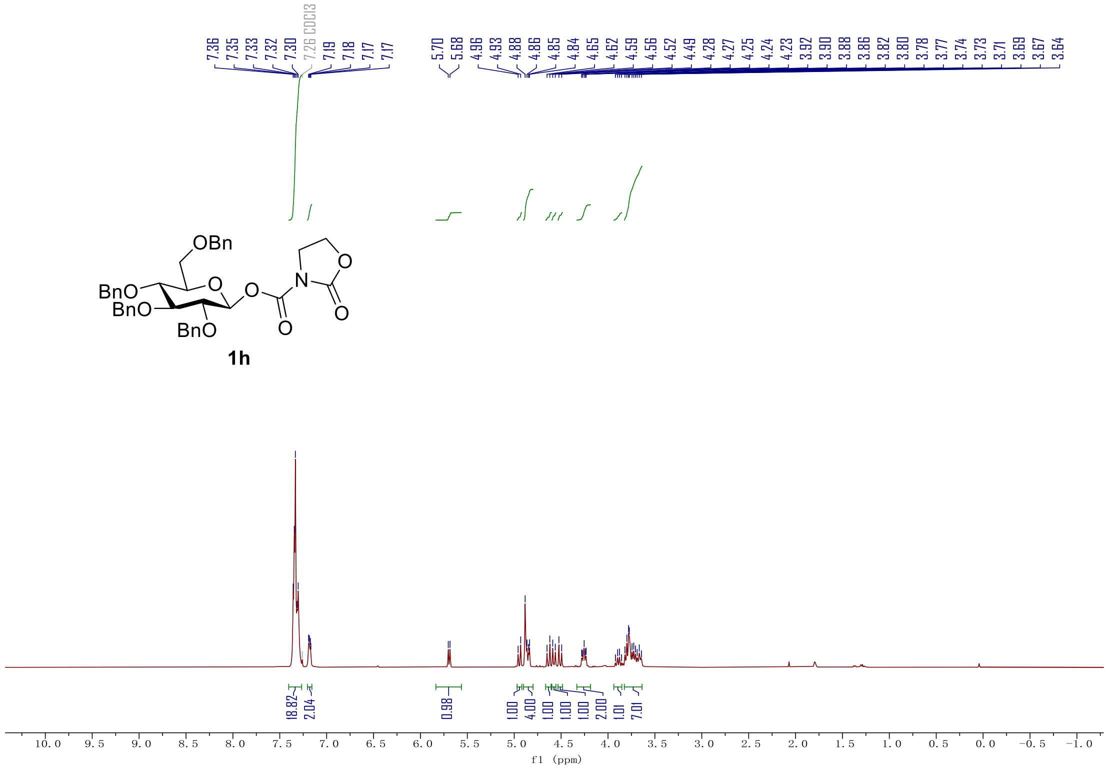

Supplementary Figure S69. $^{1}H$ NMR (400 MHz, $CDCl_3$) Spectra for compound 1h

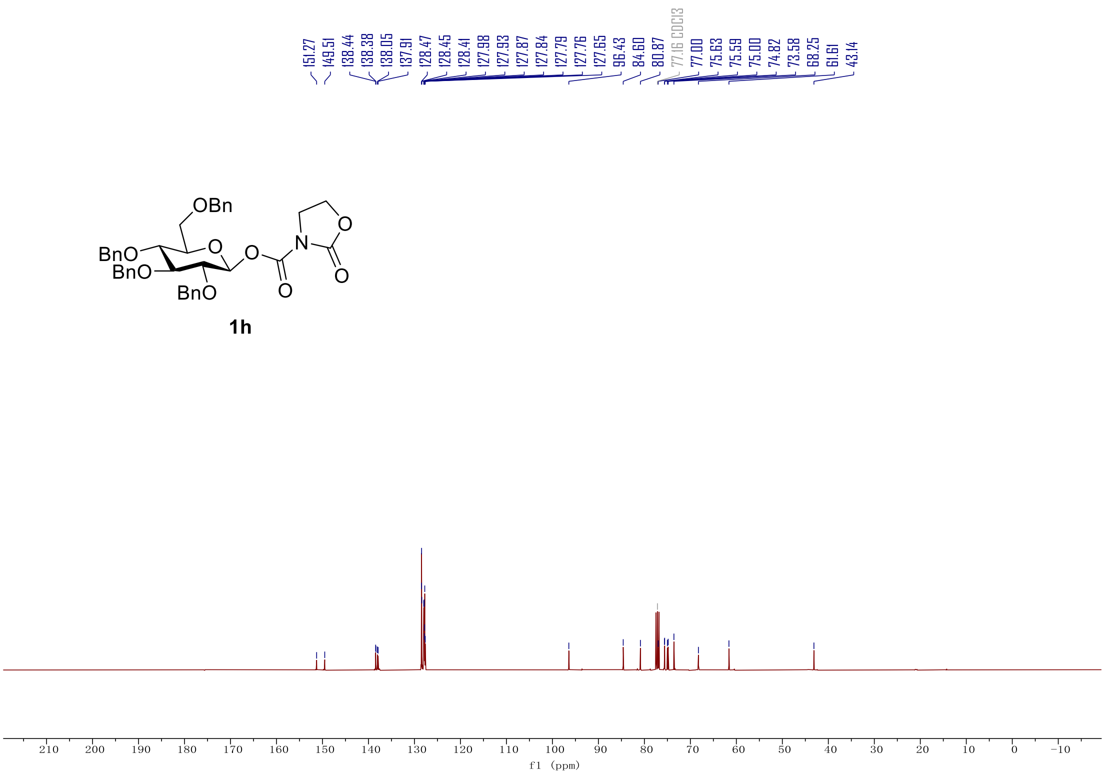


**Supplementary Figure S70. $^{13}$C NMR (101 MHz, $CDCl_3$) Spectra for compound 1h**

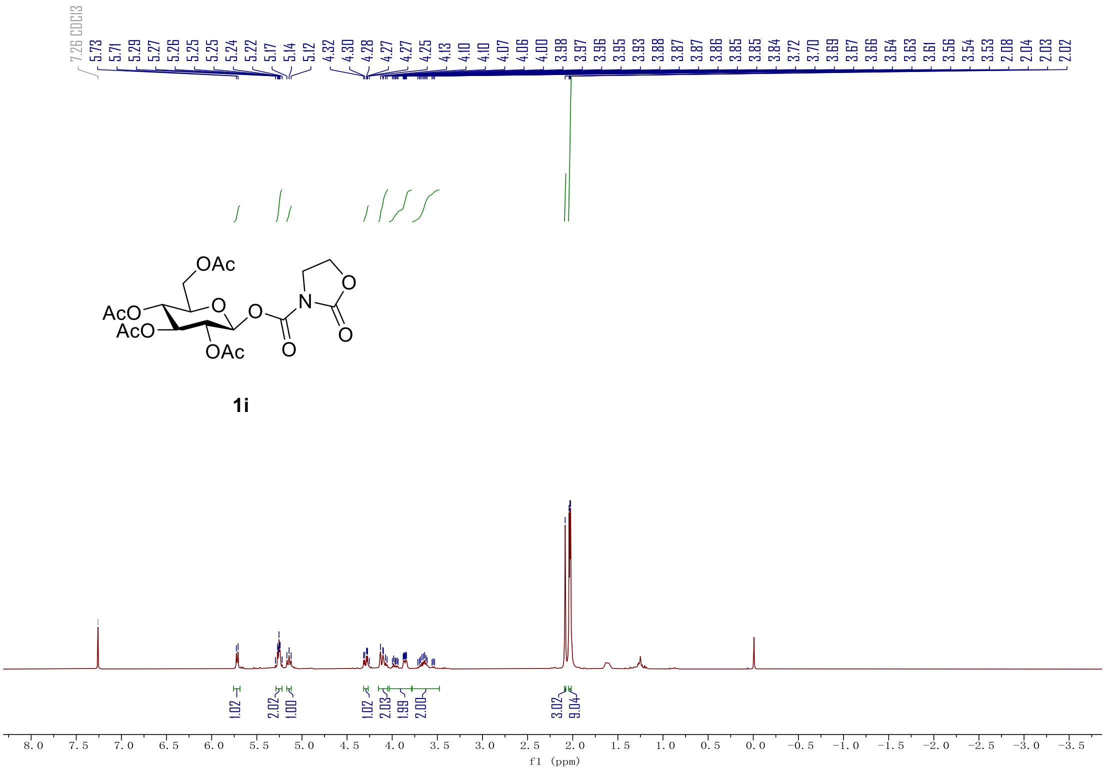


**Supplementary Figure S71. $^{1}$H NMR (400 MHz, $CDCl_3$) Spectra for compound 1i**

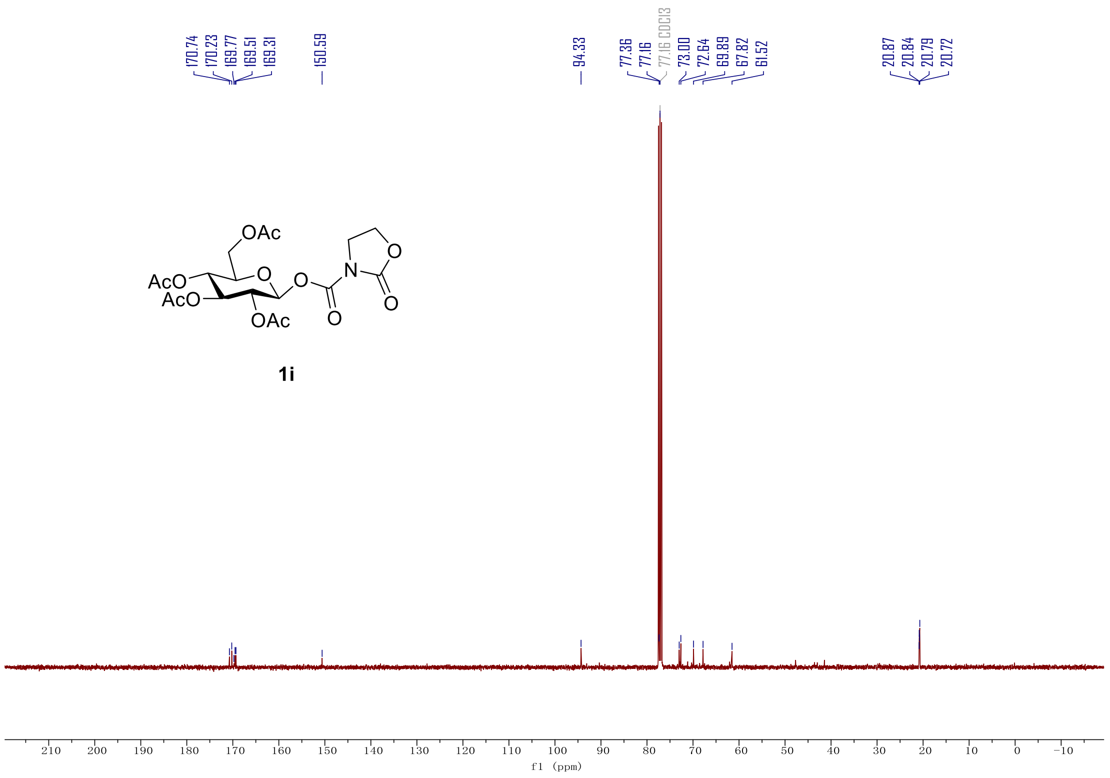


1i

Supplementary Figure S72. $^{13}C$ NMR (101 MHz, $CDCl_3$) Spectra for compound 1i

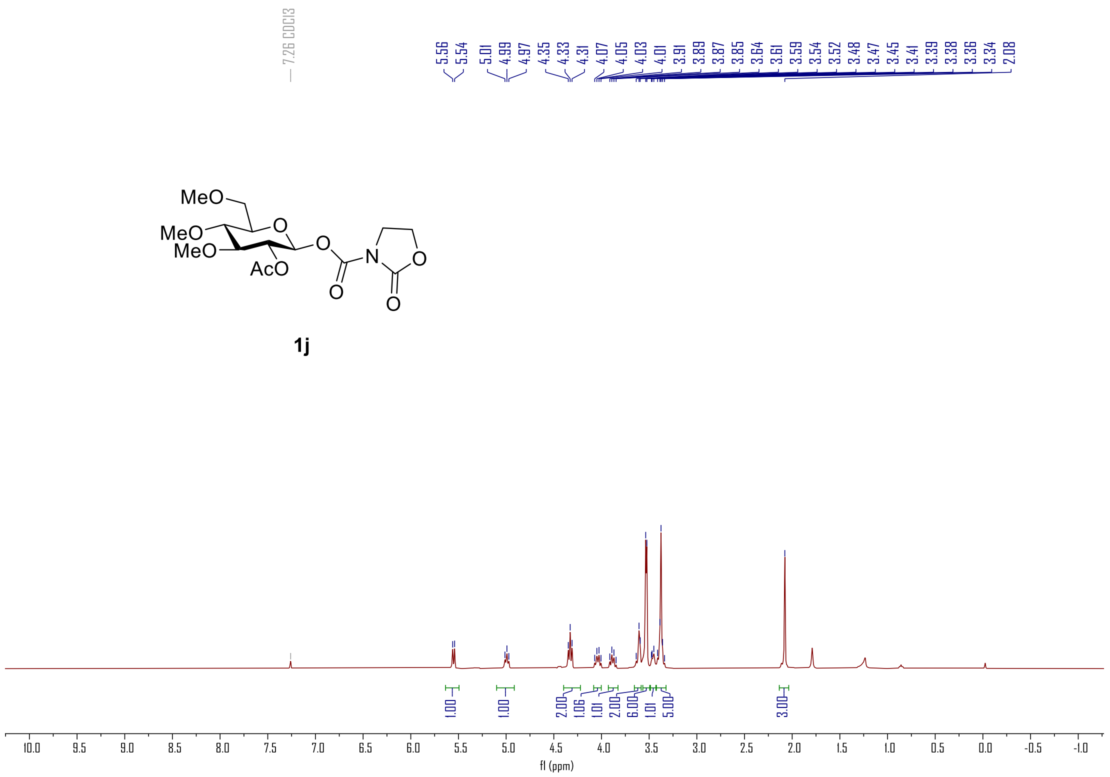


1j

Supplementary Figure S73. $^{1}H$ NMR (400 MHz, $CDCl_3$) Spectra for compound 1j

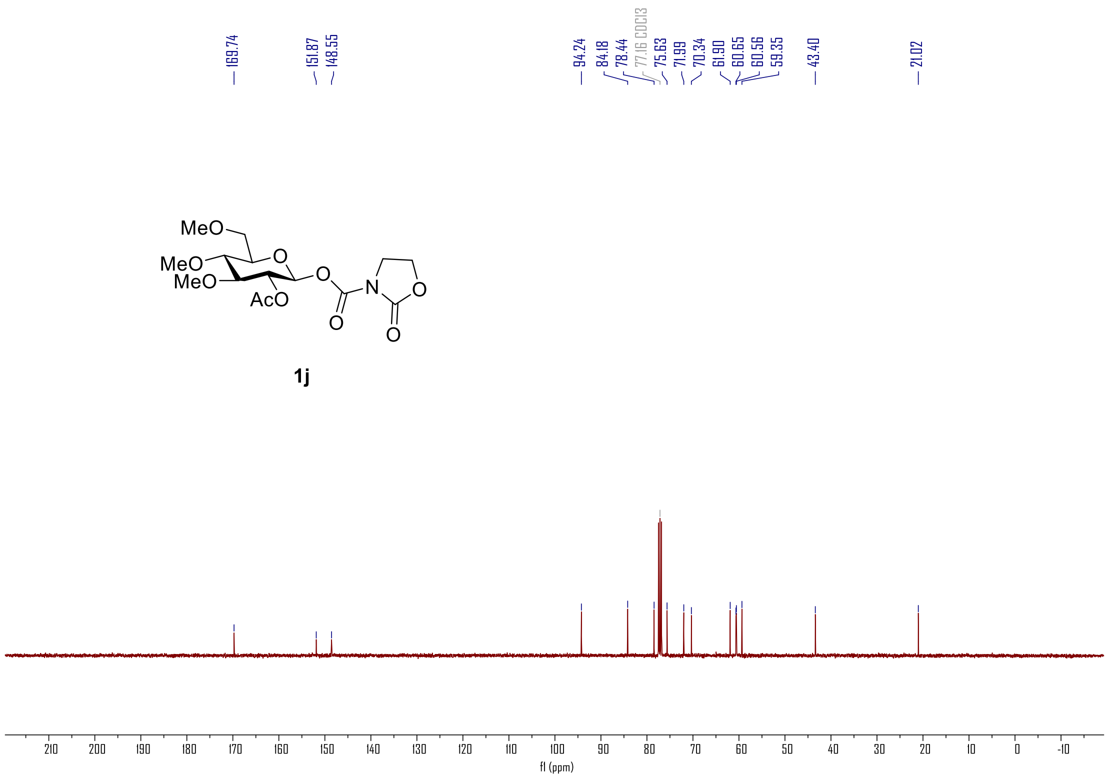

Supplementary Figure S74. $^{13}C$ NMR (101 MHz, $CDCl_3$) Spectra for compound 1j

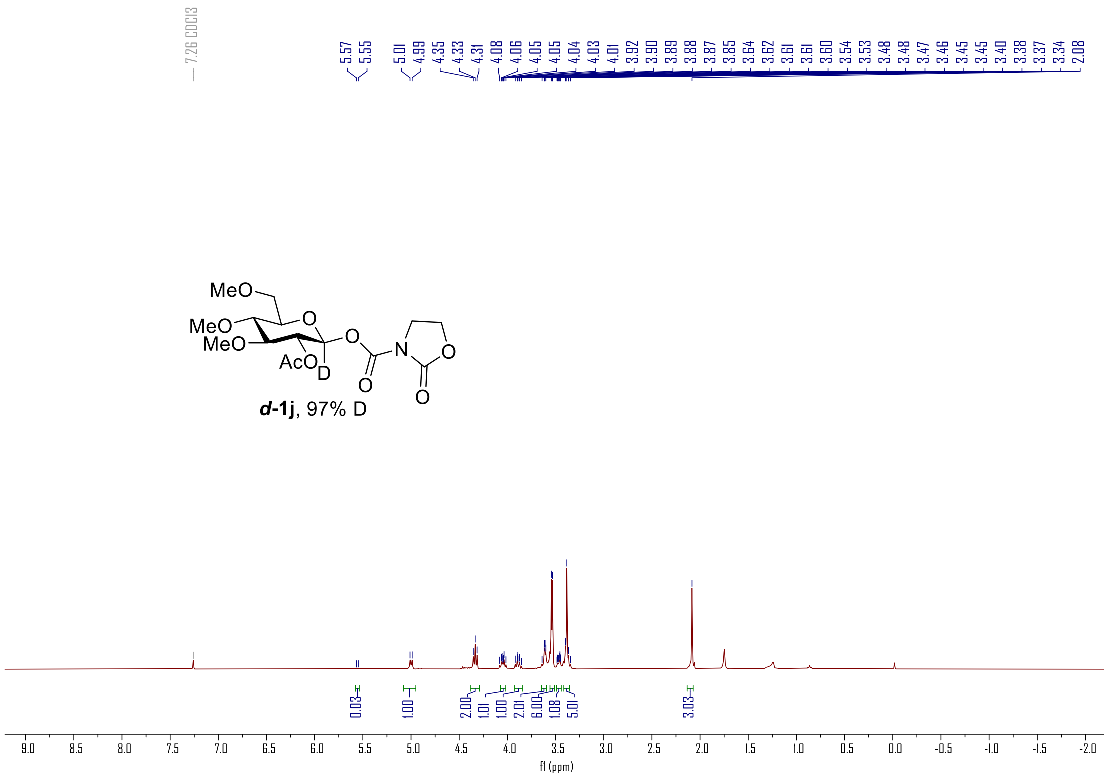

Supplementary Figure S75. $^{1}H$ NMR (400 MHz, $CDCl_3$) Spectra for compound *d*-1j

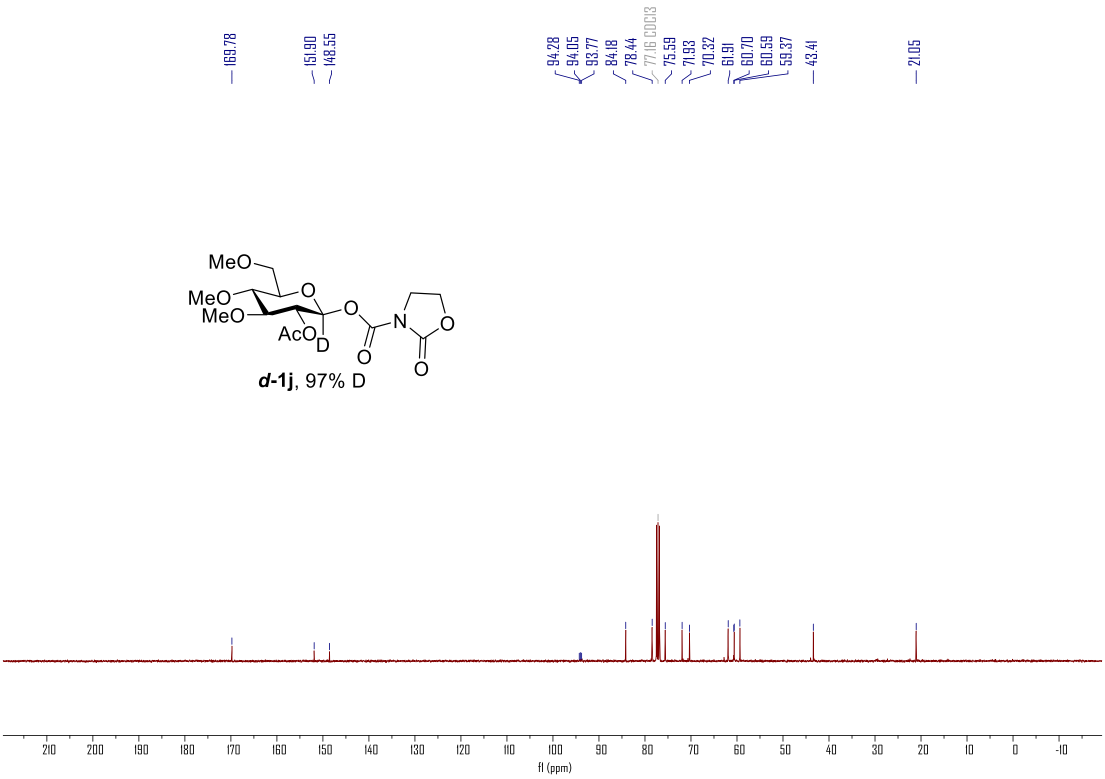


Supplementary Figure S76. $^{13}$C NMR (101 MHz, $CDCl_3$) Spectra for compound *d*-1j

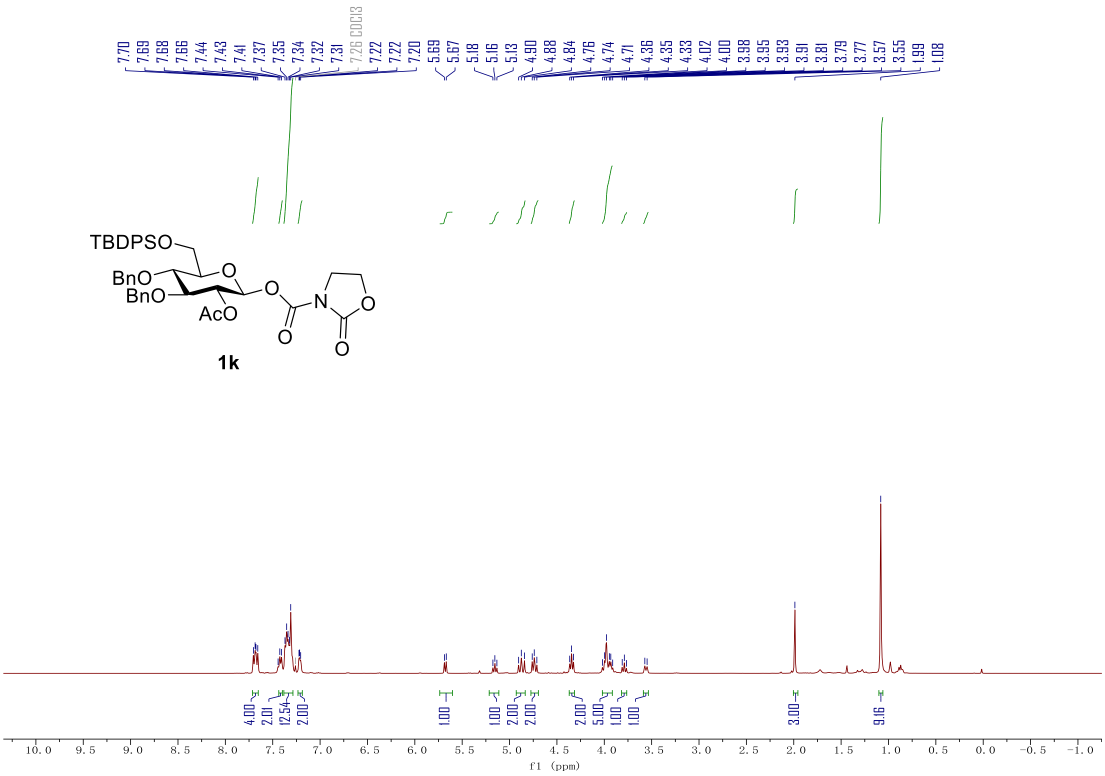


Supplementary Figure S77. $^{1}$H NMR (400 MHz, $CDCl_3$) Spectra for compound 1k

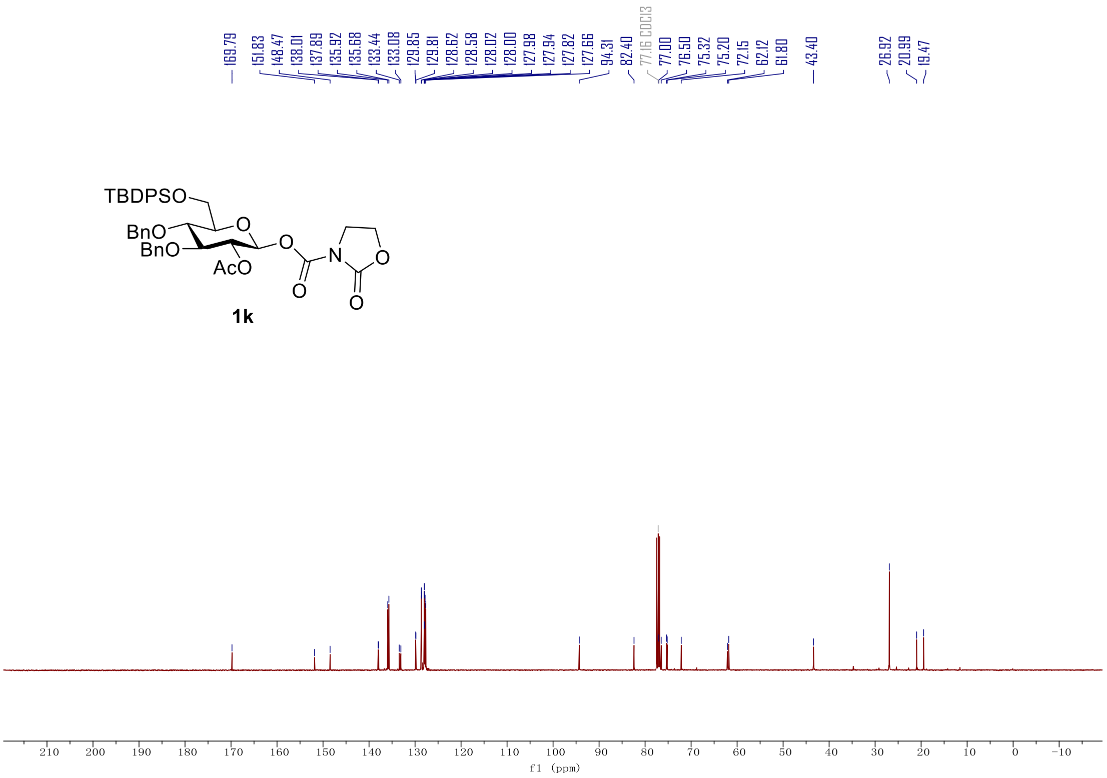


**Supplementary Figure S78. $^{13}$C NMR (101 MHz, $CDCl_3$) Spectra for compound 1k**

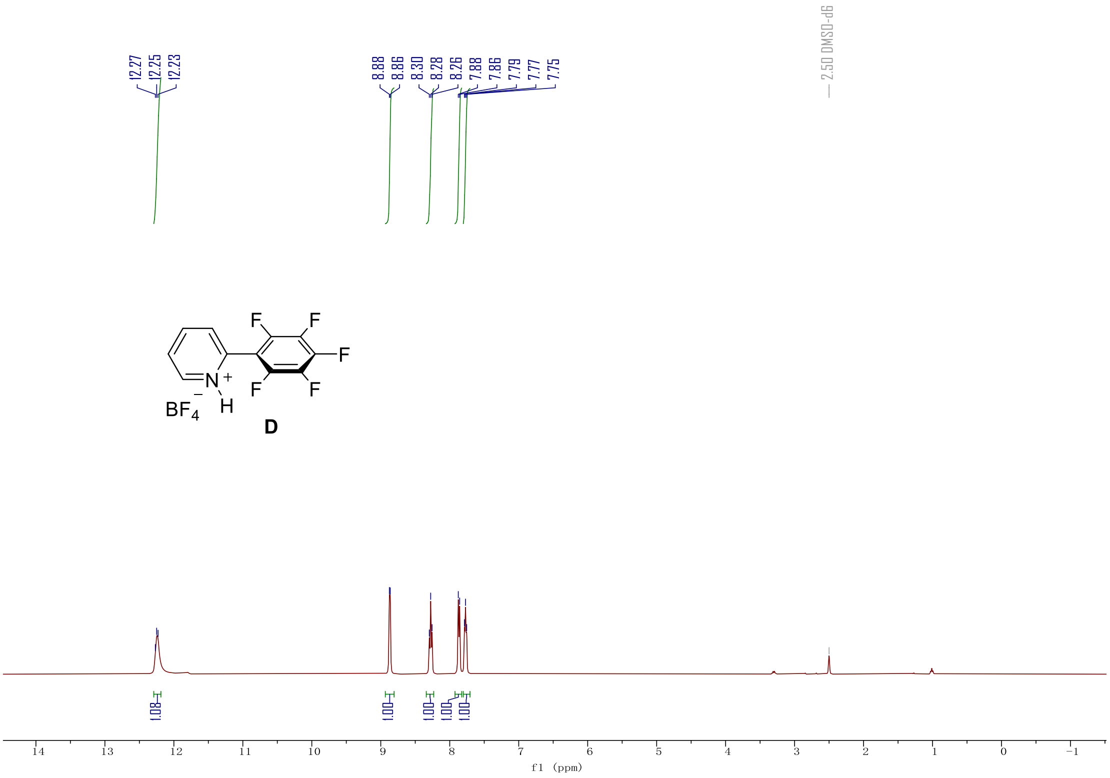


**Supplementary Figure S79. $^{1}$H NMR (400 MHz, DMSO-$d_6$) Spectra for compound D**

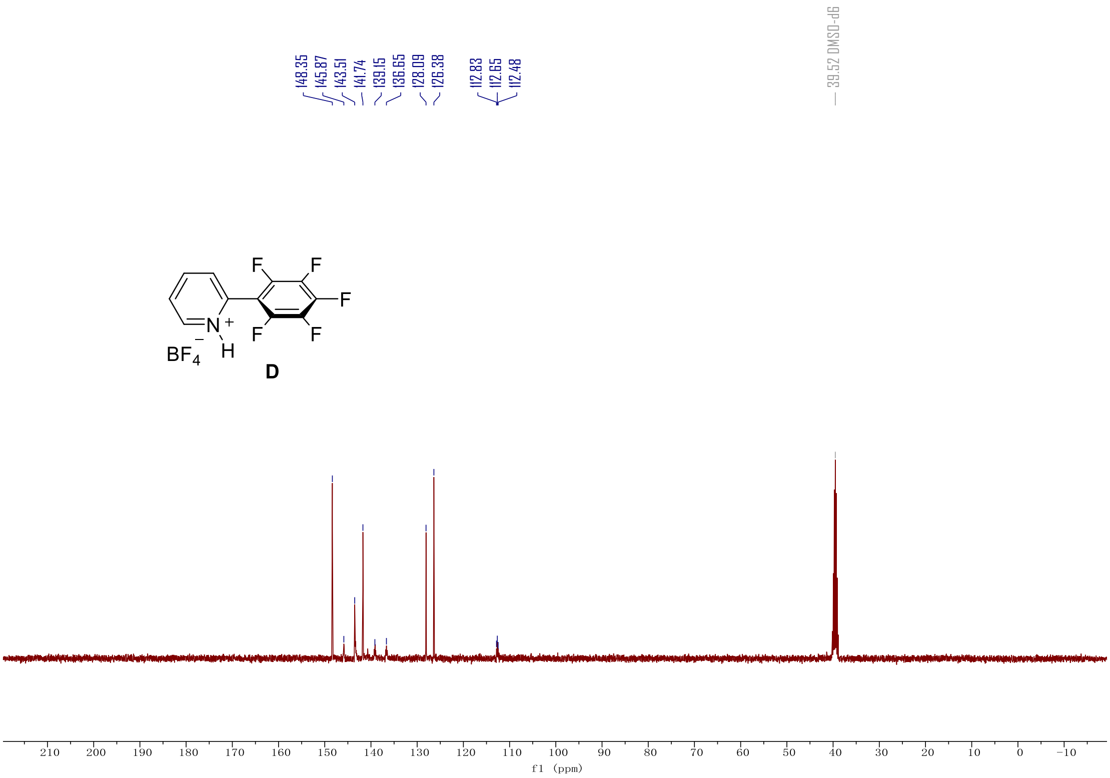


**Supplementary Figure S80.** $^{13}$**C NMR (101 MHz, DMSO-*d*$_6$) Spectra for compound D**

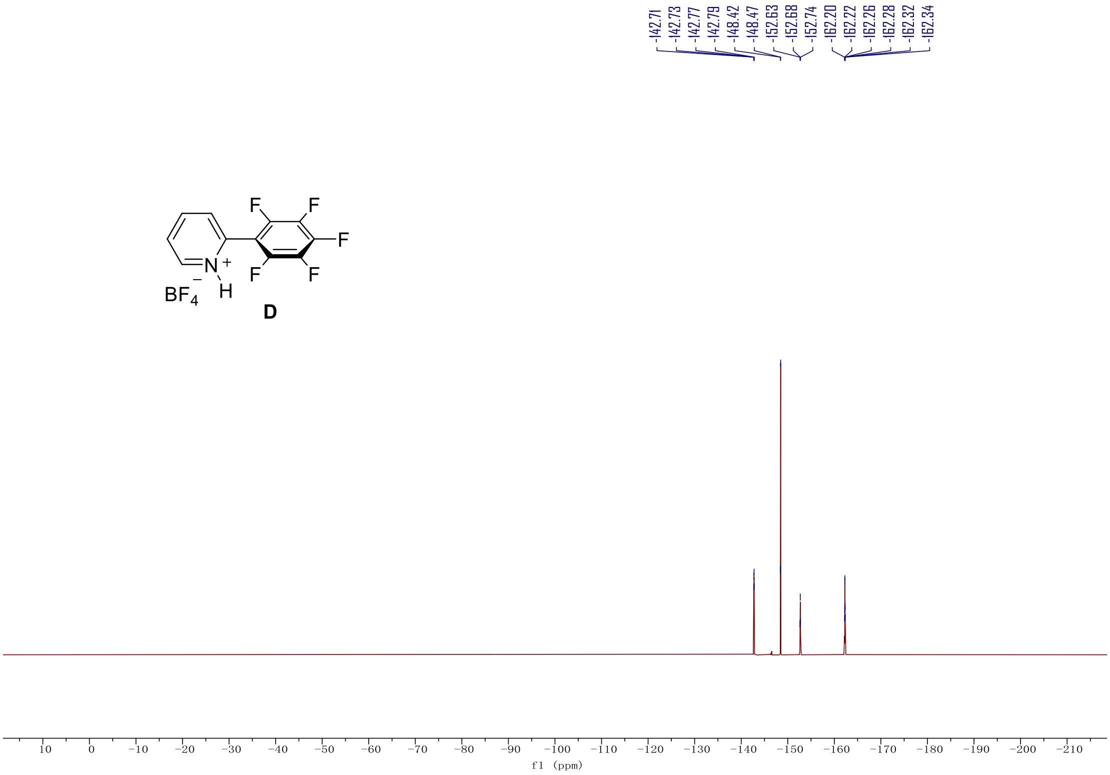


**Supplementary Figure S81.** $^{19}$**F NMR (376 MHz, DMSO-*d*$_6$) Spectra for compound D**

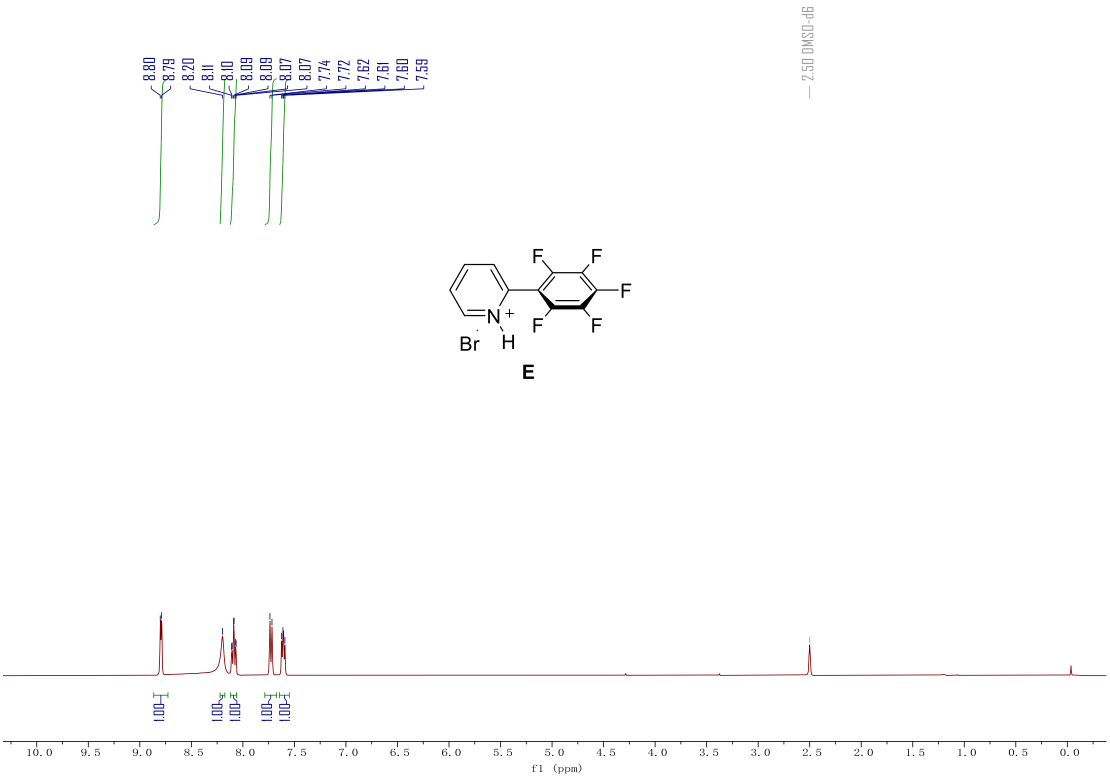

**Supplementary Figure S82.** **$^{1}$H NMR (400 MHz, DMSO-$d_6$) Spectra for compound E**

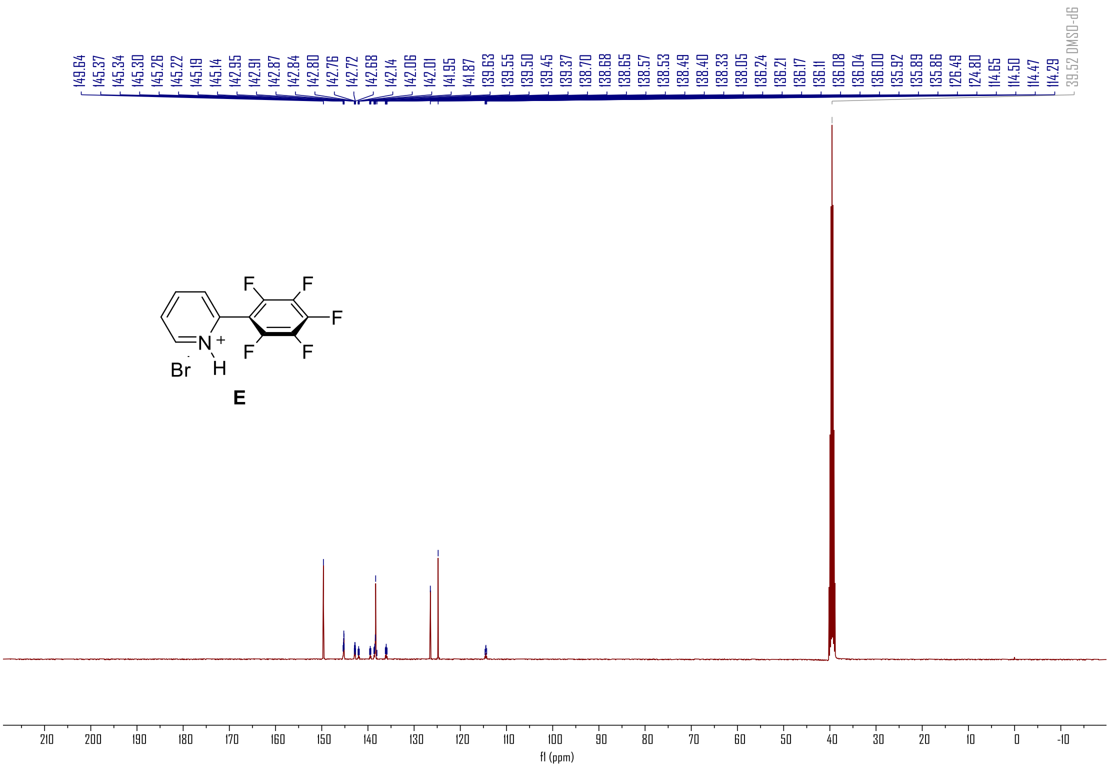

**Supplementary Figure S83.** **$^{13}$C NMR (101 MHz, DMSO-$d_6$) Spectra for compound E**

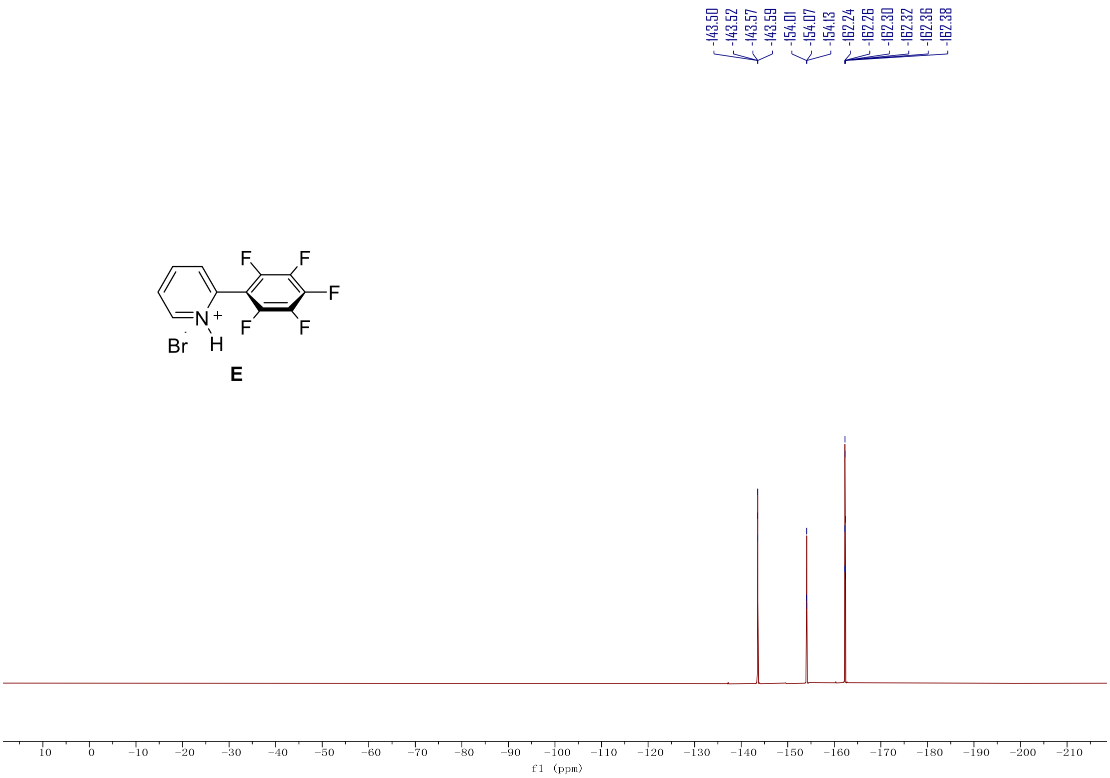


**Supplementary Figure S84. $^{19}$F NMR (376 MHz, DMSO-$d_6$) Spectra for compound E**

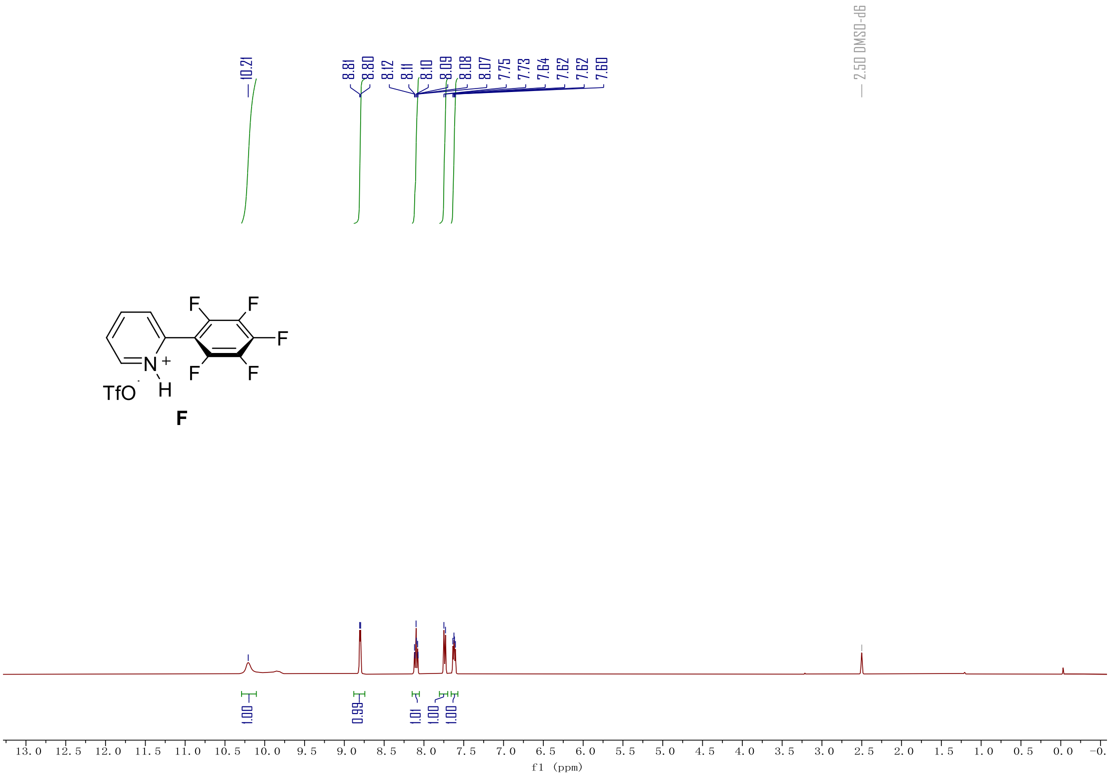


**Supplementary Figure S85. $^1$H NMR (400 MHz, DMSO-$d_6$) Spectra for compound F**

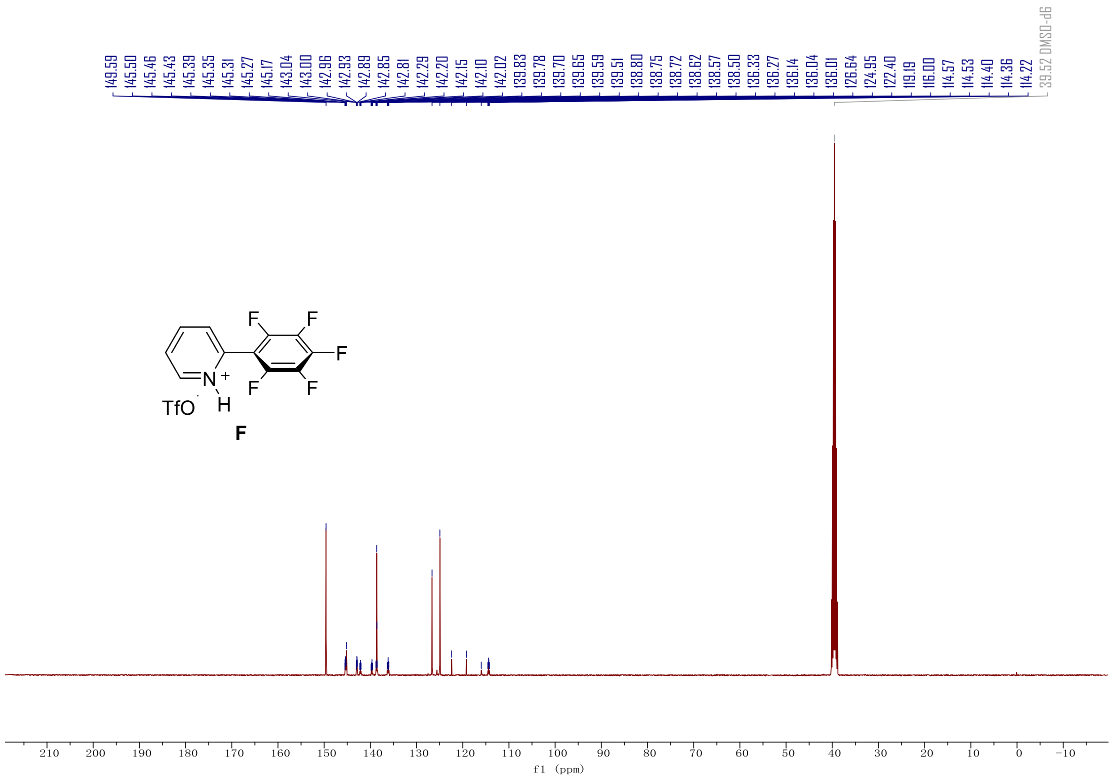

**Supplementary Figure S86. $^{13}$C NMR (101 MHz, DMSO-$d_6$) Spectra for compound F**

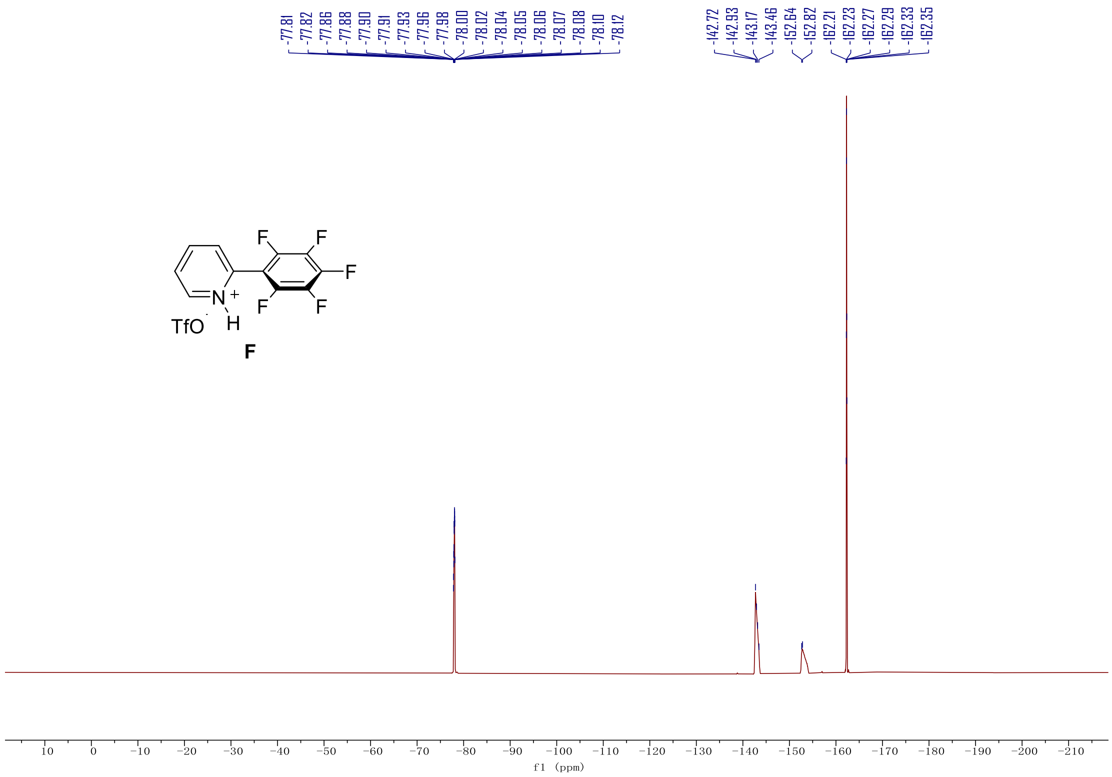

**Supplementary Figure S87. $^{19}$F NMR (376 MHz, DMSO-$d_6$) Spectra for compound F**

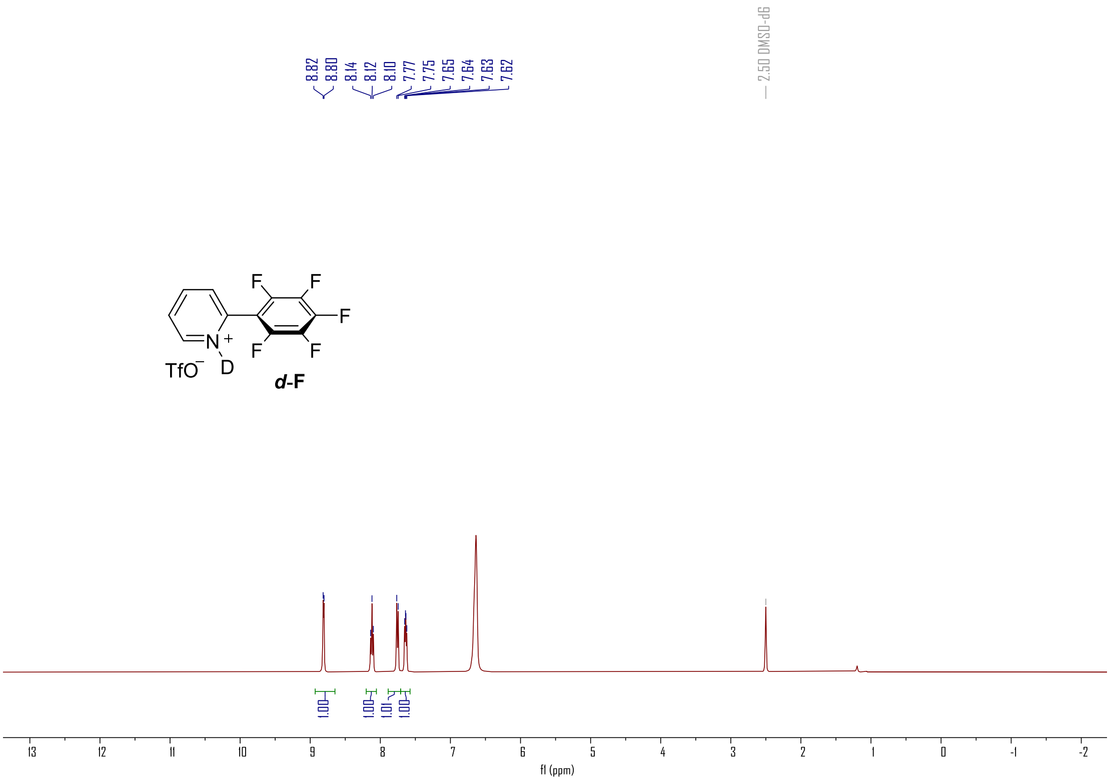


**Supplementary Figure S88.** $^{1}$**H NMR (400 MHz, DMSO-**$d_6$**) Spectra for compound** ***d*-F**

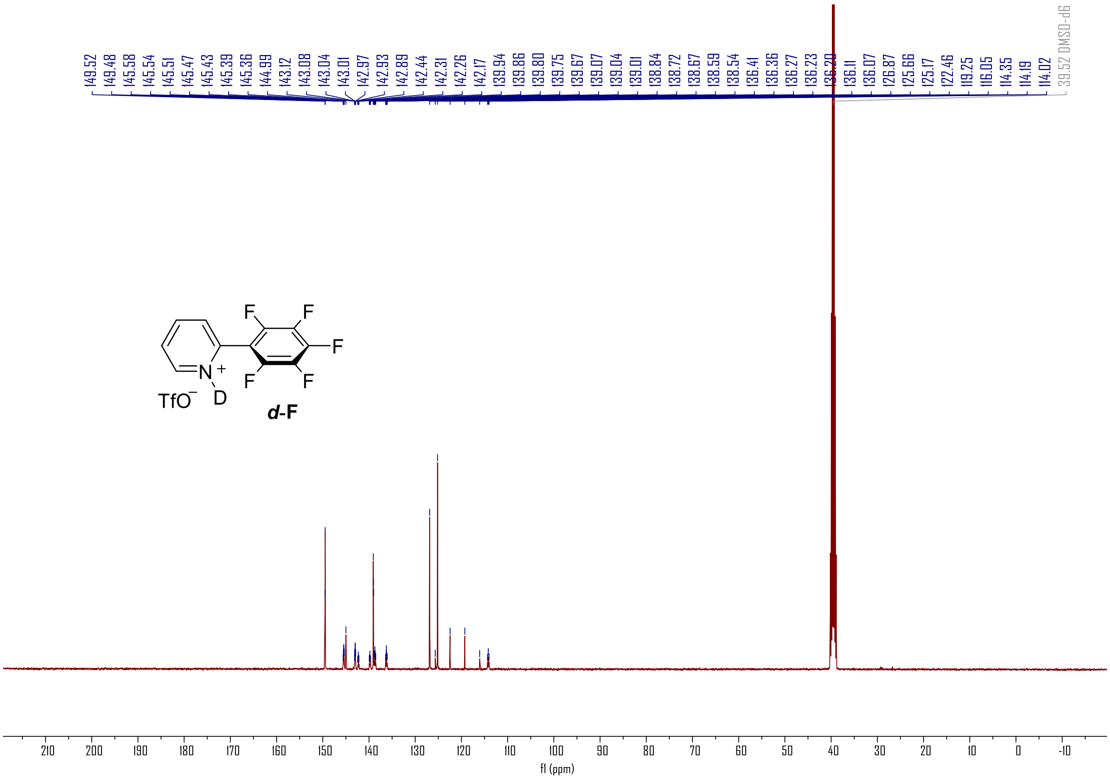


**Supplementary Figure S89.** $^{13}$**C NMR (101 MHz, DMSO-**$d_6$**) Spectra for compound** ***d*-F**

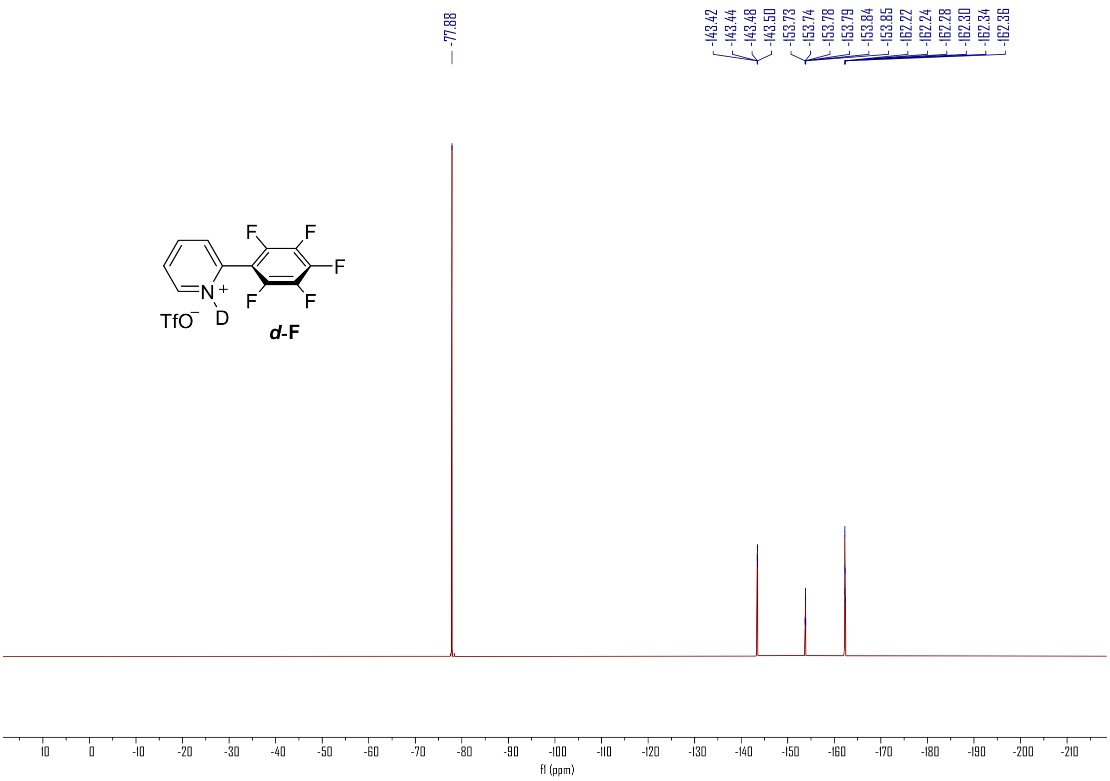

**Supplementary Figure S90. $^{19}$F NMR (376 MHz, DMSO-$d_6$) Spectra for compound *d*-F**

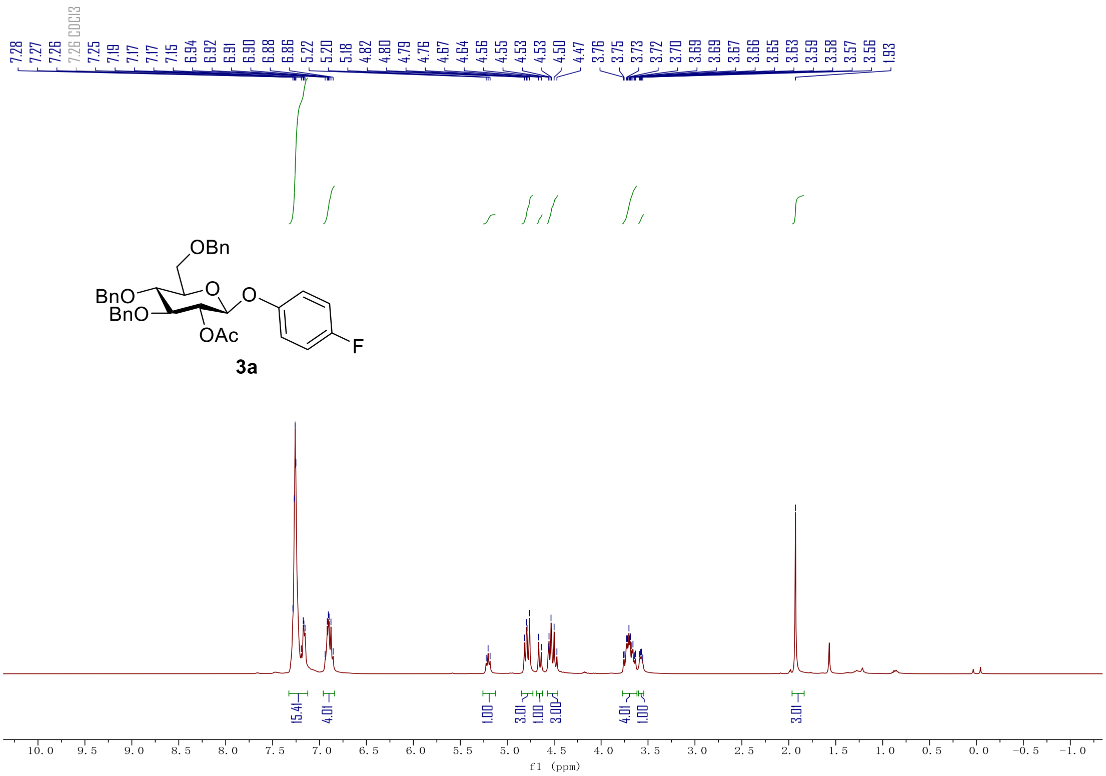

**Supplementary Figure S91. $^{1}$H NMR (400 MHz, CDCl$_3$) Spectra for compound 3a**

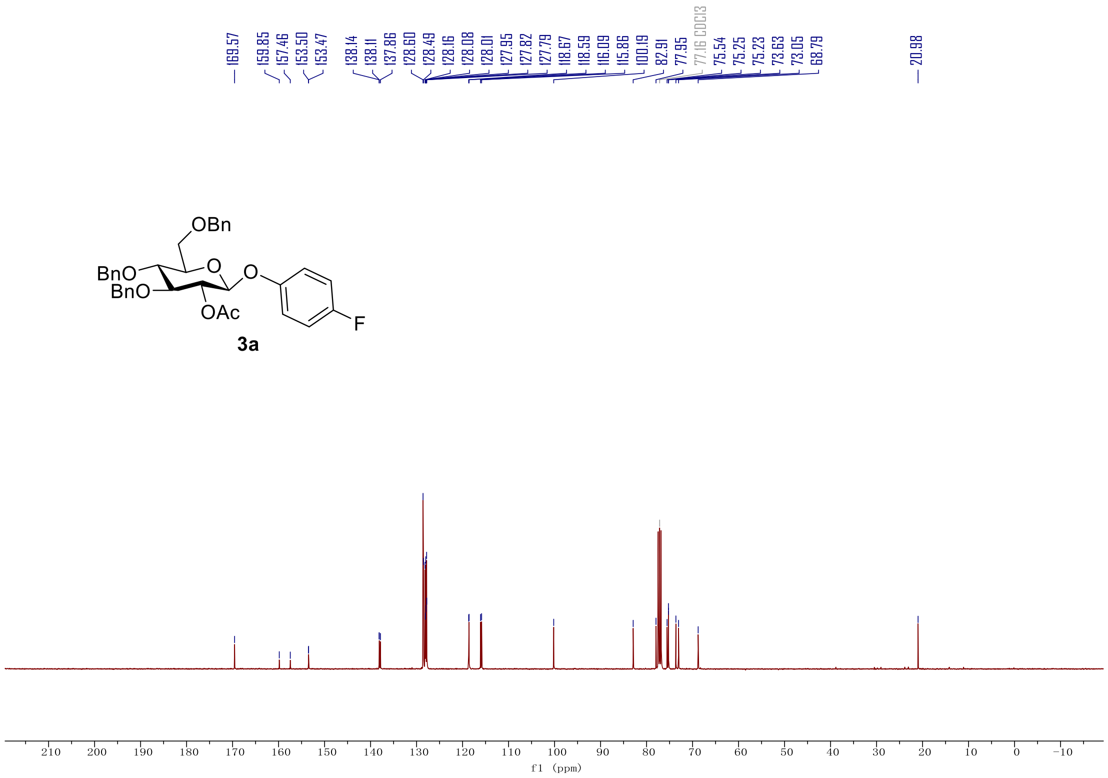


**Supplementary Figure S92. $^{13}$C NMR (101 MHz, $CDCl_3$) Spectra for compound 3a**

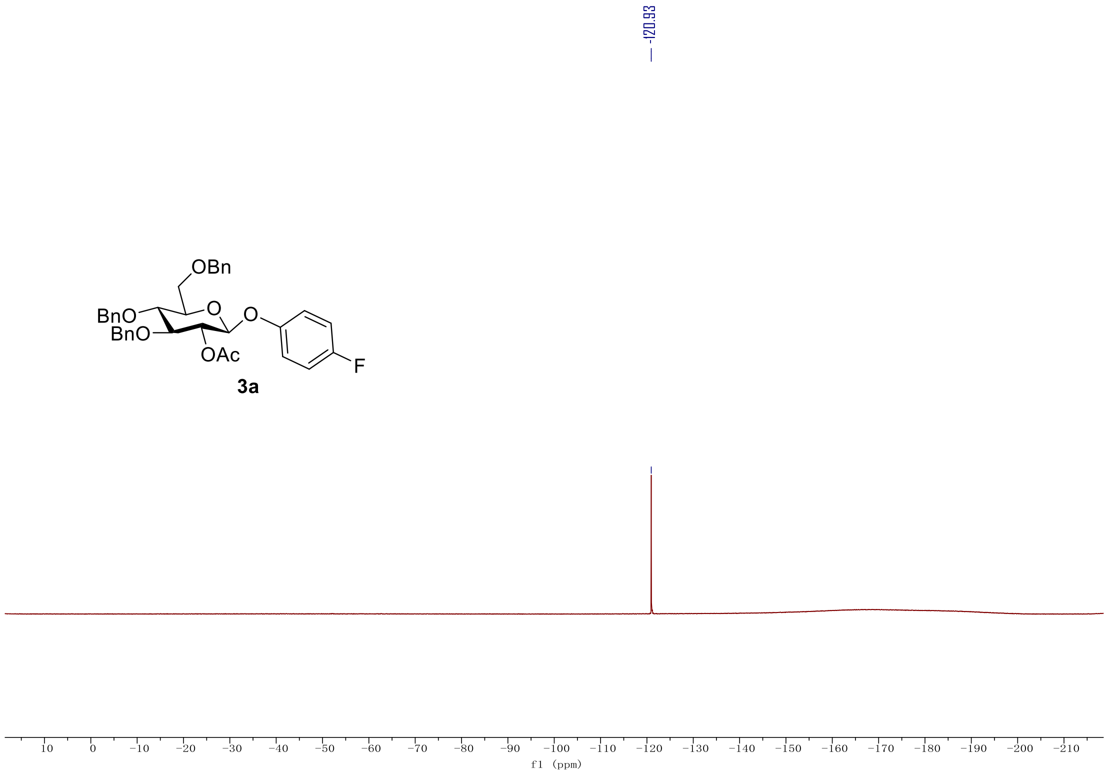


**Supplementary Figure S93. $^{19}$F NMR (376 MHz, $CDCl_3$) Spectra for compound 3a**

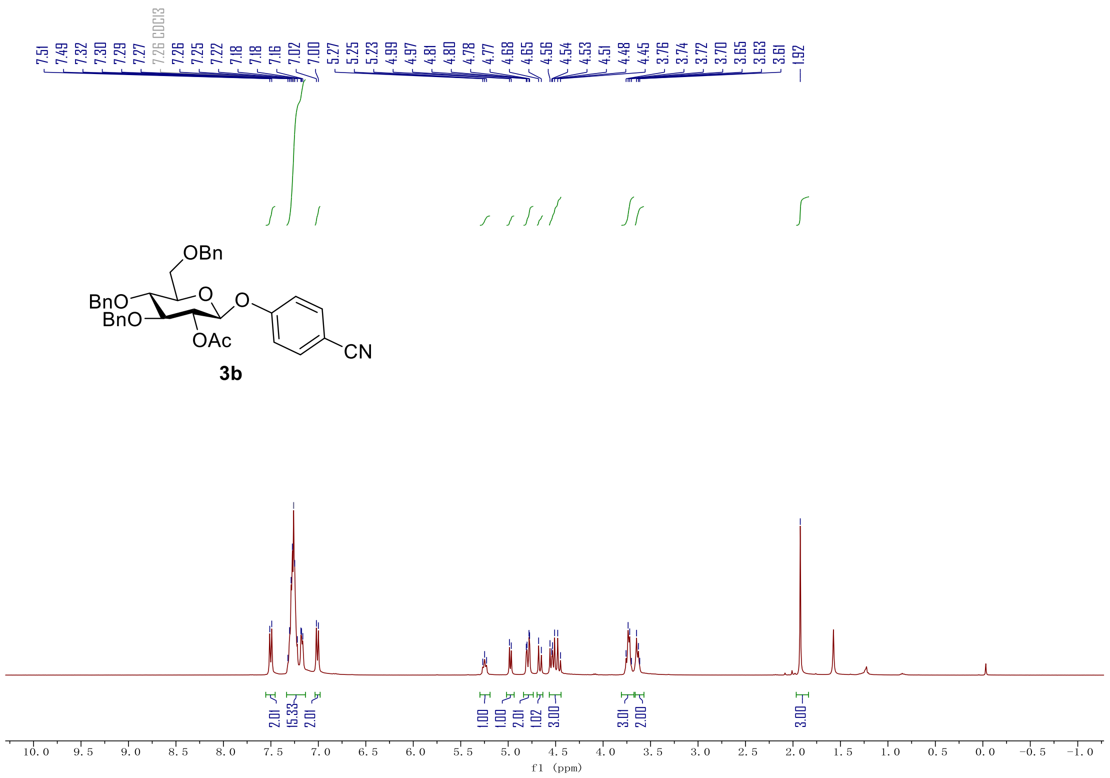

**Supplementary Figure S94. $^1$H NMR (400 MHz, $CDCl_3$) Spectra for compound 3b**

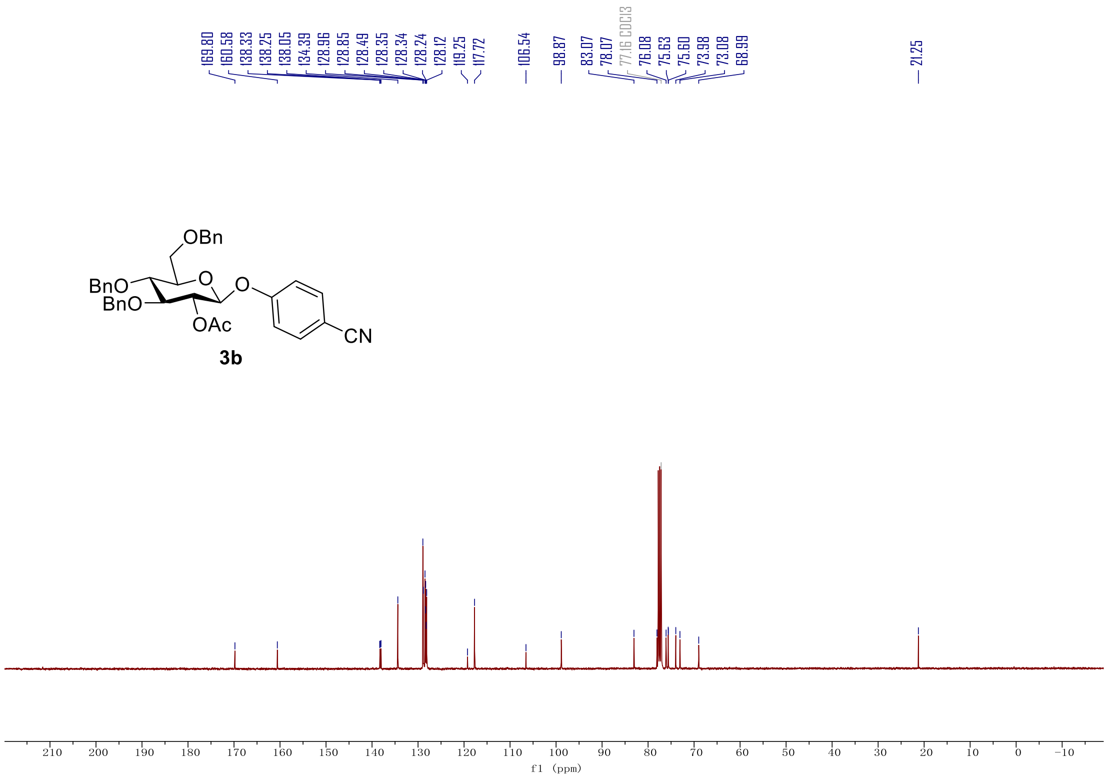

**Supplementary Figure S95. $^{13}$C NMR (101 MHz, $CDCl_3$) Spectra for compound 3b**

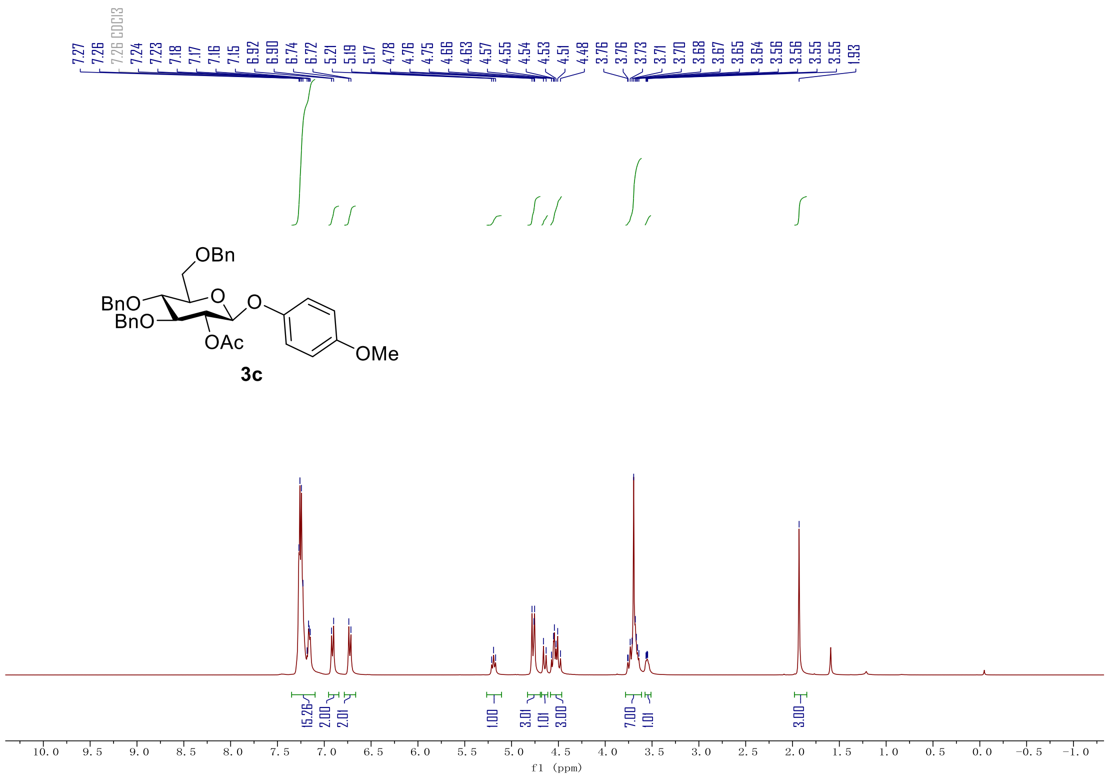

**Supplementary Figure S96. $^{1}$H NMR (400 MHz, $CDCl_3$) Spectra for compound 3c**

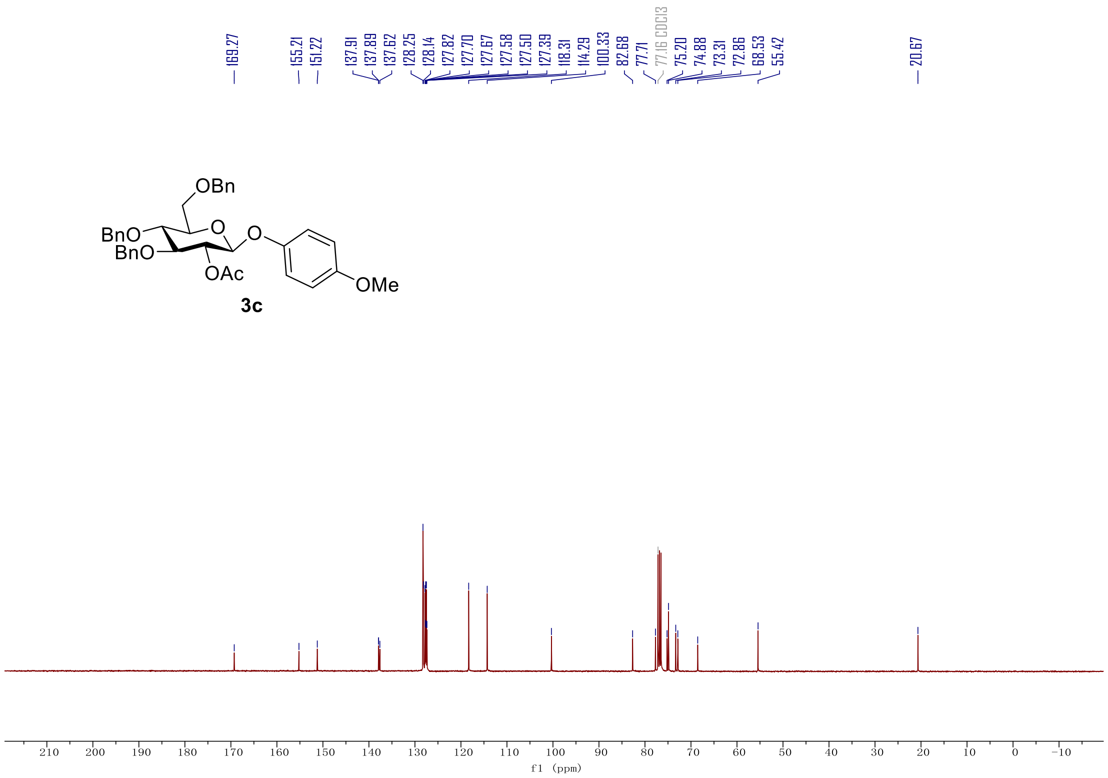

**Supplementary Figure S97. $^{13}$C NMR (101 MHz, $CDCl_3$) Spectra for compound 3c**

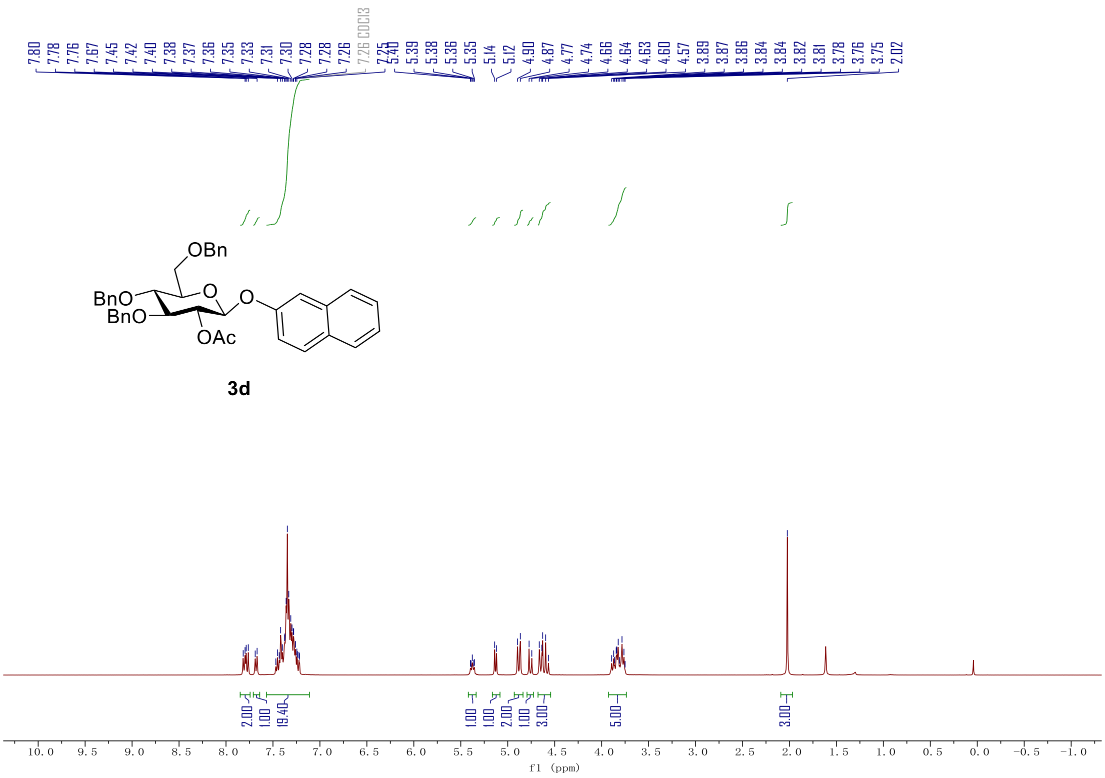

Supplementary Figure S98. $^{1}$H NMR (400 MHz, $CDCl_3$) Spectra for compound 3d

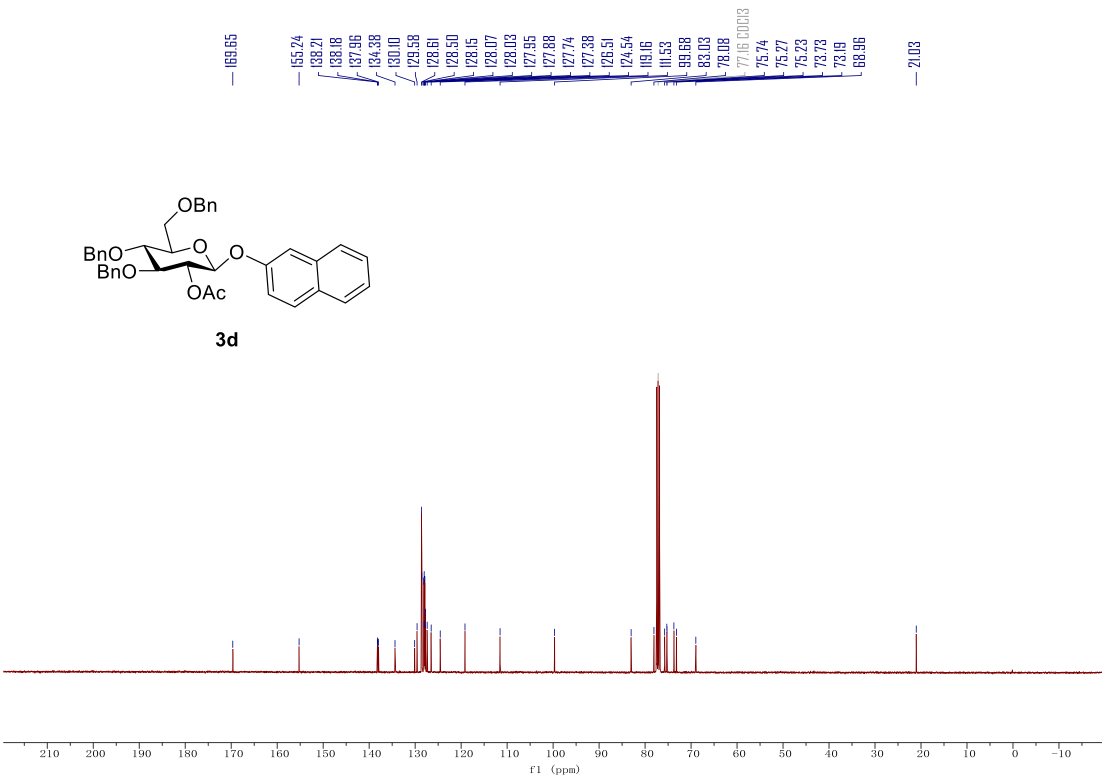

Supplementary Figure S99. $^{13}$C NMR (101 MHz, $CDCl_3$) Spectra for compound 3d

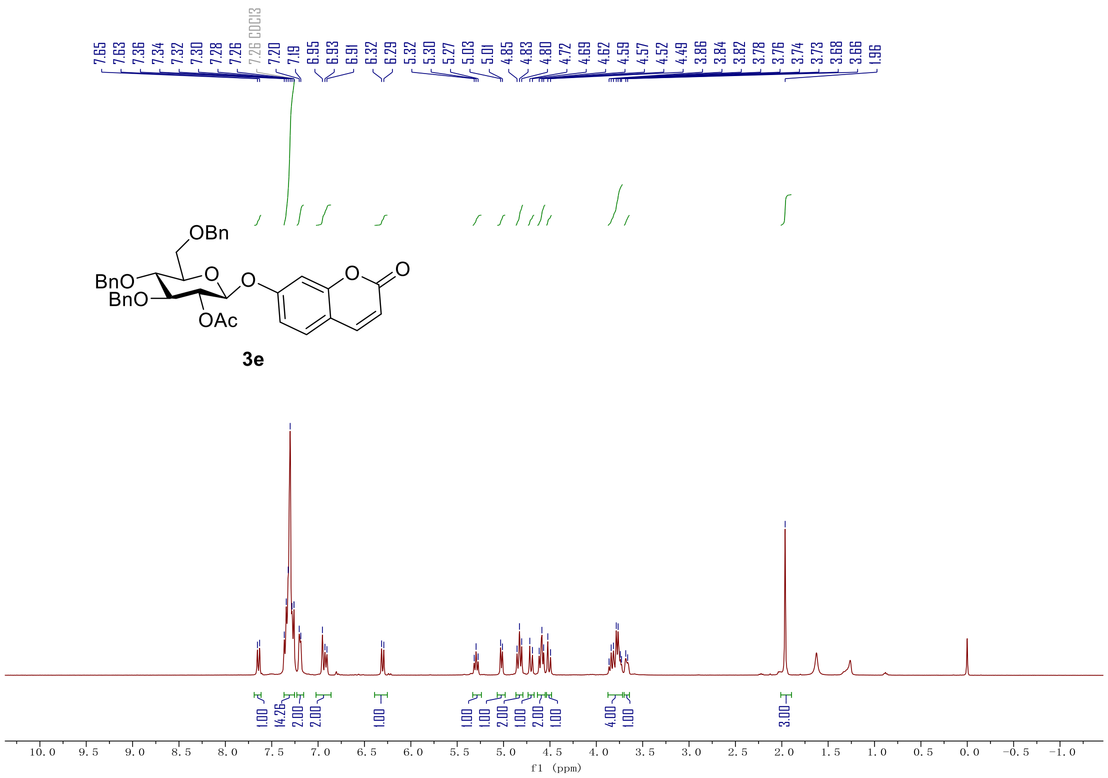


**Supplementary Figure S100. ¹H NMR (400 MHz, $CDCl_3$) Spectra for compound 3e**

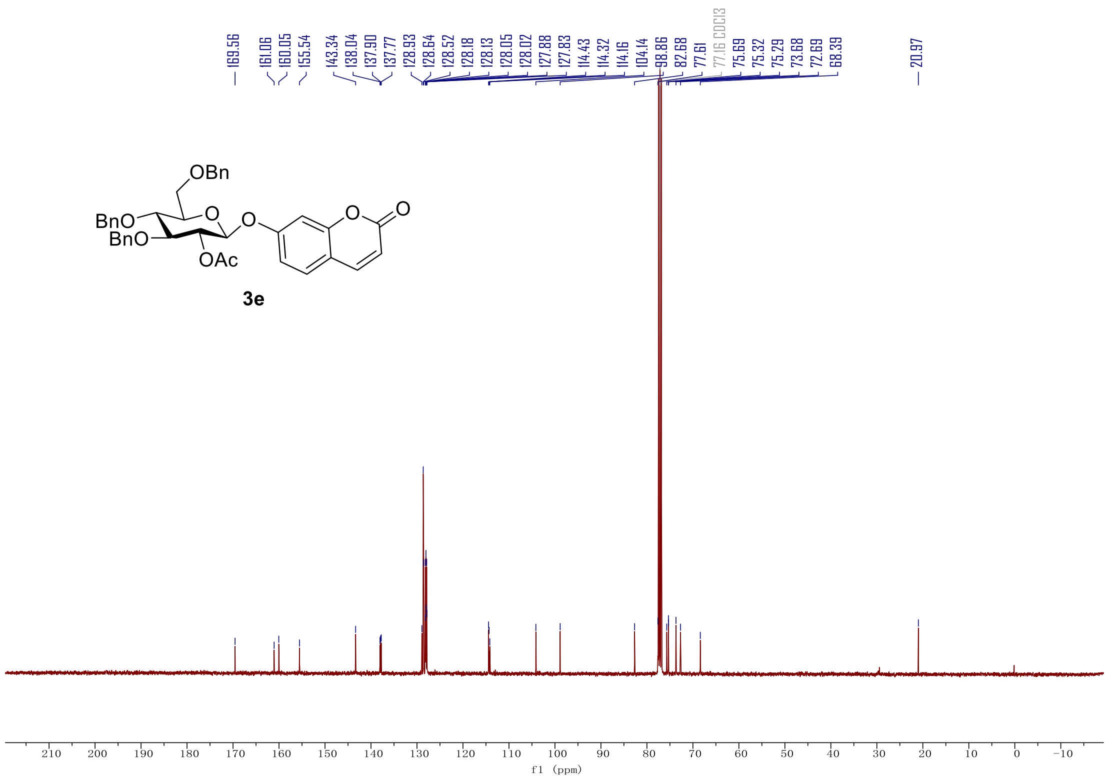


**Supplementary Figure S101. ¹³C NMR (101 MHz, $CDCl_3$) Spectra for compound 3e**

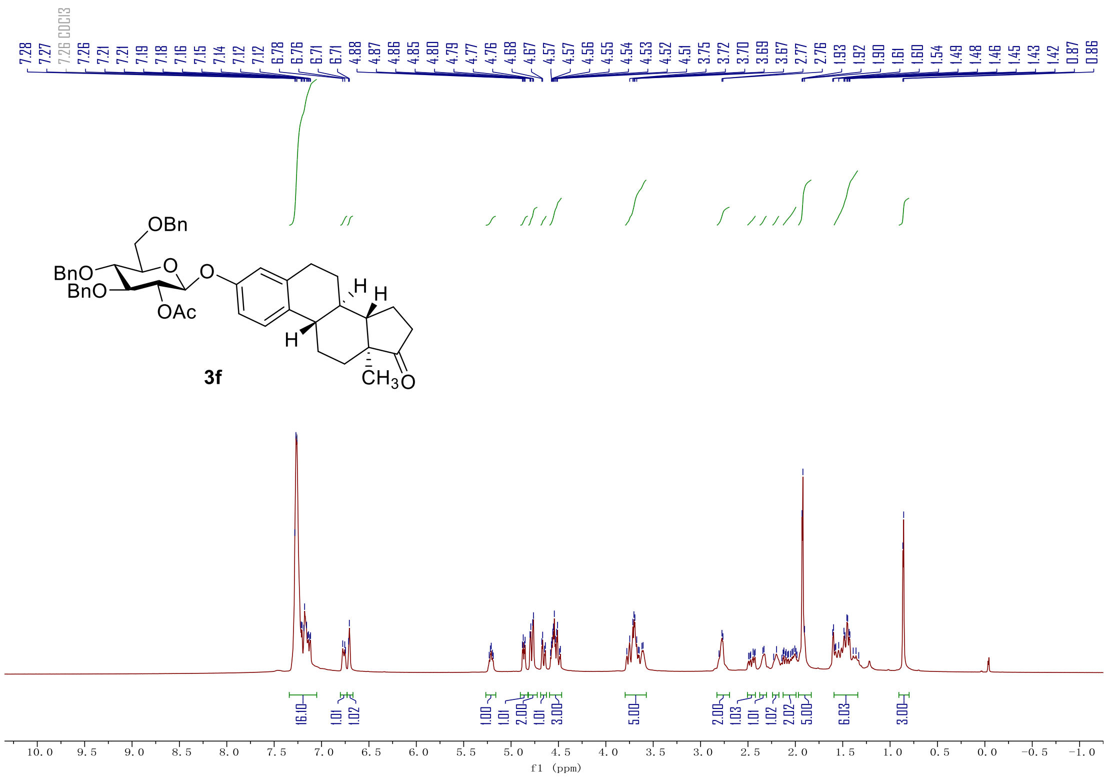

**Supplementary Figure S102.** $^{1}$**H NMR (400 MHz, CDCl$_3$) Spectra for compound 3f**

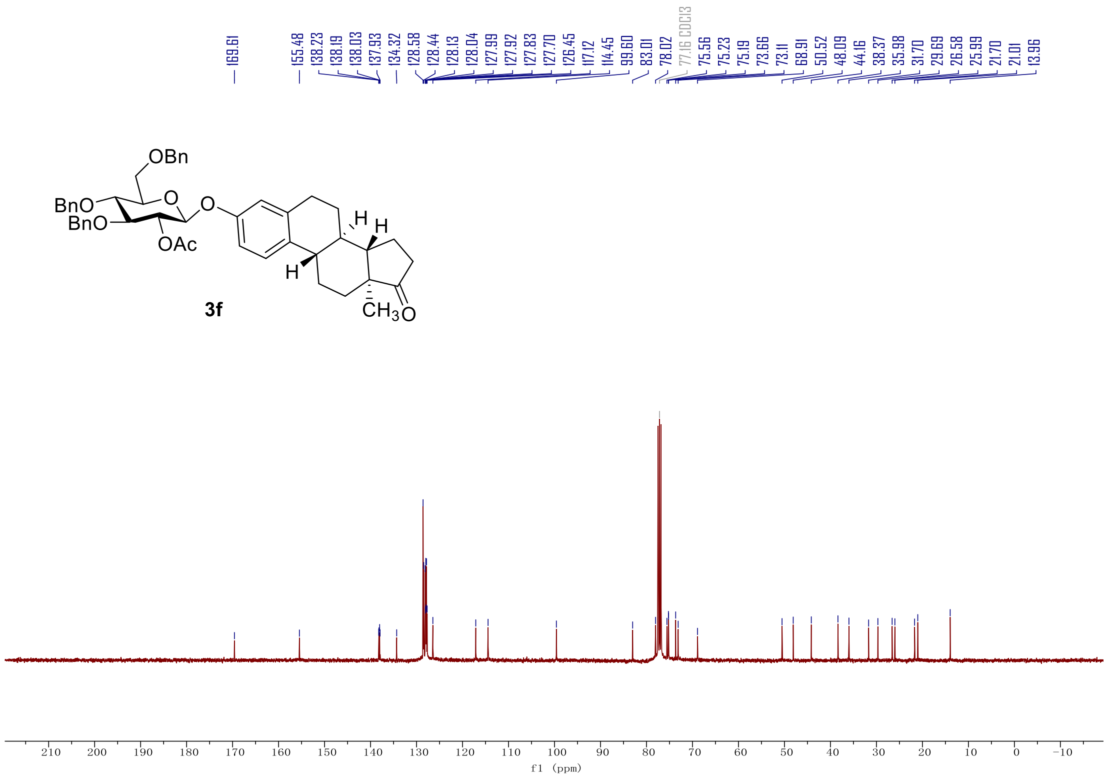

**Supplementary Figure S103.** $^{13}$**C NMR (101 MHz, CDCl$_3$) Spectra for compound 3f**

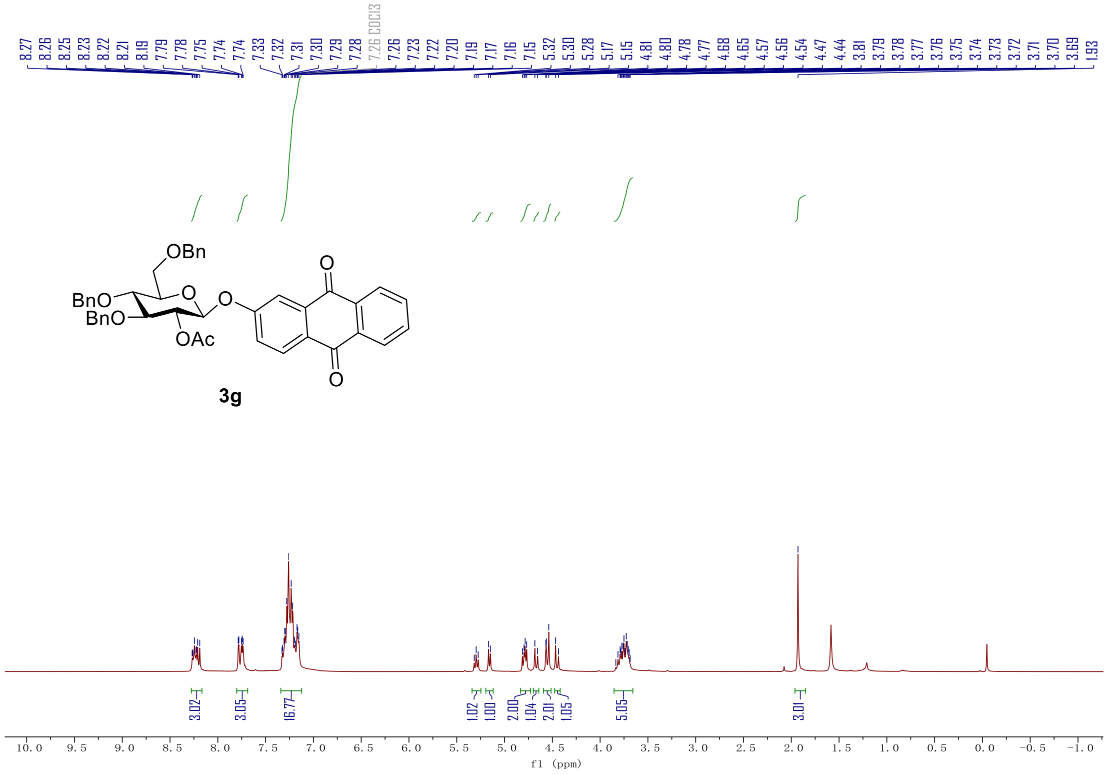


**Supplementary Figure S104. $^{1}H$ NMR (400 MHz, $CDCl_3$) Spectra for compound 3g**

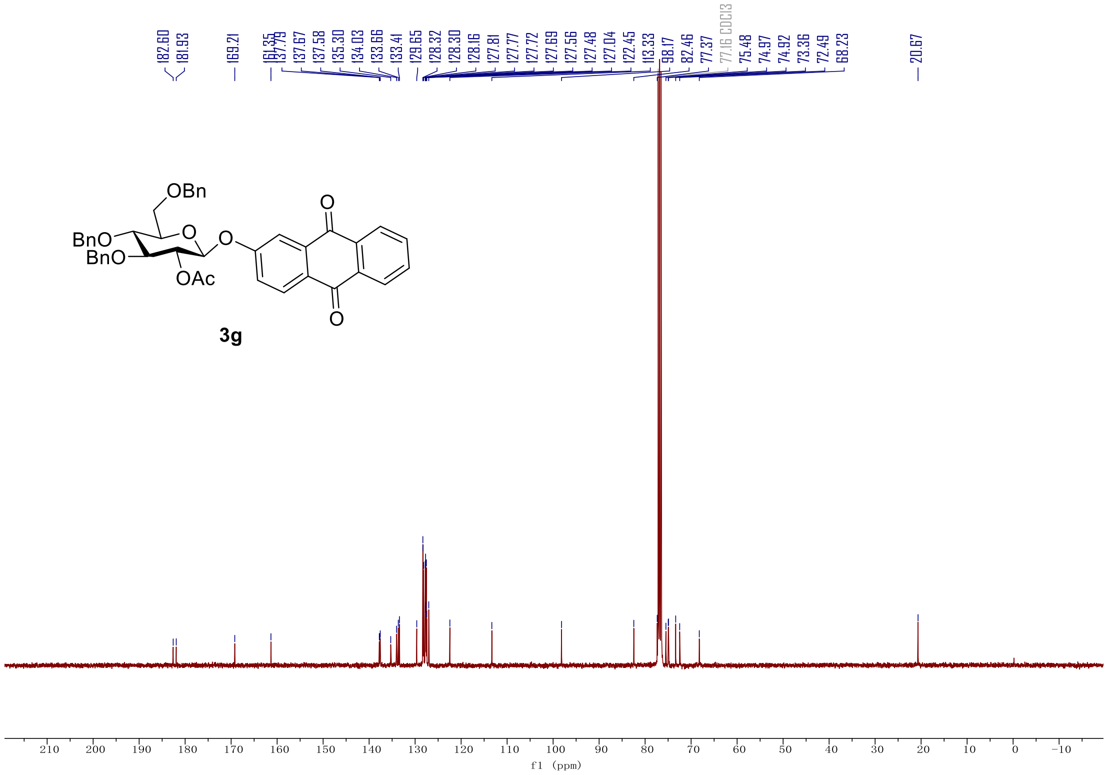


**Supplementary Figure S105. $^{13}C$ NMR (101 MHz, $CDCl_3$) Spectra for compound 3g**

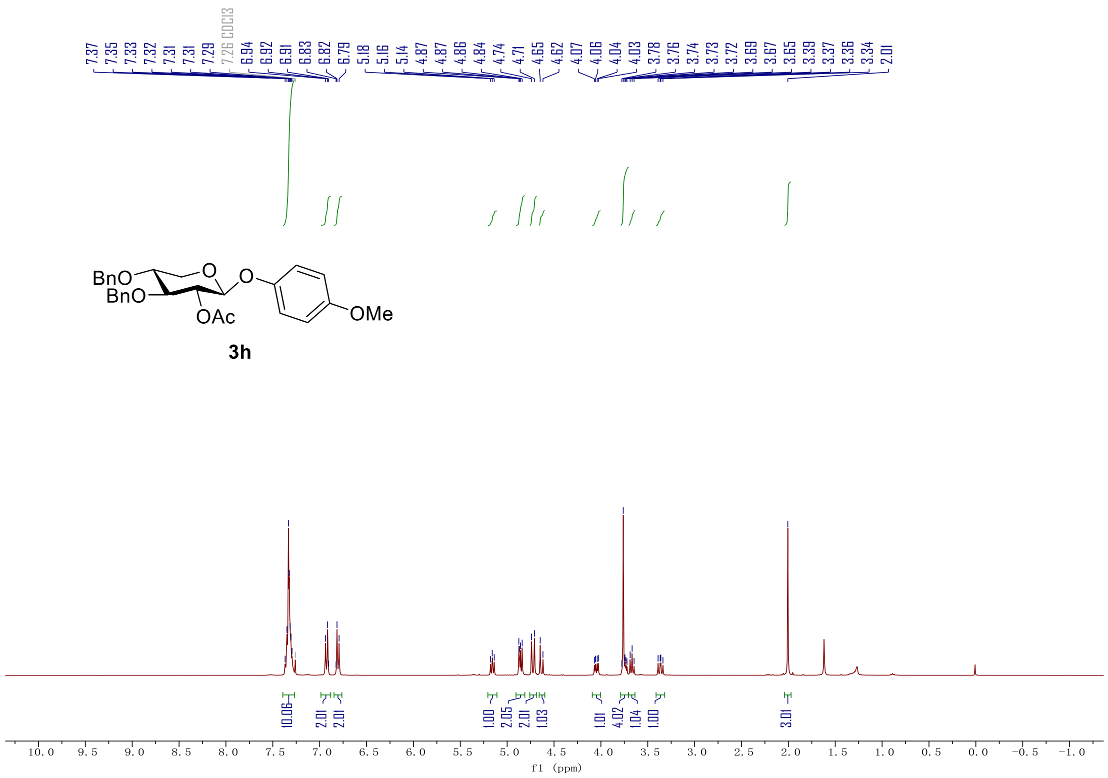

Supplementary Figure S106. $^{1}$H NMR (400 MHz, $CDCl_3$) Spectra for compound 3h

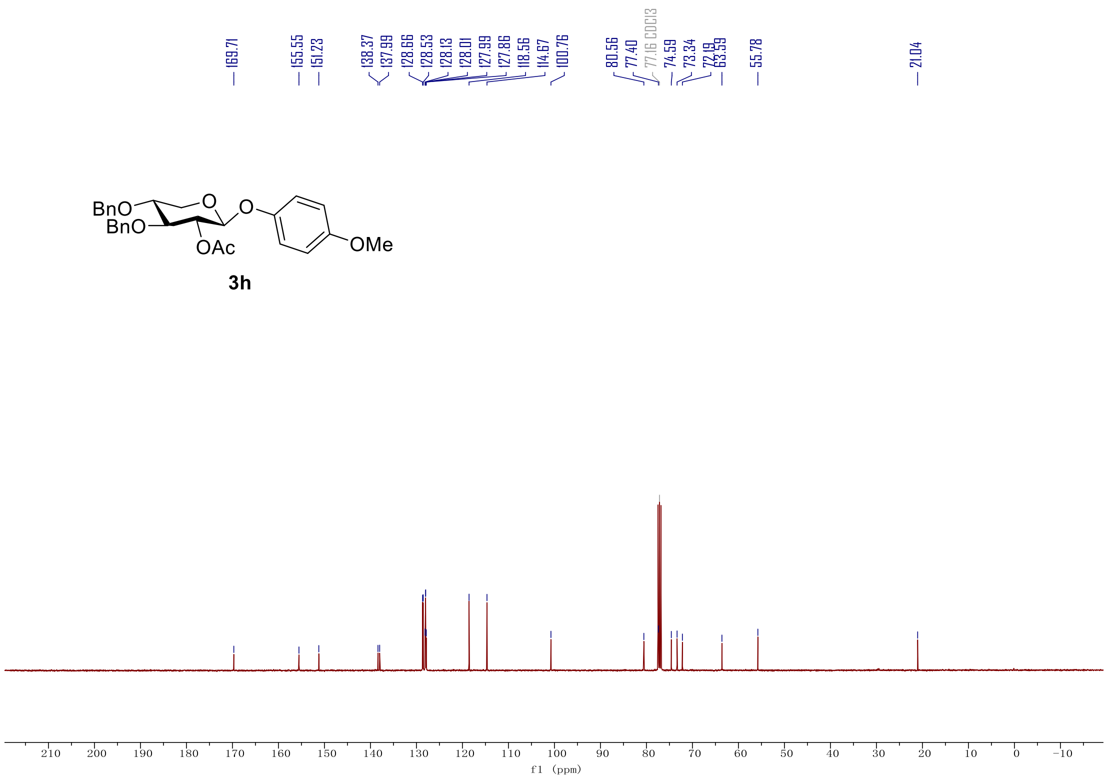

Supplementary Figure S107. $^{13}$C NMR (101 MHz, $CDCl_3$) Spectra for compound 3h

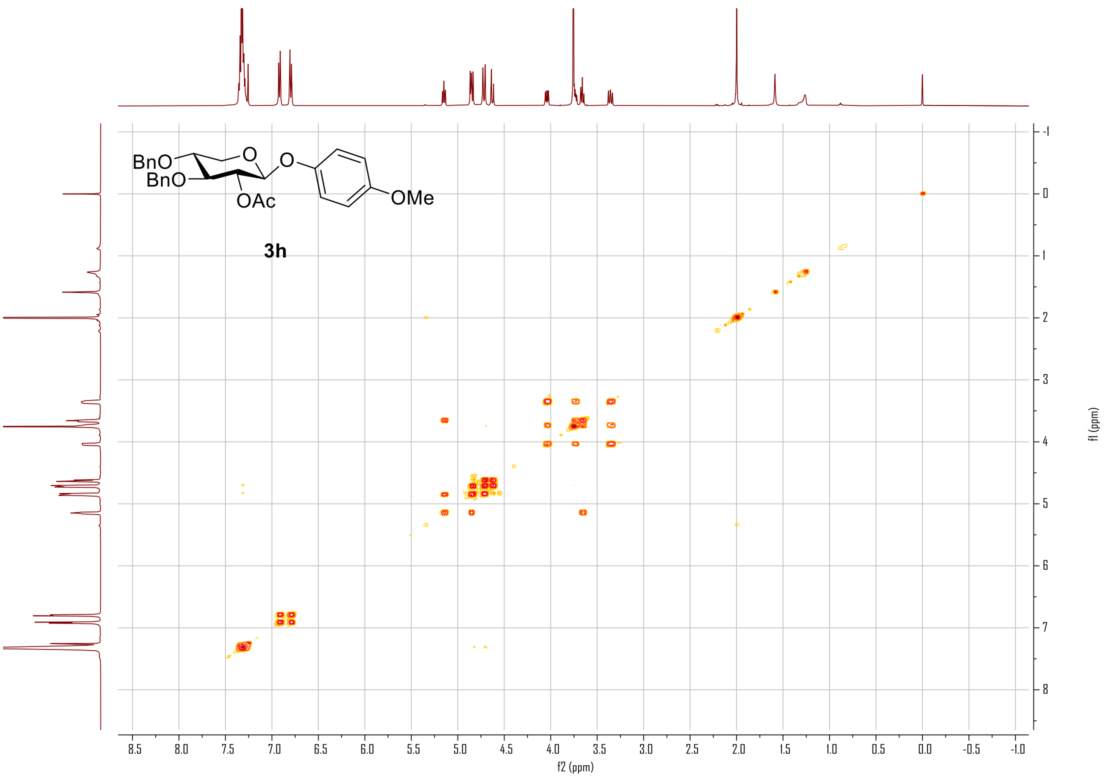


**Supplementary Figure S108. COSY (500 MHz, $CDCl_3$) Spectrum for compound 3h**

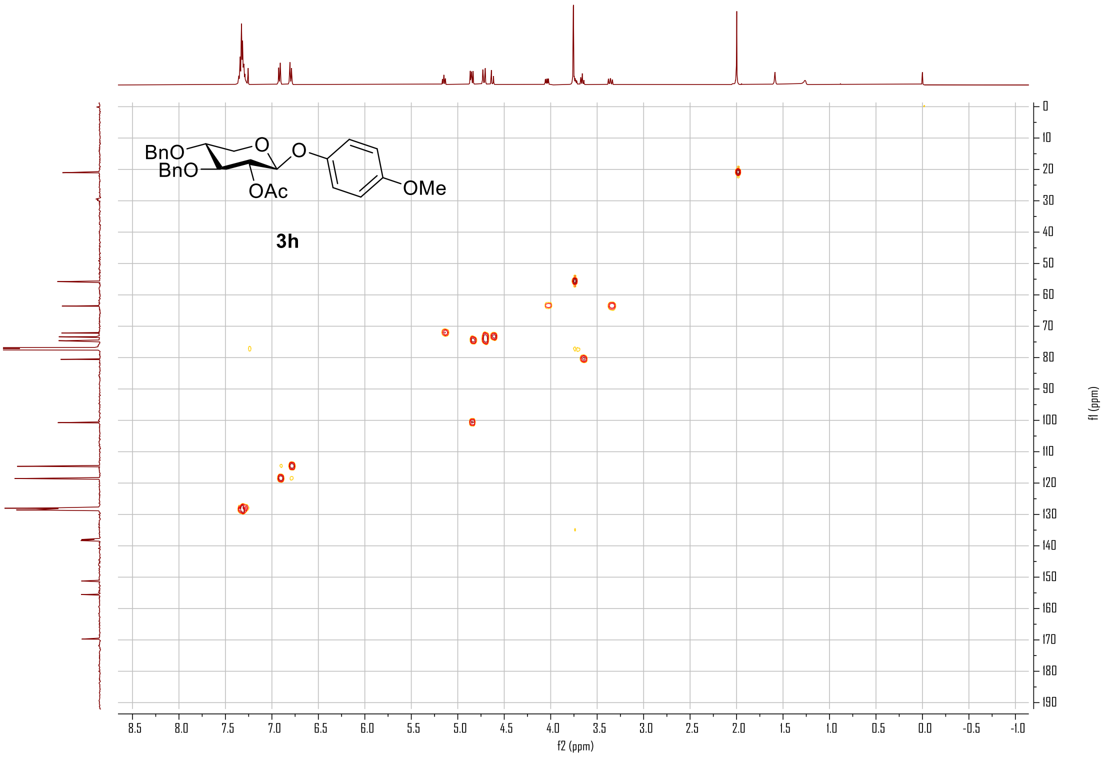


**Supplementary Figure S109. HMQC (500 MHz, $CDCl_3$) Spectrum for compound 3h**

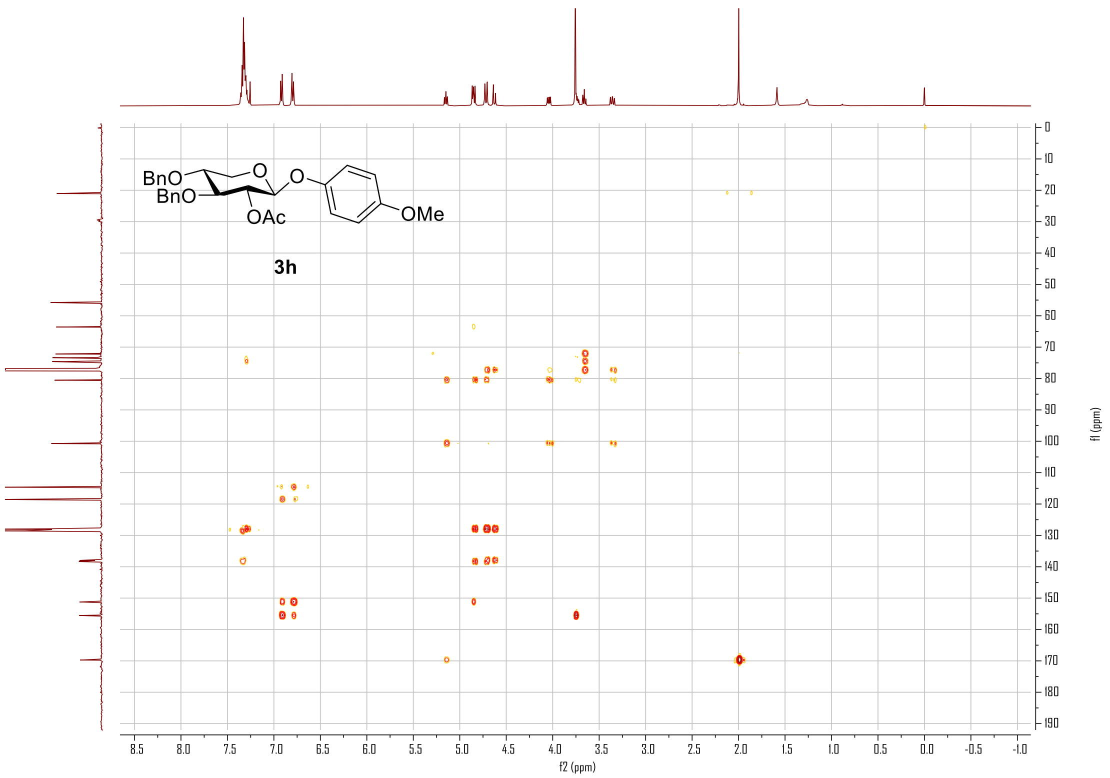


**Supplementary Figure S110. HMBC (500 MHz, CDCl₃) Spectrum for compound 3h**

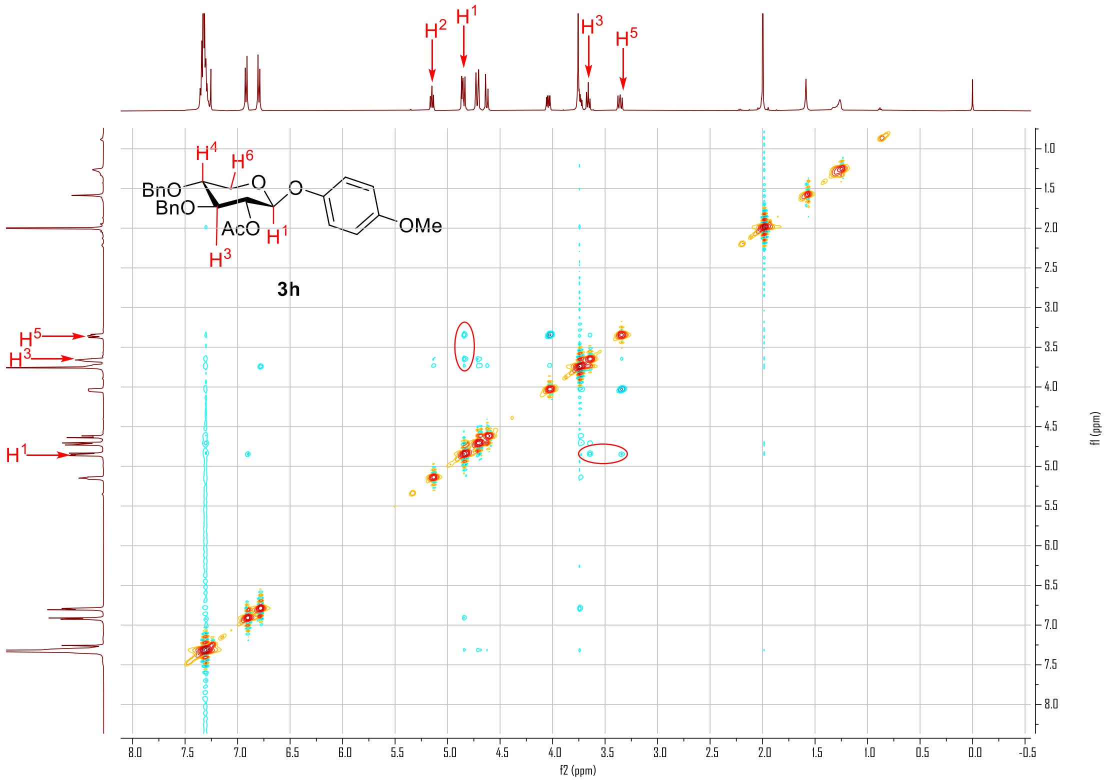


**Supplementary Figure S111. NOESY (500 MHz, $CDCl_3$) Spectrum for compound 3h**

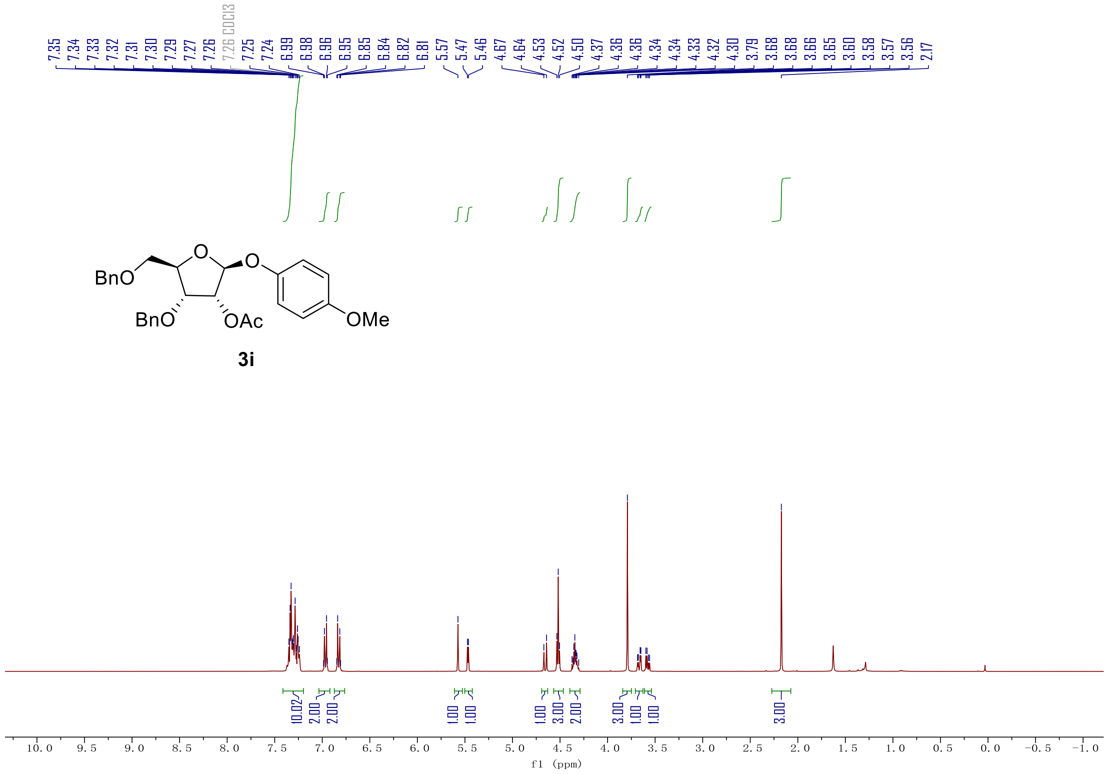


**Supplementary Figure S112. $^{1}$H NMR (400 MHz, $CDCl_3$) Spectra for compound 3i**

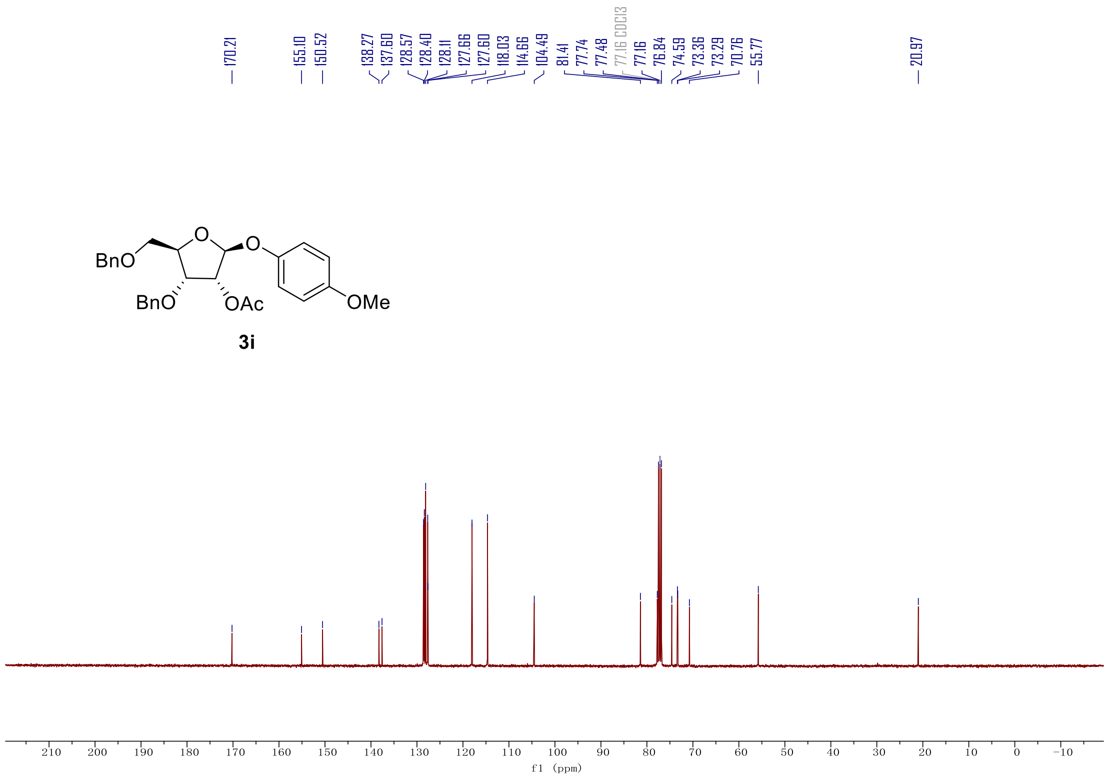


**Supplementary Figure S113. $^{13}$C NMR (101 MHz, $CDCl_3$) Spectra for compound 3i**

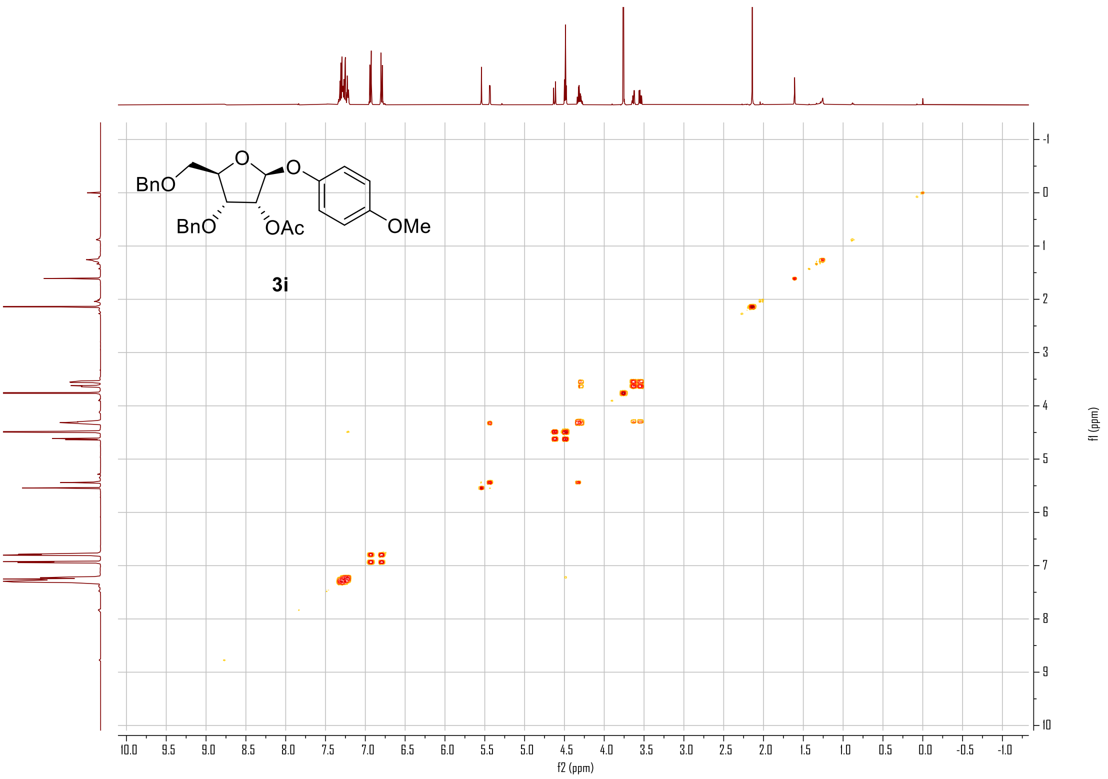


**Supplementary Figure S114. COSY (500 MHz, $CDCl_3$) Spectrum for compound 3i**

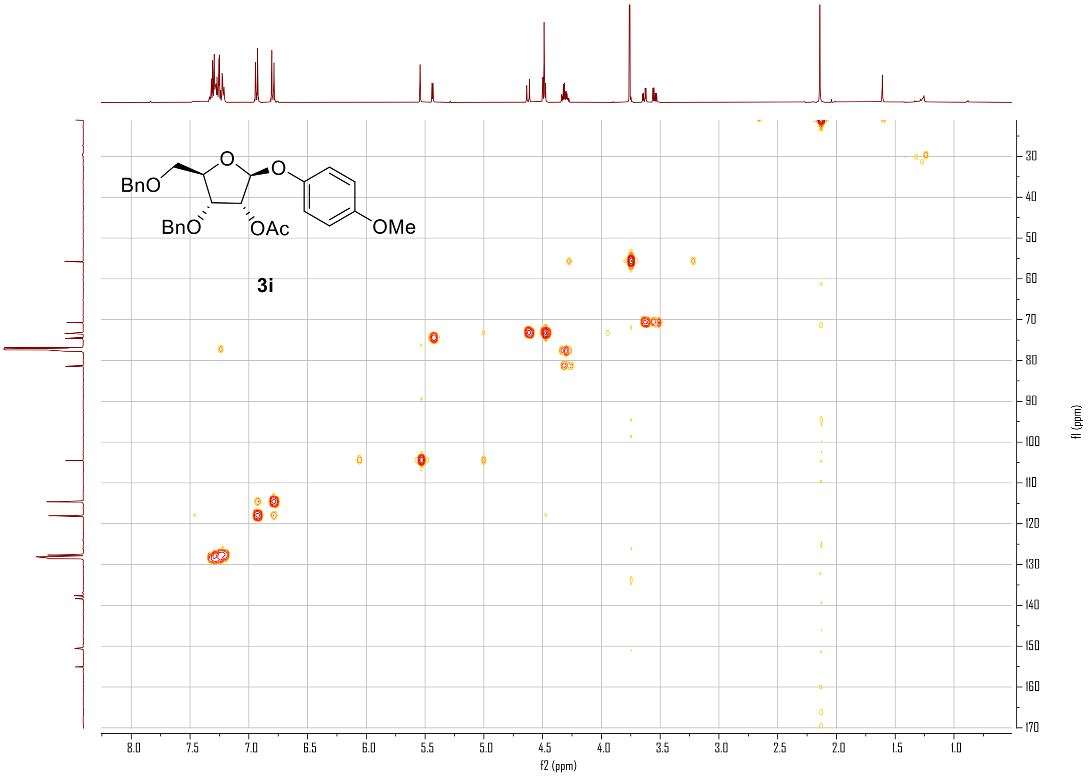


**Supplementary Figure S115. HMQC (500 MHz, $CDCl_3$) Spectrum for compound 3i**

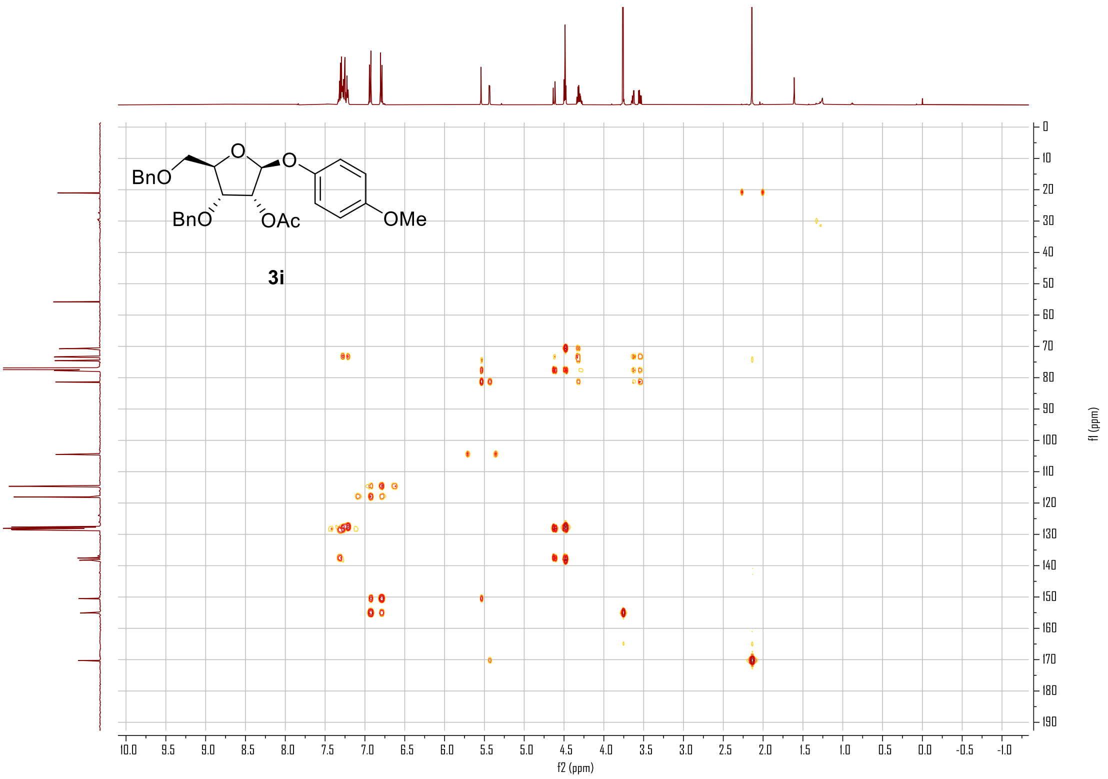


**Supplementary Figure S116. HMBC (500 MHz, $CDCl_3$) Spectrum for compound 3i**

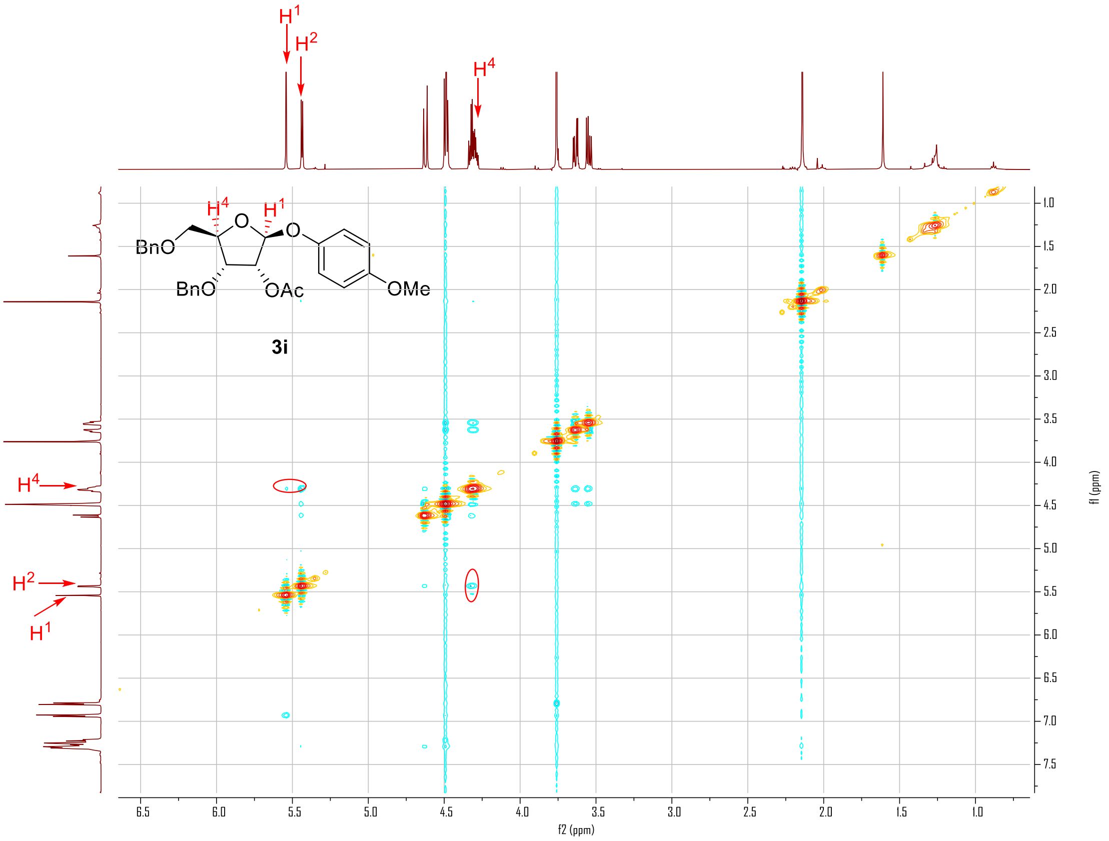


**Supplementary Figure S117. NOESY (500 MHz, $CDCl_3$) Spectrum for compound 3i**

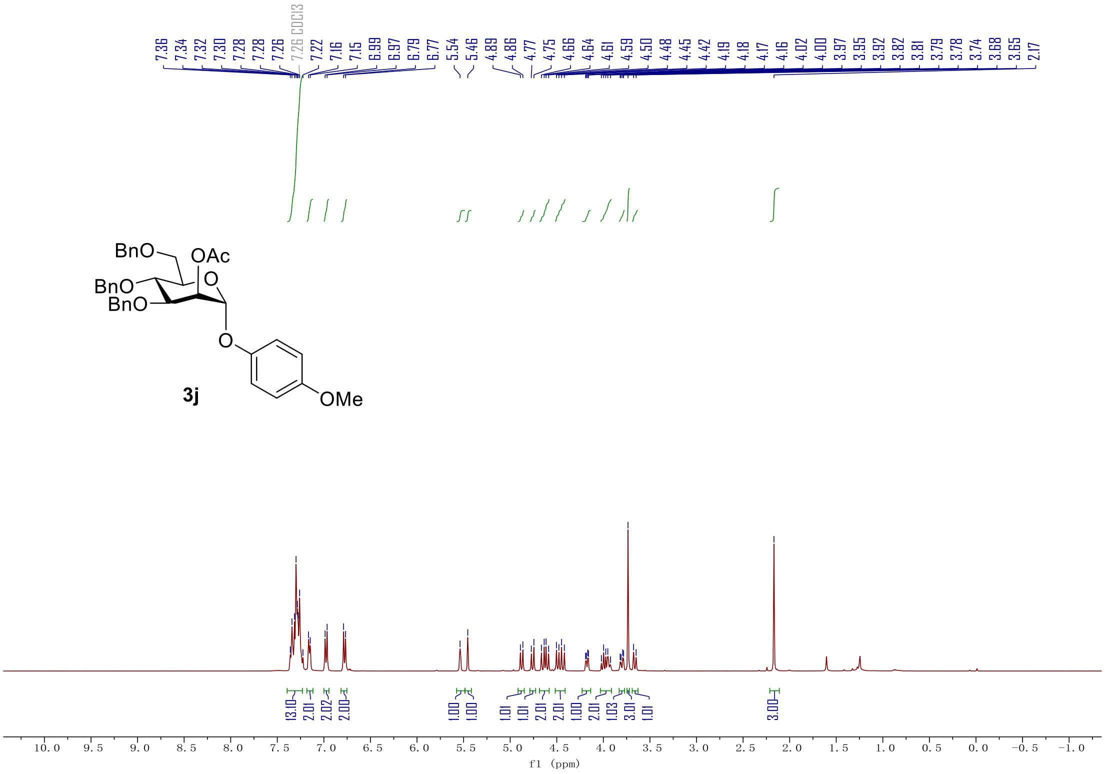


**Supplementary Figure S118. $^{1}$H NMR (400 MHz, $CDCl_3$) Spectra for compound 3j**

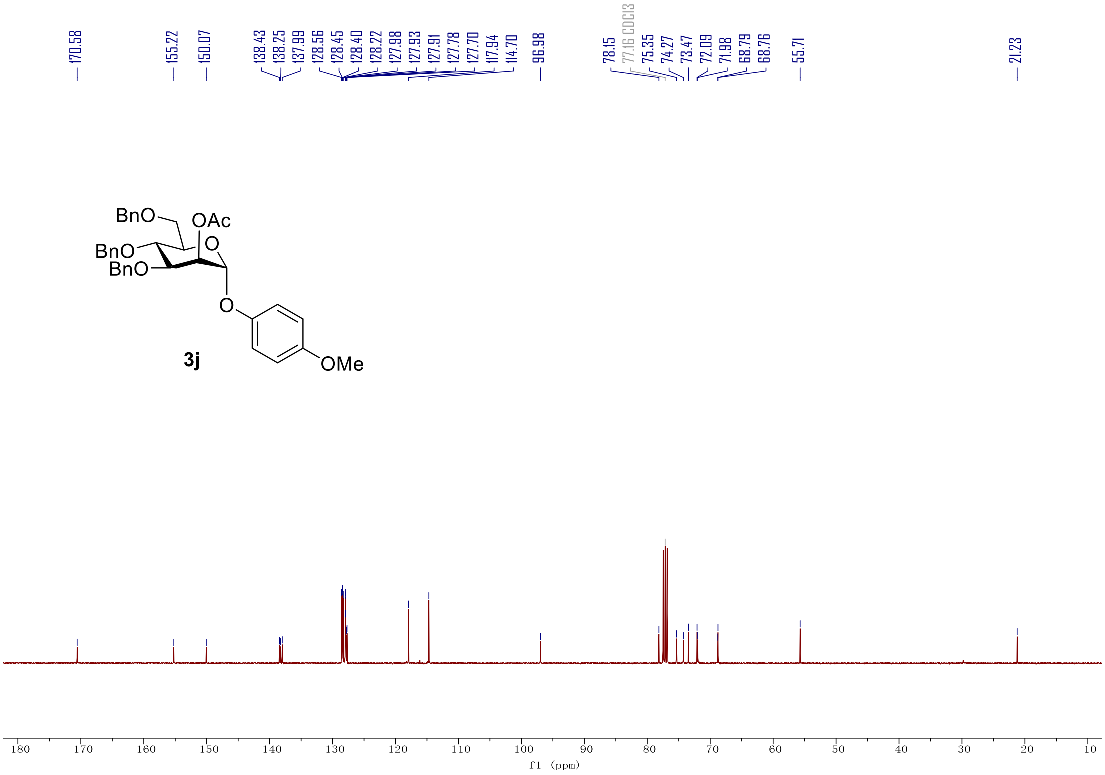


**Supplementary Figure S119. $^{13}$C NMR (101 MHz, $CDCl_3$) Spectra for compound 3j**

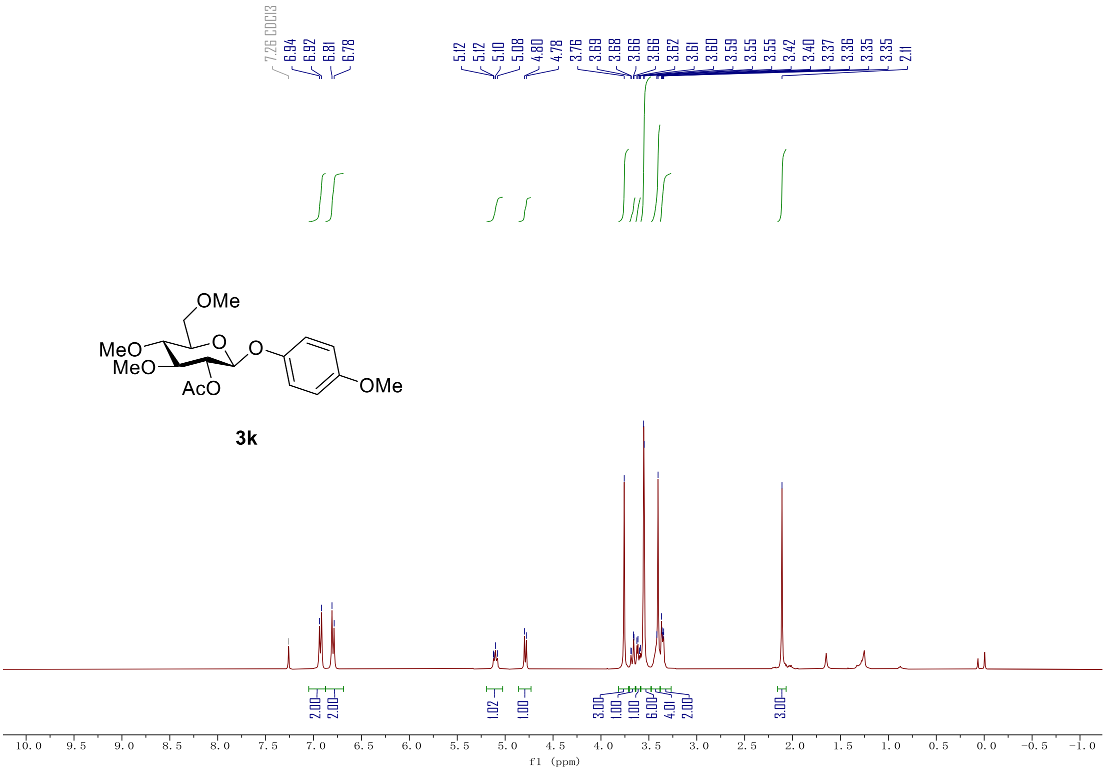

**Supplementary Figure S120. $^{1}$H NMR (400 MHz, $CDCl_3$) Spectra for compound 3k**

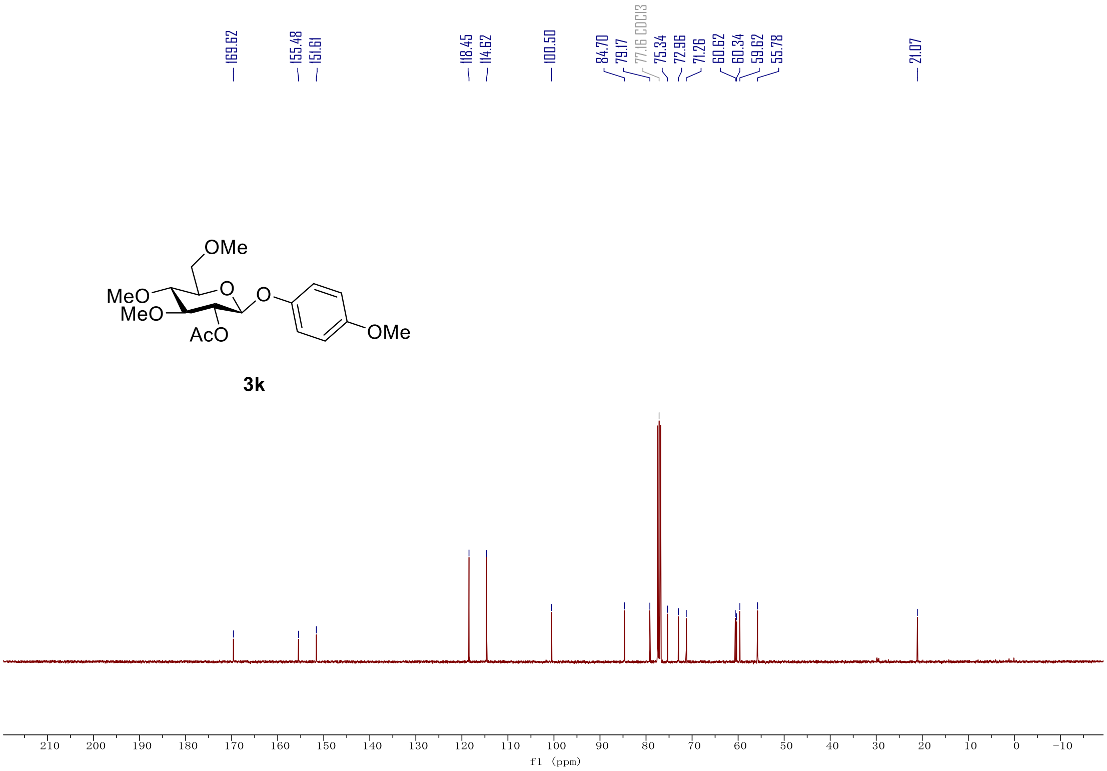

**Supplementary Figure S121. $^{13}$C NMR (101 MHz, $CDCl_3$) Spectra for compound 3k**

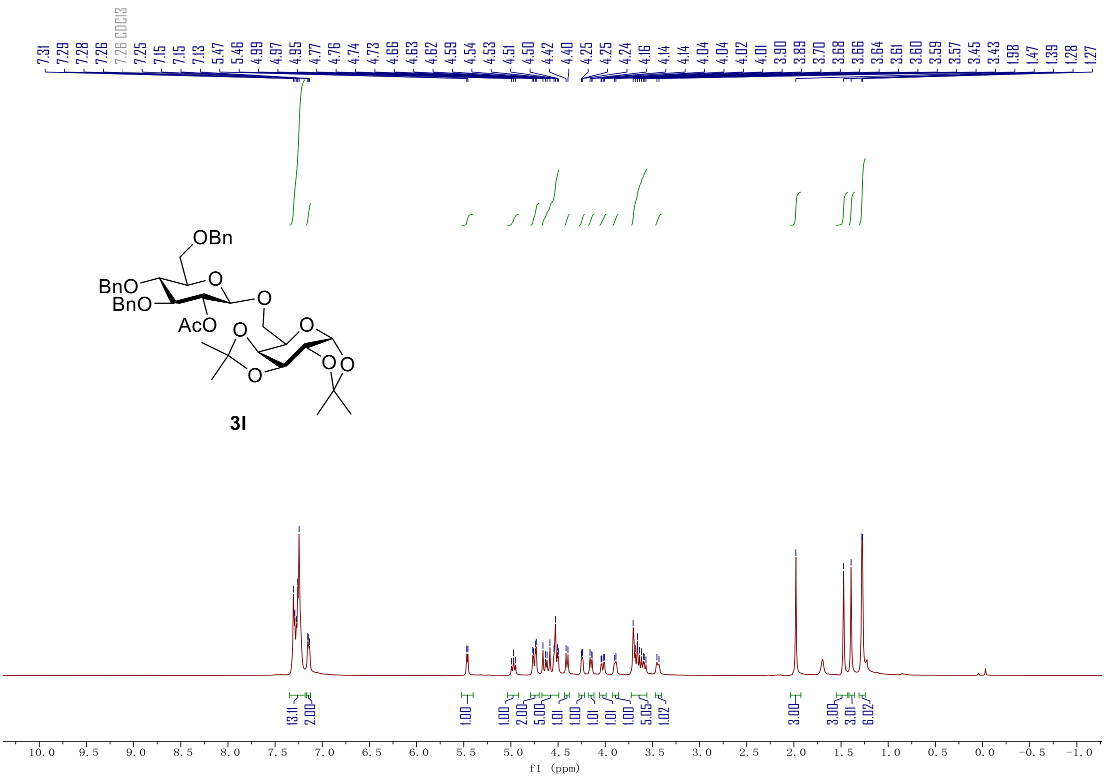

**Supplementary Figure S122.** $^{1}$**H NMR (400 MHz,** $CDCl_3$**) Spectra for compound 3l**

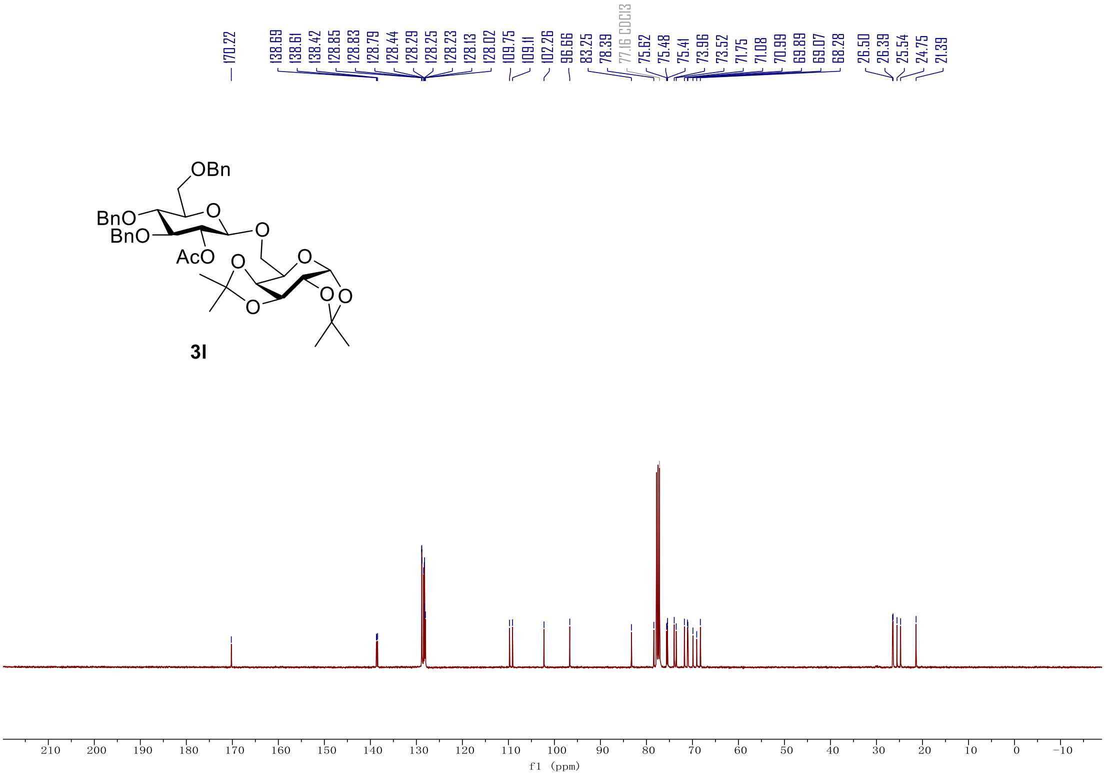

**Supplementary Figure S123.** $^{13}$**C NMR (101 MHz,** $CDCl_3$**) Spectra for compound 3l**

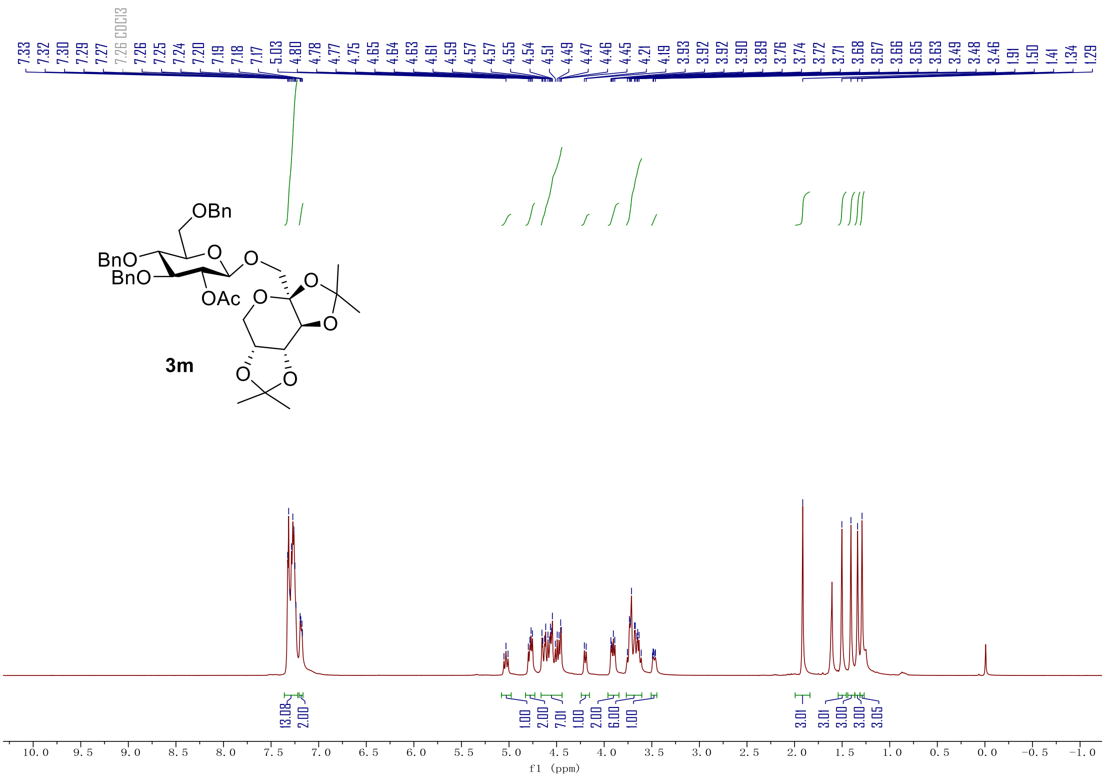


**Supplementary Figure S124.** $^{1}$H NMR (400 MHz, $CDCl_3$) Spectra for compound 3m

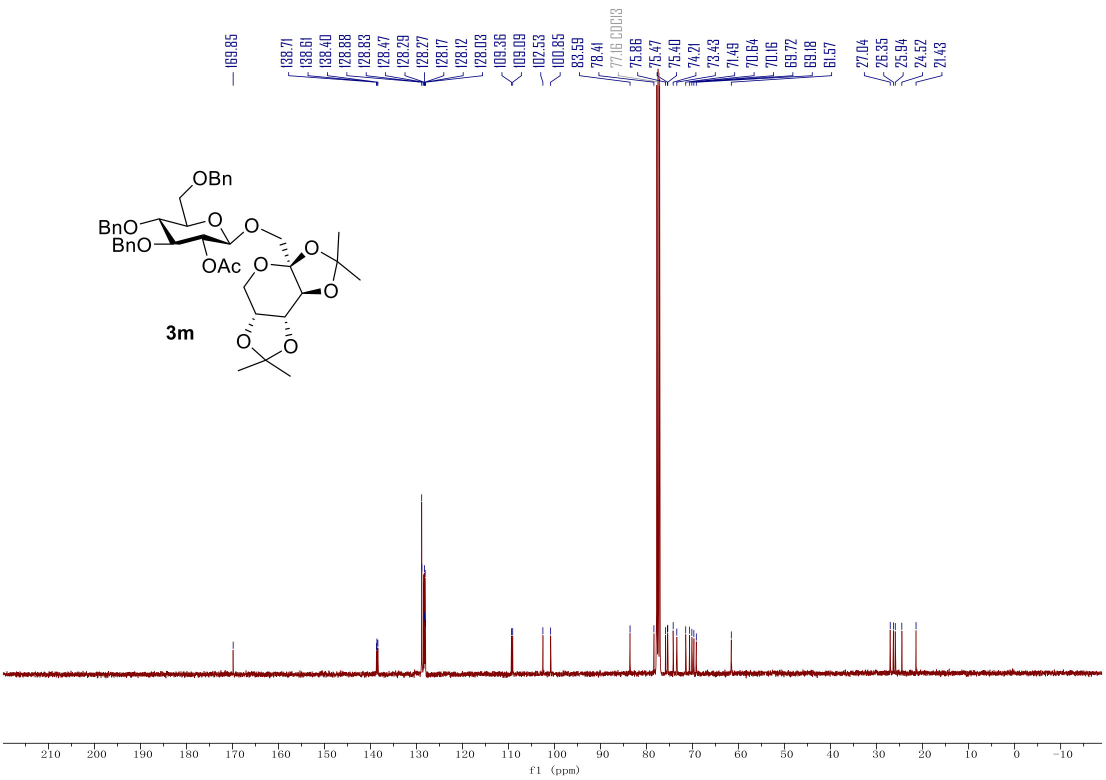


**Supplementary Figure S125.** $^{13}$C NMR (101 MHz, $CDCl_3$) Spectra for compound 3m

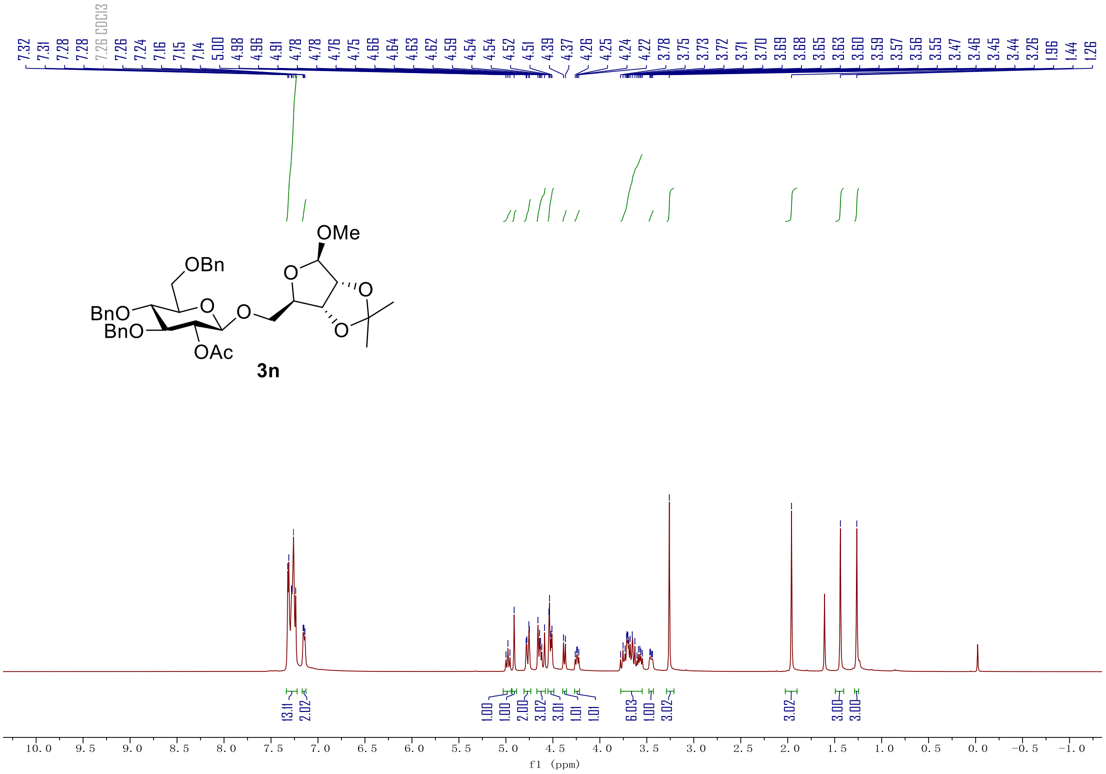


**Supplementary Figure S126. $^{1}$H NMR (400 MHz, $CDCl_3$) Spectra for compound 3n**

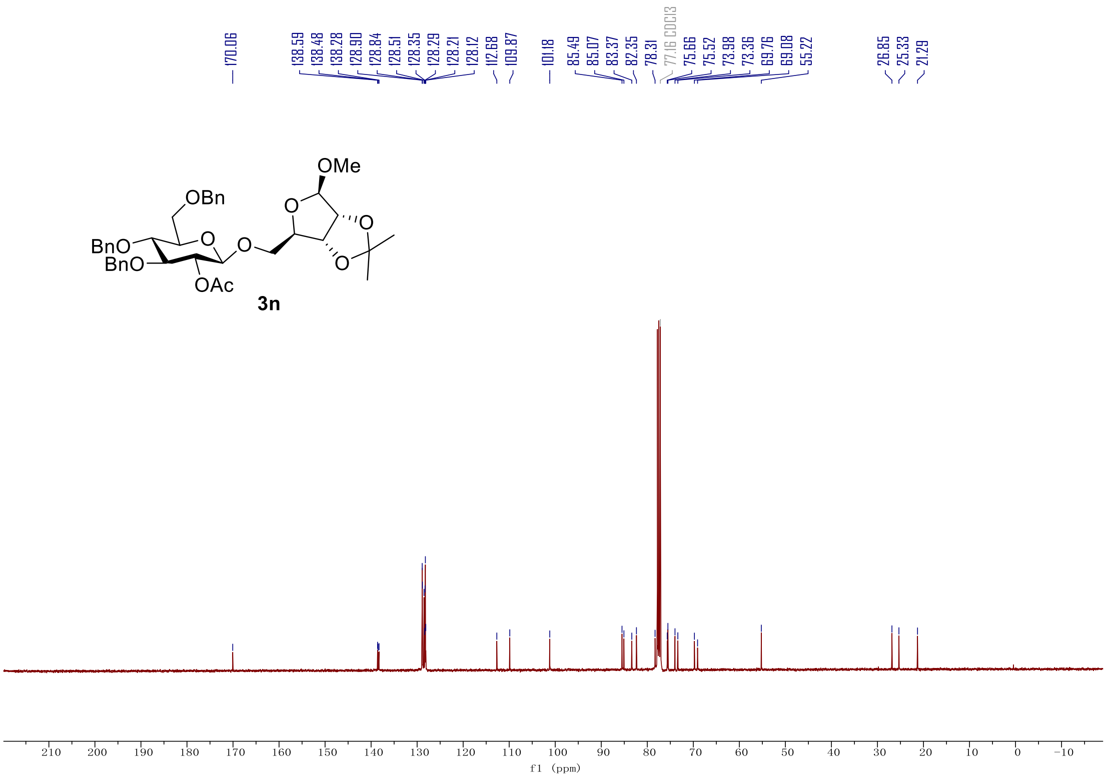


**Supplementary Figure S127. $^{13}$C NMR (101 MHz, $CDCl_3$) Spectra for compound 3n**

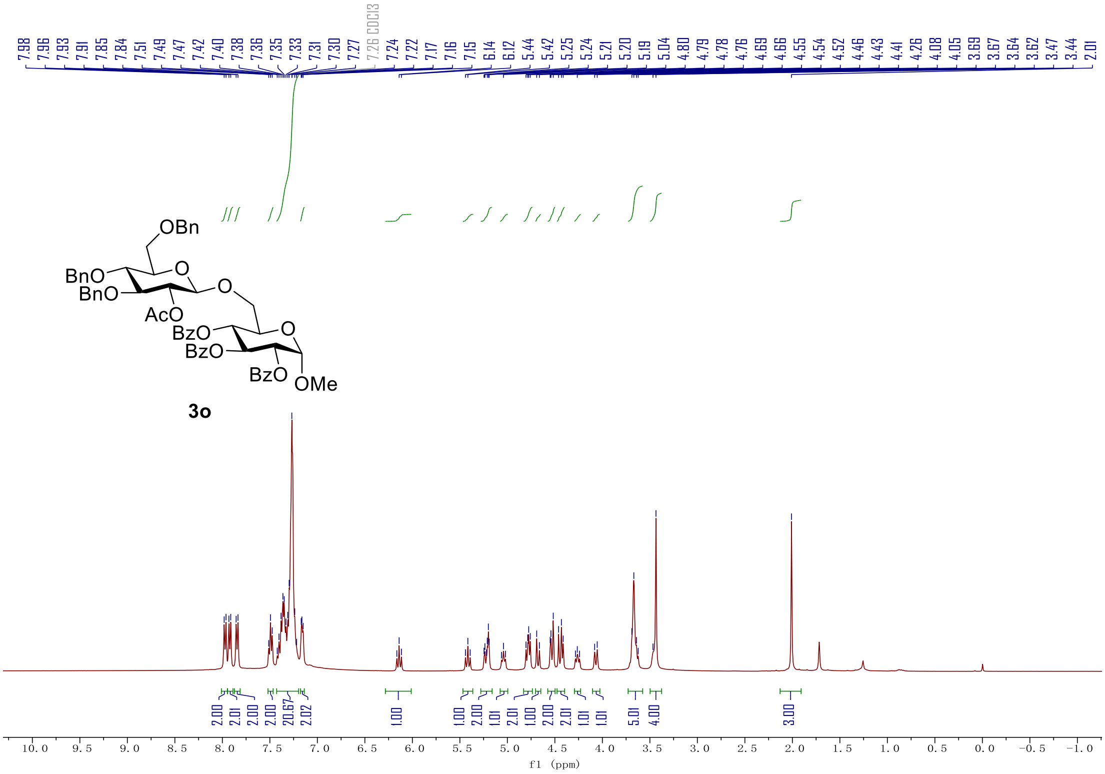


**Supplementary Figure S128. $^{1}H$ NMR (400 MHz, $CDCl_3$) Spectra for compound 3o**

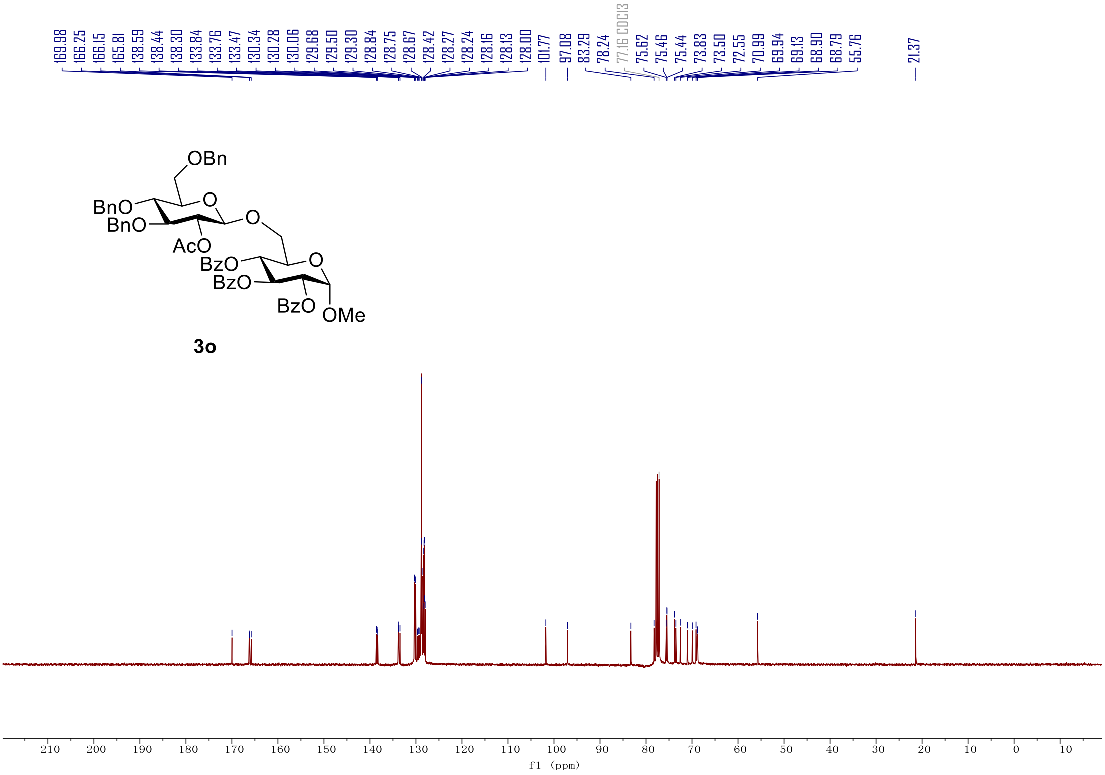


**Supplementary Figure S129. $^{13}C$ NMR (101 MHz, $CDCl_3$) Spectra for compound 3o**

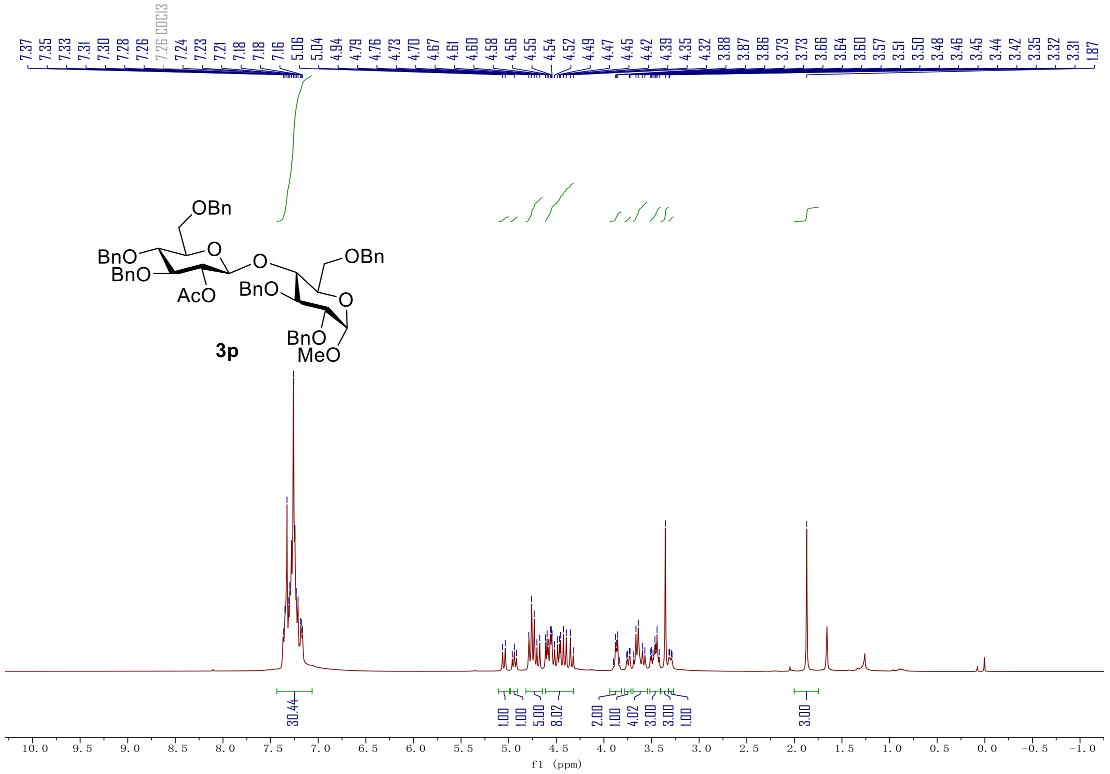

Supplementary Figure S130. $^{1}$H NMR (400 MHz, $CDCl_3$) Spectra for compound 3p

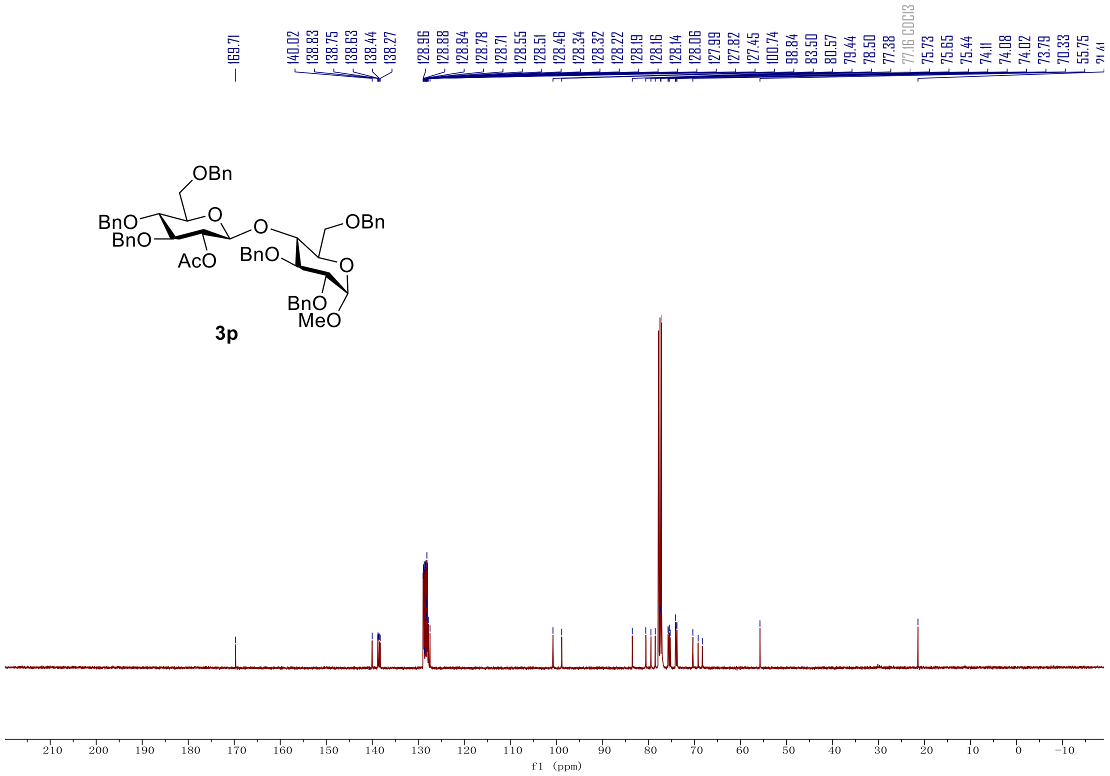

Supplementary Figure S131. $^{13}$C NMR (101 MHz, $CDCl_3$) Spectra for compound 3p

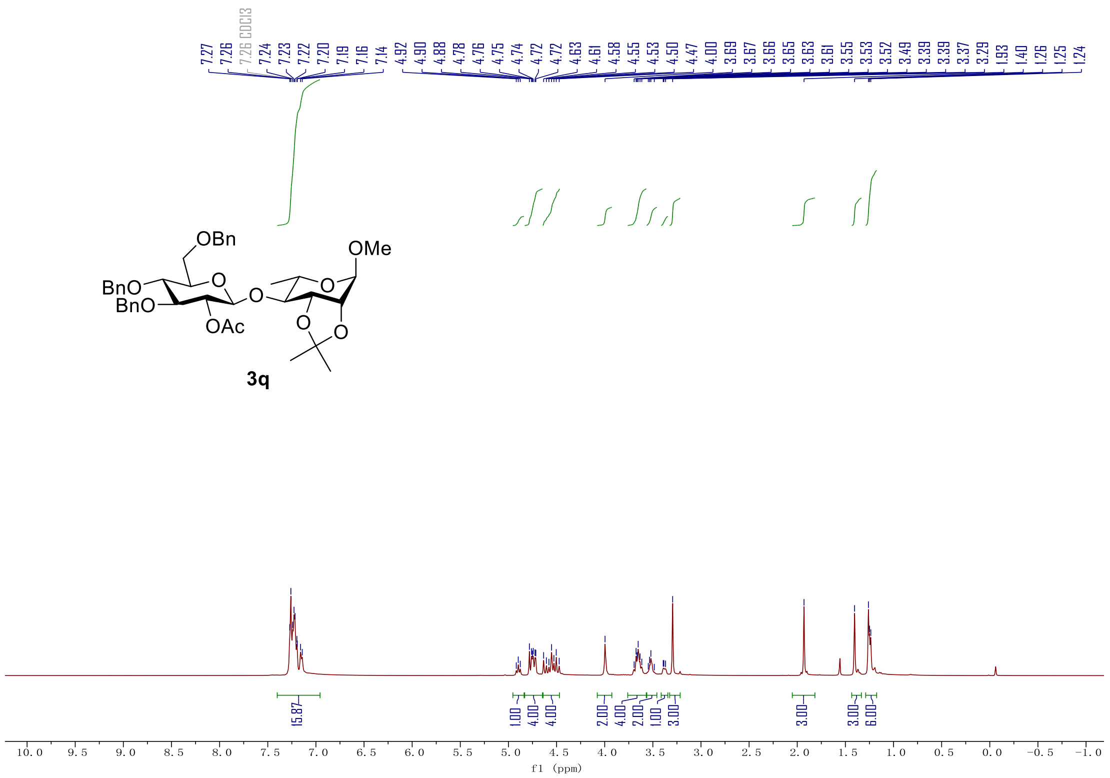

**Supplementary Figure S132. $^{1}H$ NMR (400 MHz, $CDCl_3$) Spectra for compound 3q**

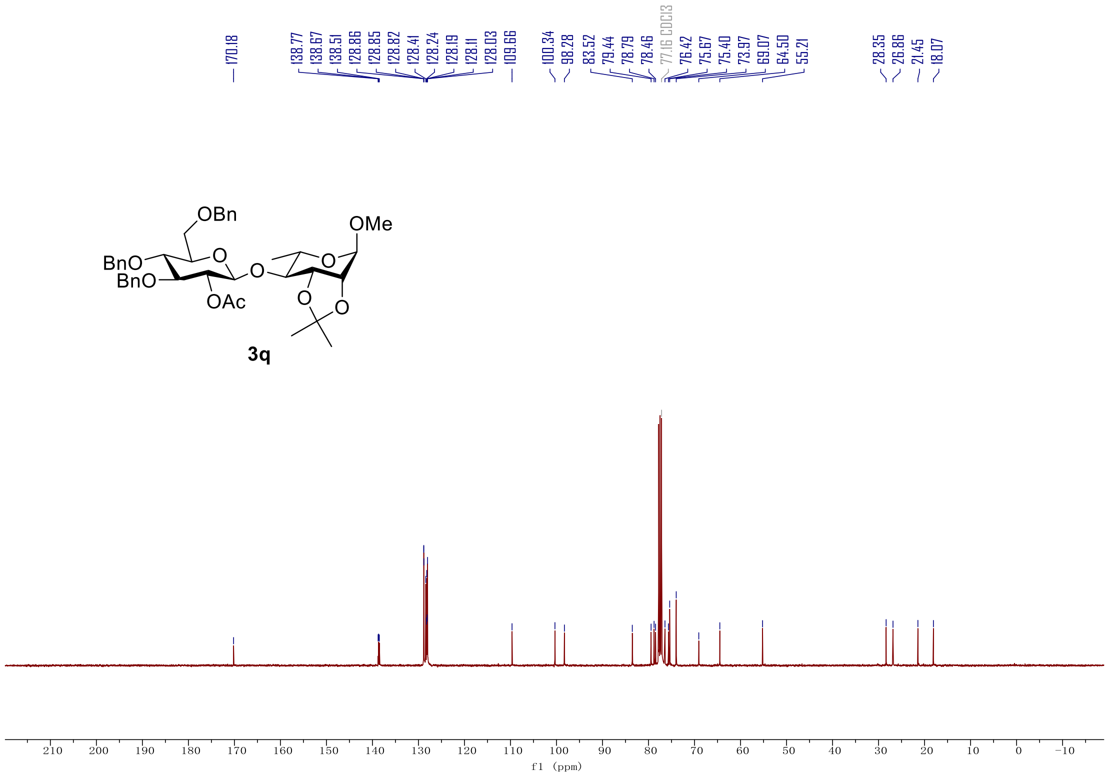

**Supplementary Figure S133. $^{13}C$ NMR (101 MHz, $CDCl_3$) Spectra for compound 3q**

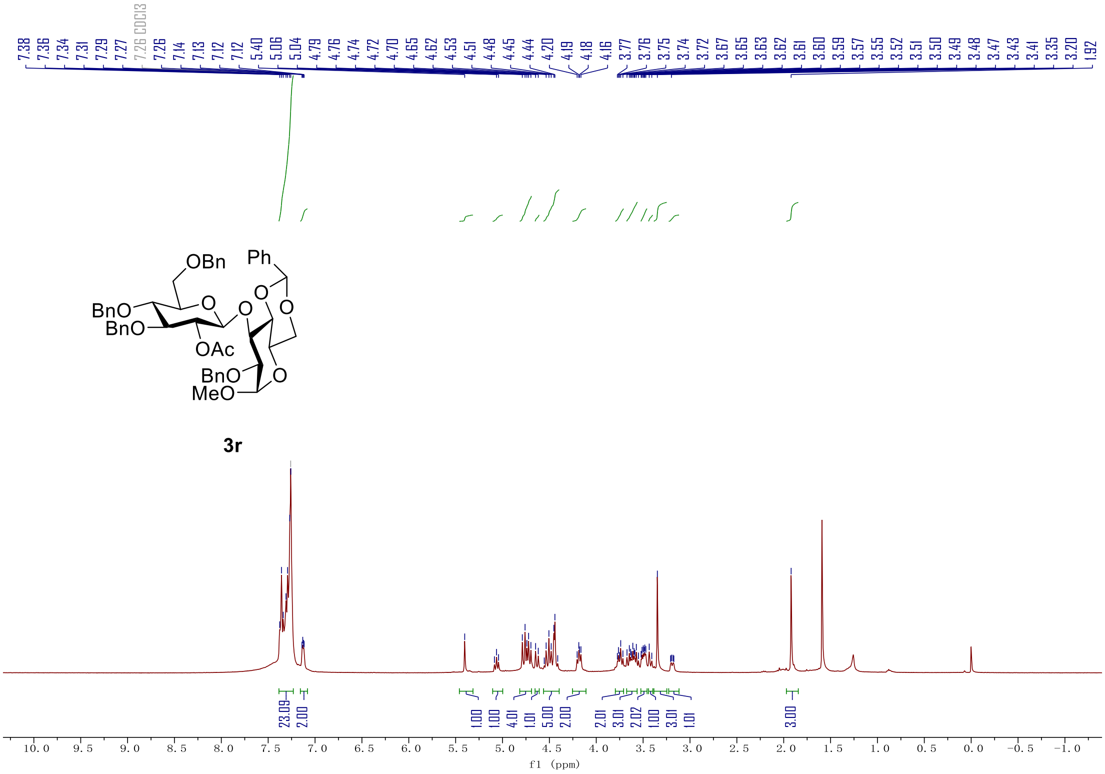


**Supplementary Figure S134. $^{1}$H NMR (400 MHz, $CDCl_3$) Spectra for compound 3r**

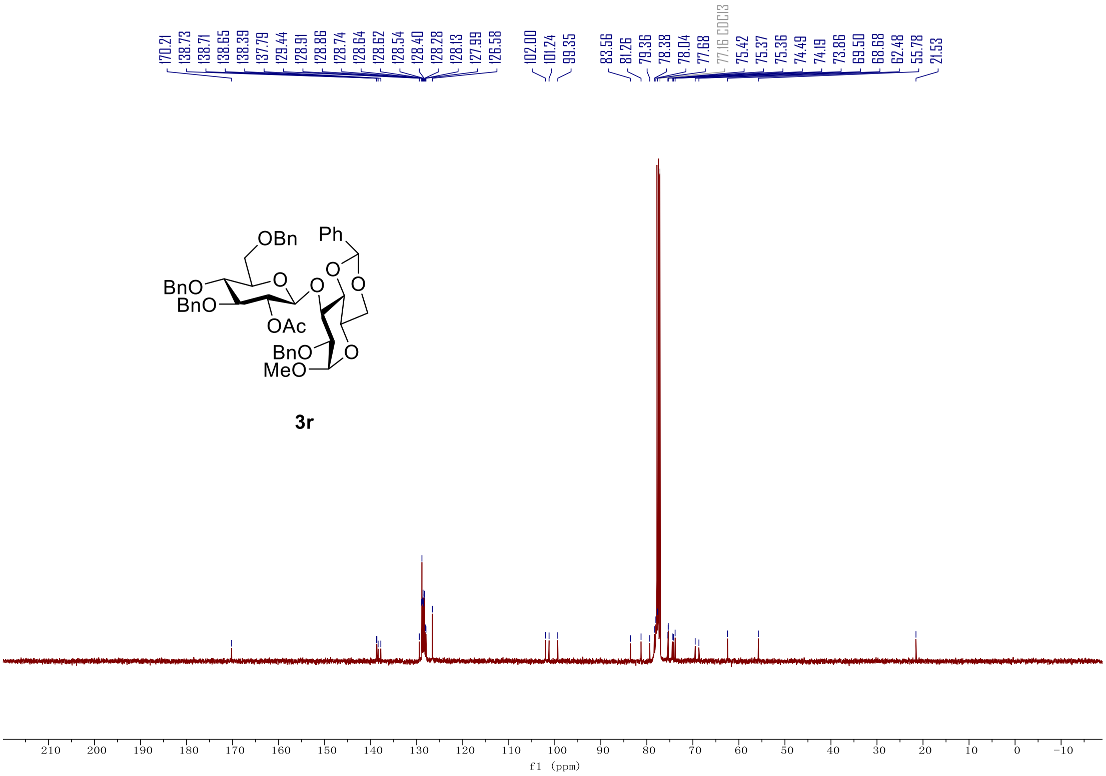


**Supplementary Figure S135. $^{13}$C NMR (101 MHz, $CDCl_3$) Spectra for compound 3r**

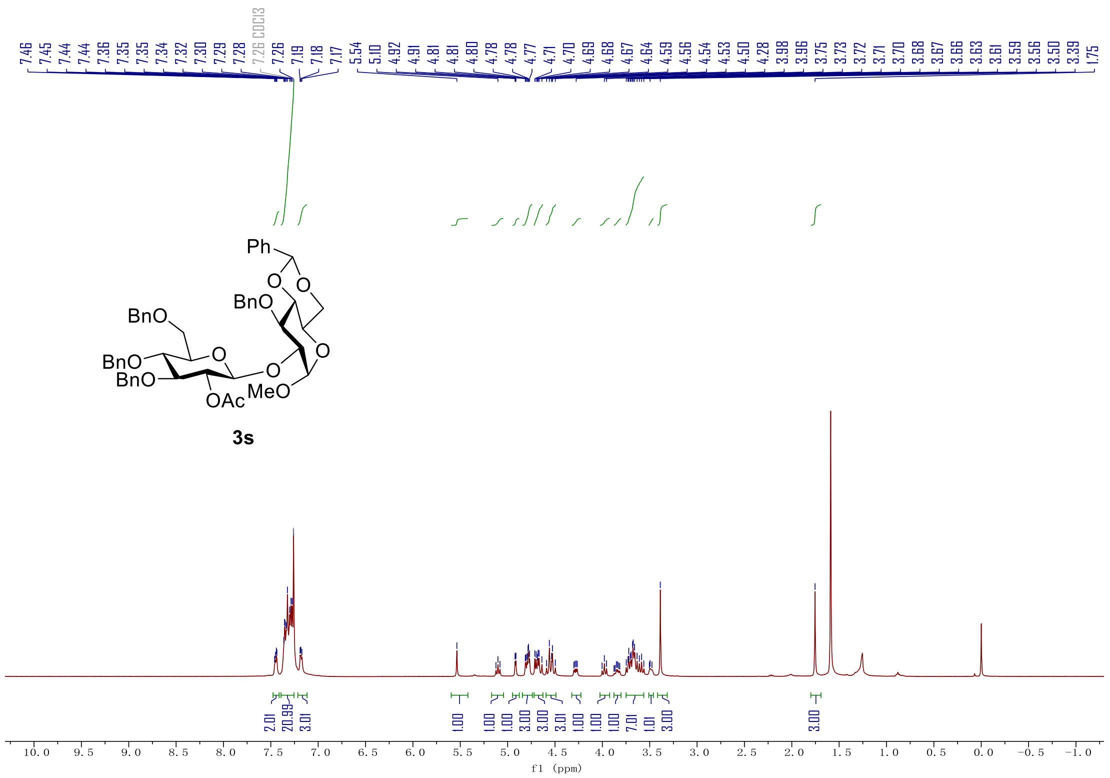


**Supplementary Figure S136. $^{1}$H NMR (400 MHz, $CDCl_3$) Spectra for compound 3s**

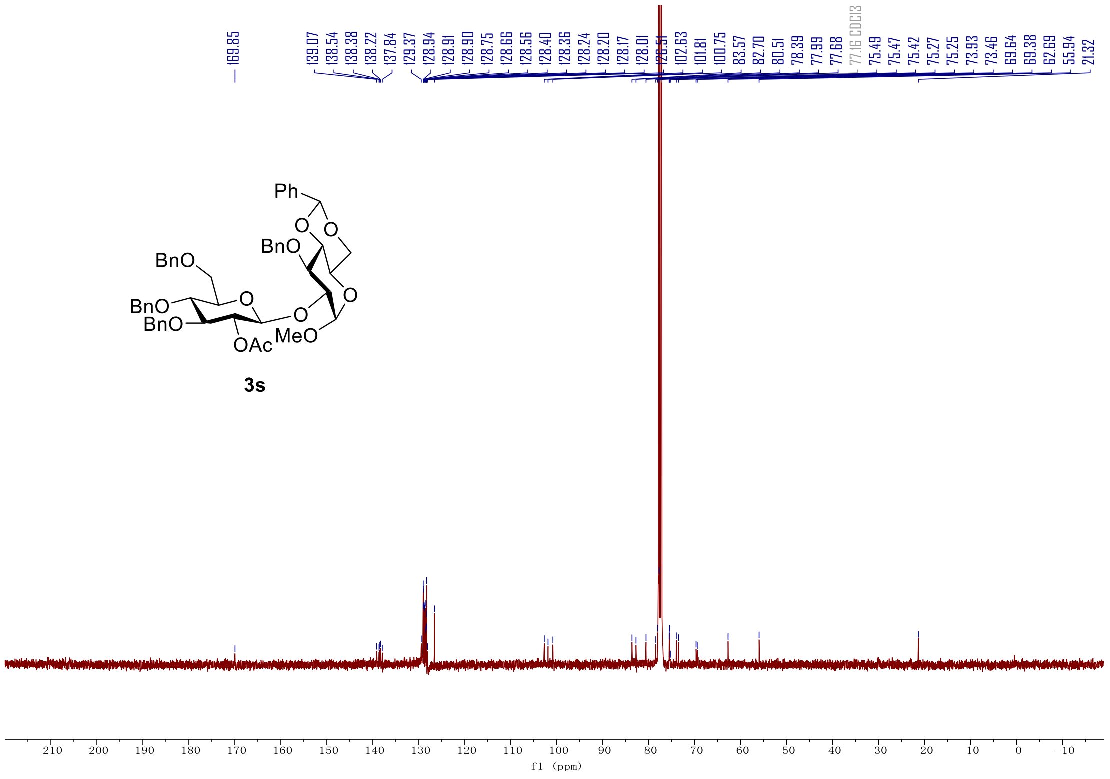


**Supplementary Figure S137. $^{13}$C NMR (101 MHz, $CDCl_3$) Spectra for compound 3s**

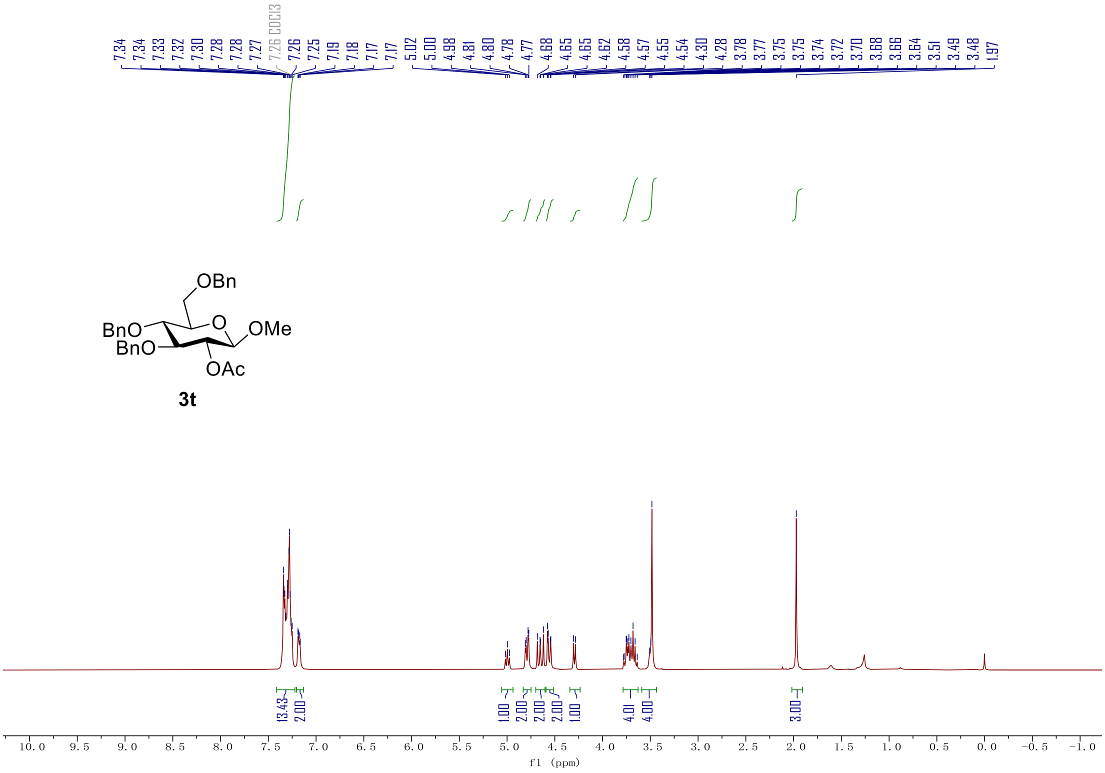

**Supplementary Figure S138. $^{1}$H NMR (400 MHz, $CDCl_3$) Spectra for compound 3t**

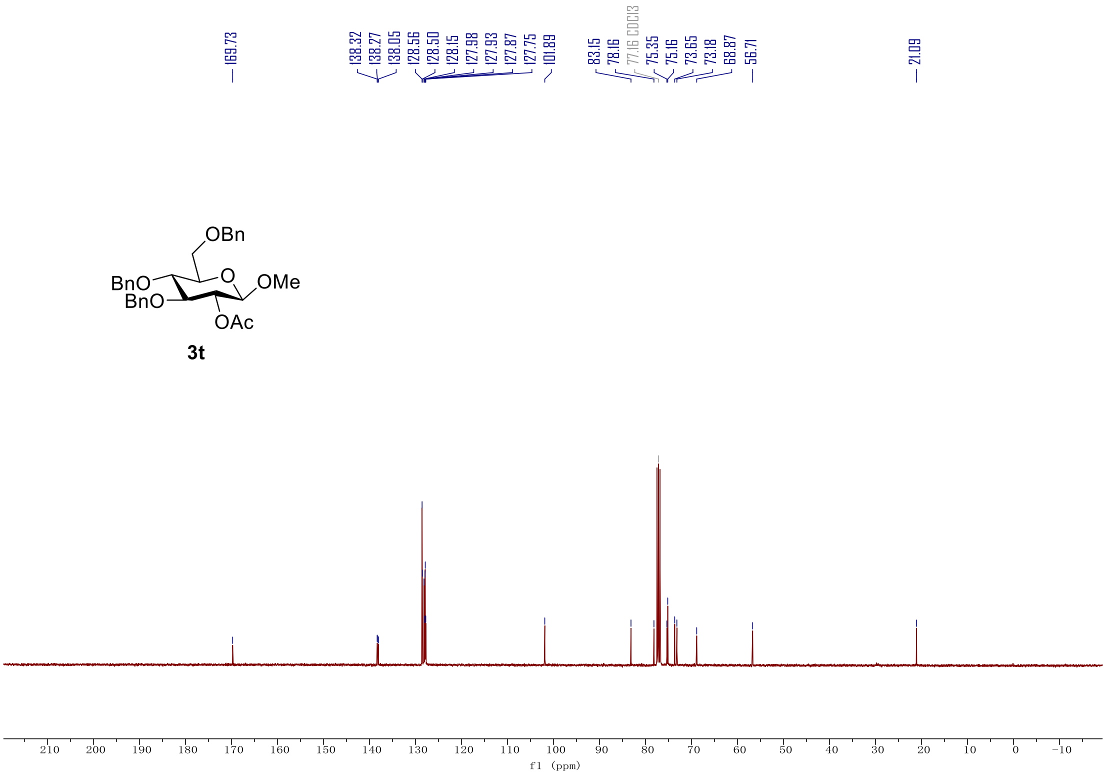

**Supplementary Figure S139. $^{13}$C NMR (101 MHz, $CDCl_3$) Spectra for compound 3t**

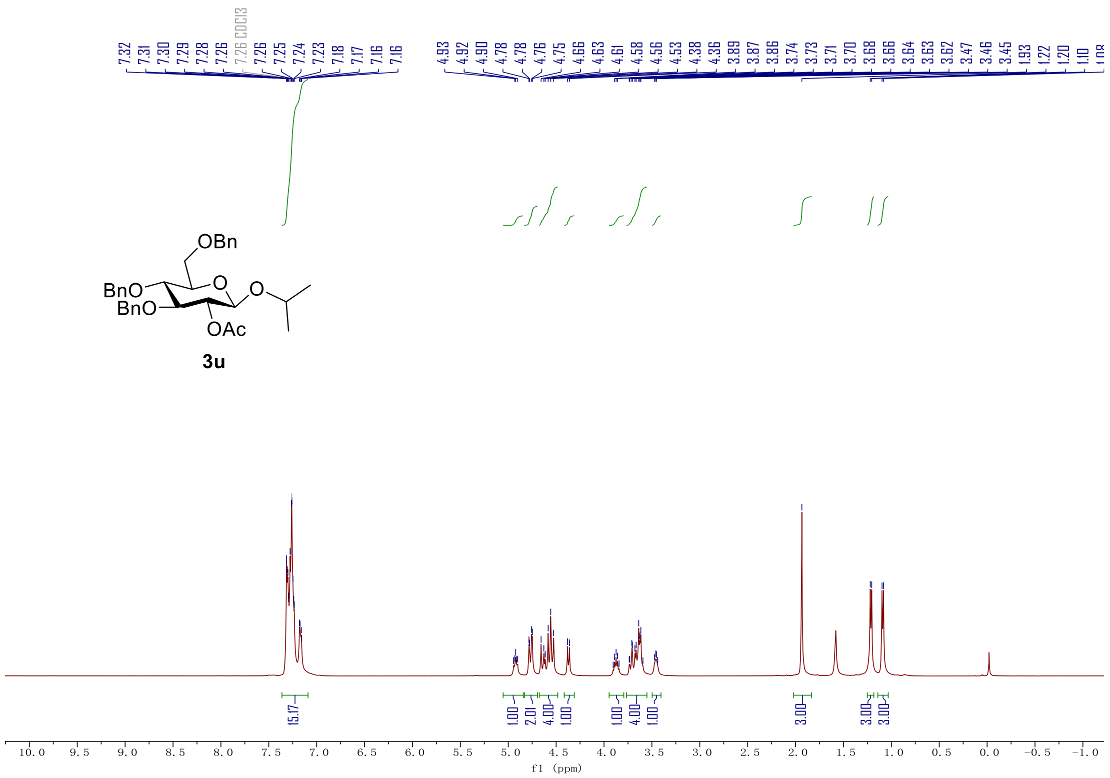

**Supplementary Figure S140. $^{1}H$ NMR (400 MHz, $CDCl_3$) Spectra for compound 3u**

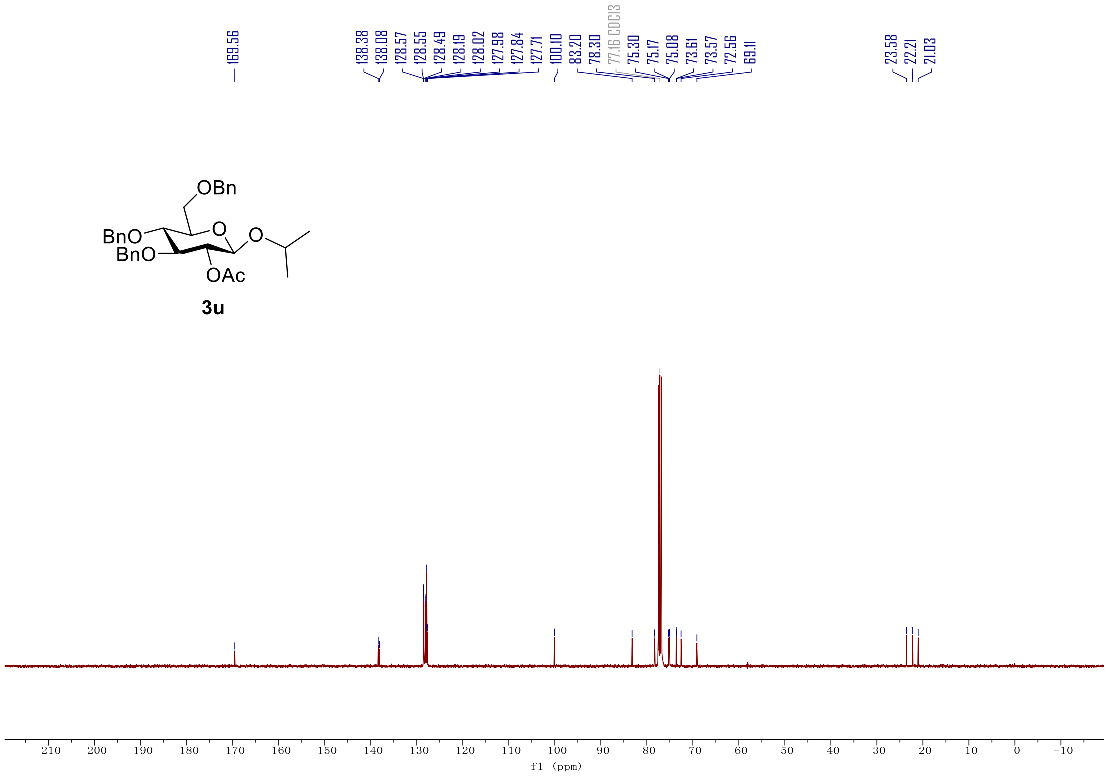

**Supplementary Figure S141. $^{13}C$ NMR (101 MHz, $CDCl_3$) Spectra for compound 3u**

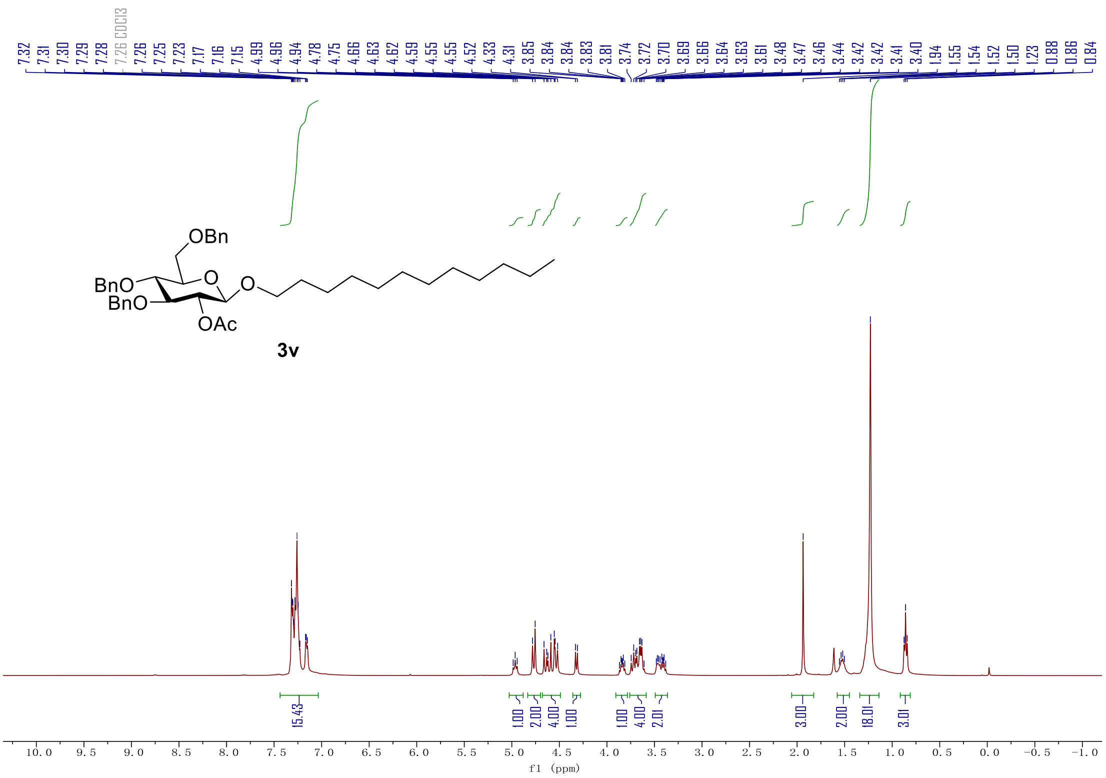


**Supplementary Figure S142. $^{1}$H NMR (400 MHz, $CDCl_3$) Spectra for compound 3v**

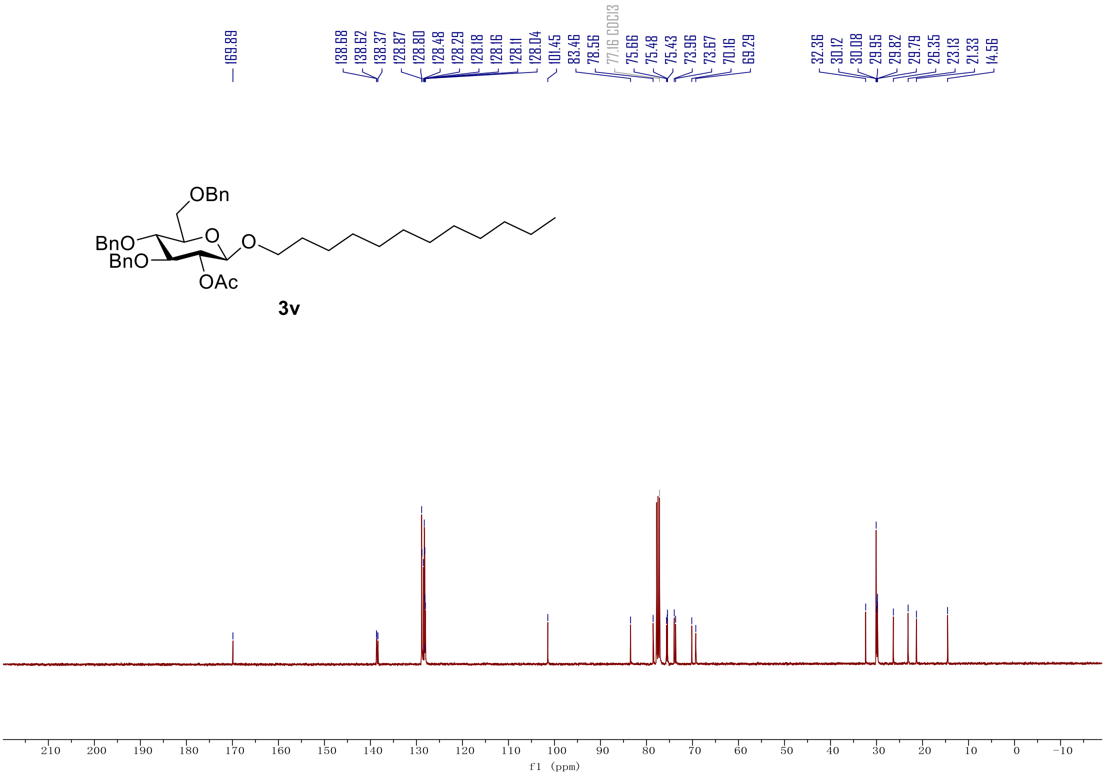


**Supplementary Figure S143. $^{13}$C NMR (101 MHz, $CDCl_3$) Spectra for compound 3v**

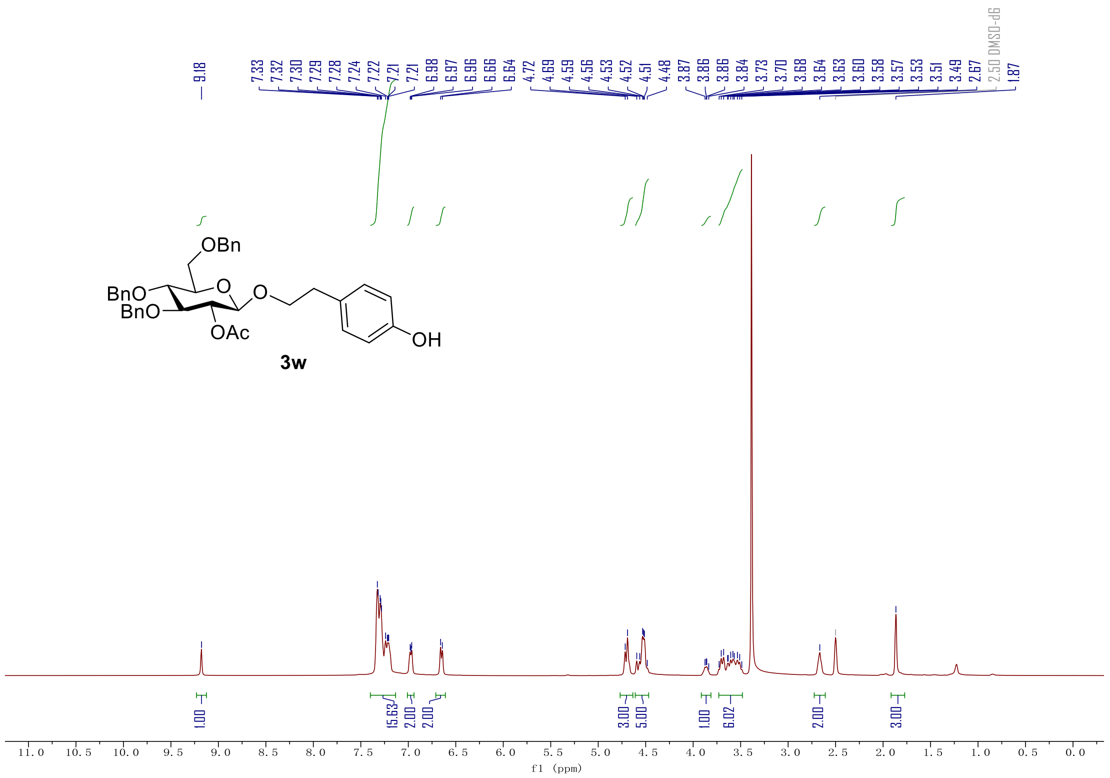


**Supplementary Figure S144. $^{1}$H NMR (400 MHz, DMSO-*d*6) Spectra for compound 3w**

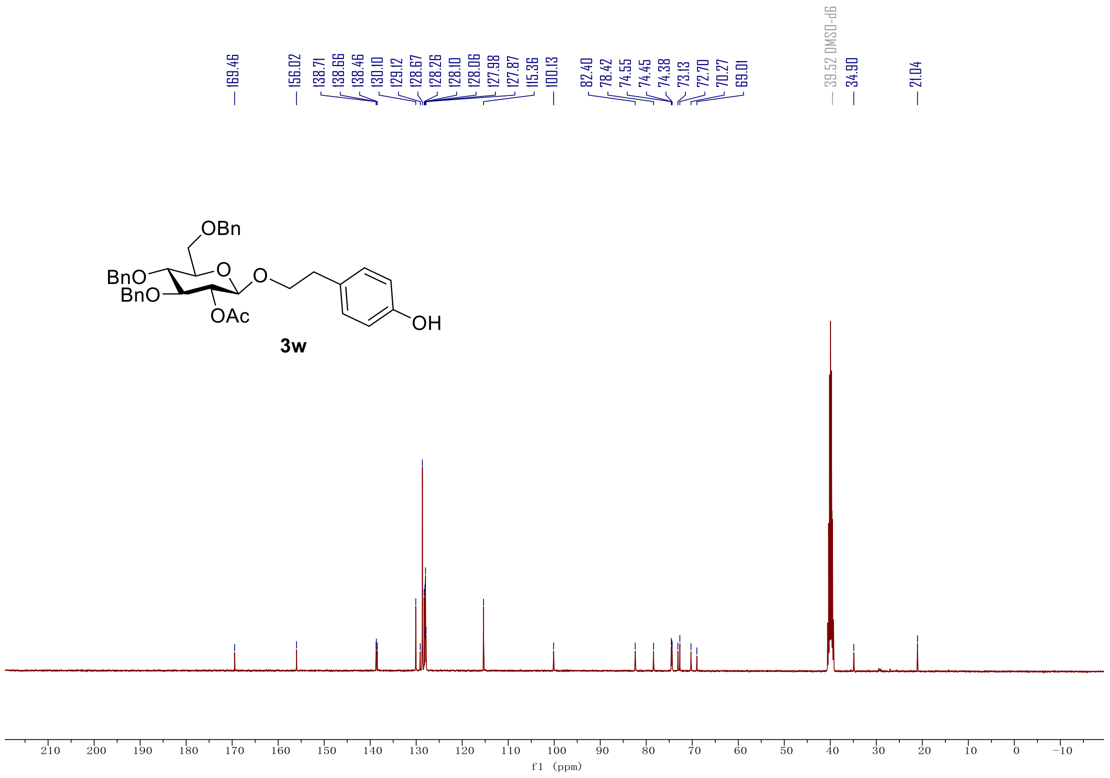


**Supplementary Figure S145. $^{13}$C NMR (101 MHz, DMSO-*d*6) Spectra for compound 3w**

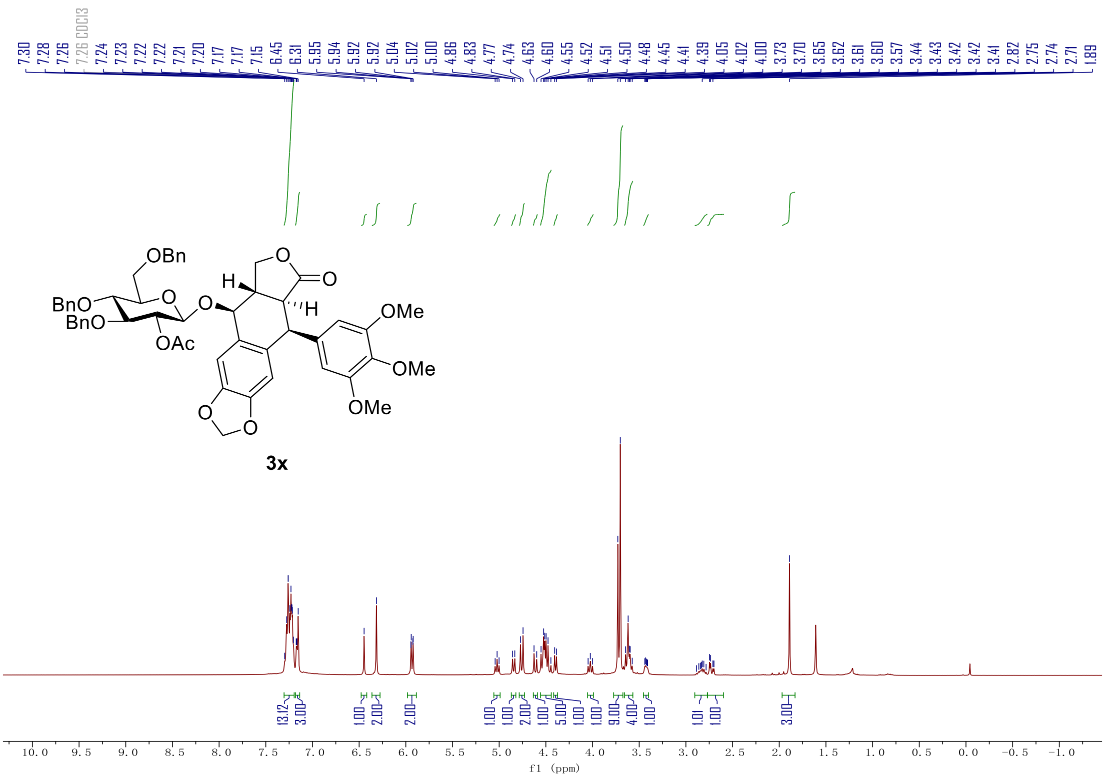


**Supplementary Figure S146. $^{1}$H NMR (400 MHz, $CDCl_3$) Spectra for compound 3x**

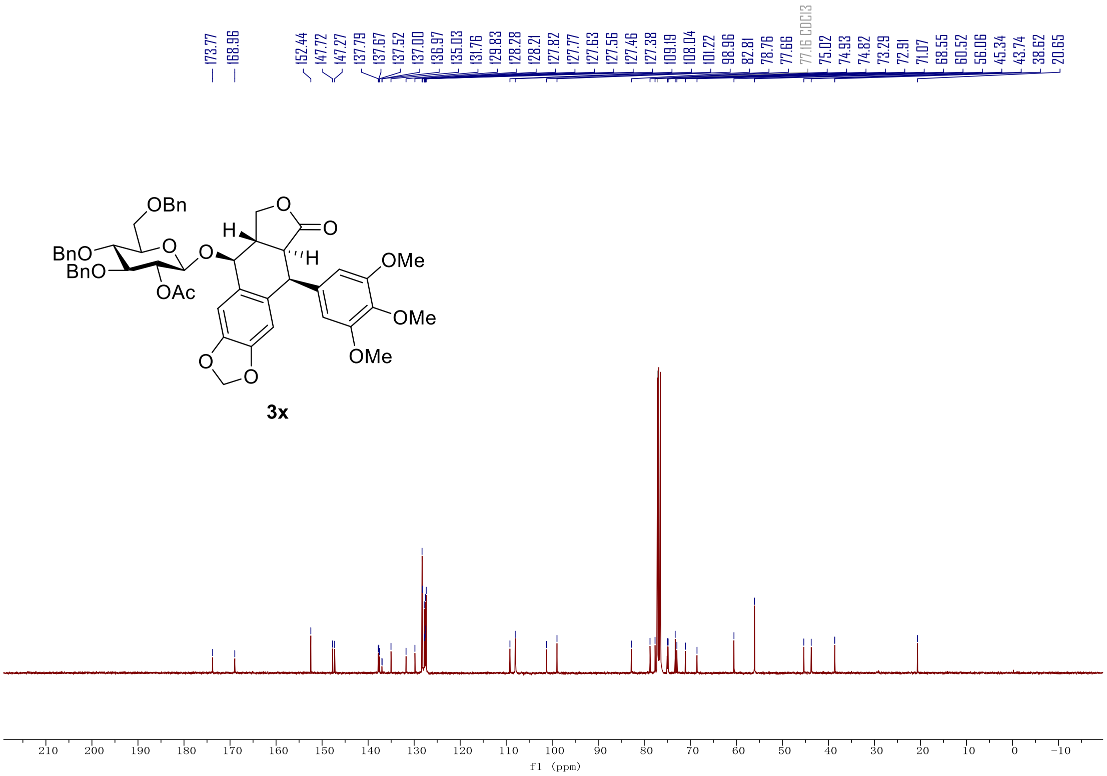


**Supplementary Figure S147. $^{13}$C NMR (101 MHz, $CDCl_3$) Spectra for compound 3x**

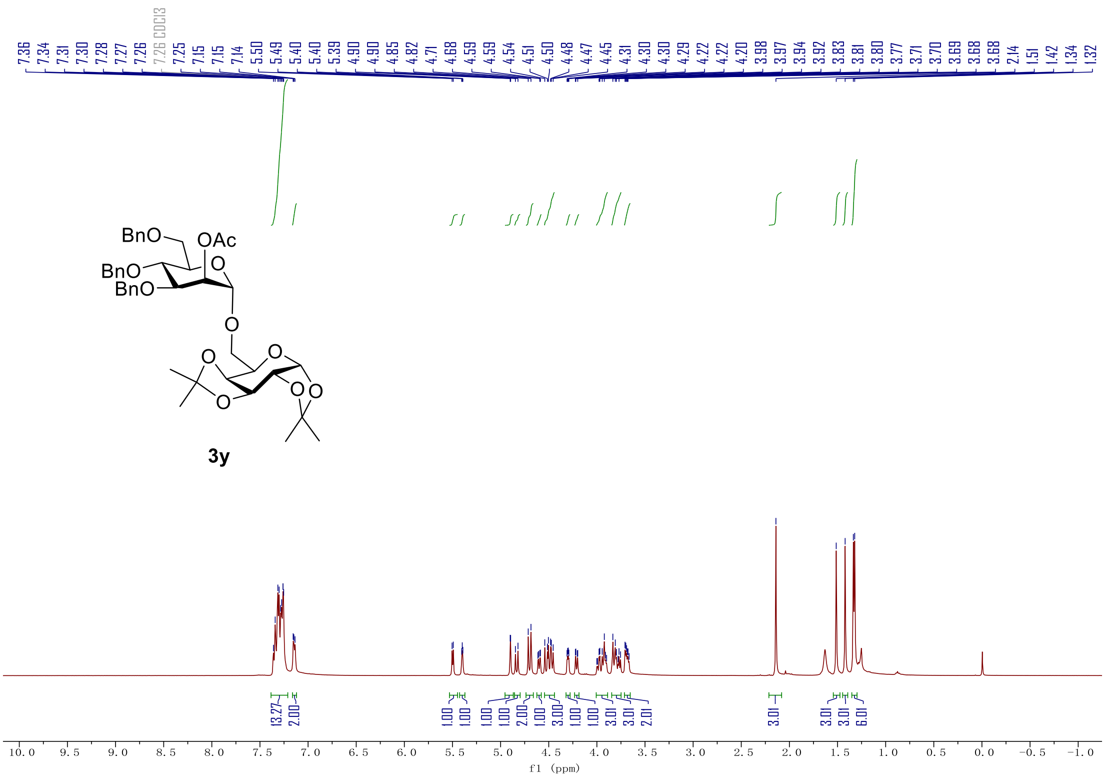

**Supplementary Figure S148. ¹H NMR (400 MHz, $CDCl_3$) Spectra for compound 3y**

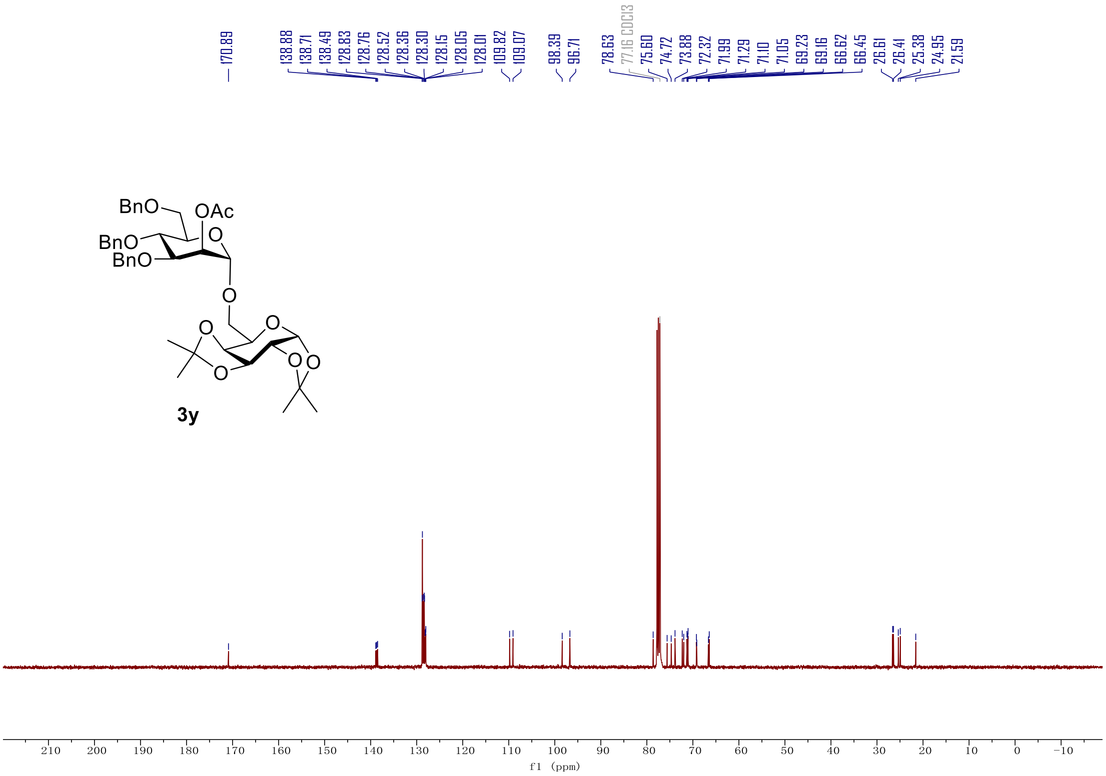

**Supplementary Figure S149. ¹³C NMR (101 MHz, $CDCl_3$) Spectra for compound 3y**

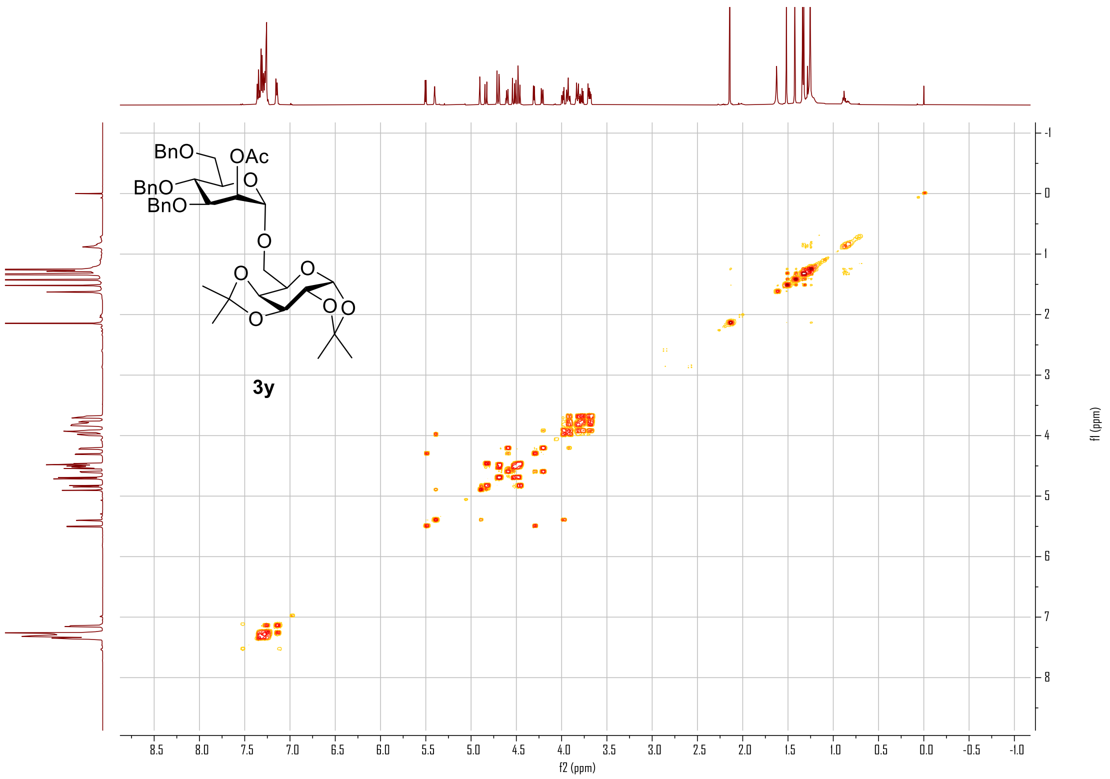


**Supplementary Figure S150. COSY (500 MHz, $CDCl_3$) Spectrum for compound 3y**

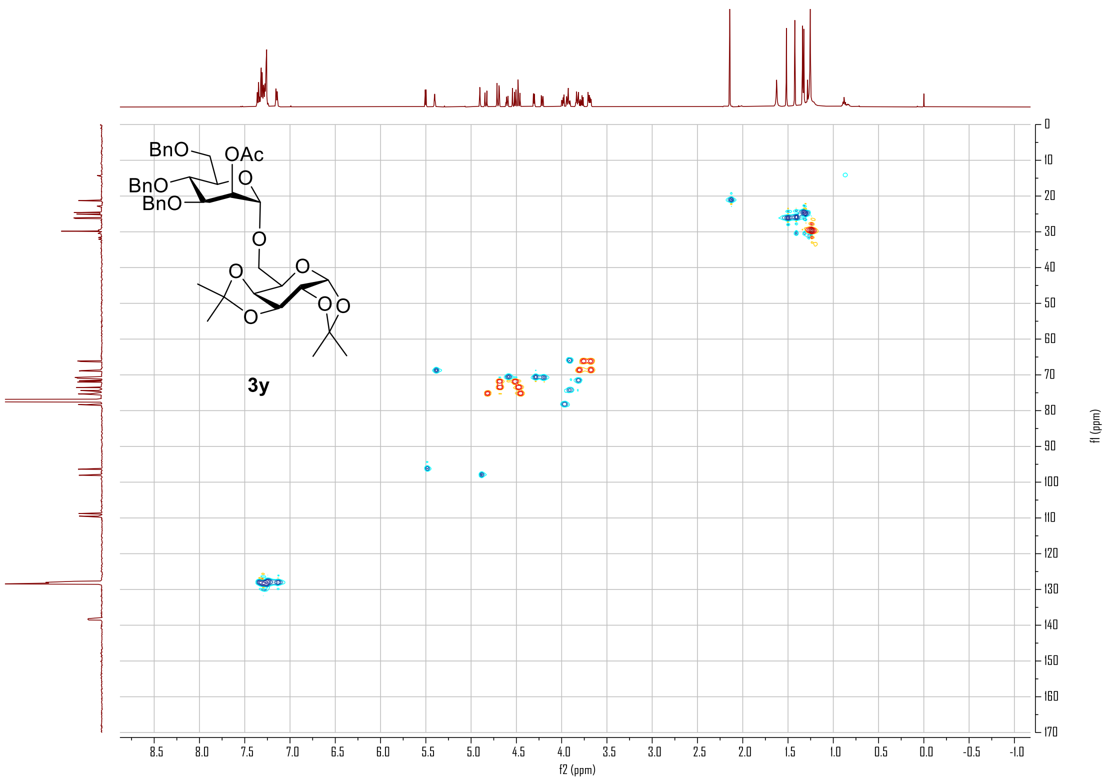


**Supplementary Figure S151. HSQC (500 MHz, $CDCl_3$) Spectrum for compound 3y**

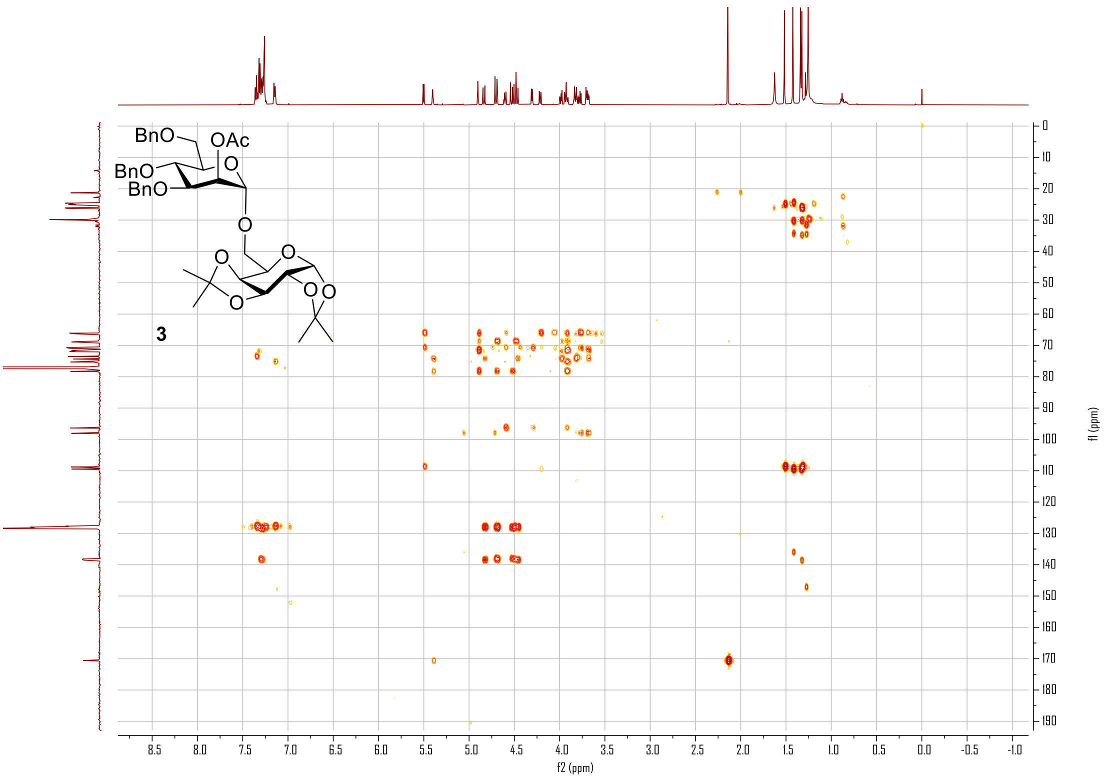


**Supplementary Figure S152. HMBC (500 MHz, CDCl₃) Spectrum for compound 3y**

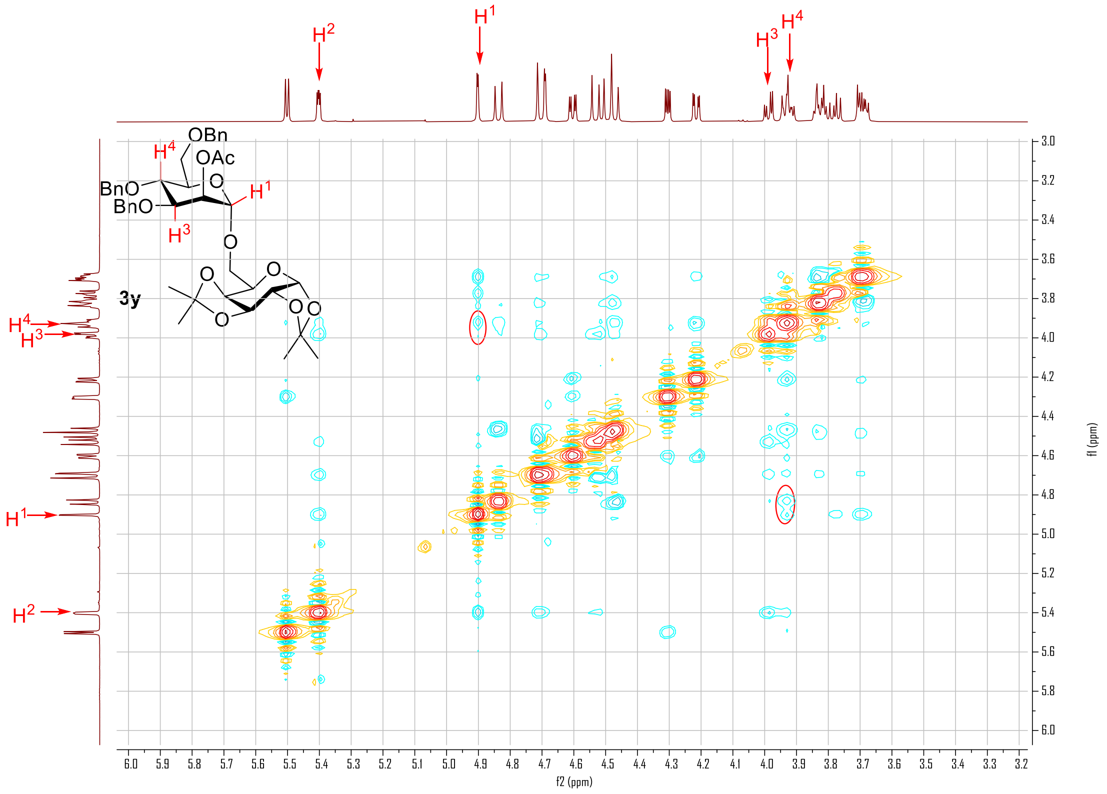


**Supplementary Figure S153. NOESY (500 MHz, CDCl₃) Spectrum for compound 3y**

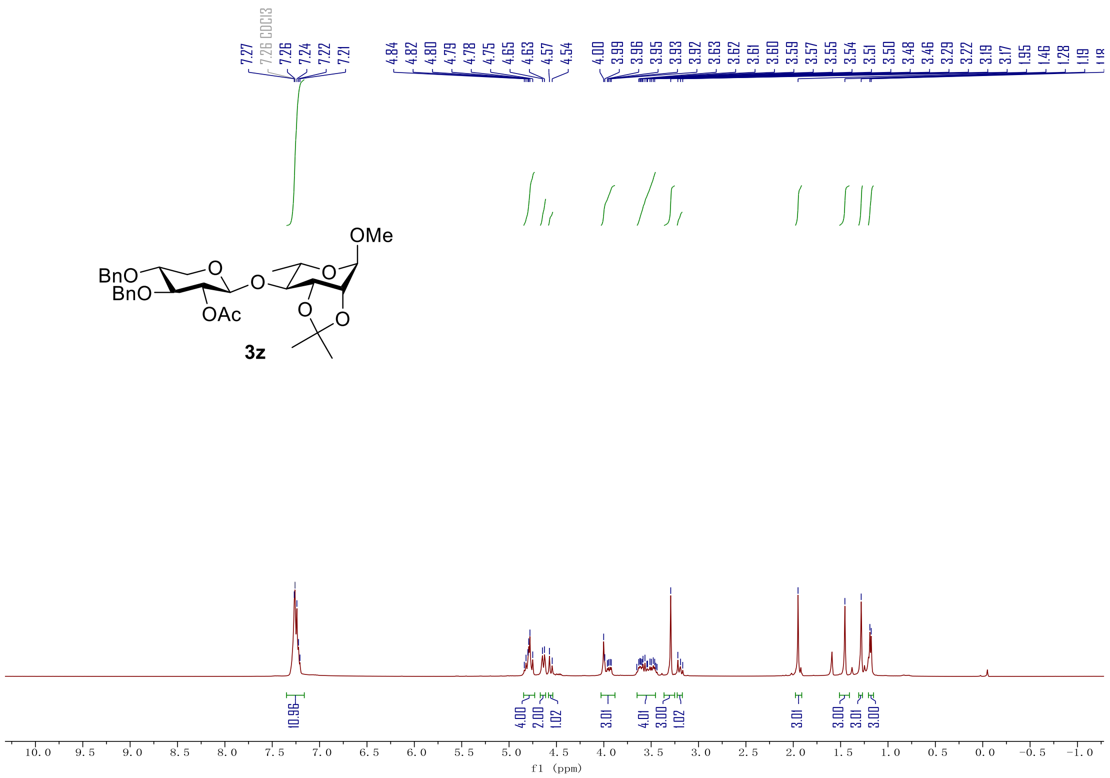

Supplementary Figure S154. Figure S. $^{1}$H NMR (400 MHz, $CDCl_3$) Spectra for compound 3z

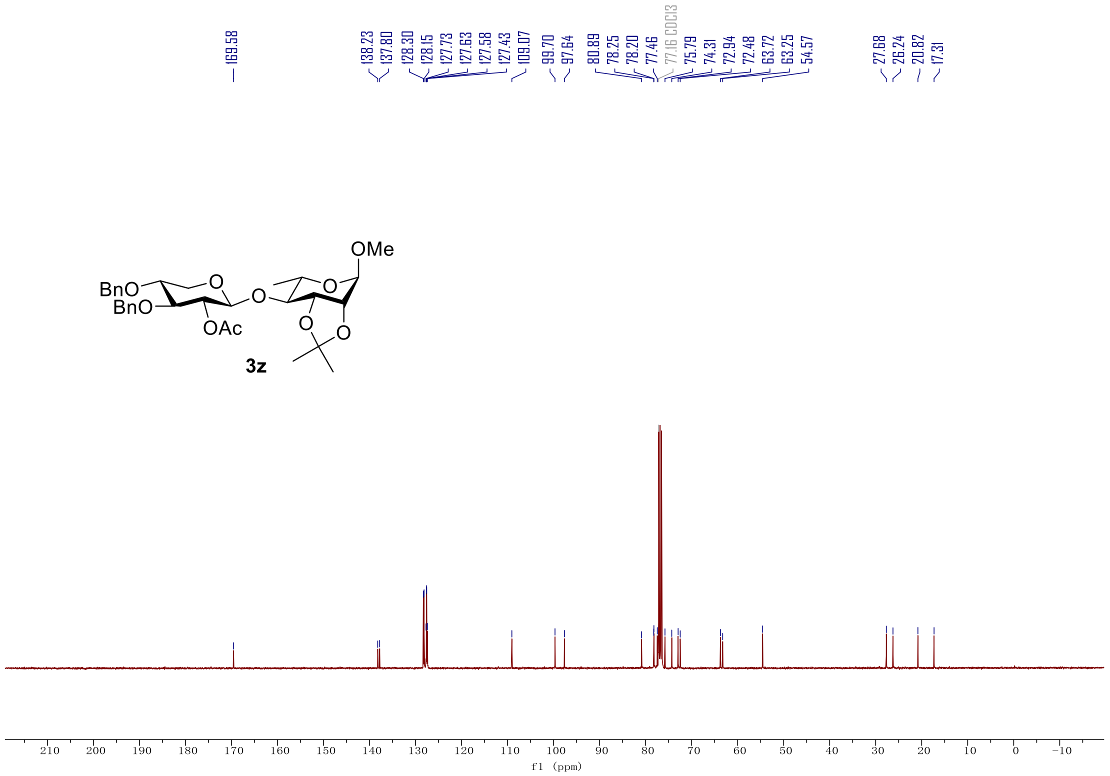

Supplementary Figure S155. $^{13}$C NMR (101 MHz, $CDCl_3$) Spectra for compound 3z

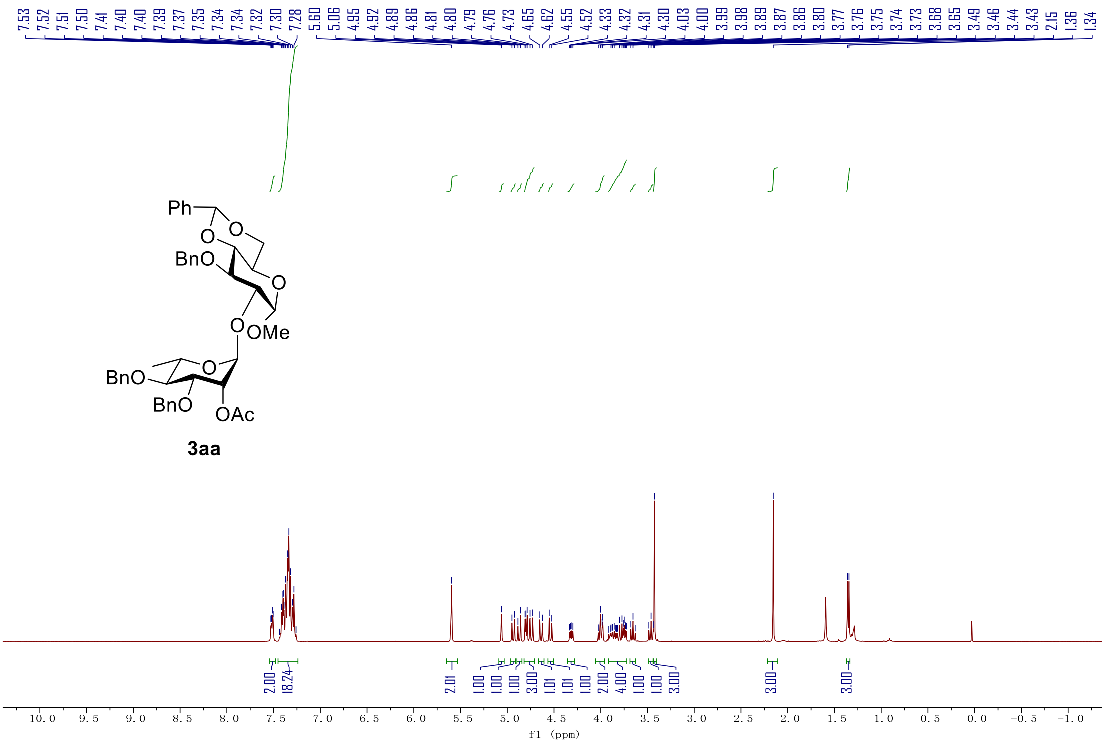

Supplementary Figure S156. ¹H NMR (400 MHz, CDCl₃) Spectra for compound 3aa

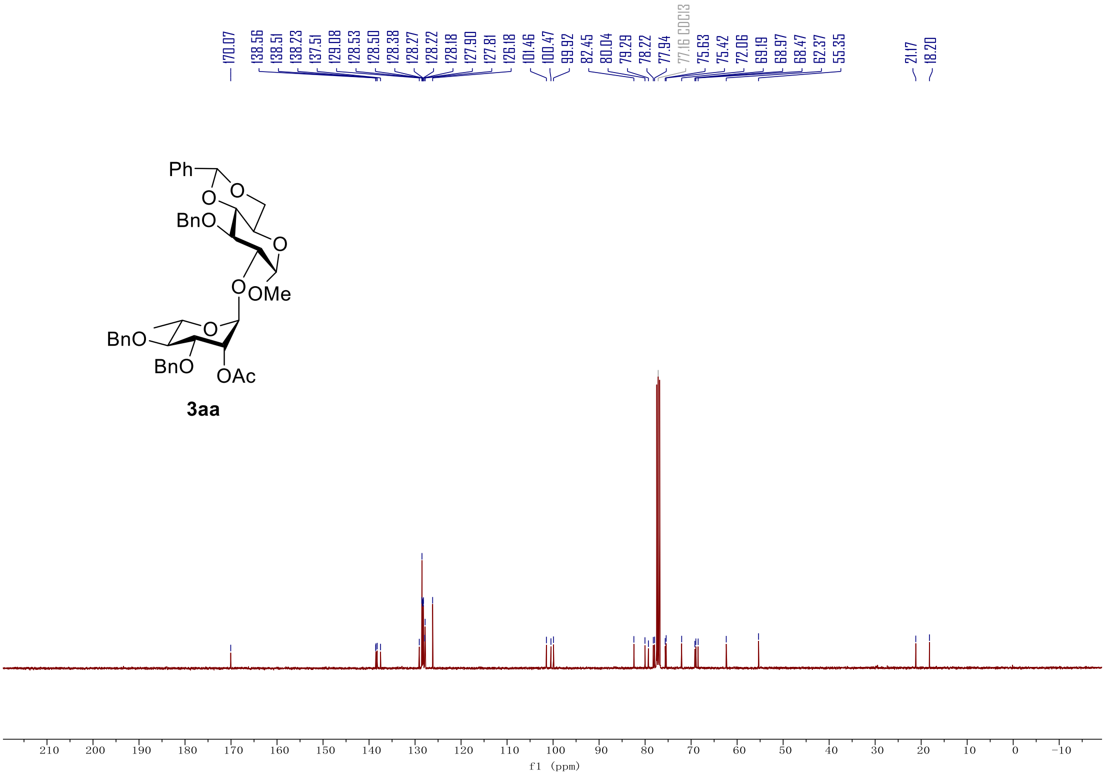

Supplementary Figure S157. ¹³C NMR (101 MHz, CDCl₃) Spectra for compound 3aa

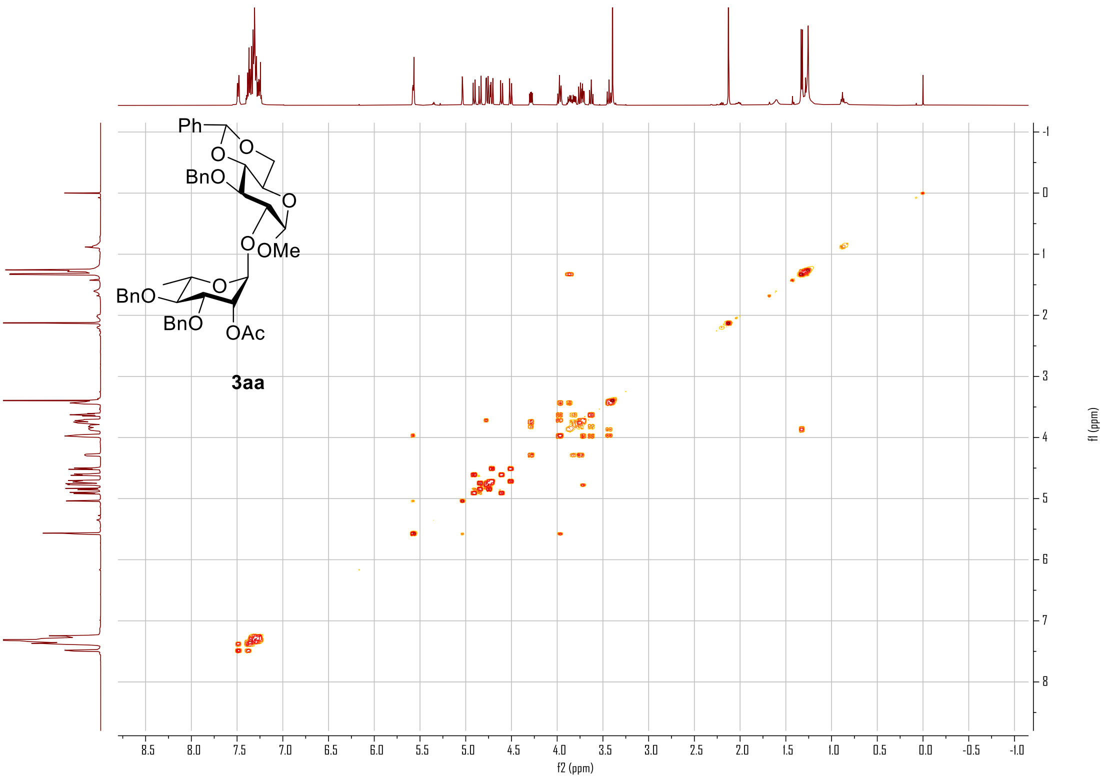


**Supplementary Figure S158. COSY (500 MHz, $CDCl_3$) Spectrum for compound 3aa**

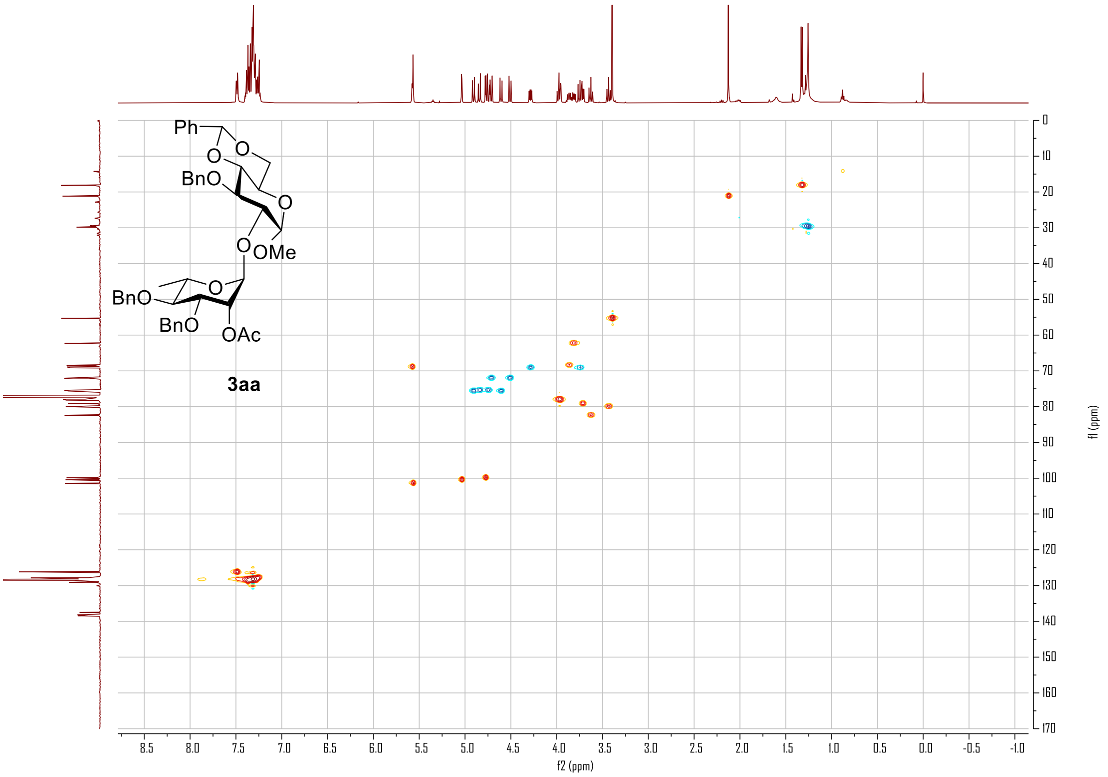


**Supplementary Figure S159. HSQC (500 MHz, $CDCl_3$) Spectrum for compound 3aa**

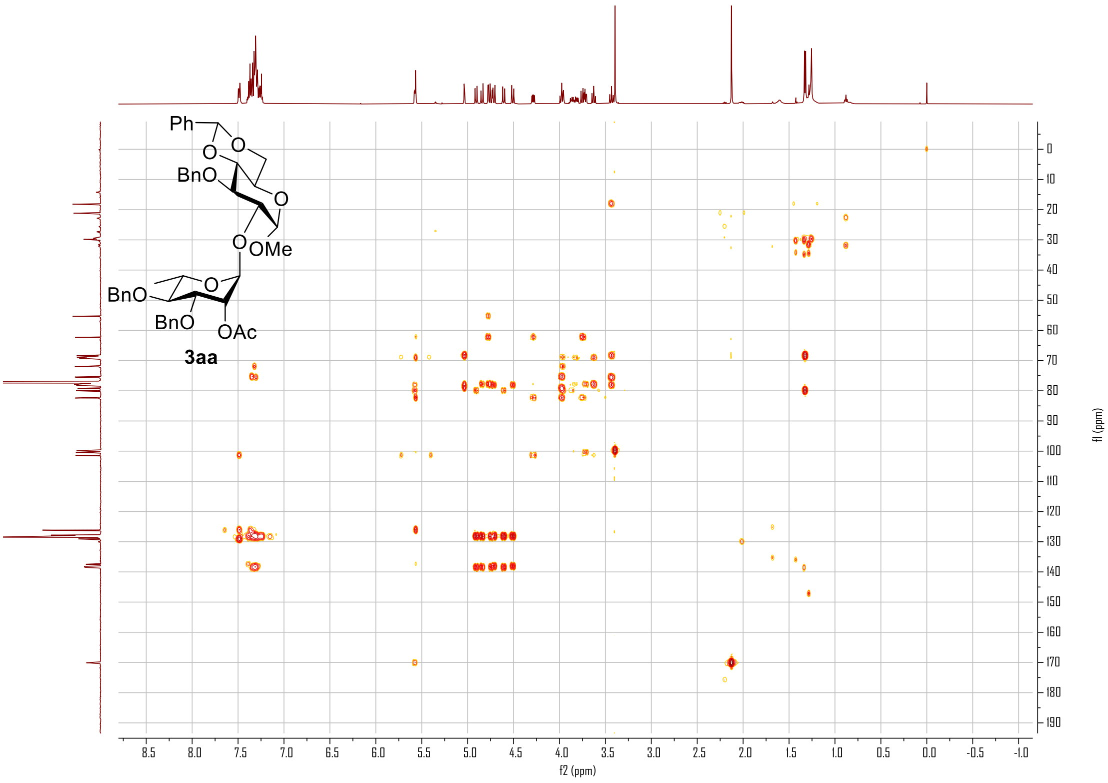


**Supplementary Figure S160. HMBC (500 MHz, CDCl₃) Spectrum for compound 3aa**

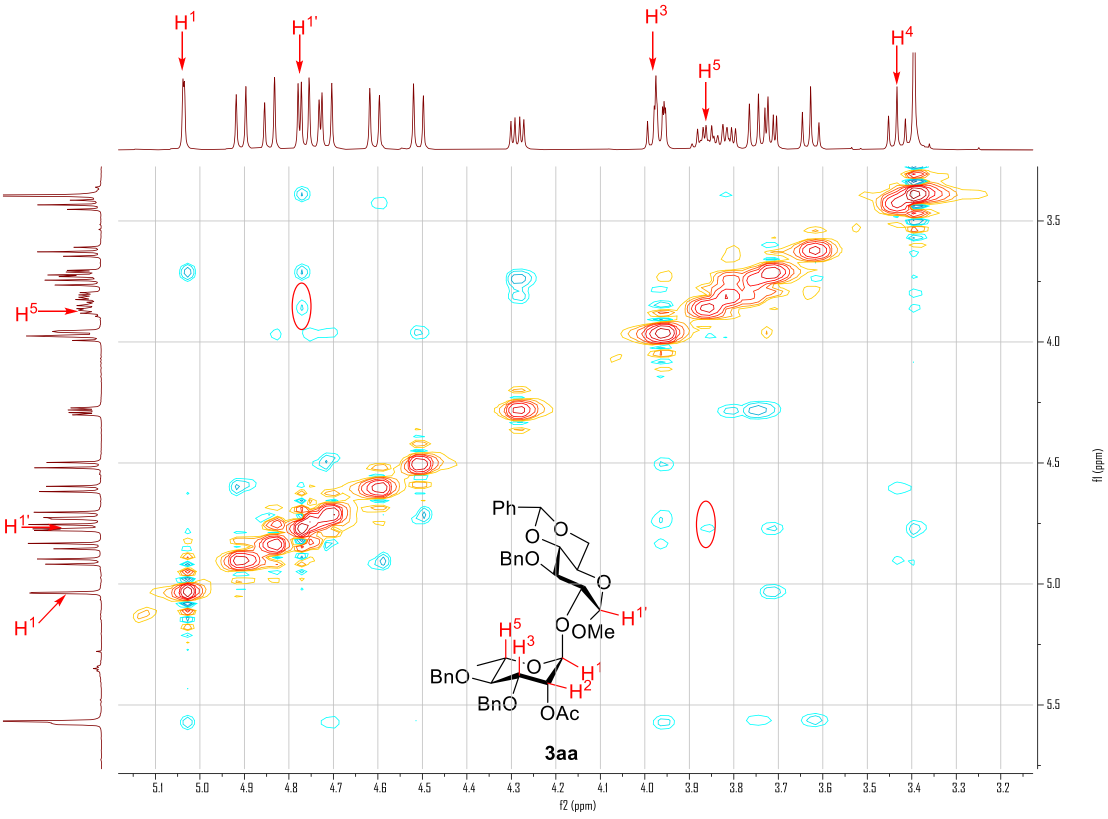


**Supplementary Figure S161. NOESY (500 MHz, CDCl₃) Spectrum for compound 3aa**

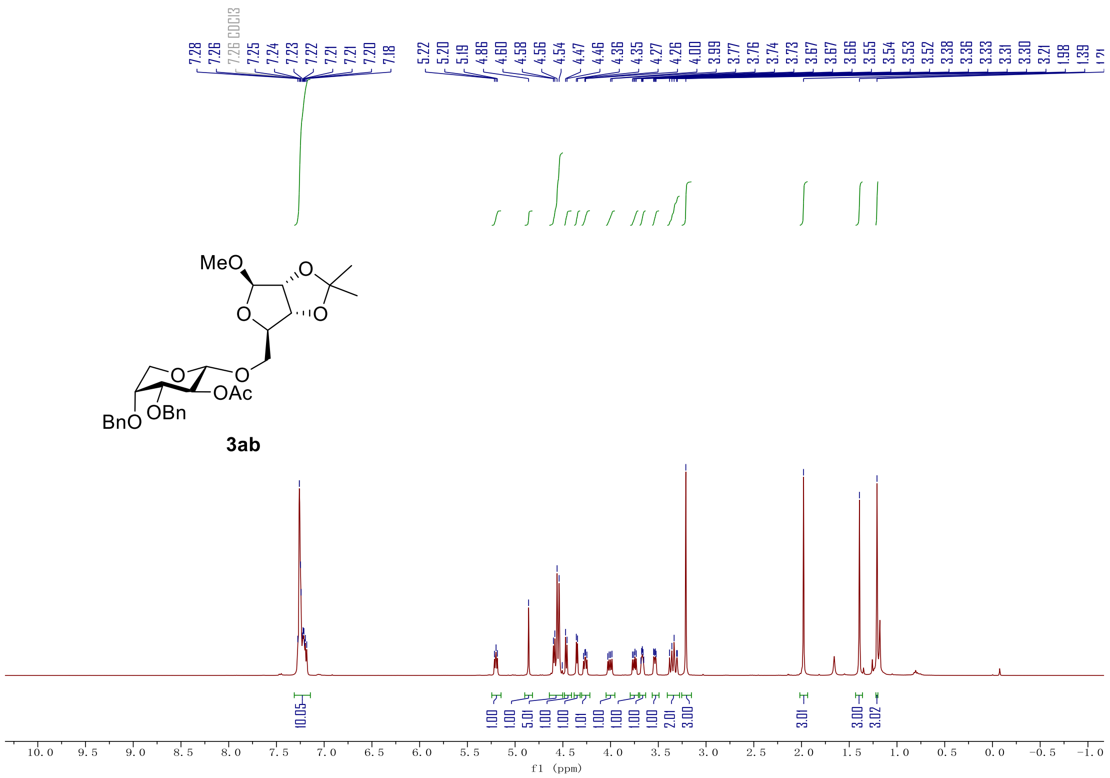


**Supplementary Figure S162. ¹H NMR (400 MHz, $CDCl_3$) Spectra for compound 3ab**

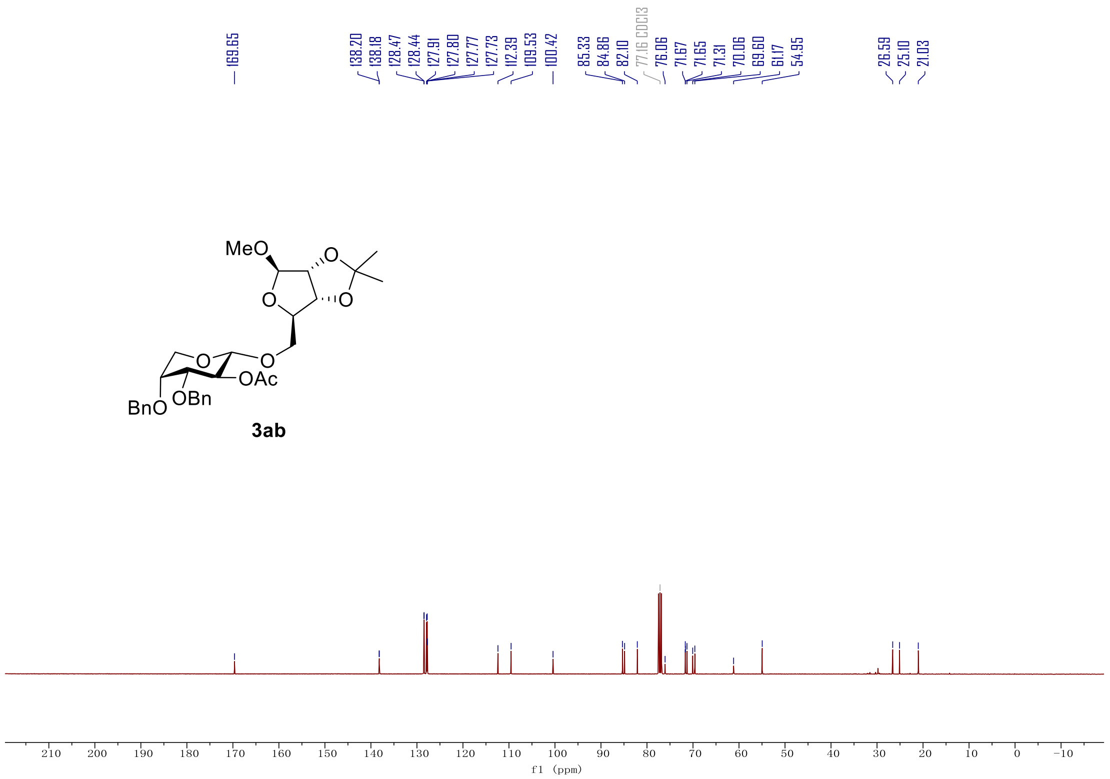


**Supplementary Figure S163. ¹³C NMR (101 MHz, $CDCl_3$) Spectra for compound 3ab**

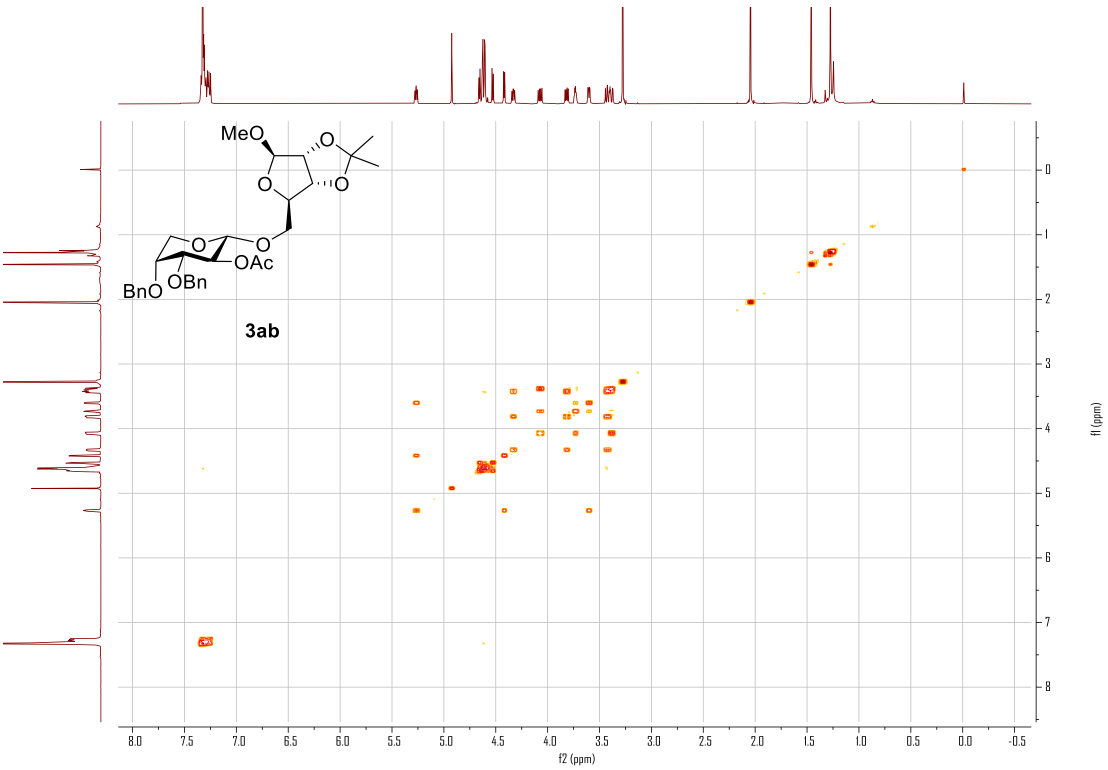


**Supplementary Figure S164. COSY (500 MHz, $CDCl_3$) Spectrum for compound 3ab**

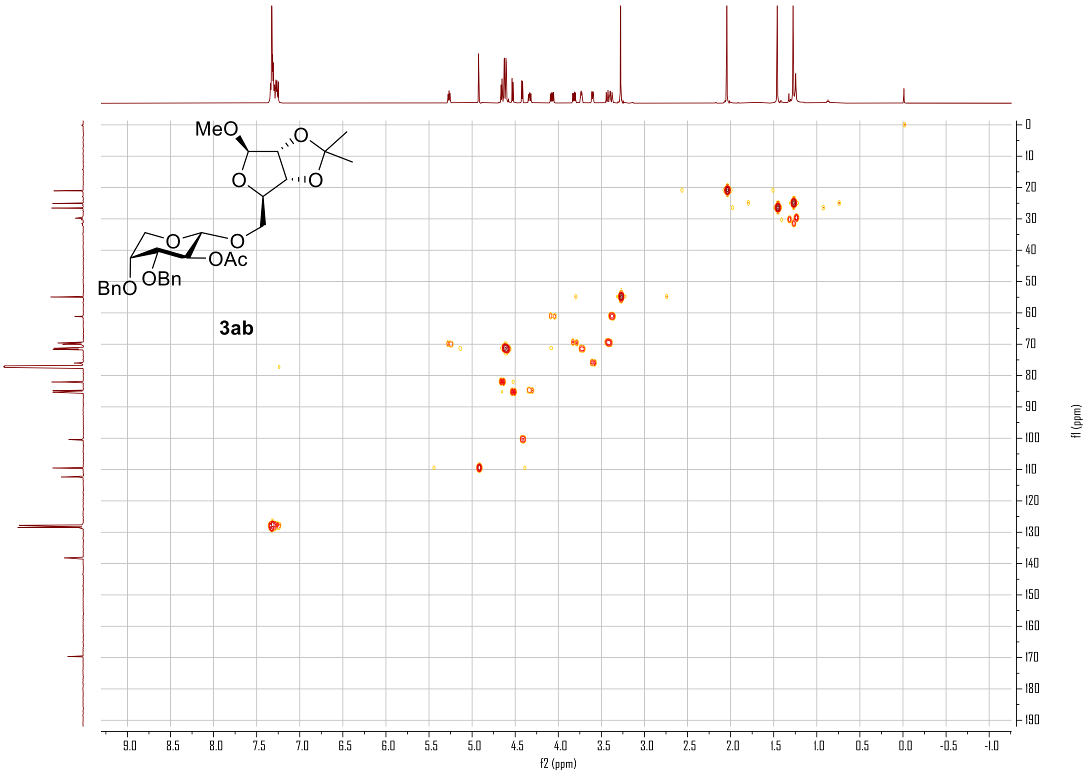


**Supplementary Figure S165. HMQC (500 MHz, $CDCl_3$) Spectrum for compound 3ab**

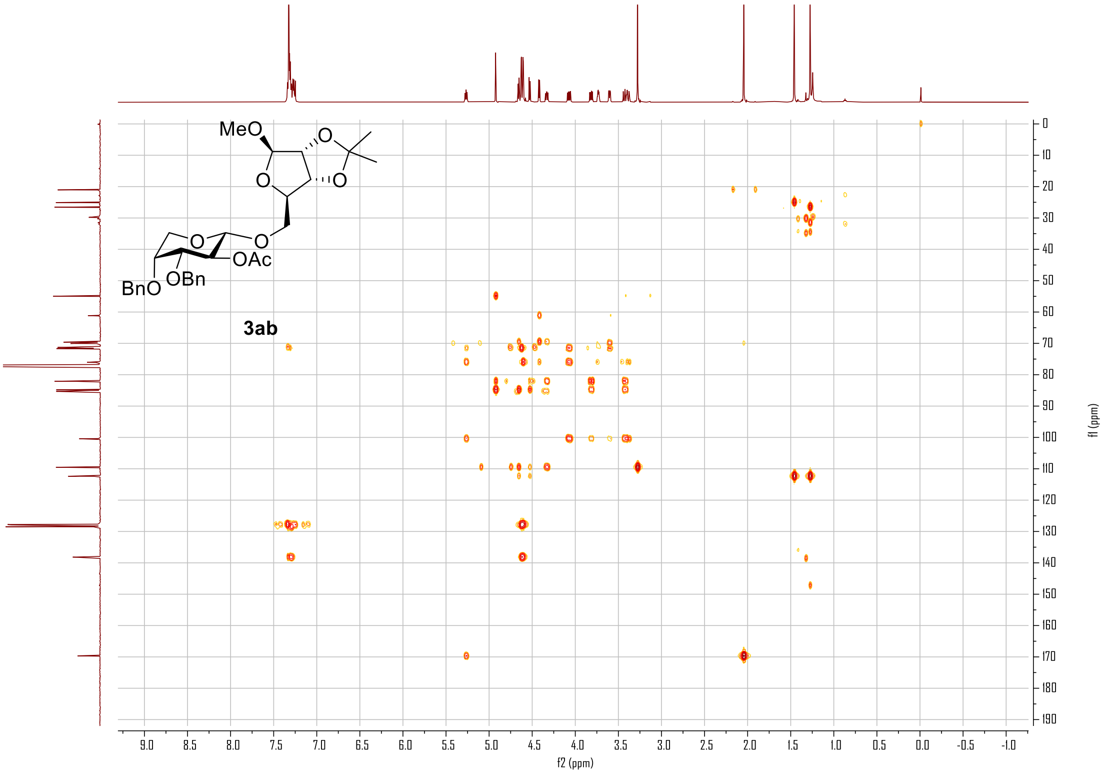


**Supplementary Figure S166. HMBC (500 MHz, $CDCl_3$) Spectrum for compound 3ab**

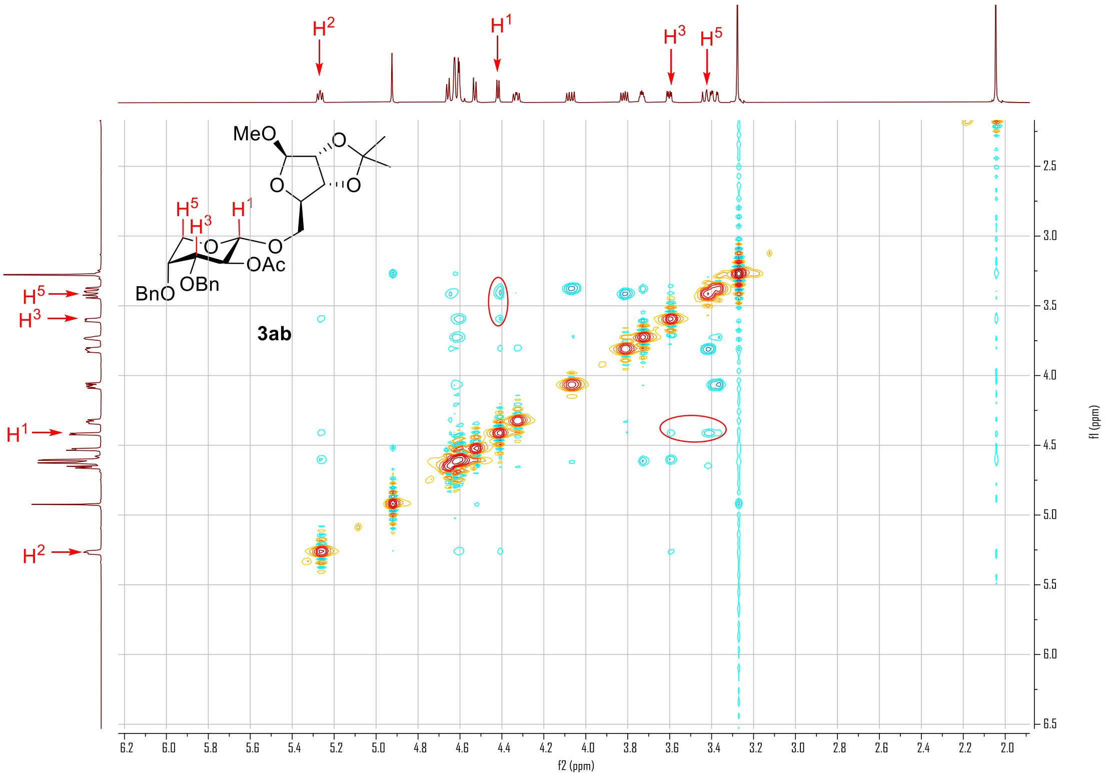


**Supplementary Figure S167. NOESY (500 MHz, $CDCl_3$) Spectrum for compound 3ab**

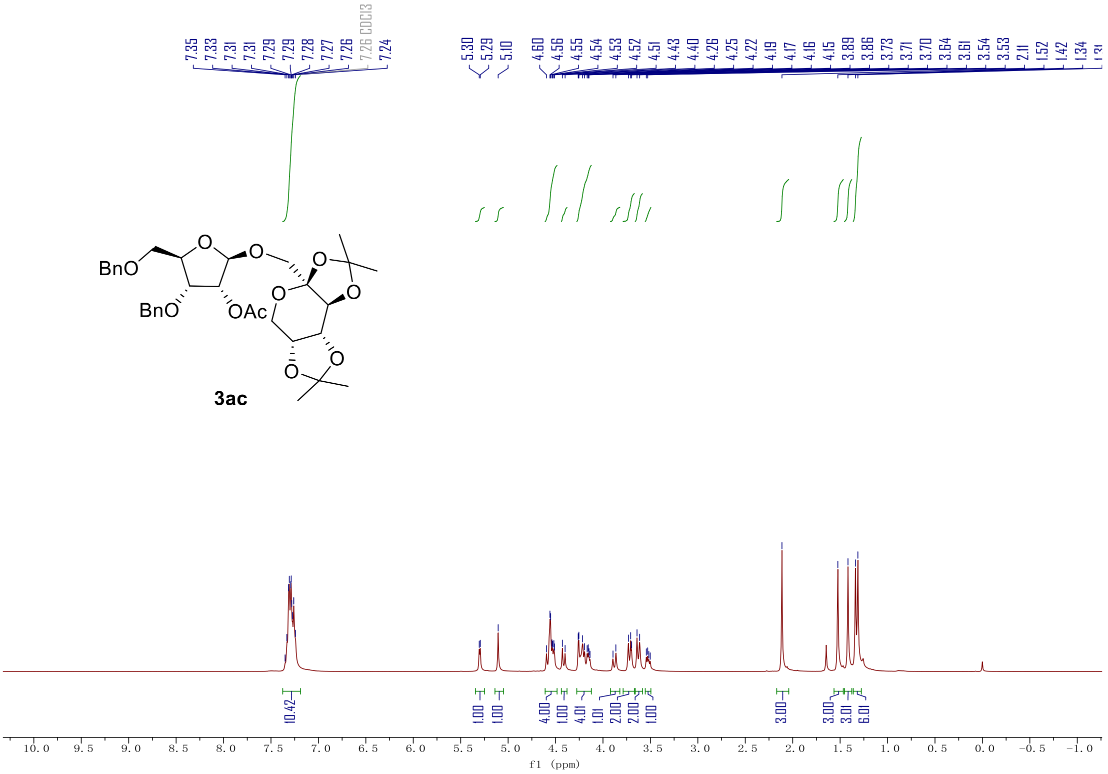


**Supplementary Figure S168. $^{1}$H NMR (400 MHz, $CDCl_3$) Spectra for compound 3ac**

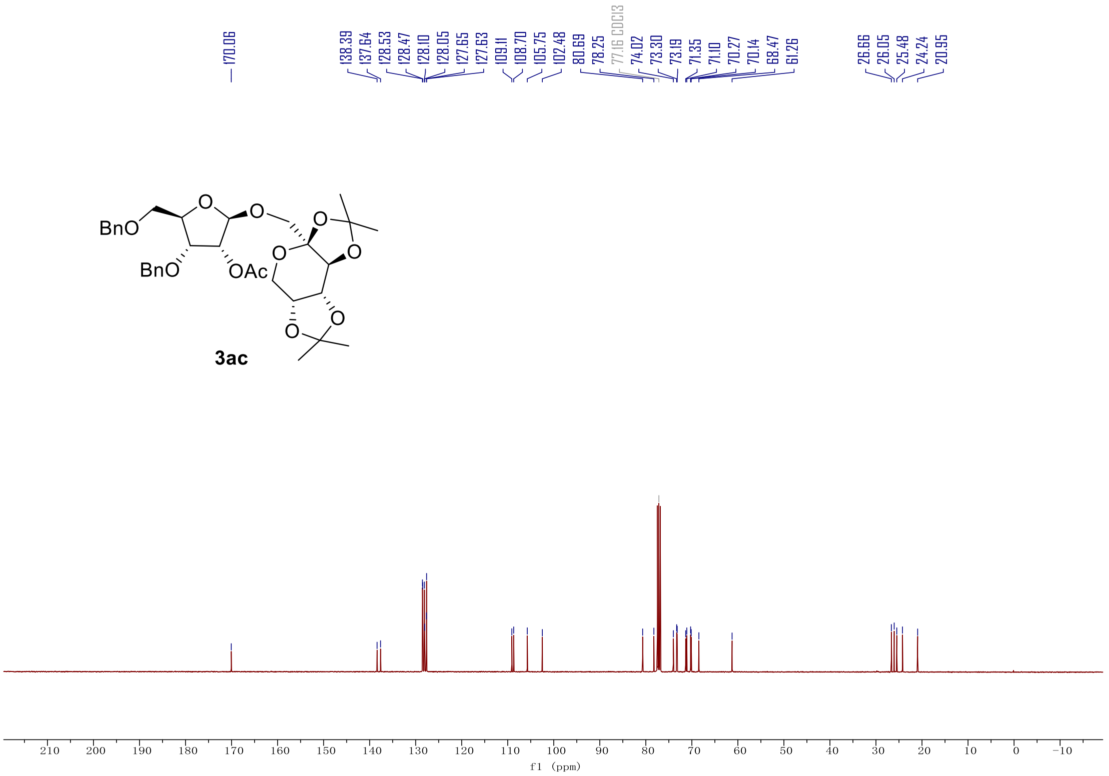


**Supplementary Figure S169. $^{13}$C NMR (101 MHz, $CDCl_3$) Spectra for compound 3ac**

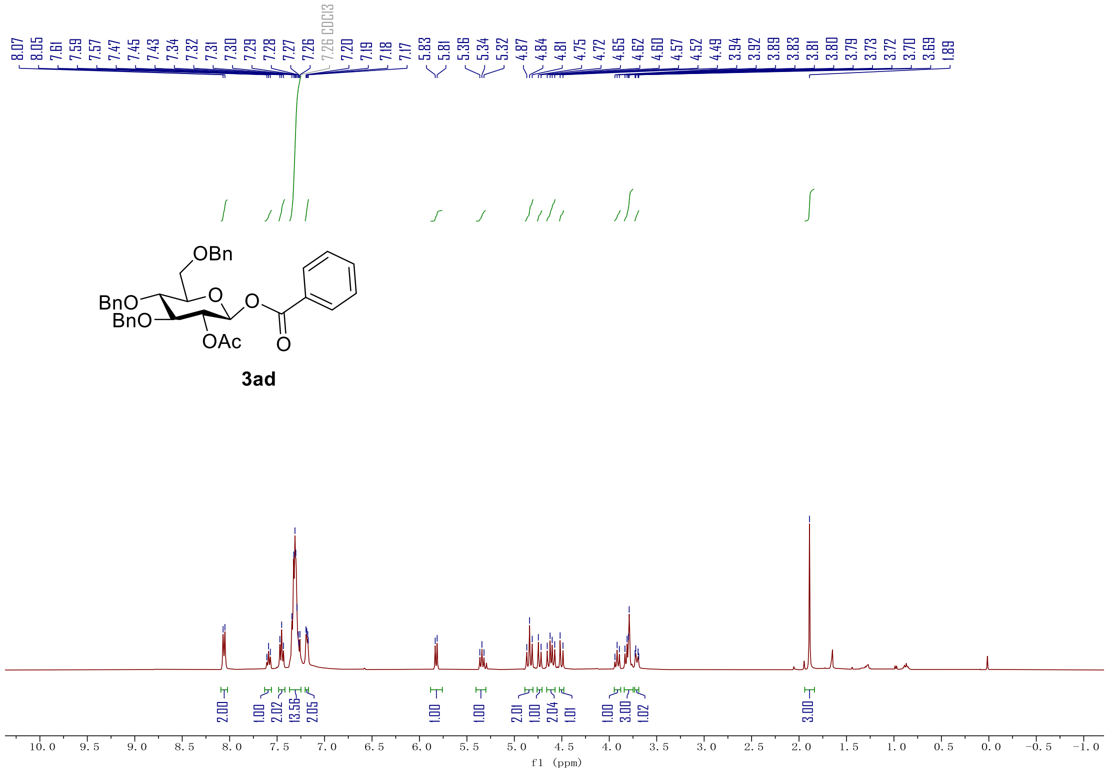

Supplementary Figure S170. $^{1}H$ NMR (400 MHz, $CDCl_3$) Spectra for compound 3ad

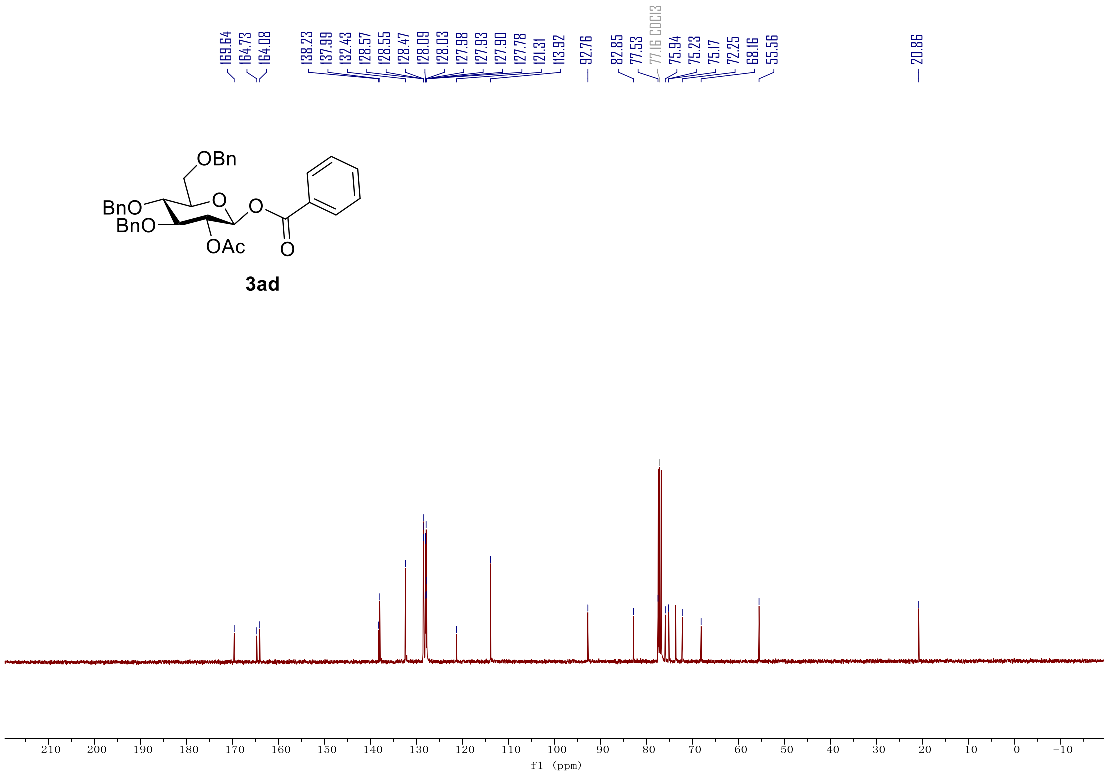

Supplementary Figure S171. $^{13}C$ NMR (101 MHz, $CDCl_3$) Spectra for compound 3ad

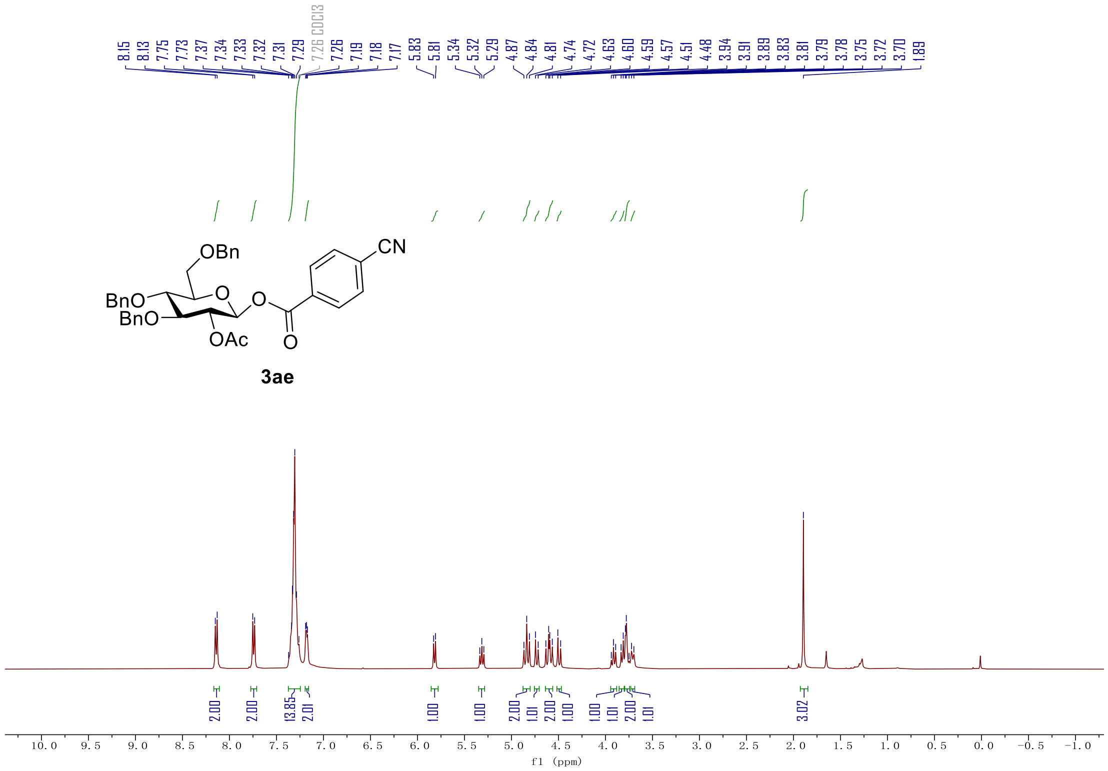

**Supplementary Figure S172. $^{1}$H NMR (400 MHz, CDCl$_3$) Spectra for compound 3ae**

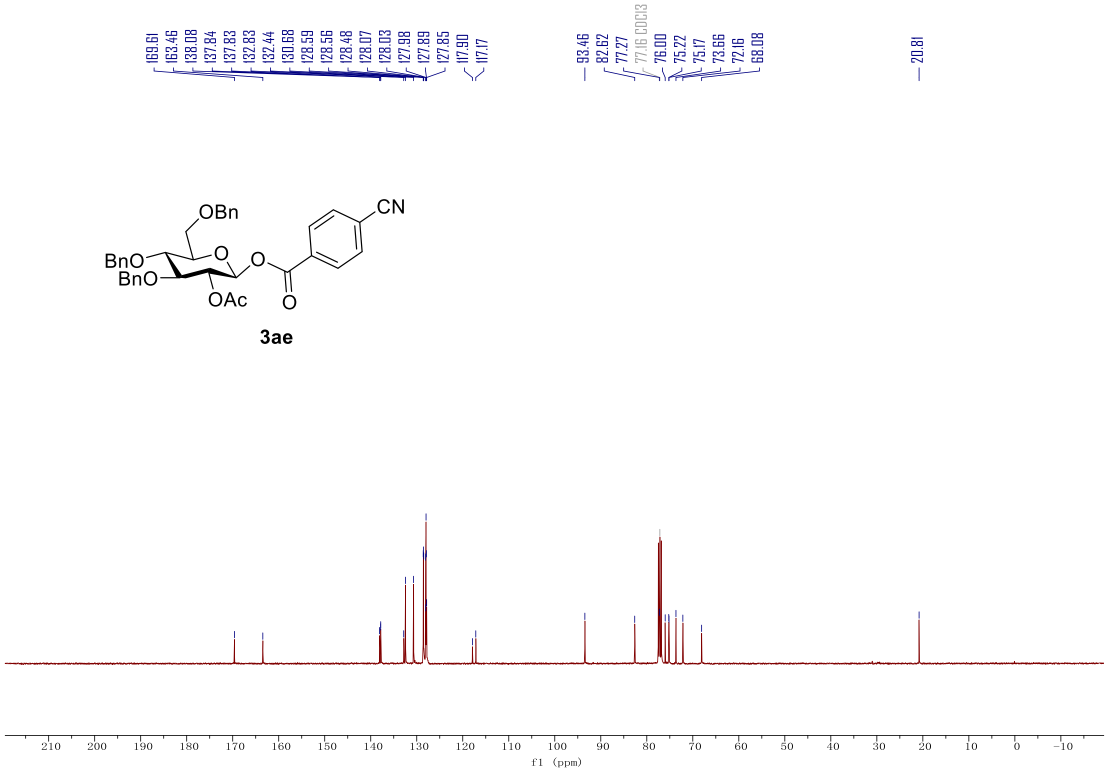

**Supplementary Figure S173. $^{13}$C NMR (101 MHz, CDCl$_3$) Spectra for compound 3ae**

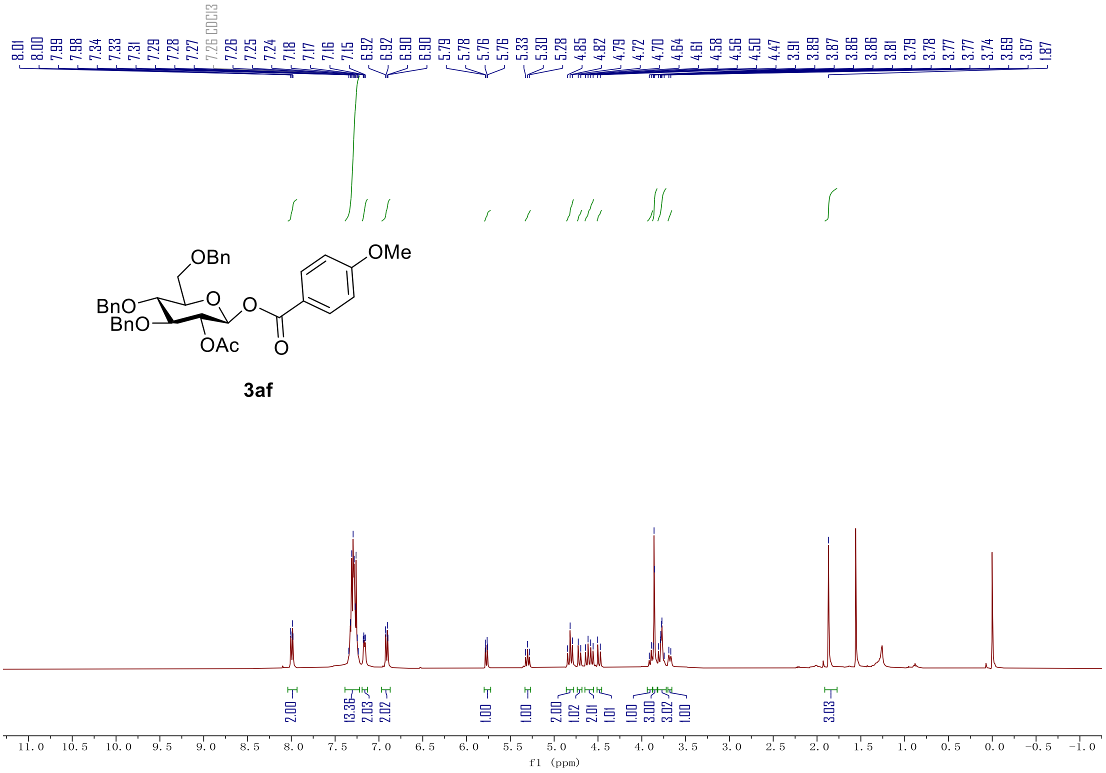


**Supplementary Figure S174. $^{1}$H NMR (400 MHz, $CDCl_3$) Spectra for compound 3af**

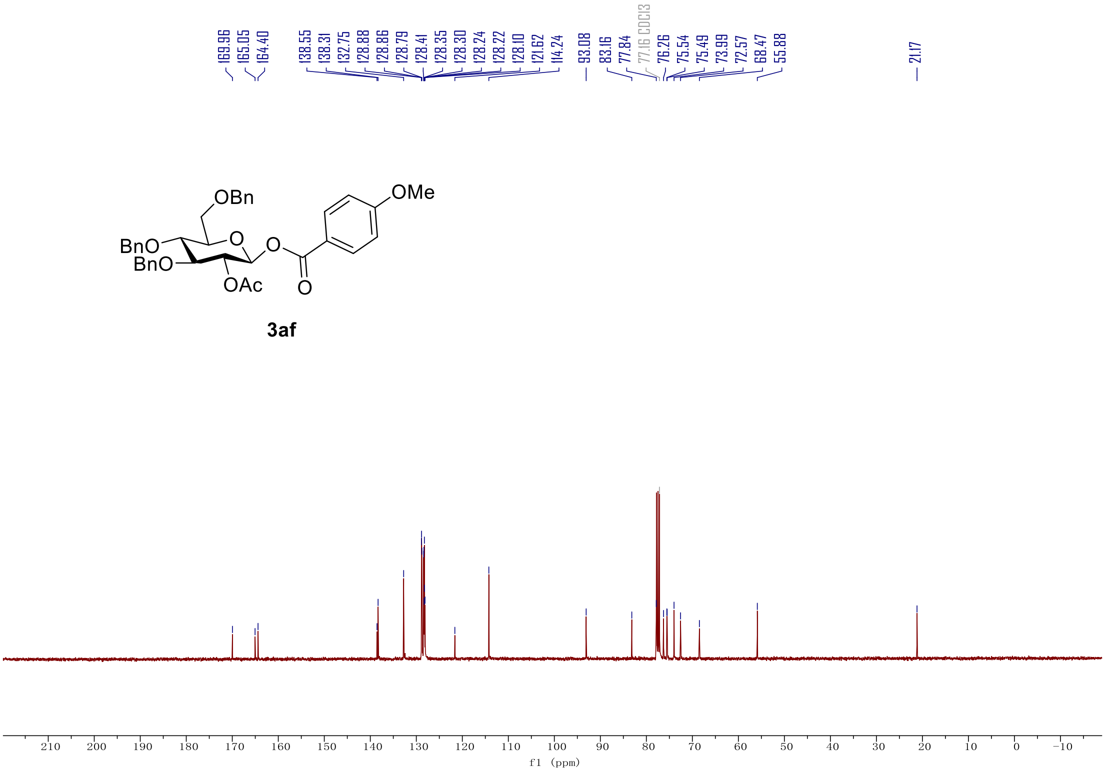


**Supplementary Figure S175. $^{13}$C NMR (101 MHz, $CDCl_3$) Spectra for compound 3af**

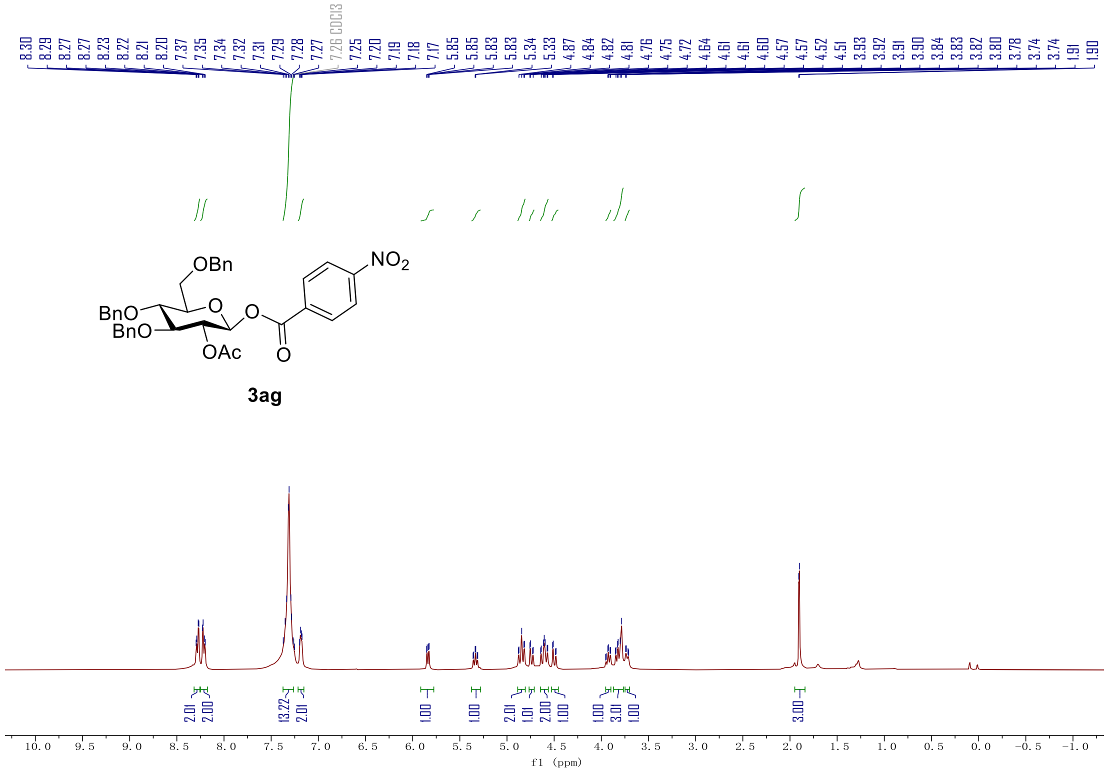


**Supplementary Figure S176. $^{1}$H NMR (400 MHz, $CDCl_3$) Spectra for compound 3ag**

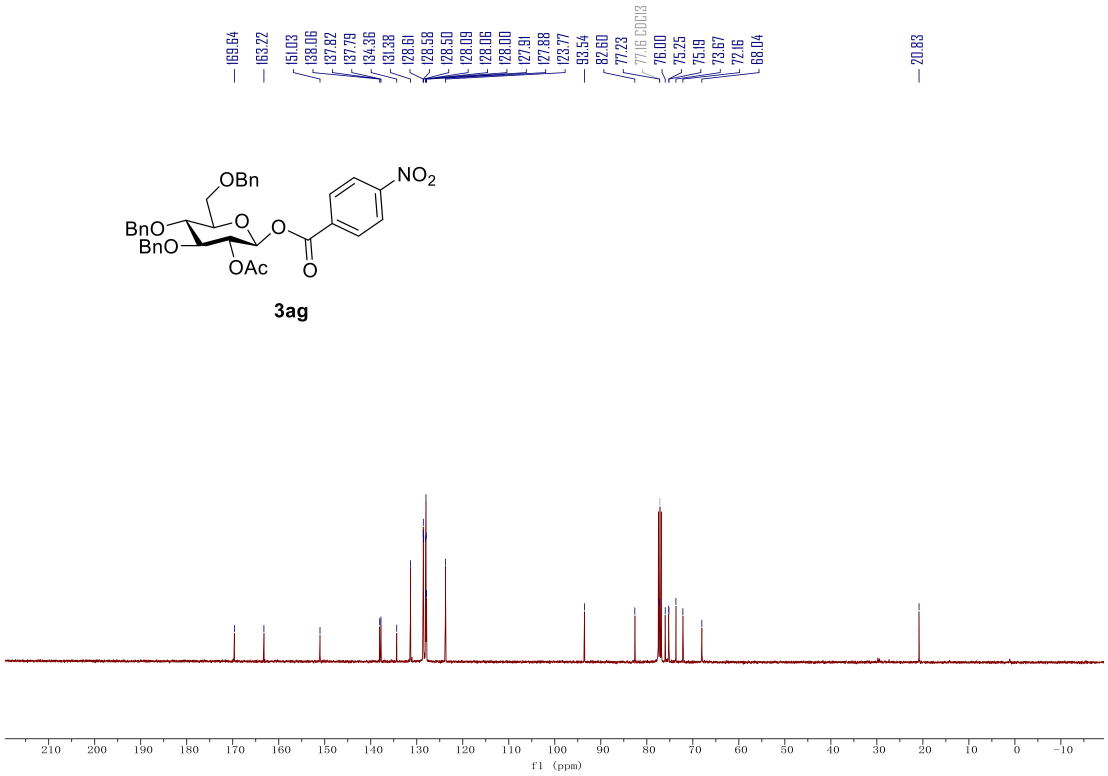


**Supplementary Figure S177. $^{13}$C NMR (101 MHz, $CDCl_3$) Spectra for compound 3ag**

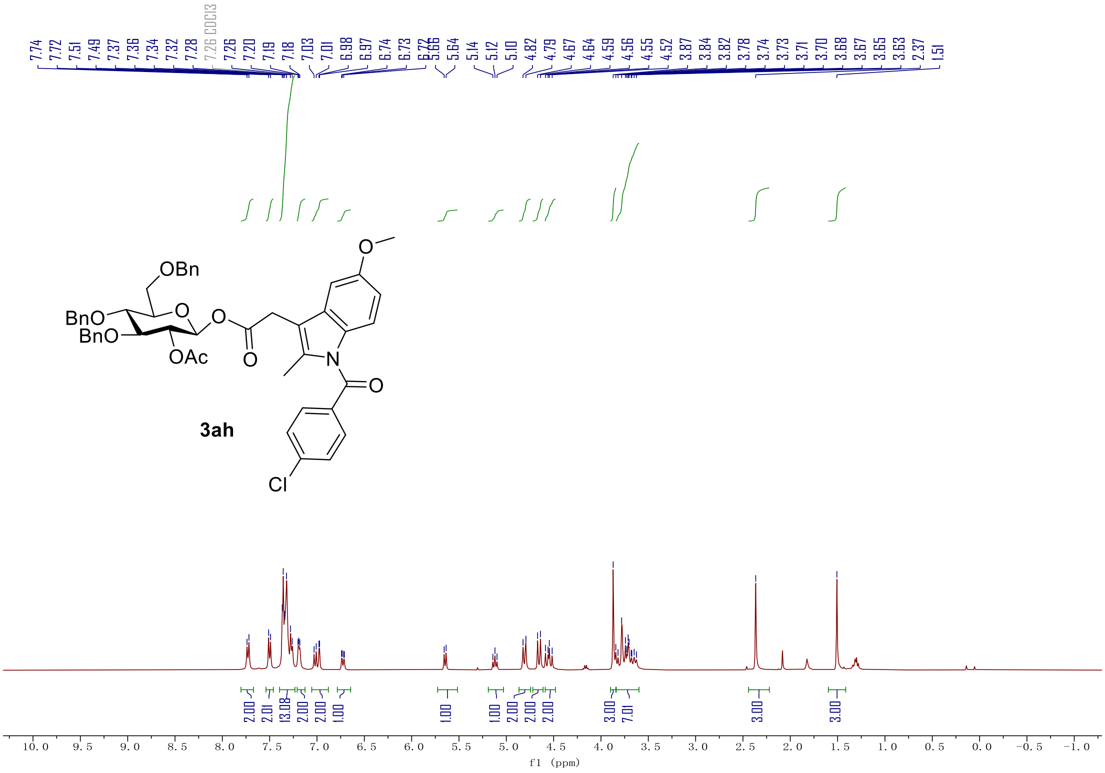


**Supplementary Figure S178.** **$^{1}$H NMR (400 MHz, $CDCl_3$) Spectra for compound 3ah**

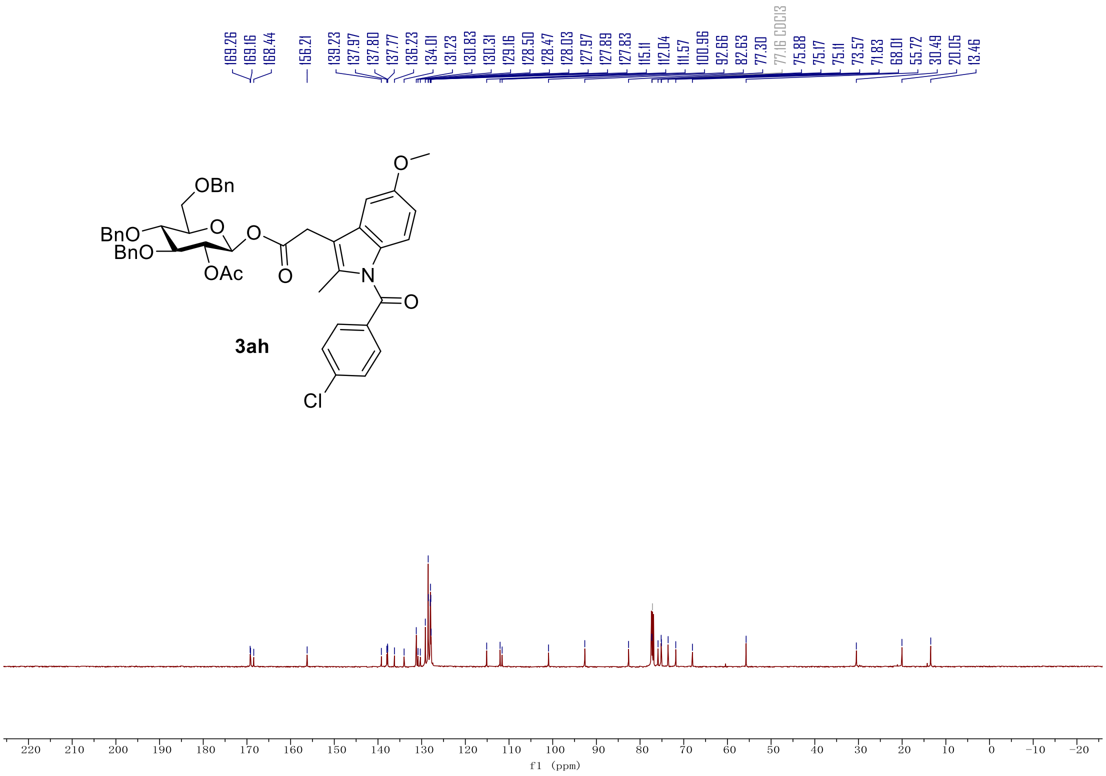


**Supplementary Figure S179.** **$^{13}$C NMR (126 MHz, $CDCl_3$) Spectra for compound 3ah**

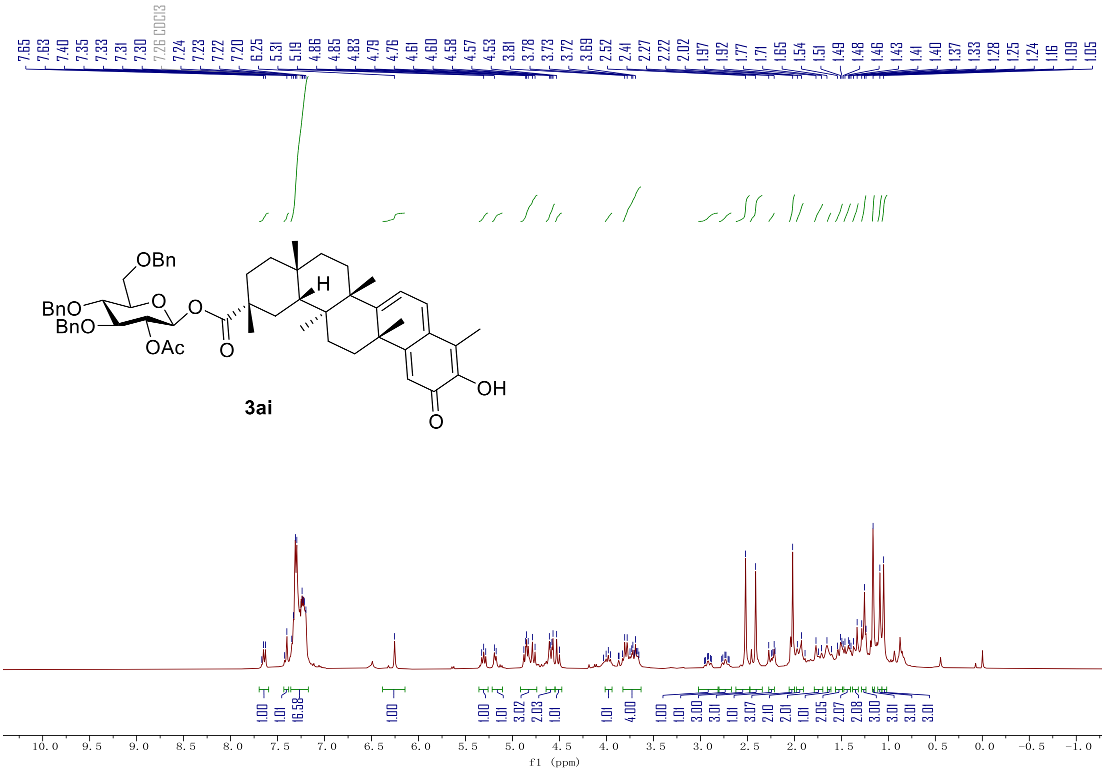


**Supplementary Figure S180.** $^{1}$**H NMR (400 MHz,** $CDCl_3$**) Spectra for compound 3ai**

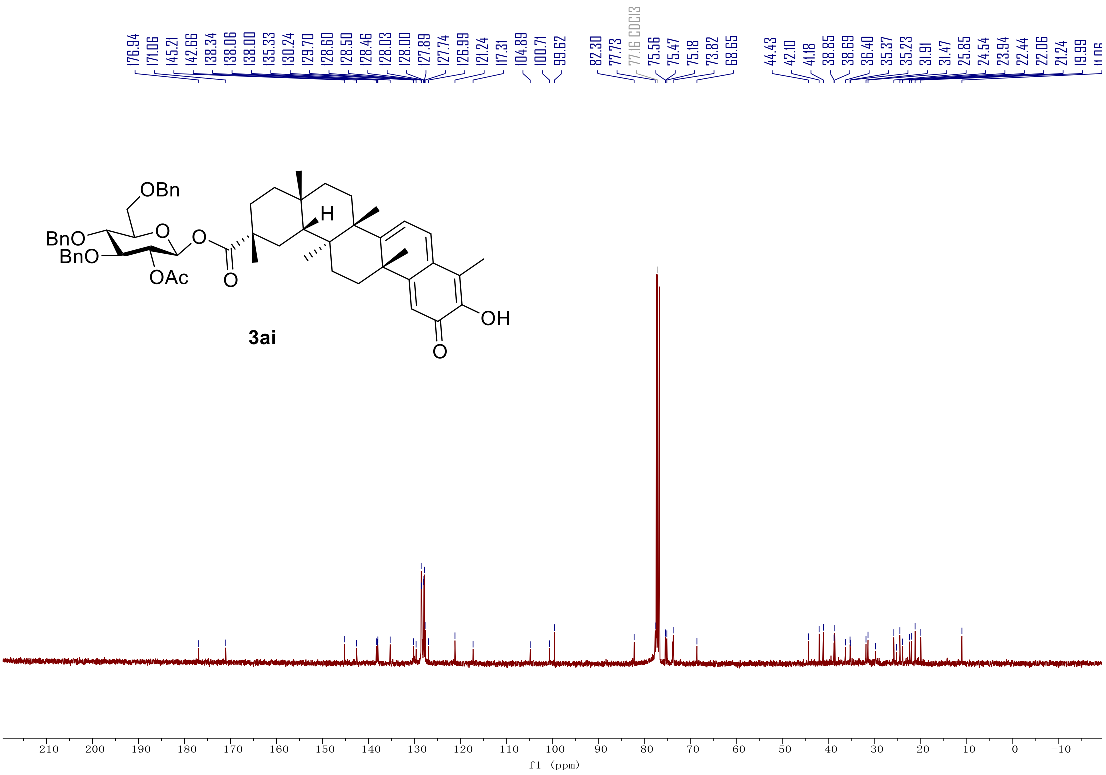


**Supplementary Figure S181.** $^{13}$**C NMR (101 MHz,** $CDCl_3$**) Spectra for compound 3ai**

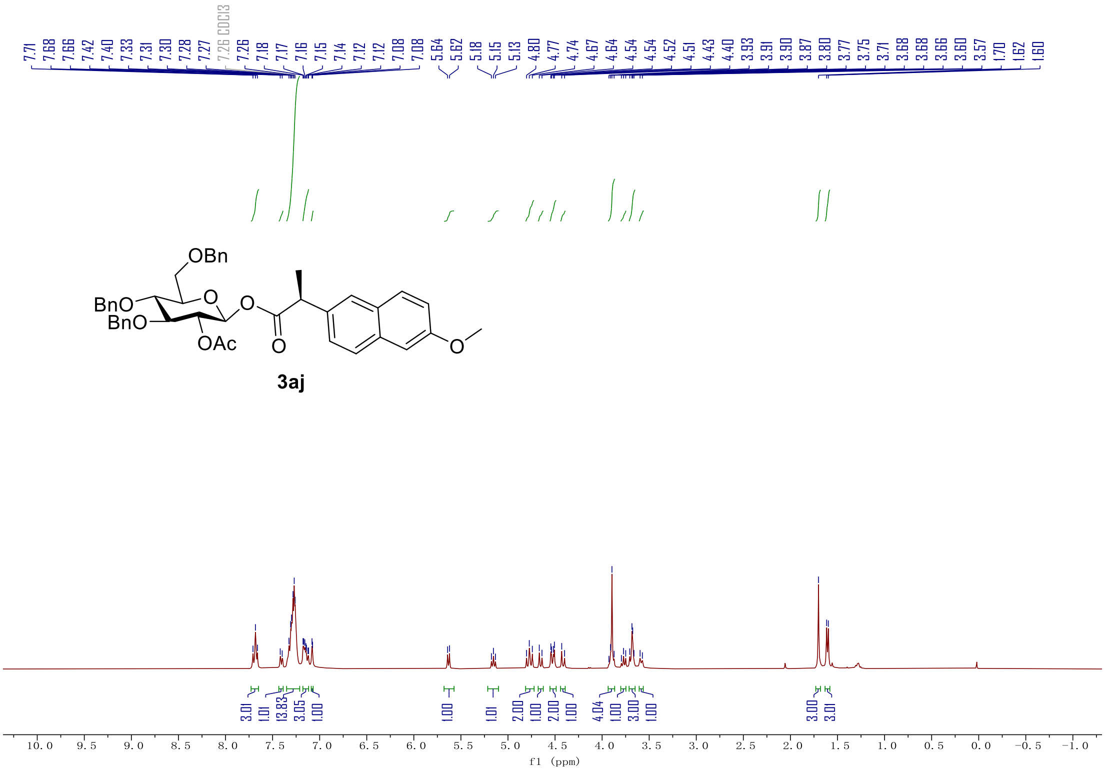


**Supplementary Figure S182. $^{1}$H NMR (400 MHz, $CDCl_3$) Spectra for compound 3aj**

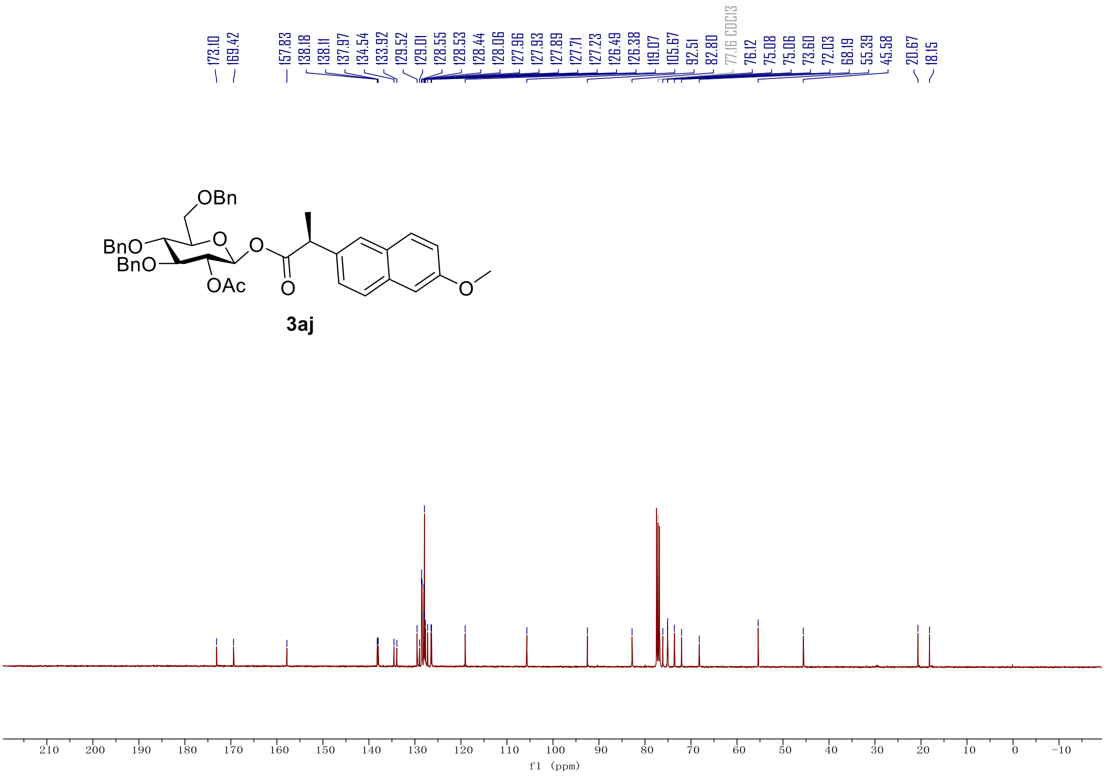


**Supplementary Figure S183. $^{13}$C NMR (101 MHz, $CDCl_3$) Spectra for compound 3aj**

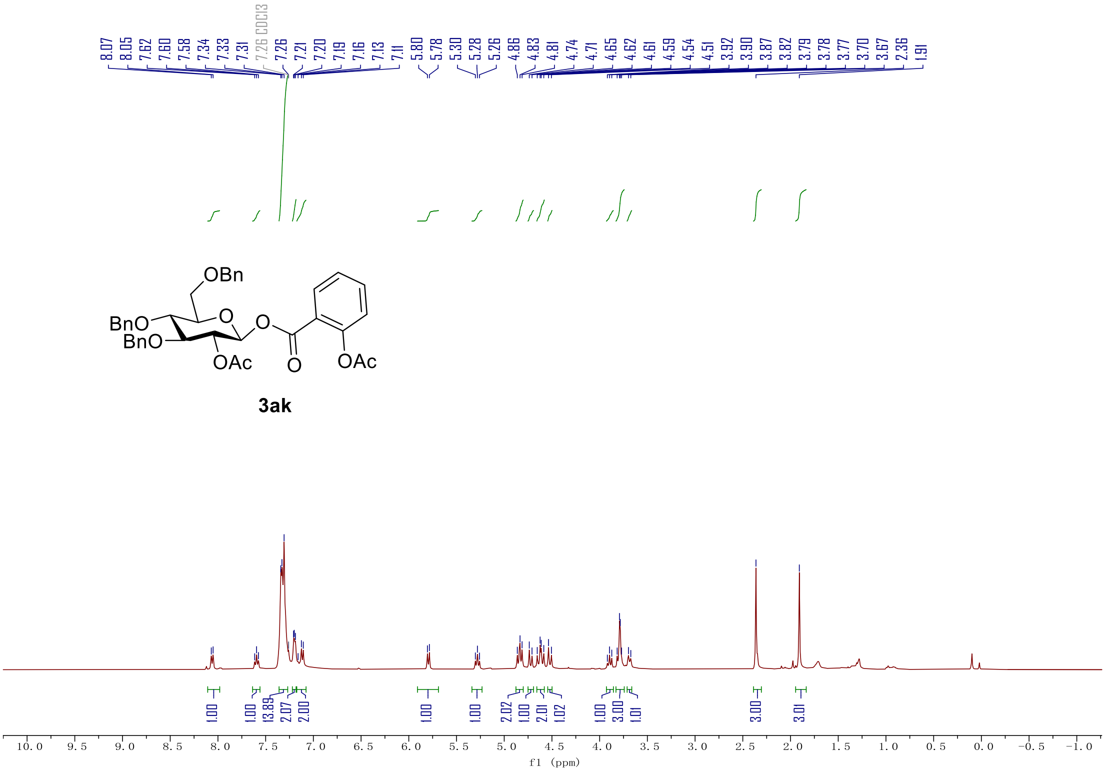


**Supplementary Figure S184. $^{1}$H NMR (400 MHz, $CDCl_3$) Spectra for compound 3ak**

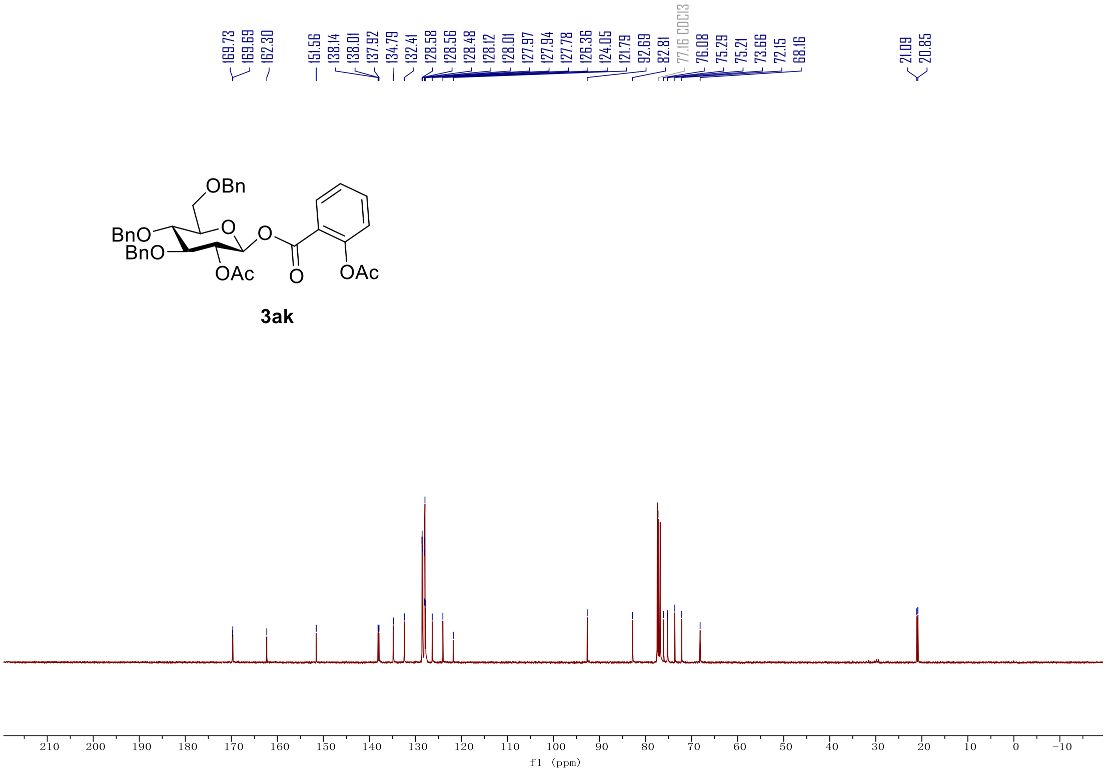


**Supplementary Figure S185. $^{13}$C NMR (101 MHz, $CDCl_3$) Spectra for compound 3ak**

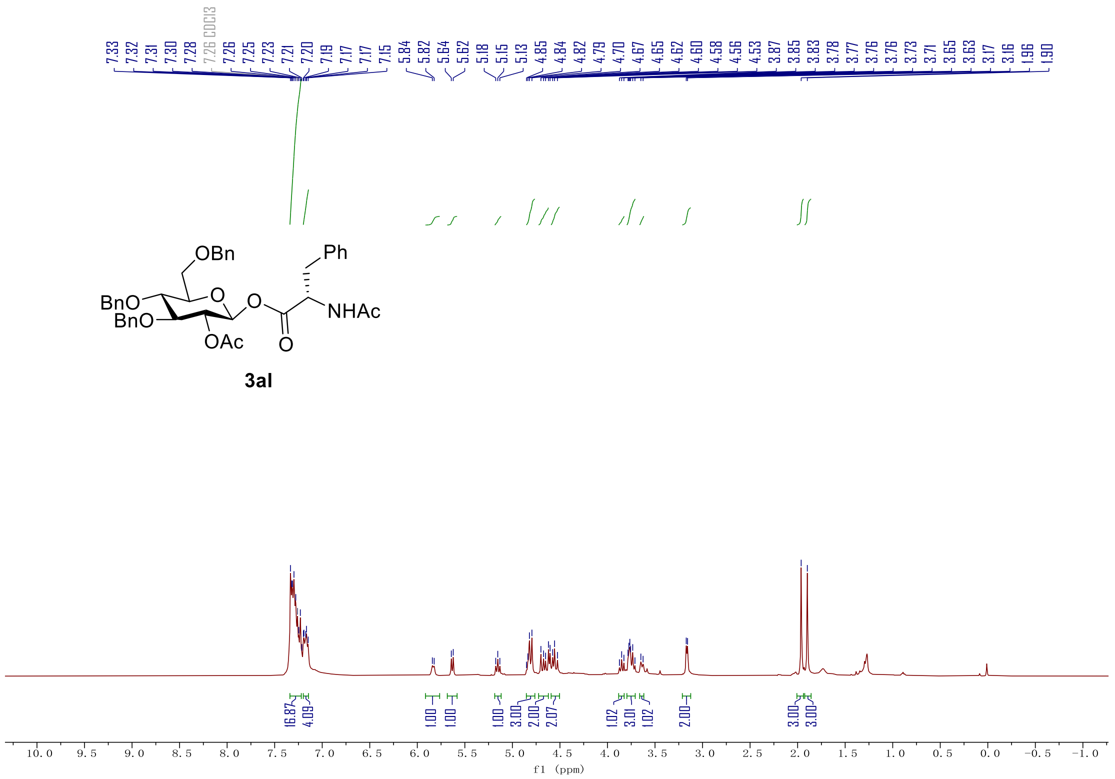


**Supplementary Figure S186. $^{1}$H NMR (400 MHz, $CDCl_3$) Spectra for compound 3al**

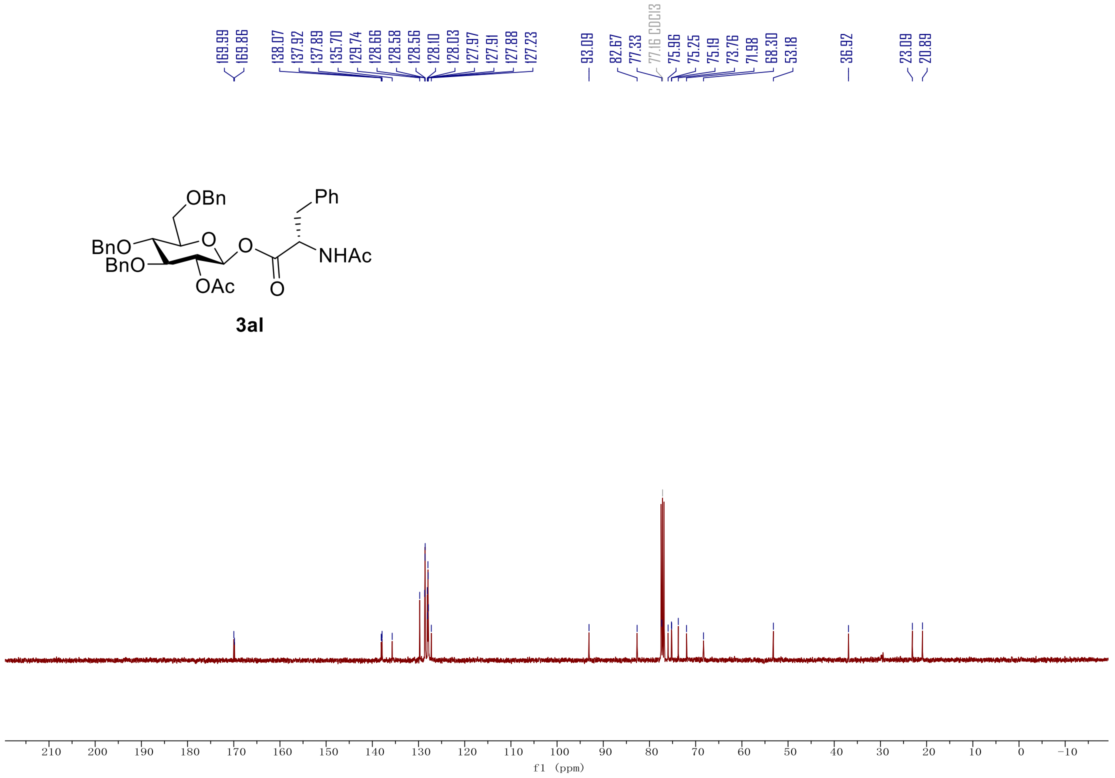


**Supplementary Figure S187. $^{13}$C NMR (101 MHz, $CDCl_3$) Spectra for compound 3al**

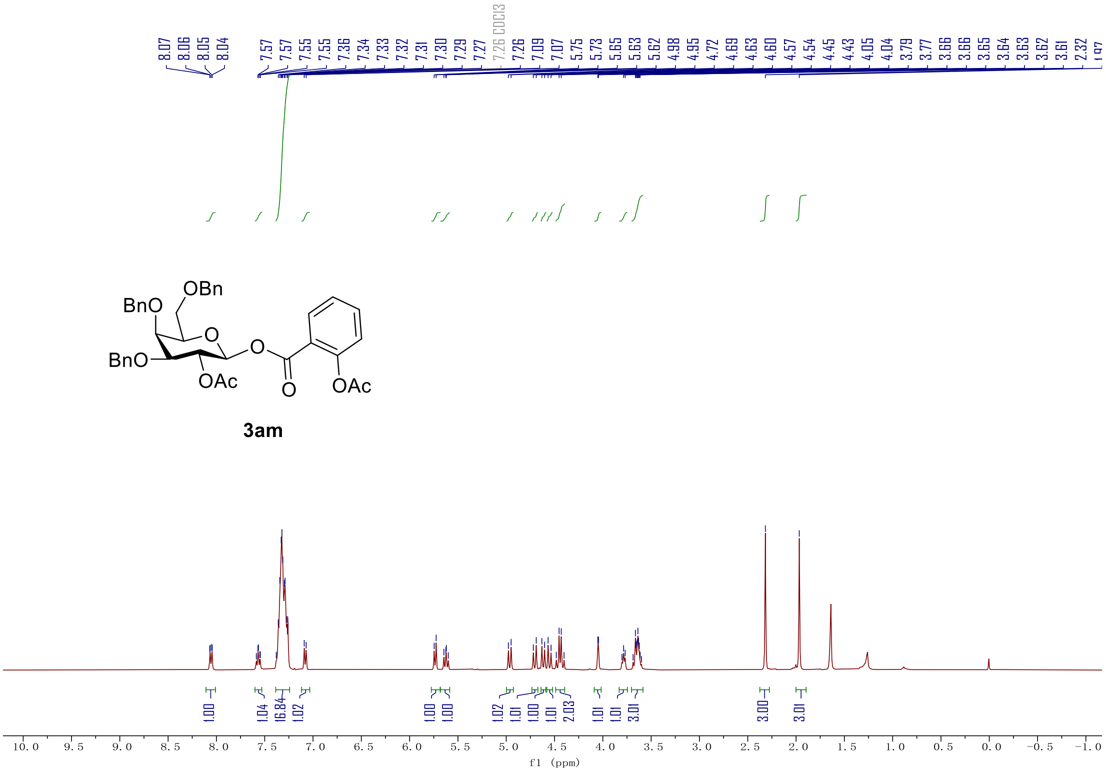


**Supplementary Figure S188. $^{1}$H NMR (400 MHz, $CDCl_3$) Spectra for compound 3am**

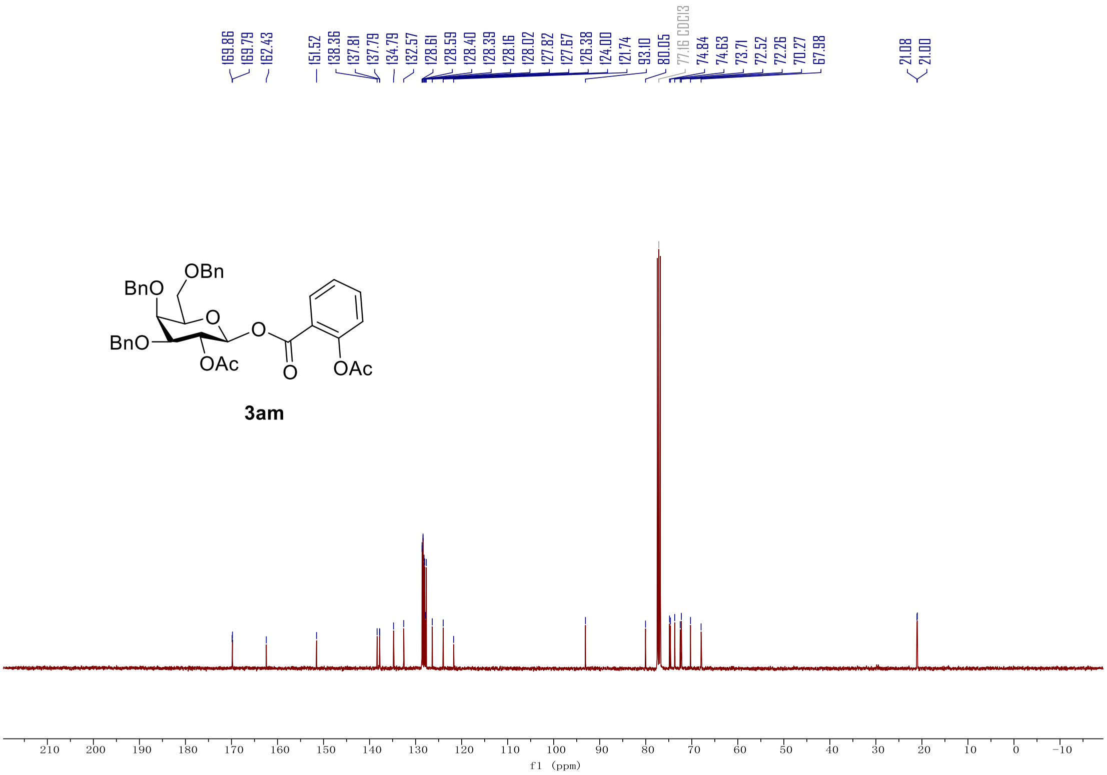


**Supplementary Figure S189. $^{13}$C NMR (101 MHz, $CDCl_3$) Spectra for compound 3am**

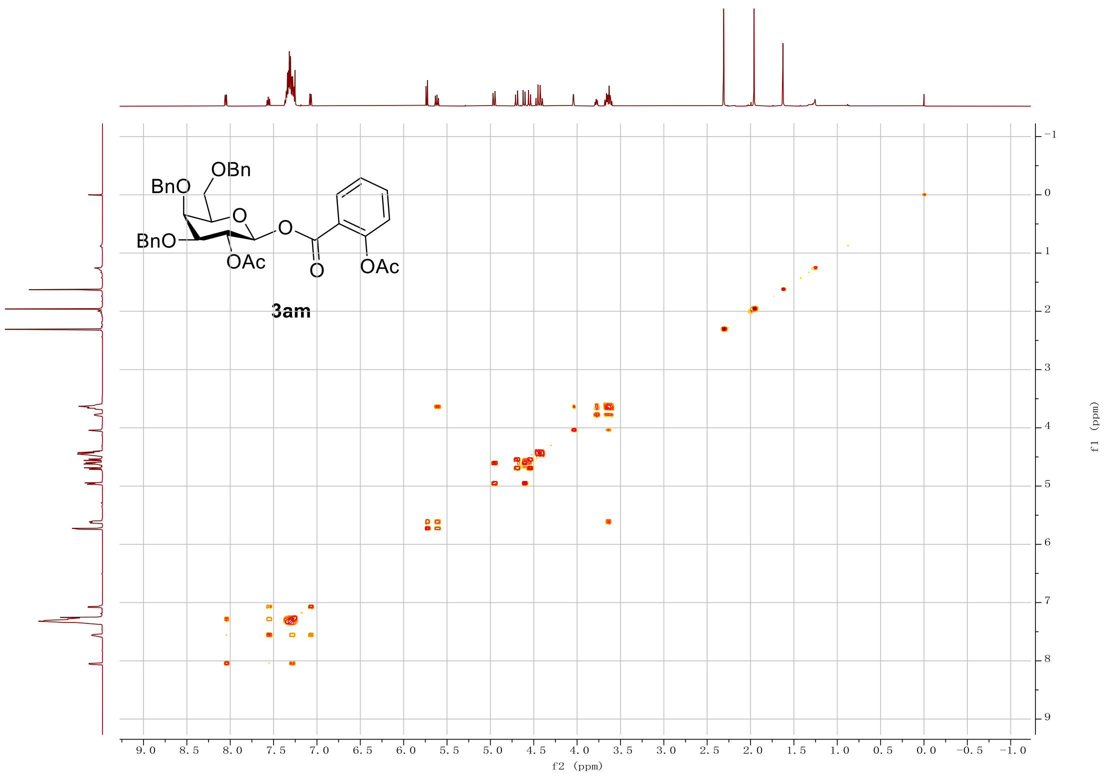


**Supplementary Figure S190. COSY (500 MHz, $CDCl_3$) Spectrum for compound 3am**

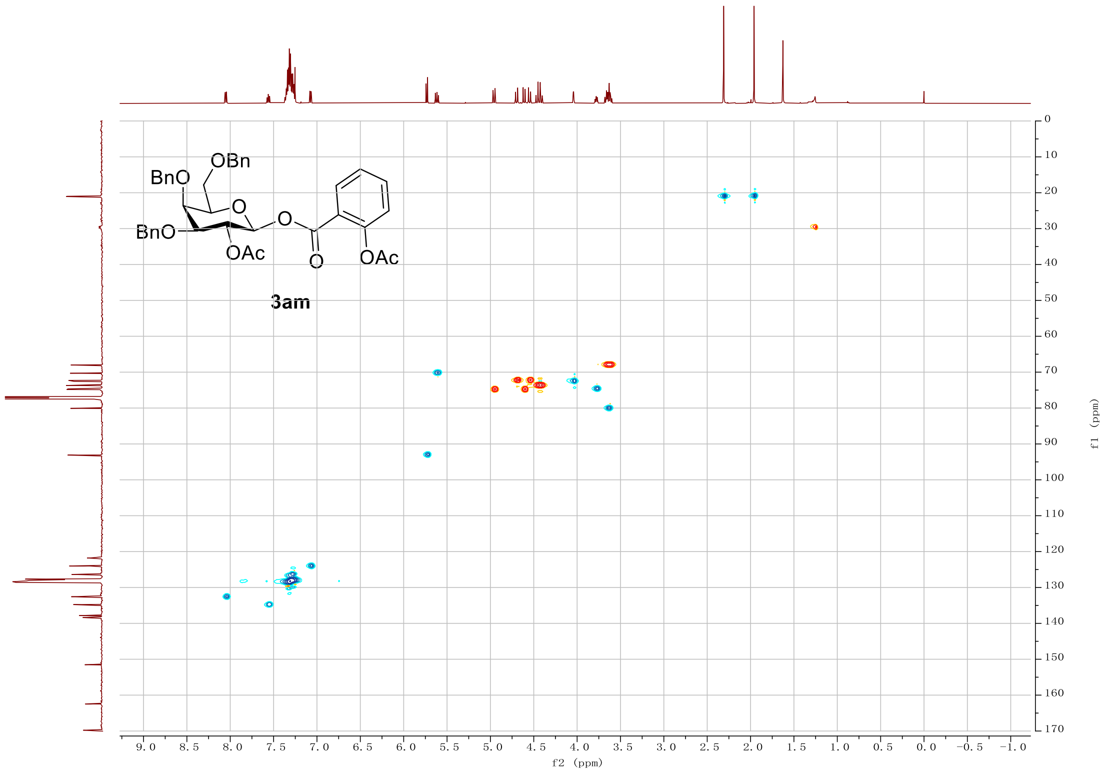


**Supplementary Figure S191. HSQC (500 MHz, $CDCl_3$) Spectrum for compound 3am**

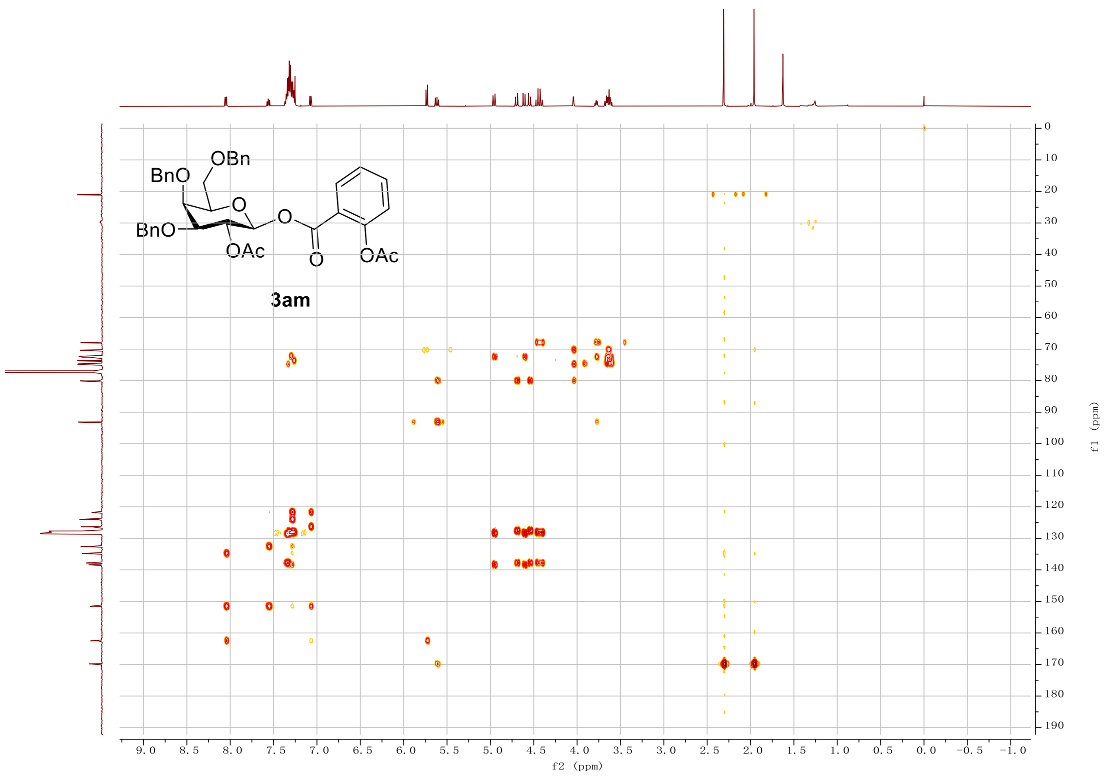


**Supplementary Figure S192. HMBC (500 MHz, $CDCl_3$) Spectrum for compound 3am**

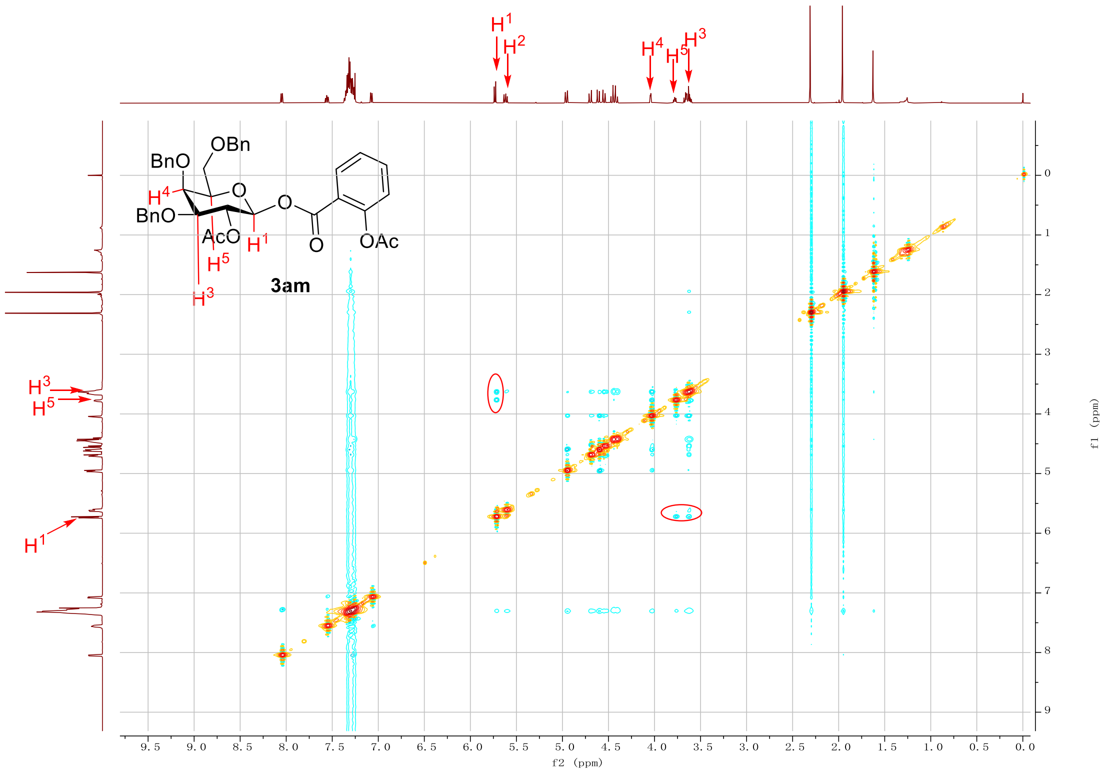


**Supplementary Figure S193. NOESY (500 MHz, $CDCl_3$) Spectrum for compound 3am**

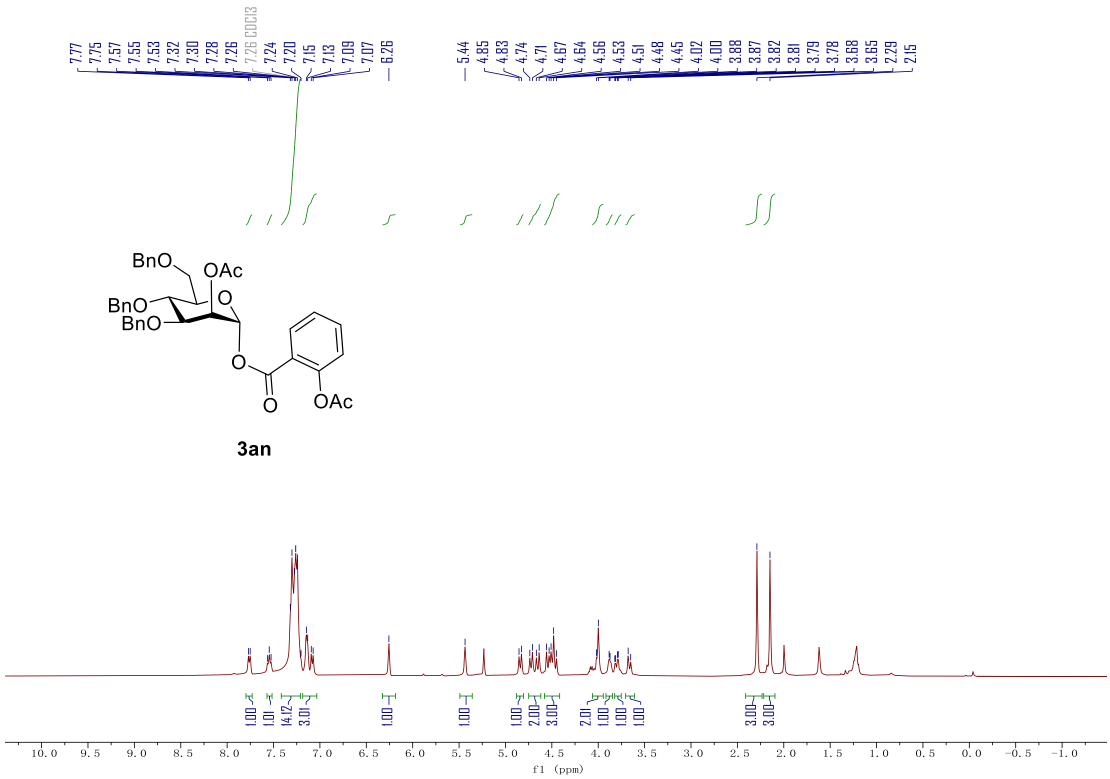


**Supplementary Figure S194. $^{1}$H NMR (400 MHz, $CDCl_3$) Spectra for compound 3an**

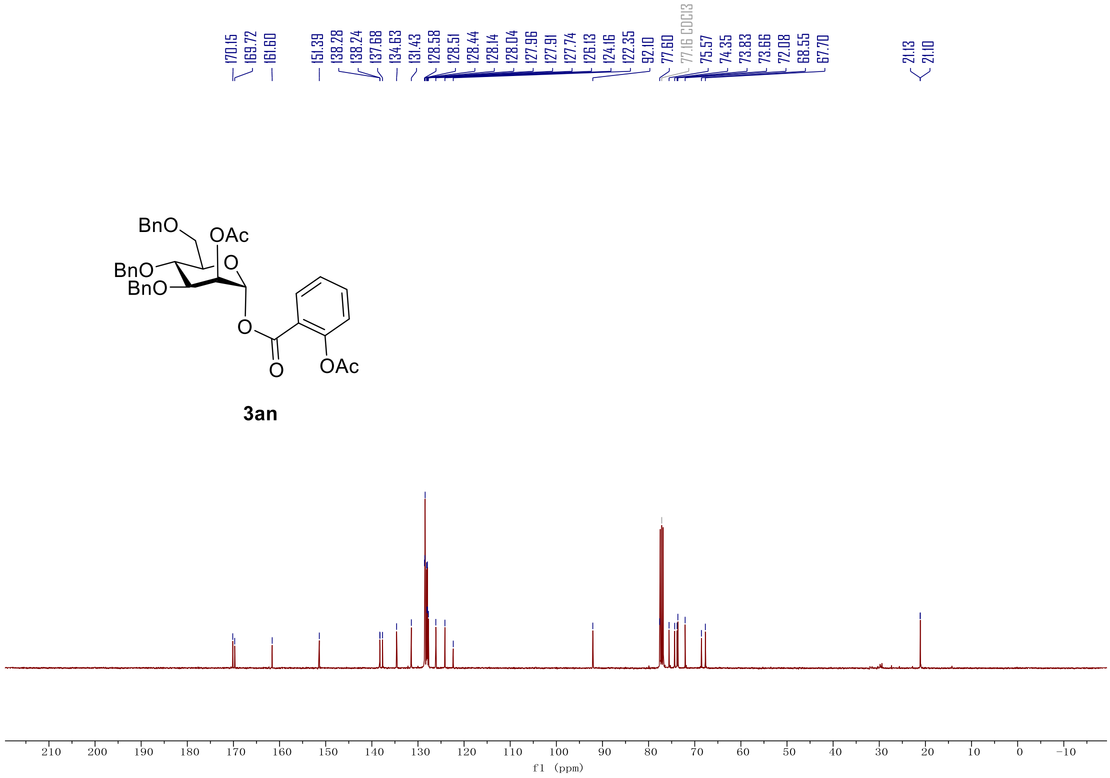


**Supplementary Figure S195. $^{13}$C NMR (101 MHz, $CDCl_3$) Spectra for compound 3an**

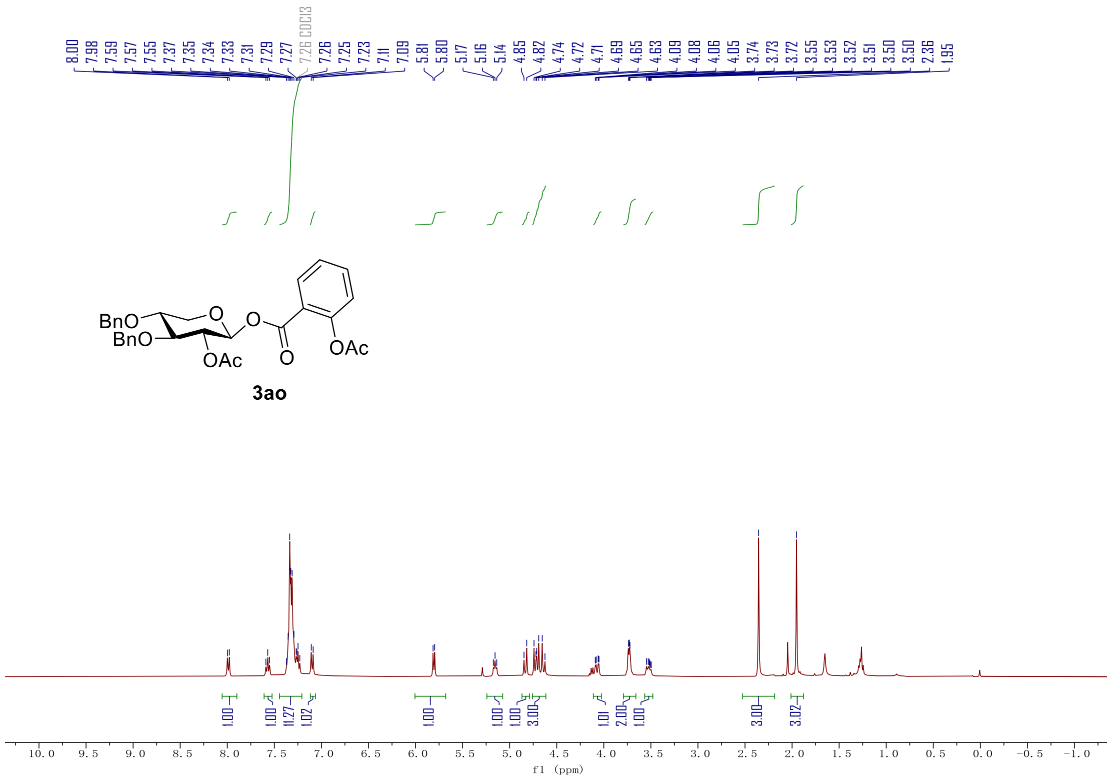

Supplementary Figure S196. $^{1}H$ NMR (400 MHz, $CDCl_3$) Spectra for compound 3ao

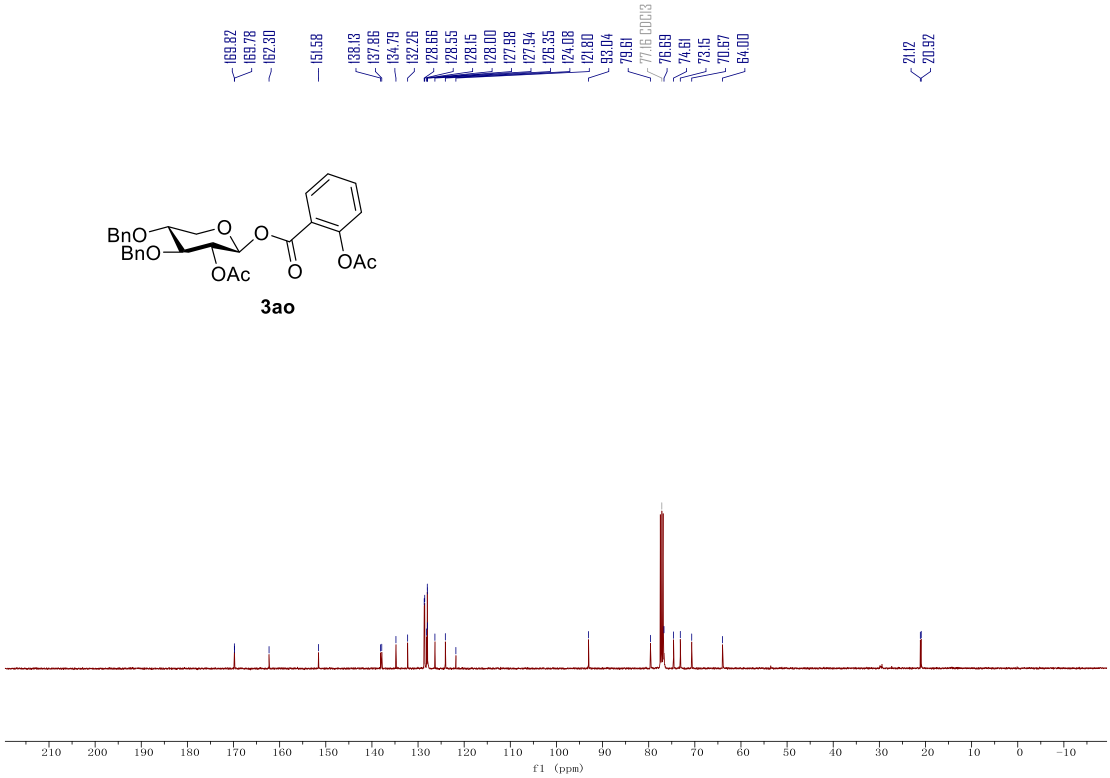

Supplementary Figure S197. $^{13}C$ NMR (101 MHz, $CDCl_3$) Spectra for compound 3ao

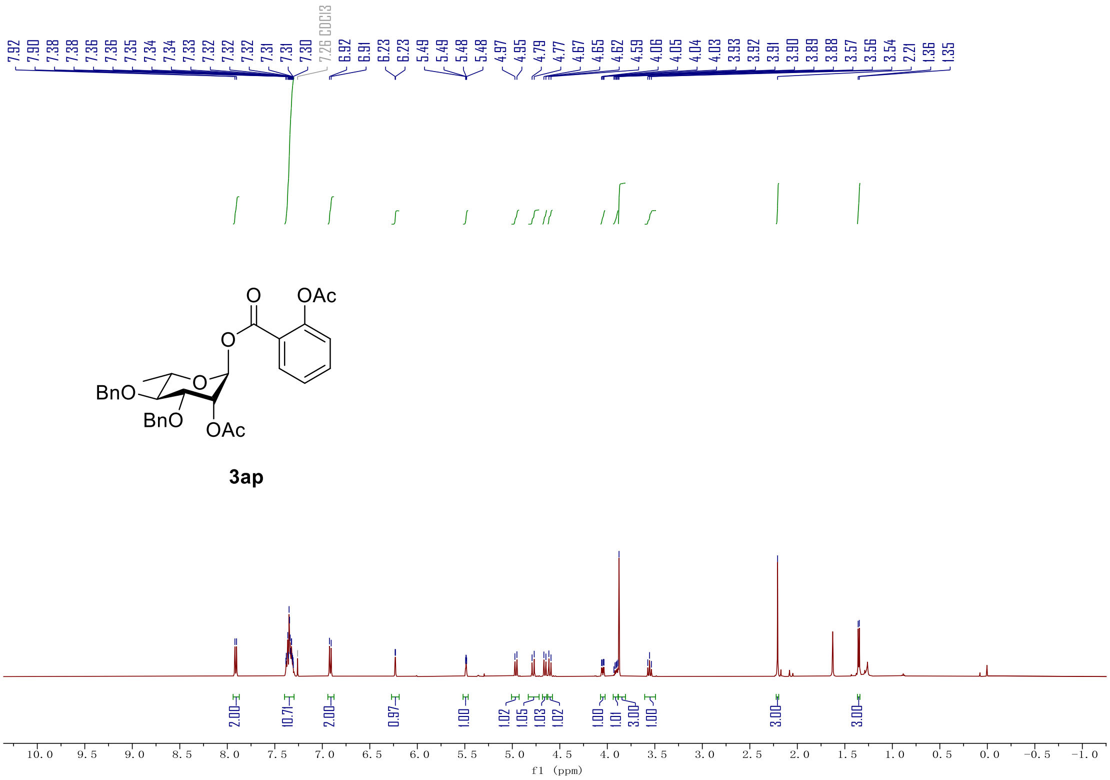


**Supplementary Figure S198. $^{1}H$ NMR (400 MHz, $CDCl_3$) Spectra for compound 3ap**

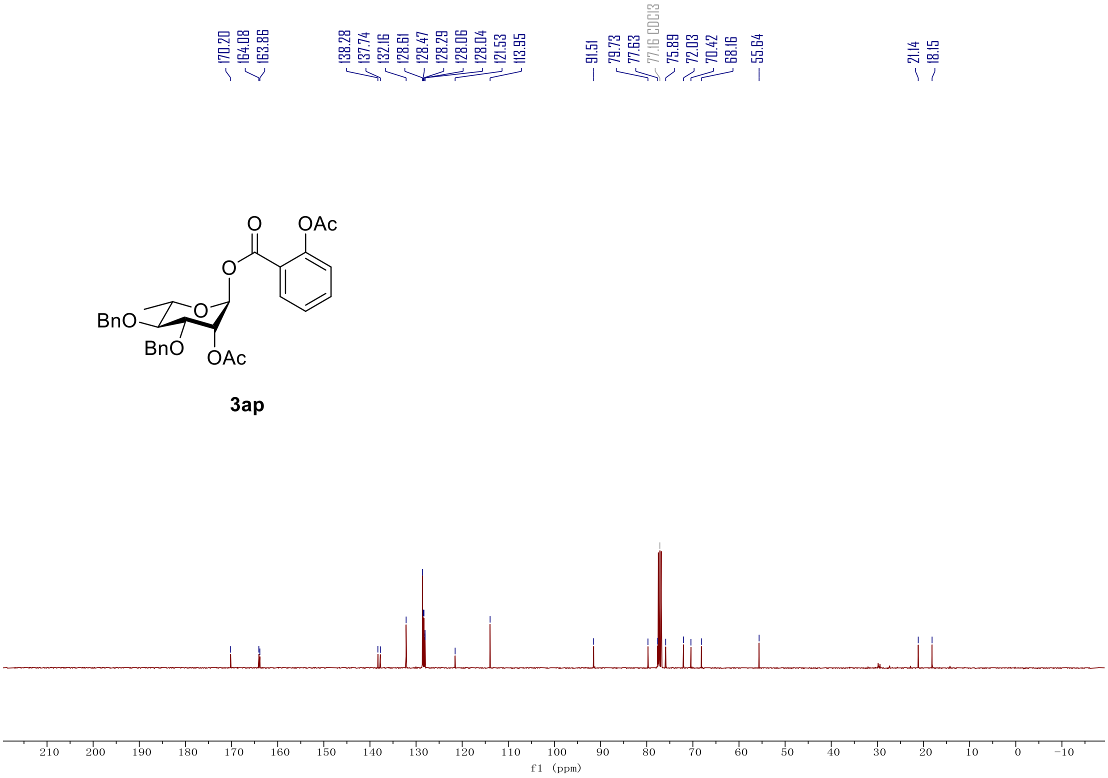


**Supplementary Figure S199. $^{13}C$ NMR (101 MHz, $CDCl_3$) Spectra for compound 3ap**

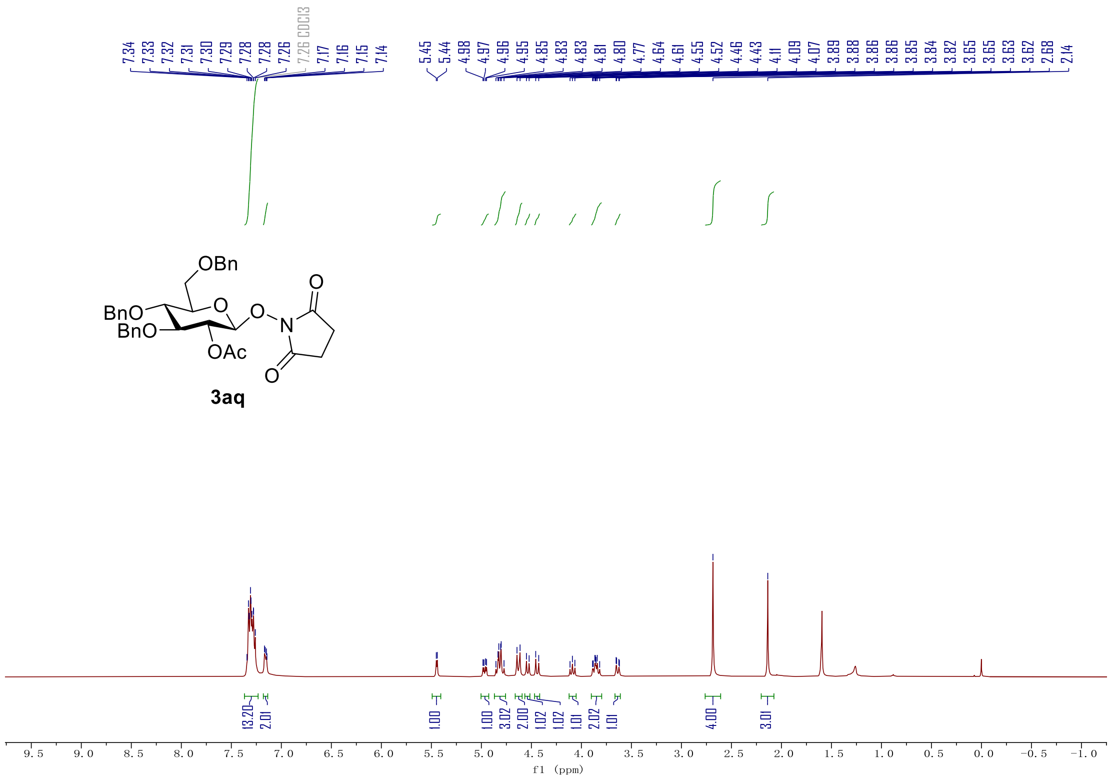


**Supplementary Figure S200.** **$^{1}$H NMR (400 MHz, $CDCl_3$) Spectra for compound 3aq**

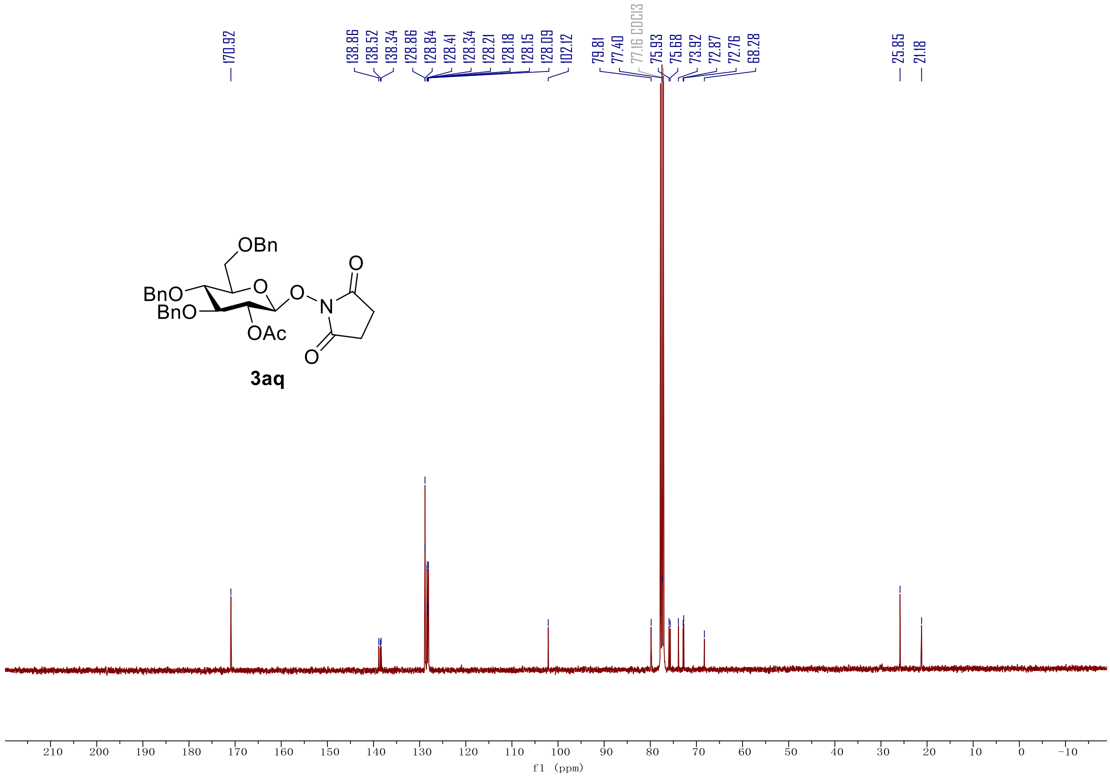


**Supplementary Figure S201.** **$^{13}$C NMR (101 MHz, $CDCl_3$) Spectra for compound 3aq**

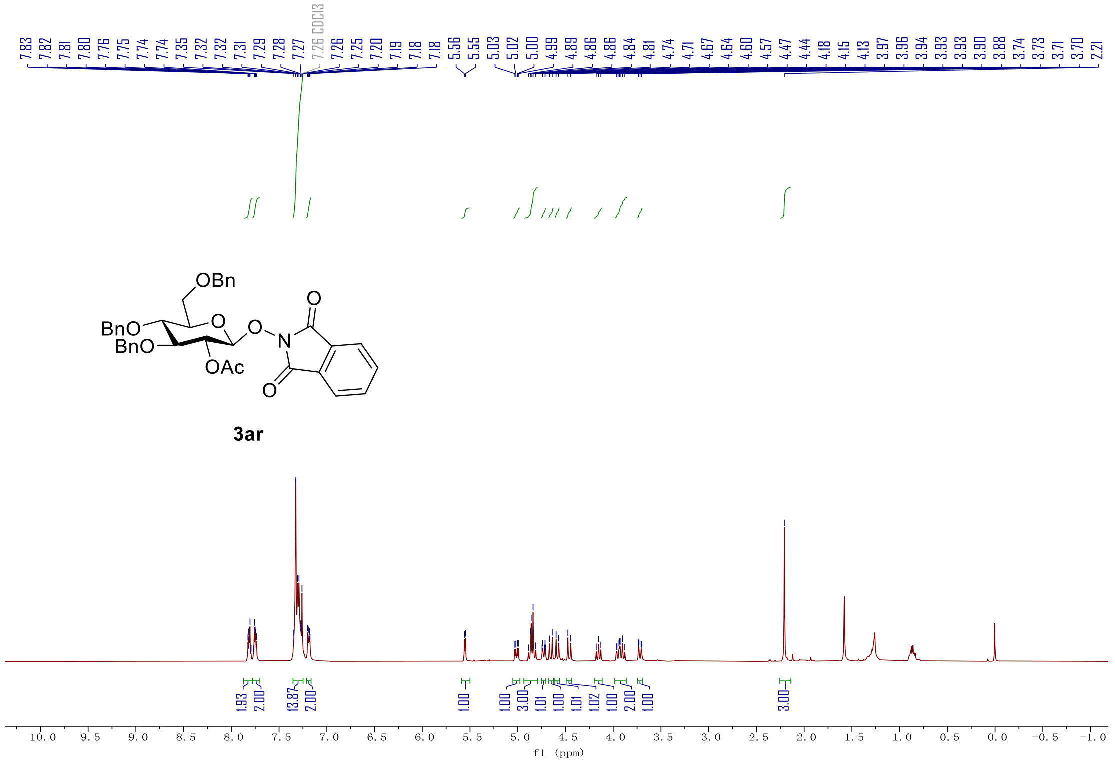


**Supplementary Figure S202. $^{1}$H NMR (400 MHz, $CDCl_3$) Spectra for compound 3ar**

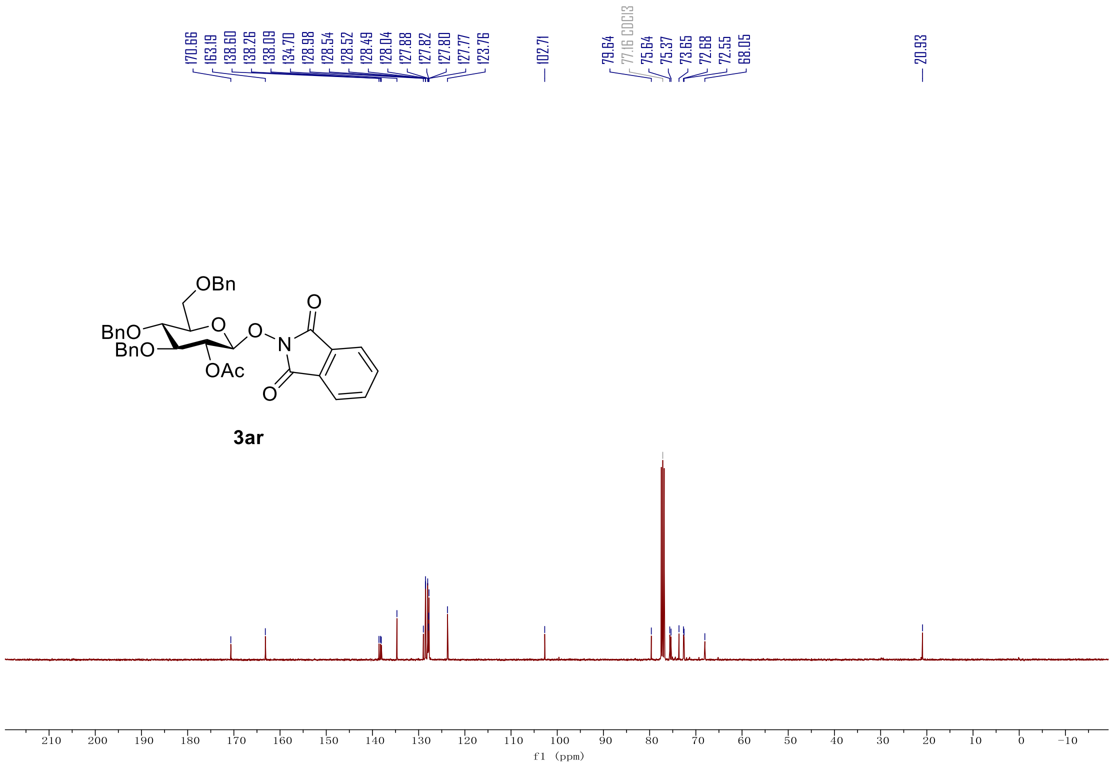


**Supplementary Figure S203. $^{13}$C NMR (101 MHz, $CDCl_3$) Spectra for compound 3ar**

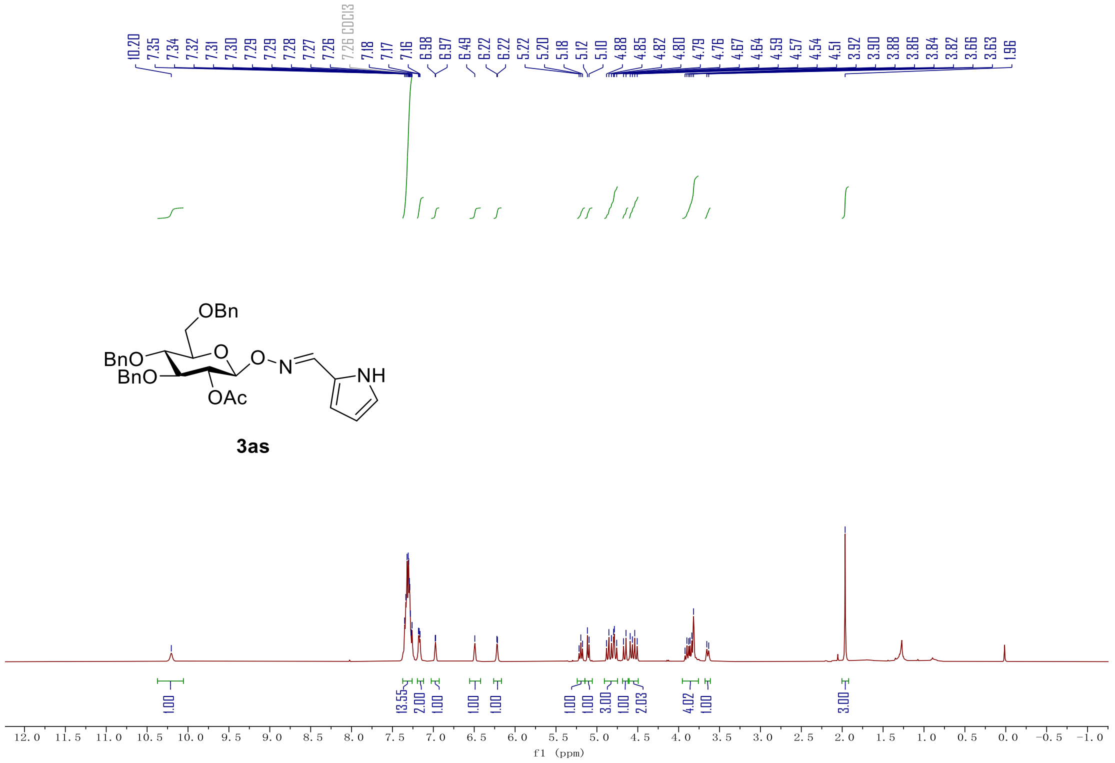


**Supplementary Figure S204. $^{1}$H NMR (400 MHz, $CDCl_3$) Spectra for compound 3as**

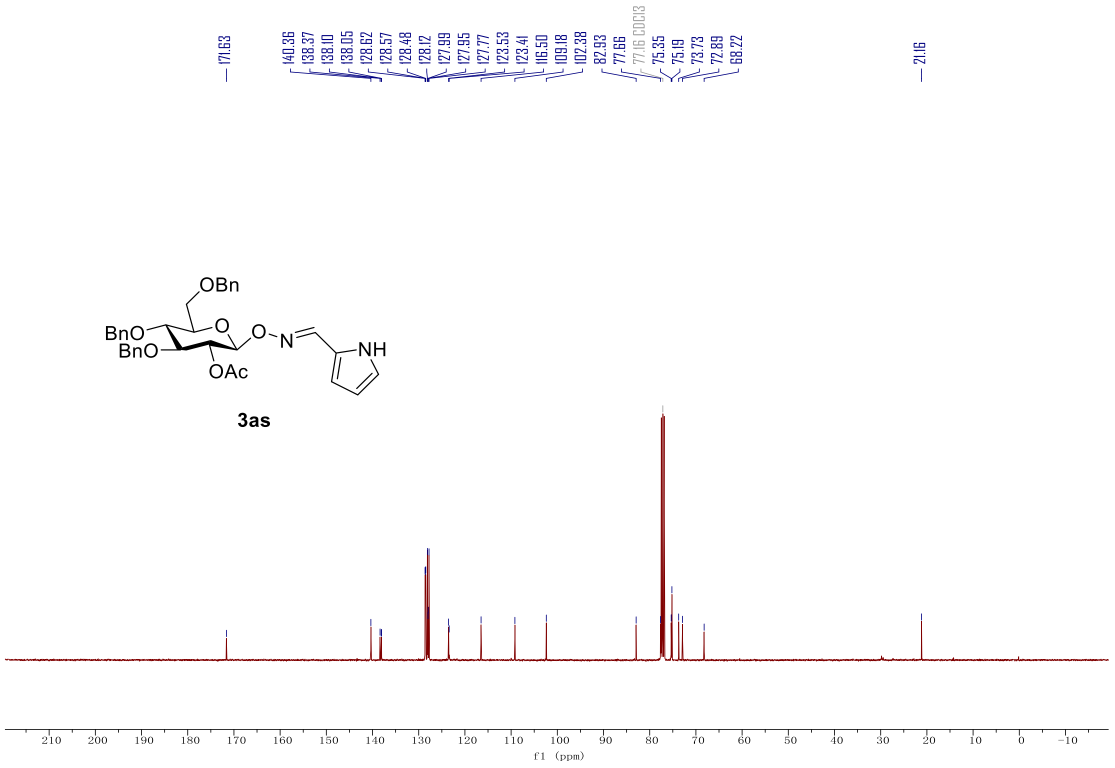


**Supplementary Figure S205. $^{13}$C NMR (101 MHz, $CDCl_3$) Spectra for compound 3as**

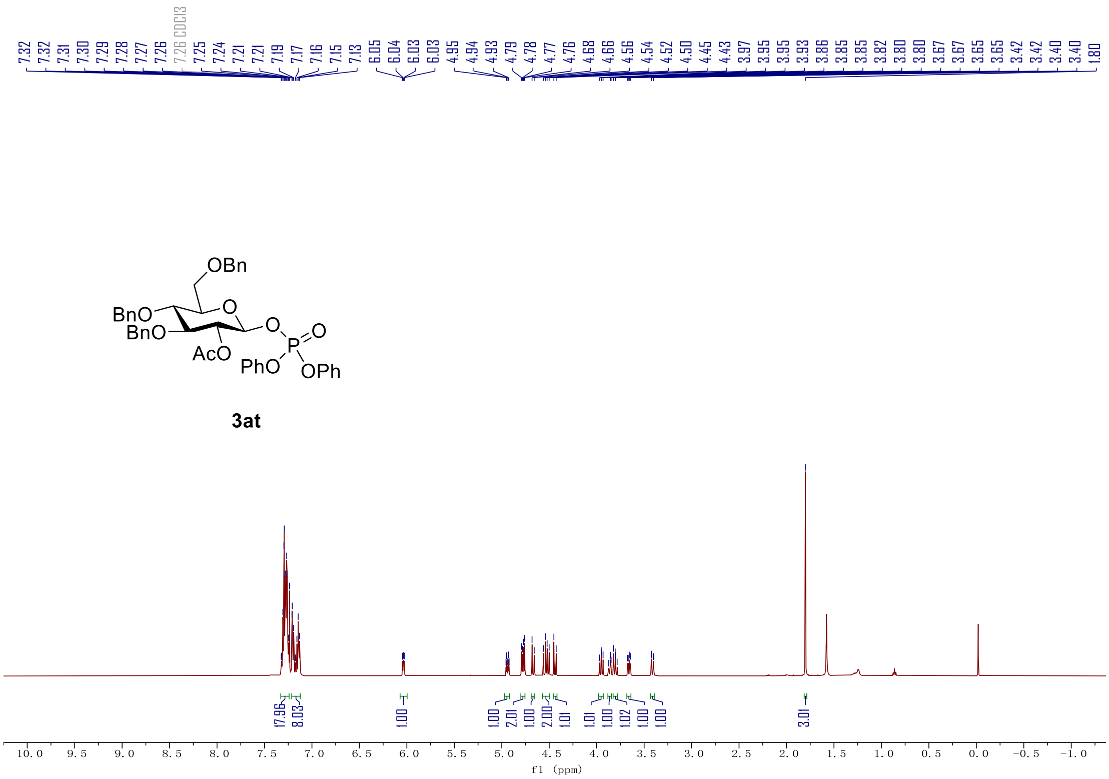


**Supplementary Figure S206. $^{1}H$ NMR (500 MHz, $CDCl_3$) Spectra for compound 3at**

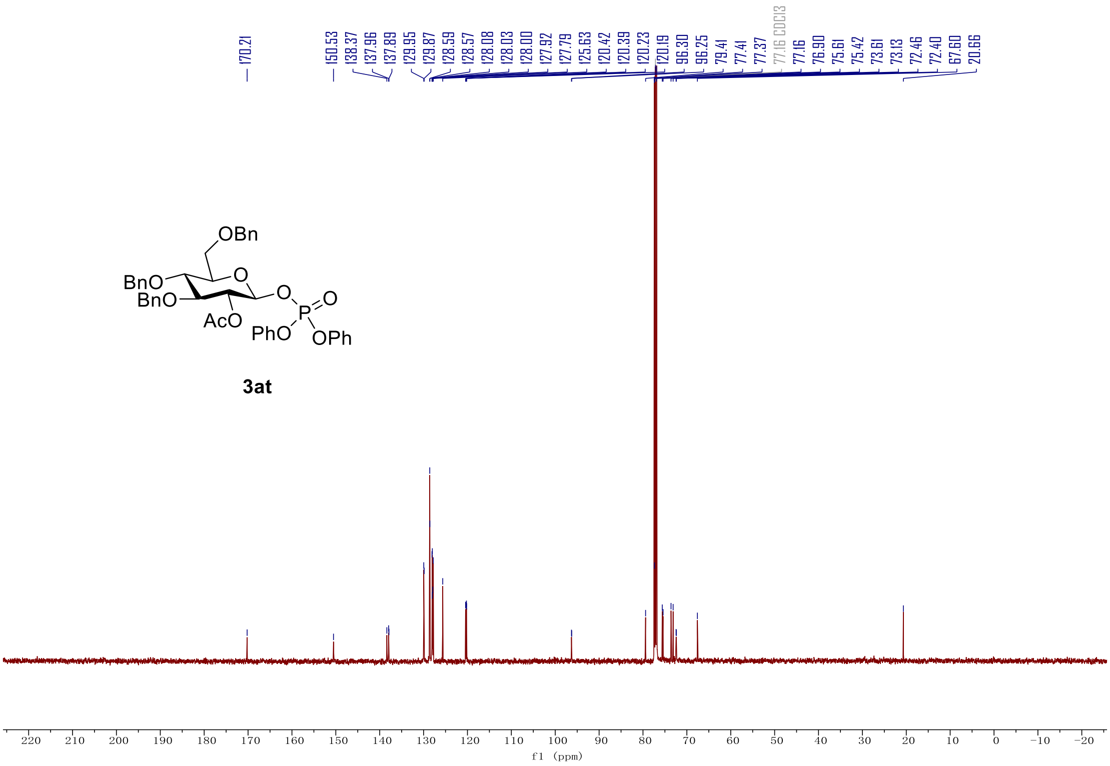


**Supplementary Figure S207. $^{13}C$ NMR (126 MHz, $CDCl_3$) Spectra for compound 3at**

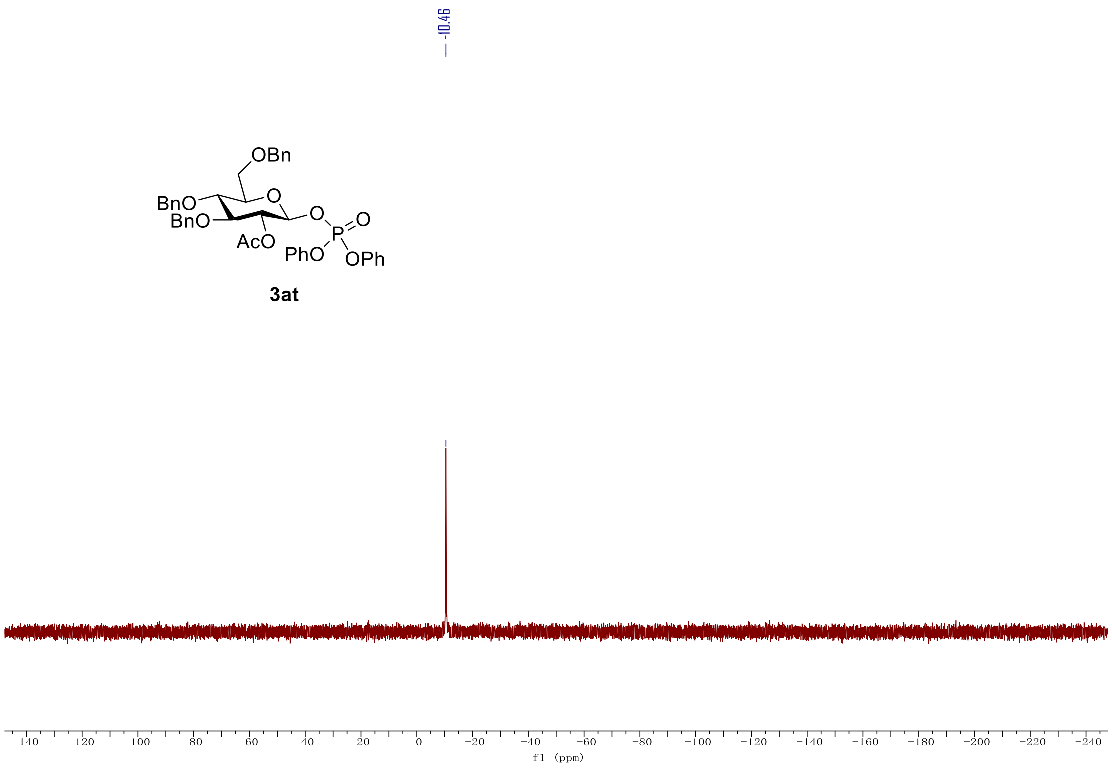


**Supplementary Figure S208. $^{31}$P NMR (162 MHz, $CDCl_3$) Spectra for crude compound 3at**

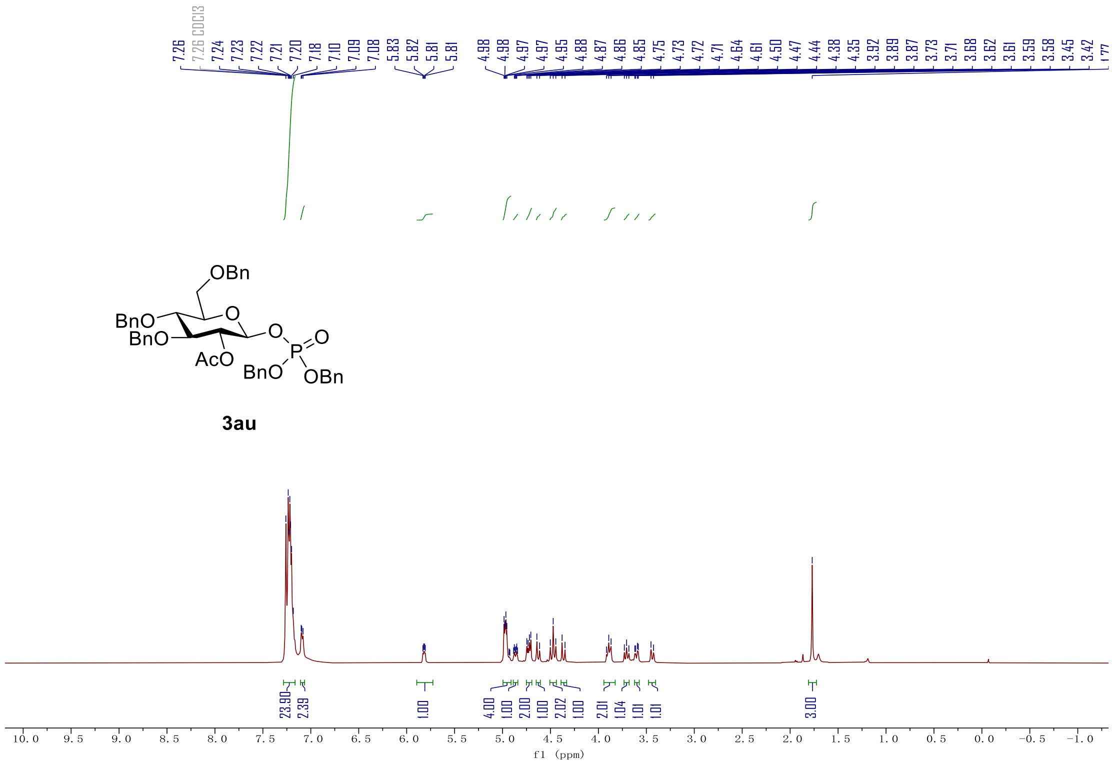


**Supplementary Figure S209. $^{1}$H NMR (400 MHz, $CDCl_3$) Spectra for compound 3au**

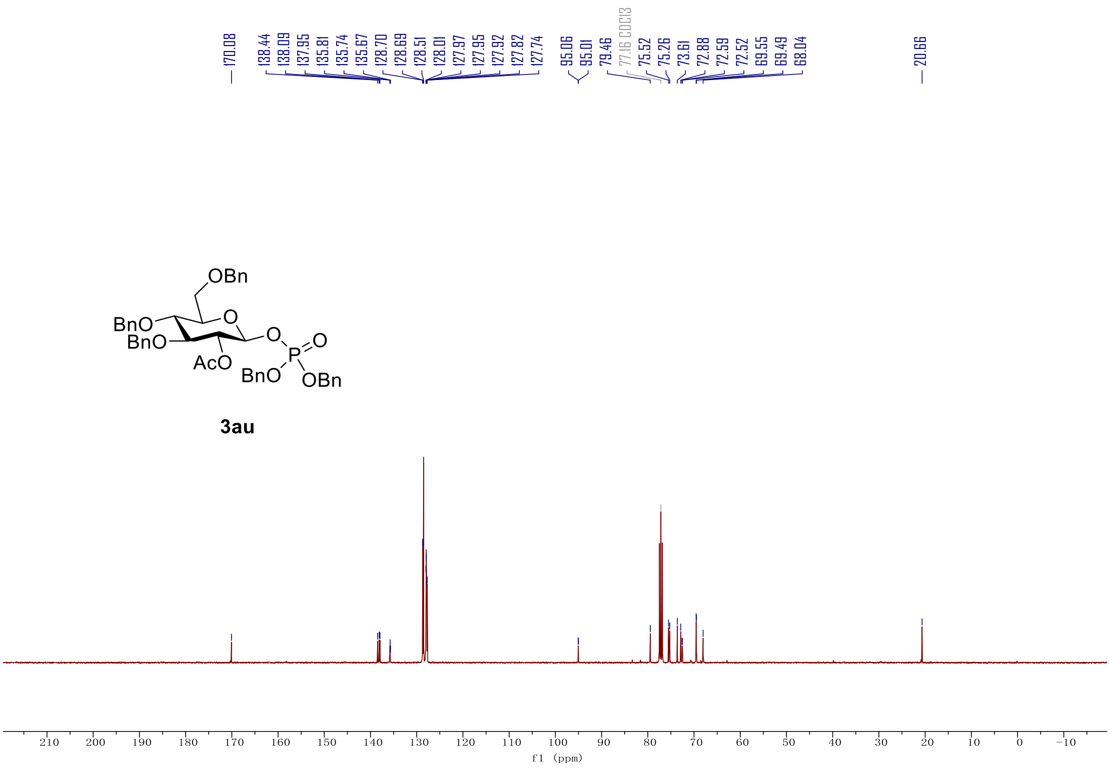


**Supplementary Figure S210.** $^{13}C$ **NMR (101 MHz,** $CDCl_3$**) Spectra for compound 3au**

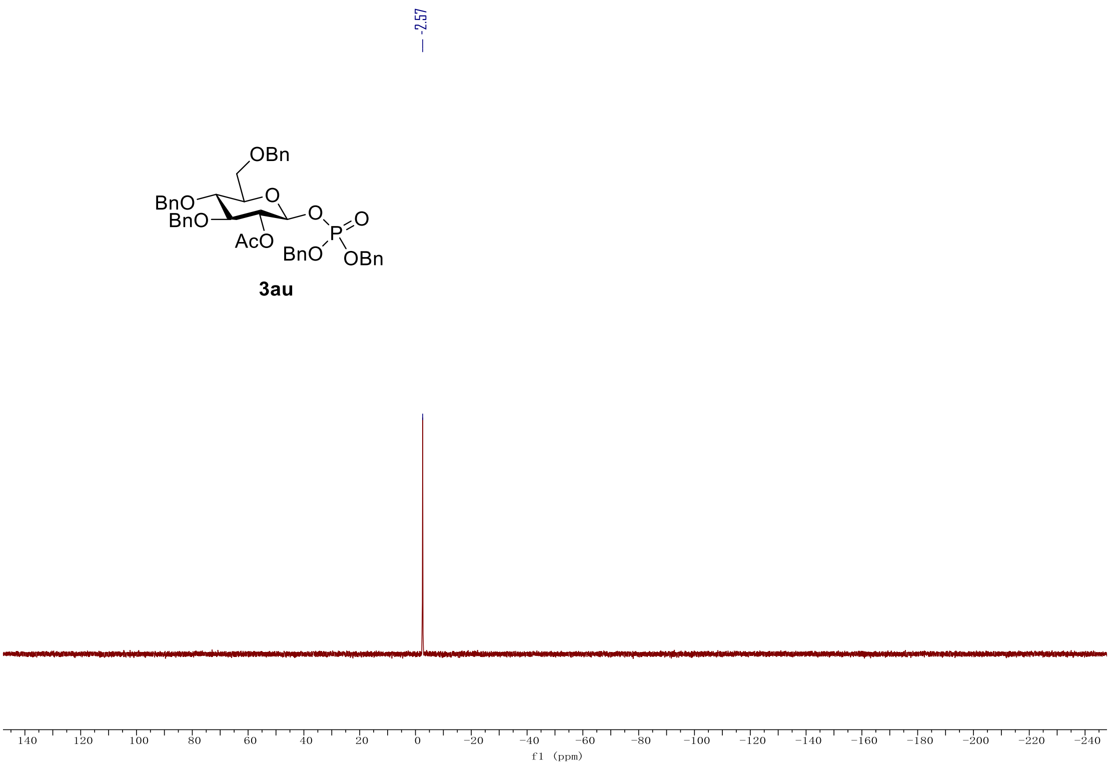


**Supplementary Figure S211.** $^{31}P$ **NMR (162 MHz,** $CDCl_3$**) Spectra for crude compound 3au**

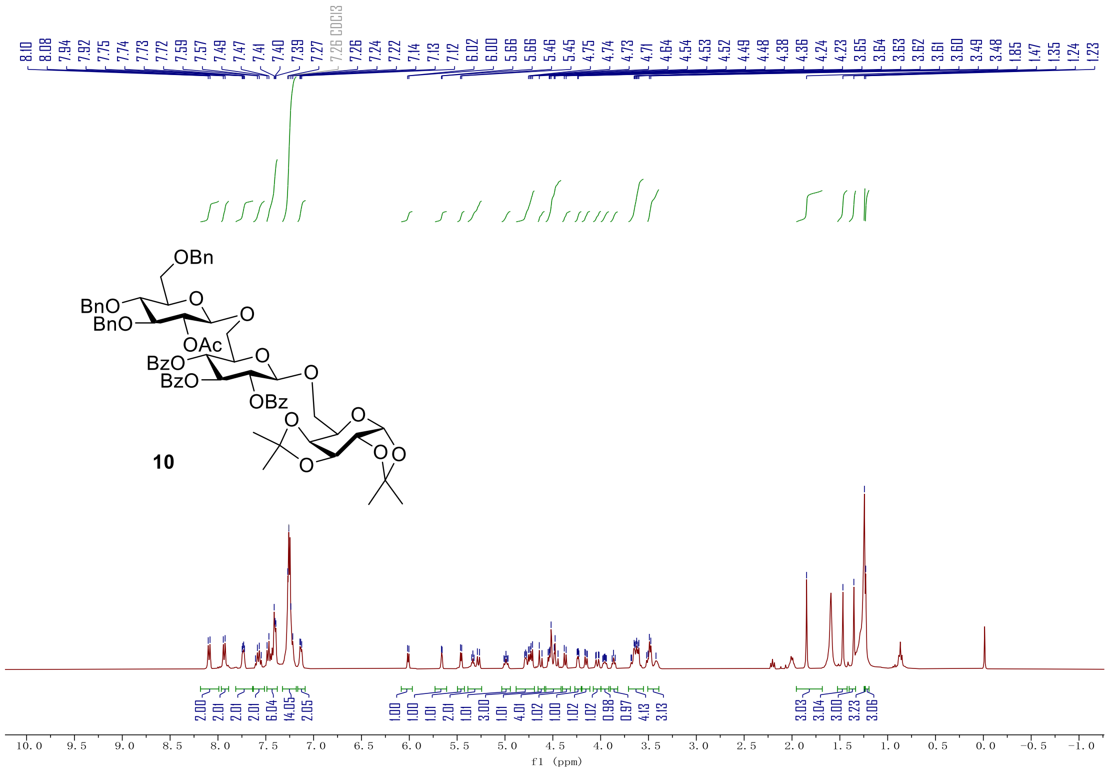


**Supplementary Figure S212.** $^{1}$**H NMR (400 MHz,** $CDCl_3$**) Spectra for compound 10**

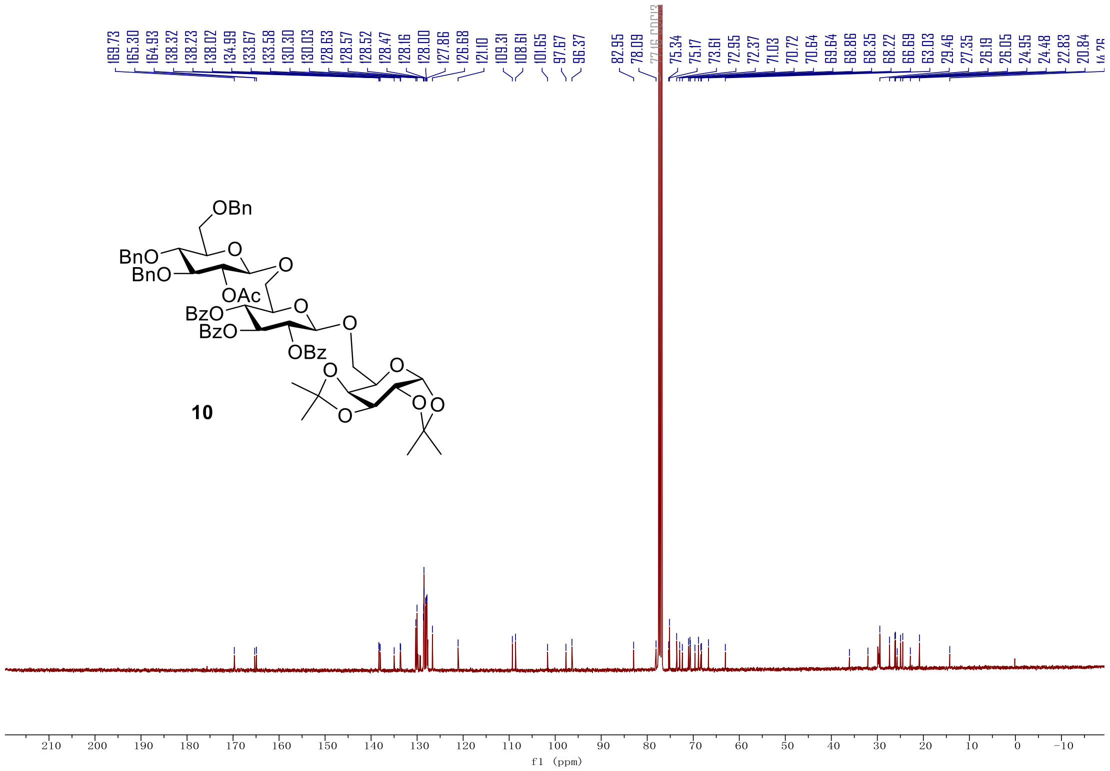


**Supplementary Figure S213.** $^{13}$**C NMR (101 MHz,** $CDCl_3$**) Spectra for compound 10**

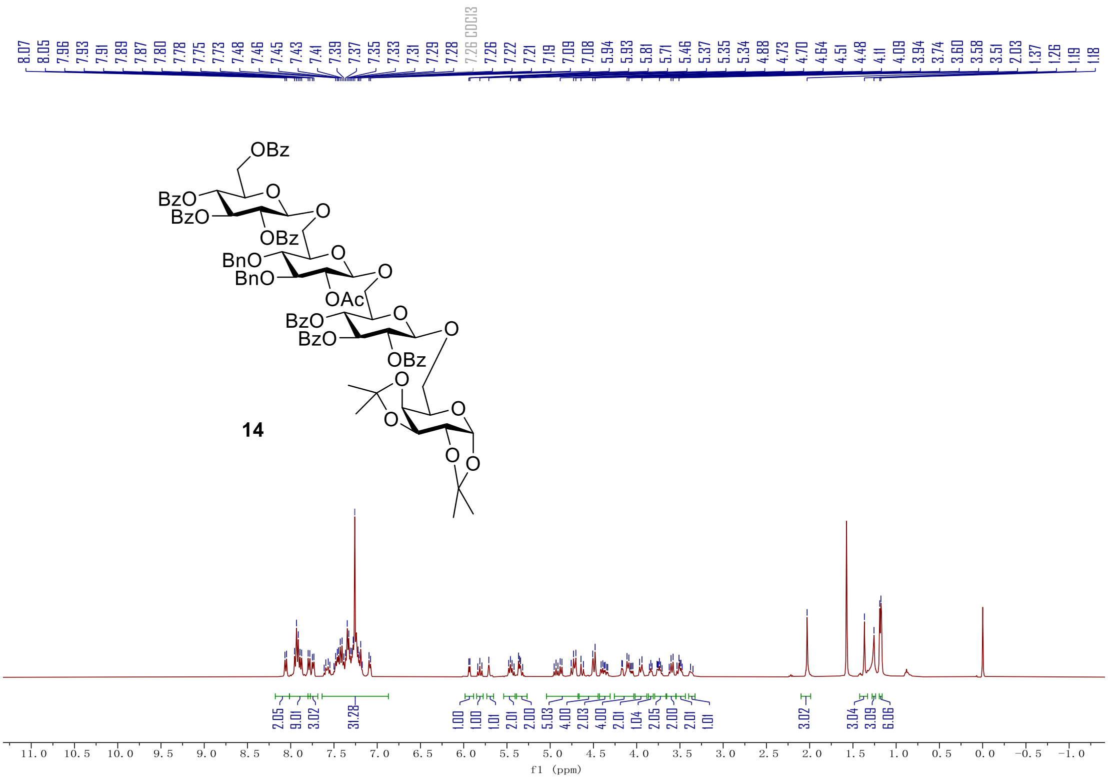


**Supplementary Figure S214.** $^{1}$**H NMR (400 MHz,** $CDCl_3$**) Spectra for compound 14**

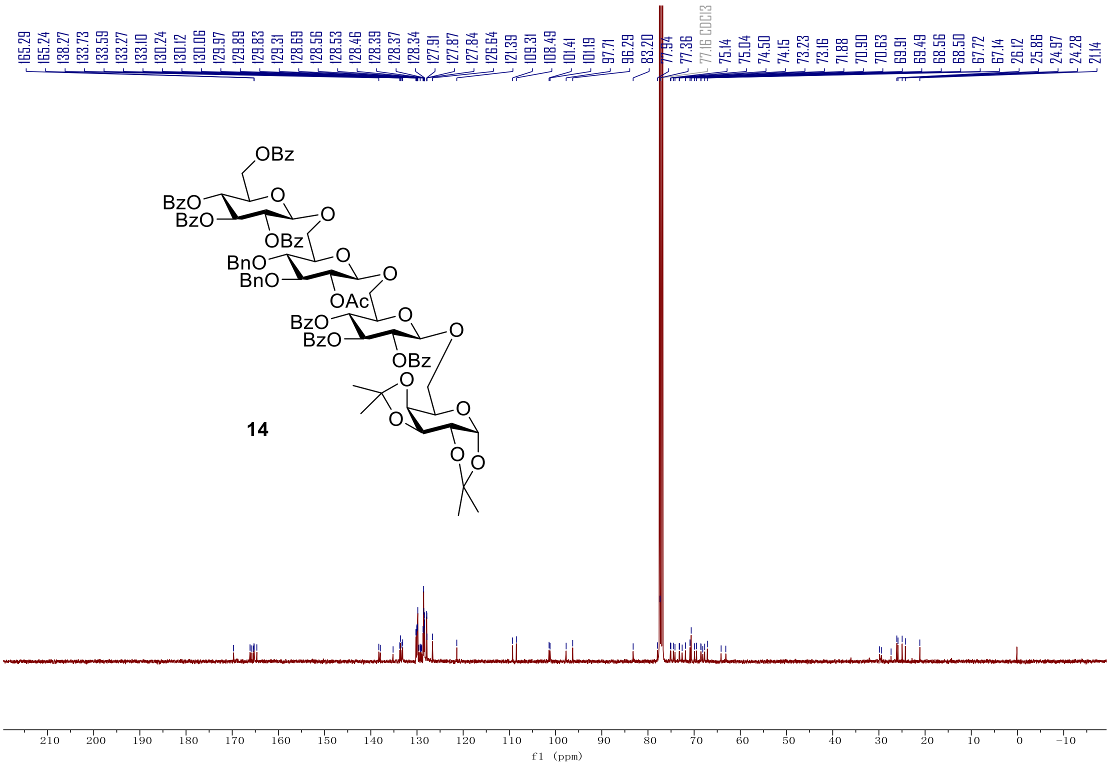


**Supplementary Figure S215.** $^{13}$**C NMR (101 MHz,** $CDCl_3$**) Spectra for compound 14**

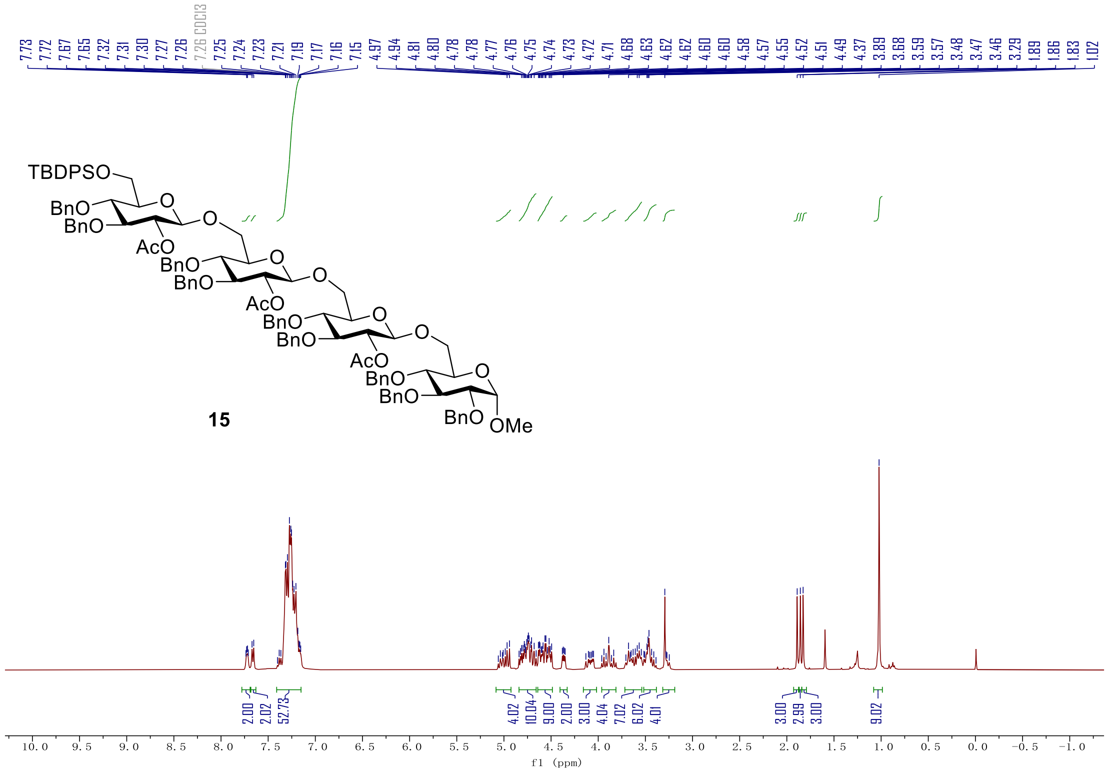

**Supplementary Figure S216. $^{1}$H NMR (400 MHz, $CDCl_3$) Spectra for compound 15**

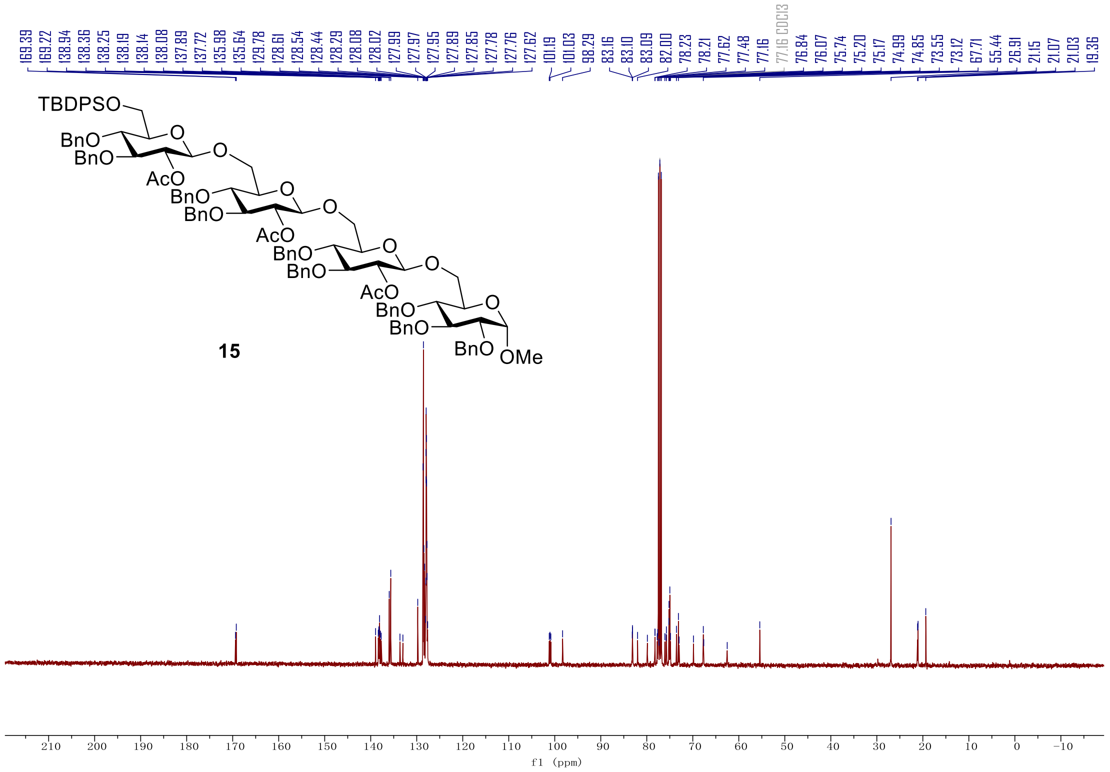

**Supplementary Figure S217. $^{13}$C NMR (101 MHz, $CDCl_3$) Spectra for compound 15**

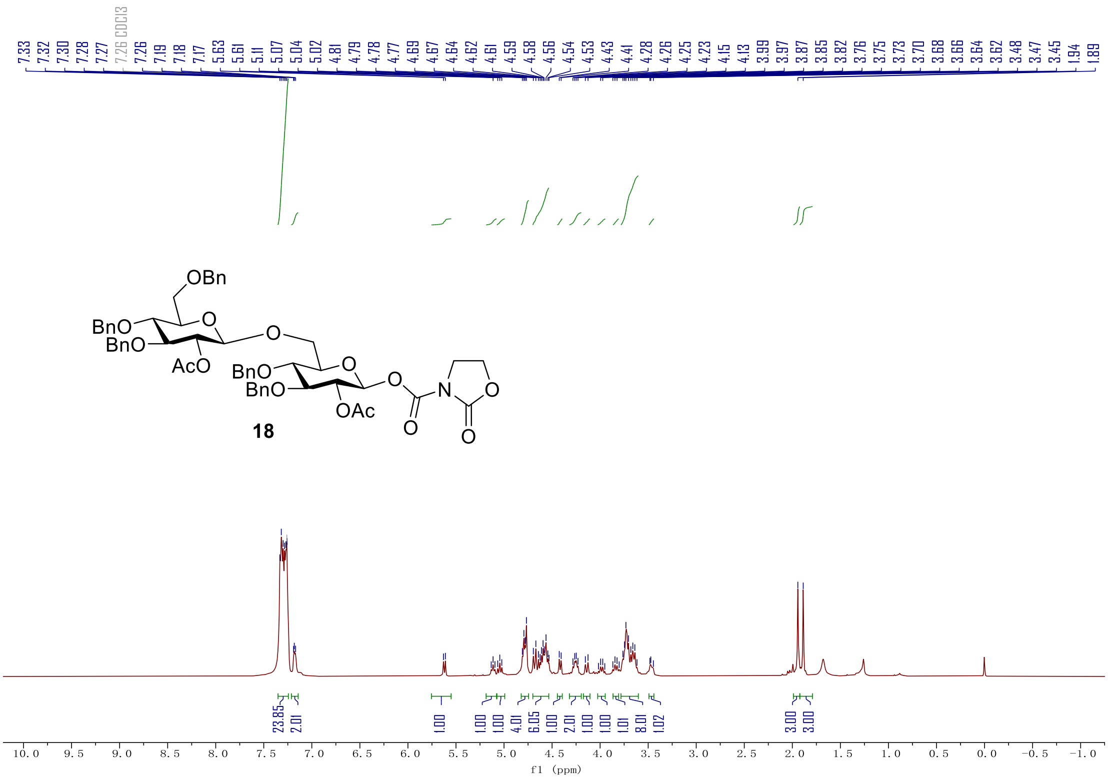


**Supplementary Figure S218.** $^{1}$**H NMR (400 MHz, CDCl$_3$) Spectra for compound 18**

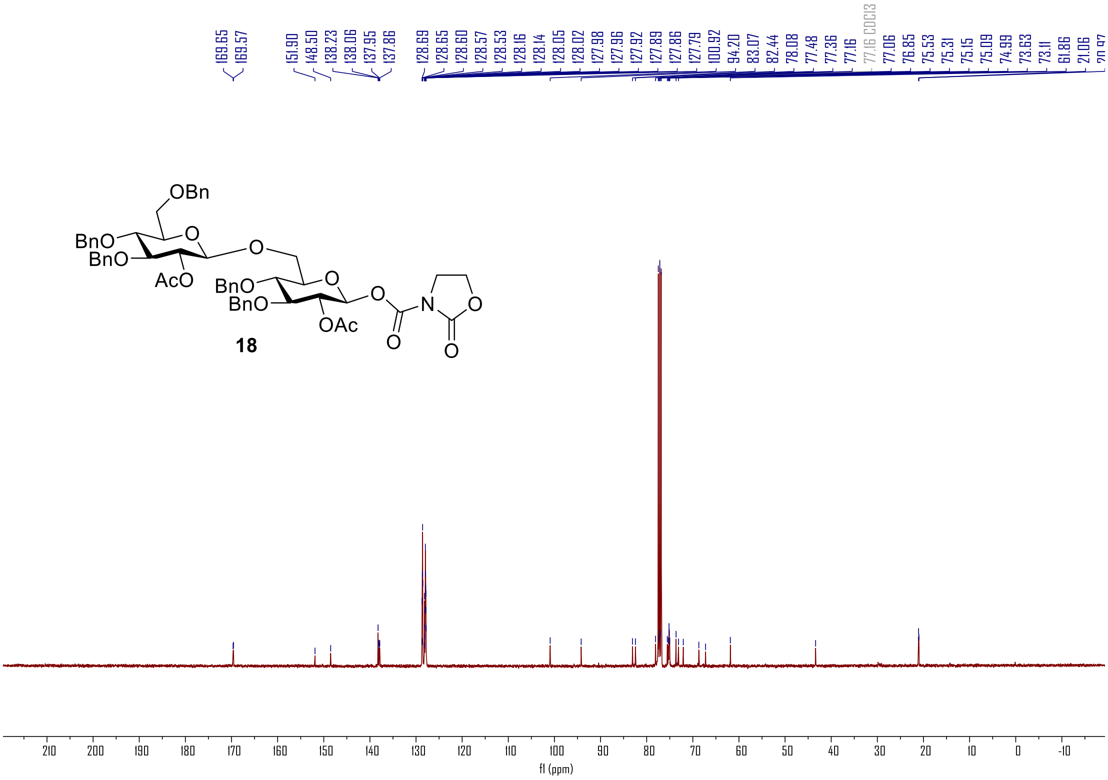


**Supplementary Figure S219.** $^{13}$**C NMR (101 MHz, CDCl$_3$) Spectra for compound 18**

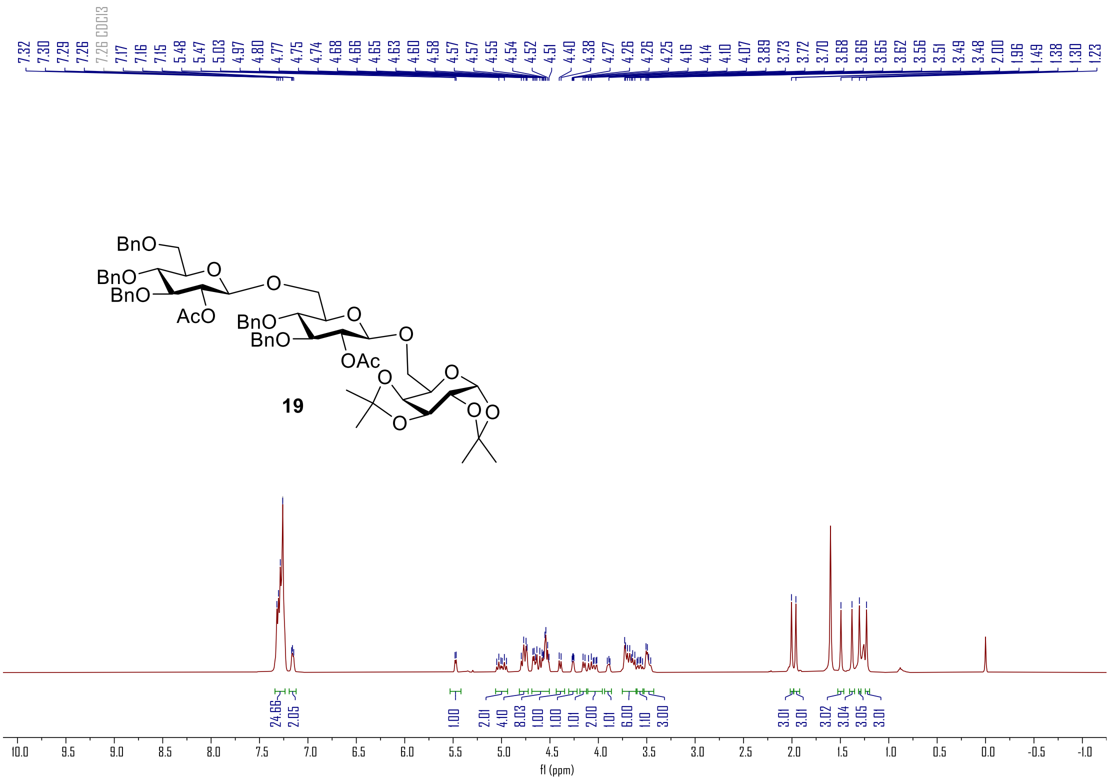


**Supplementary Figure S220. $^{1}$H NMR (400 MHz, $CDCl_3$) Spectra for compound 19**

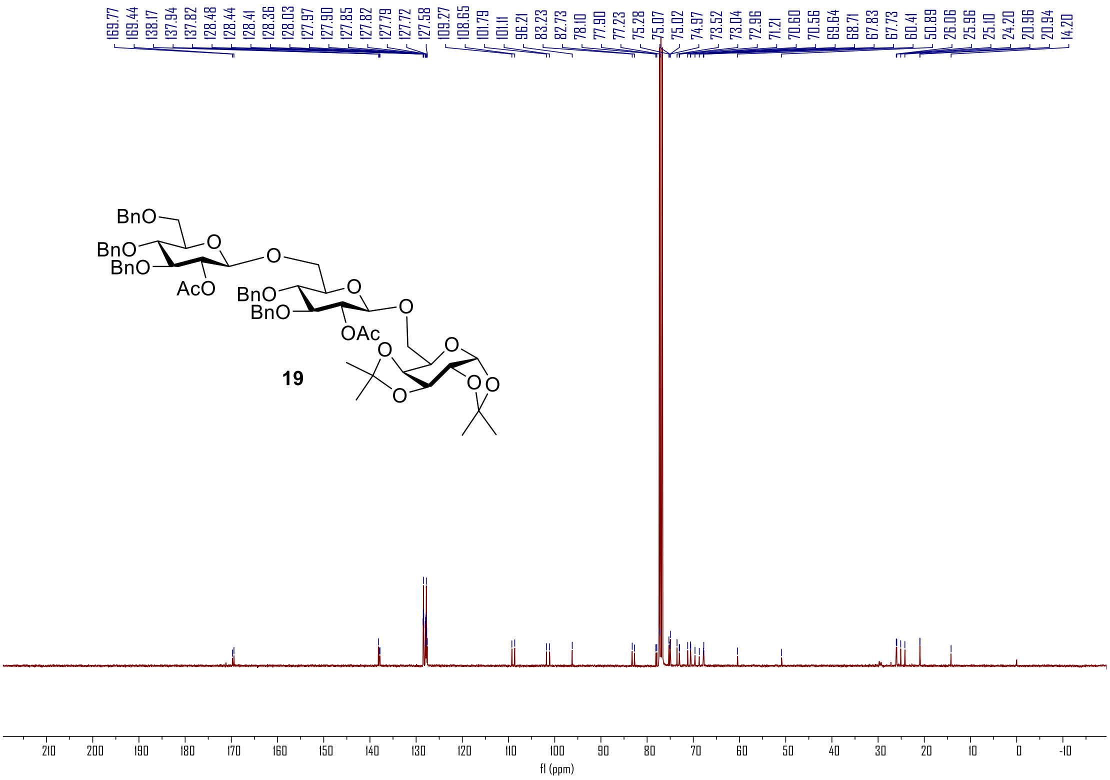


**Supplementary Figure S221. $^{13}$C NMR (101 MHz, $CDCl_3$) Spectra for compound 19**

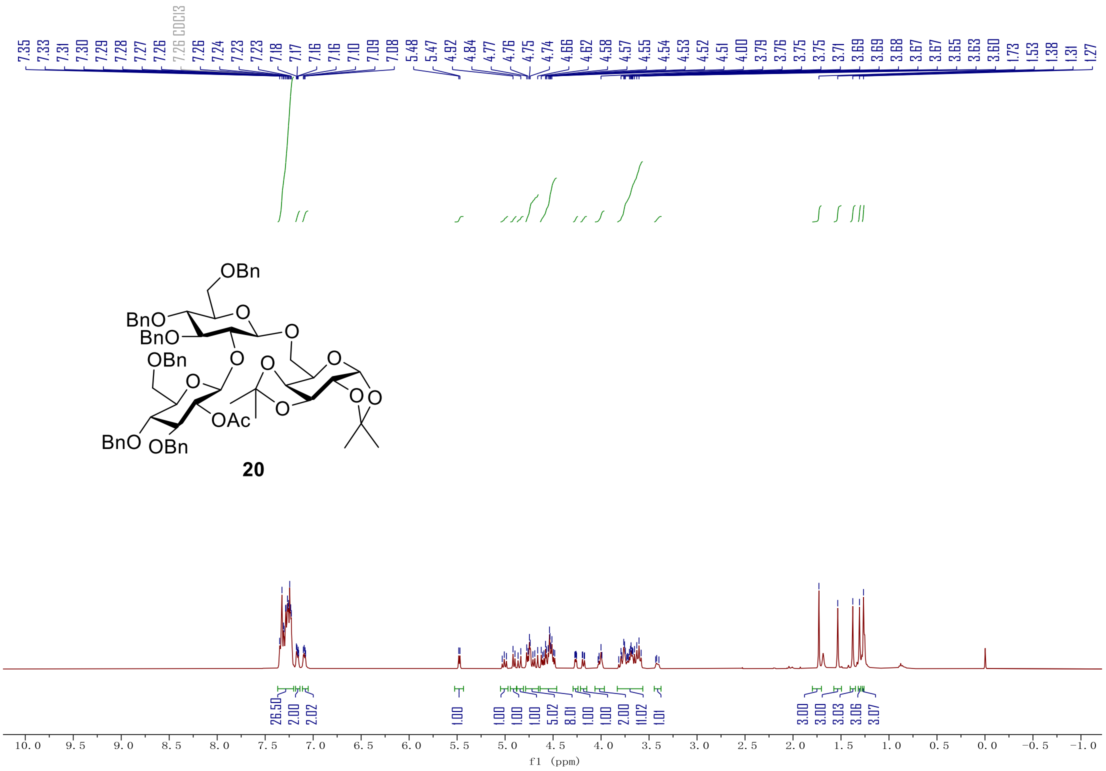


**Supplementary Figure S222. $^{1}H$ NMR (400 MHz, $CDCl_3$) Spectra for compound 20**

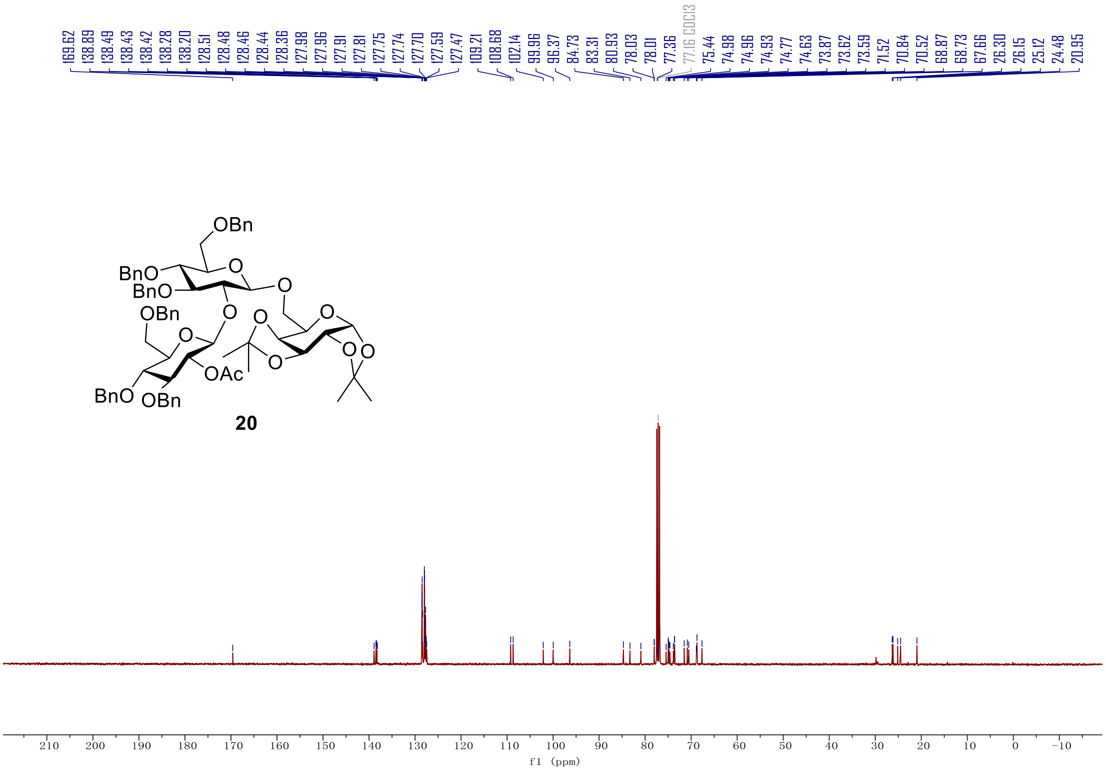


**Supplementary Figure S223. $^{13}C$ NMR (101 MHz, $CDCl_3$) Spectra for compound 20**

## 11.NMR evidence for the high β selectivity

Compound **3a (α and β mixture) and 3e-α** served as a reference for determining the chemical shift of the alpha anomeric signal, thus the high beta selectivity of the other glycoside products was established from the crude NMR spectrum of the reaction mixture.

### Synthesis of 3e-α

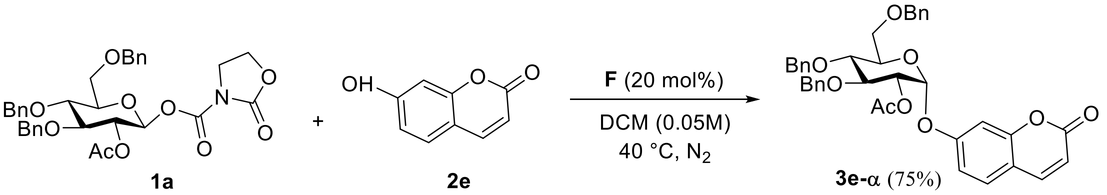


To an oven-dried vial was added glycosyl donor **1a** (45.4 mg, 0.075 mmol, 1.5 equiv.), acceptor **2e** (8.1 mg, 0.05 mmol, 1.0 equiv.), **F** (4.0 mg, 20 mol%) and anhydrous $CH_2Cl_2$ (1 mL, 0.05 M) under nitrogen atmosphere. The reaction mixture was stirred at 40 °C for 12 h and then purified by column chromatography on silica gel with petroleum ether/ethyl acetate (6:1) as eluent to afford **3e-α** as a white solid (23.8 mg, 75% yield).

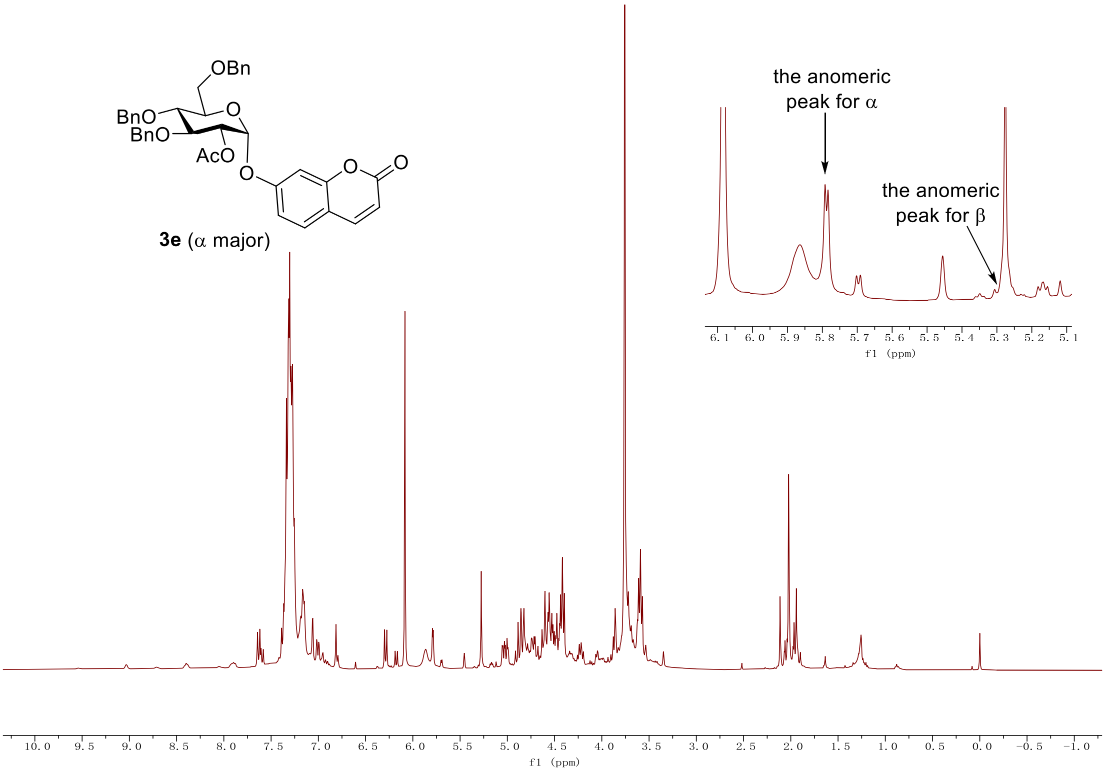


**Supplementary Figure S224. $^1$H NMR (400 MHz, $CDCl_3$) Spectra for crude compound 3e-α**

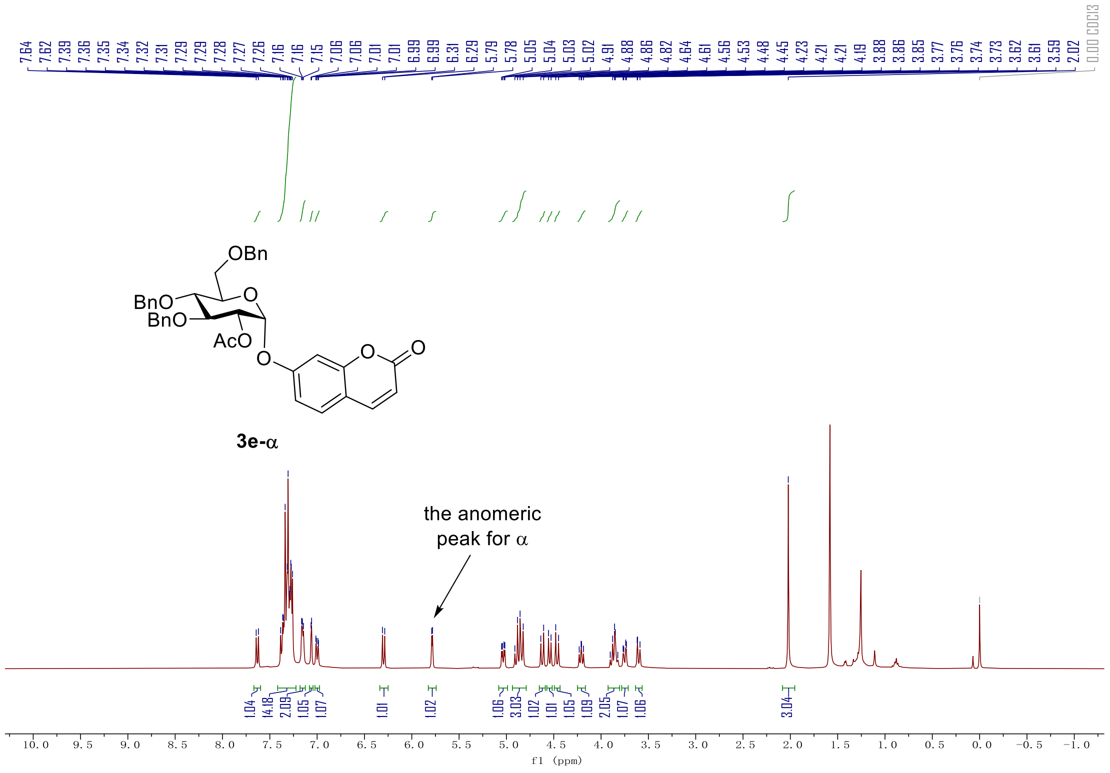


**Supplementary Figure S225. $^{1}$H NMR (400 MHz, $CDCl_3$) Spectra for compound 3e-α**

**Representative crude NMR spectra of the glycosylation reaction mixtures**

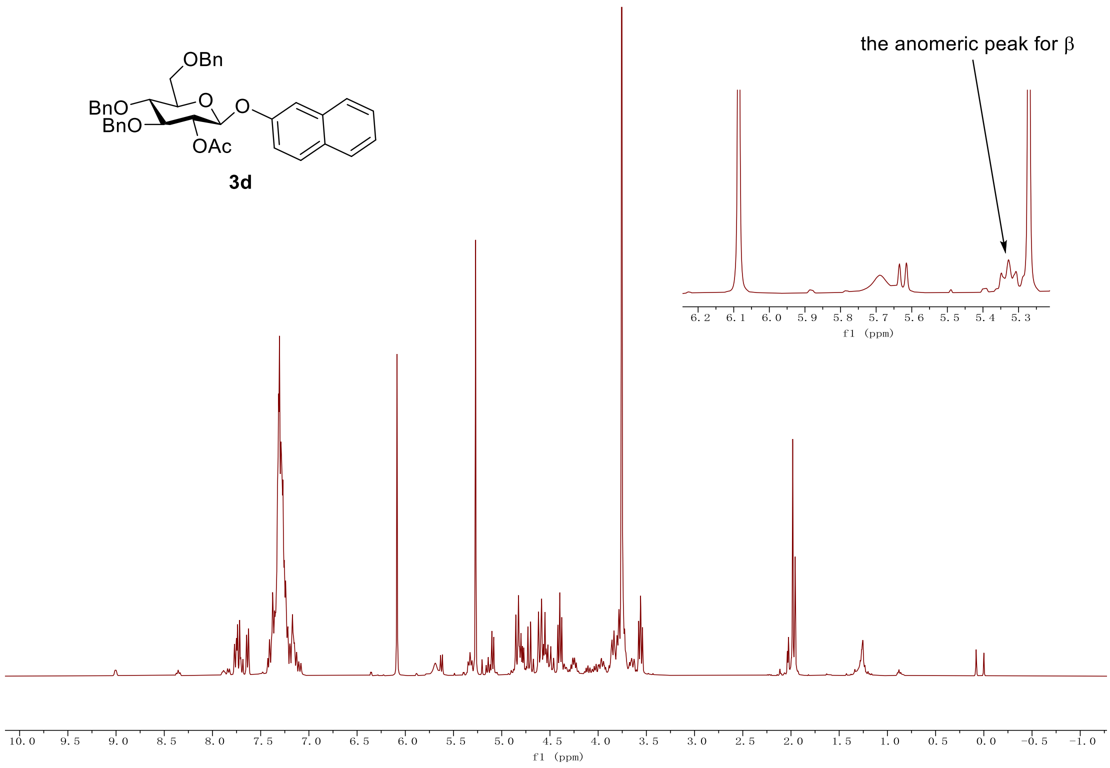


**Supplementary Figure S226. $^{1}$H NMR (400 MHz, $CDCl_3$) Spectra for crude compound 3d**

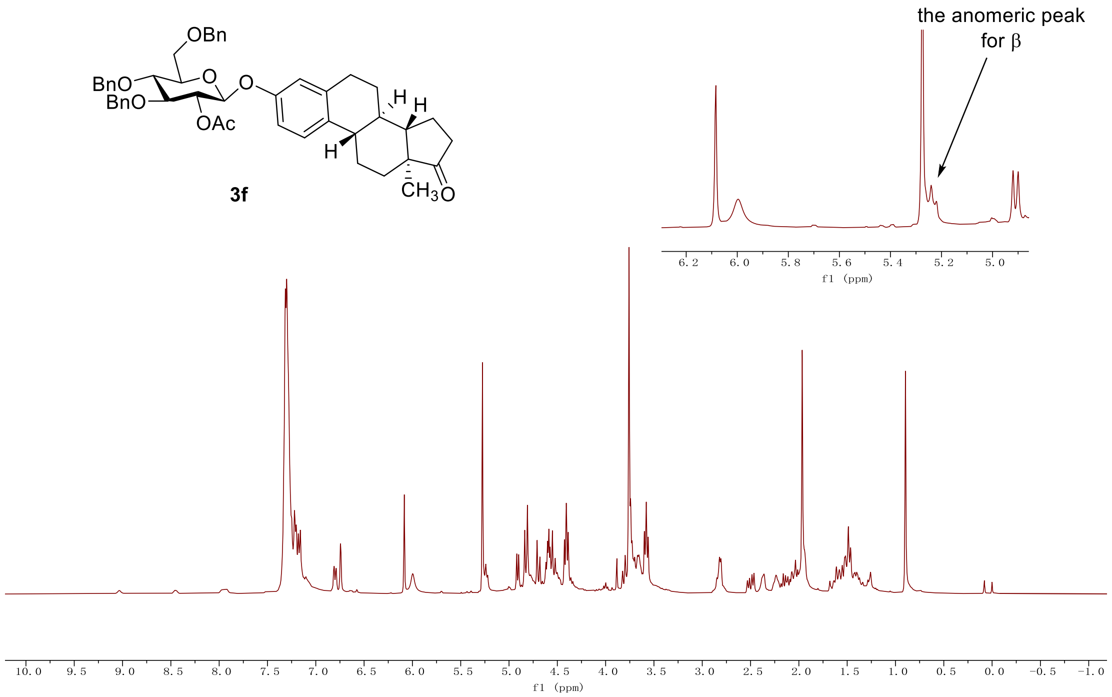


**Supplementary Figure S227.** $^{1}$**H NMR (400 MHz, CDCl$_3$) Spectra for crude compound 3f**

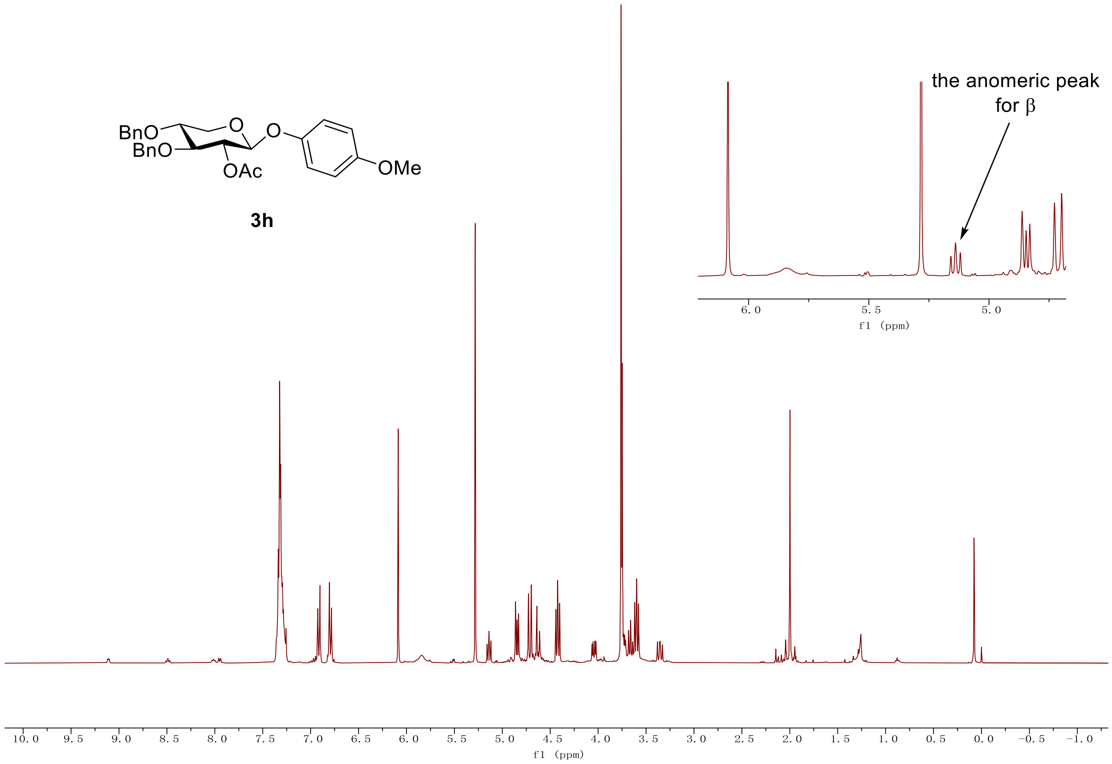


**Supplementary Figure S228.** $^{1}$**H NMR (400 MHz, CDCl$_3$) Spectra for crude compound 3h**

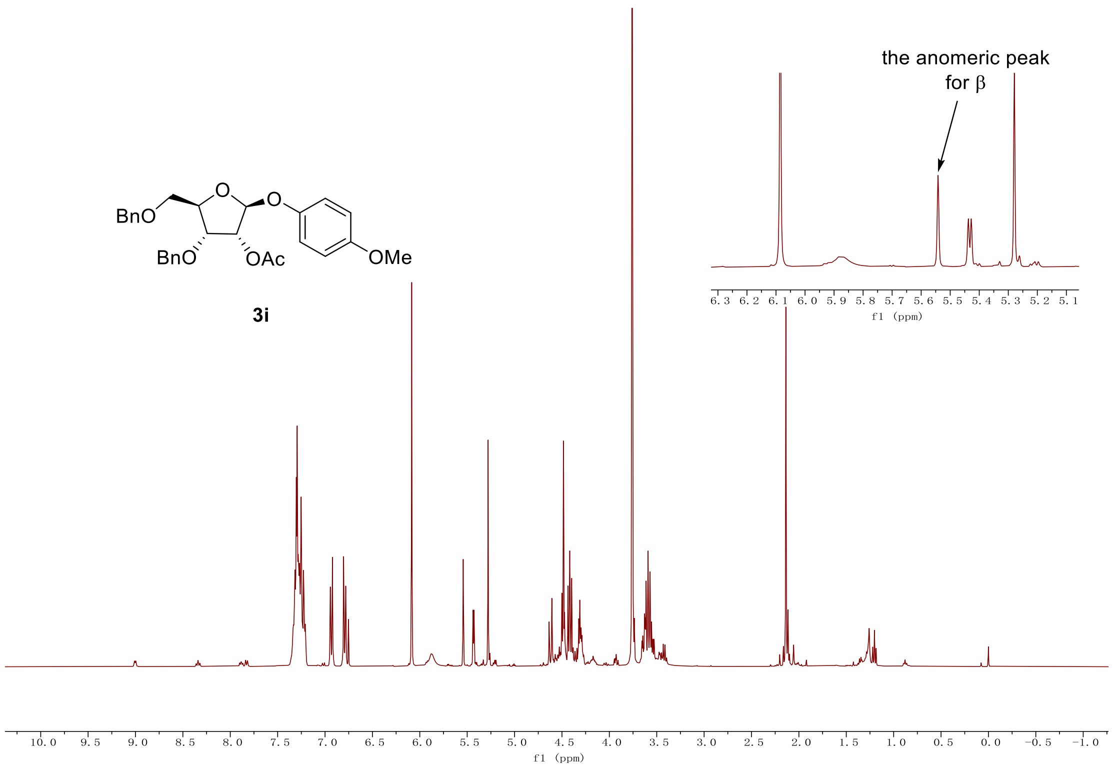


**Supplementary Figure S229. $^{1}$H NMR (400 MHz, $CDCl_3$) Spectra for crude compound 3i**

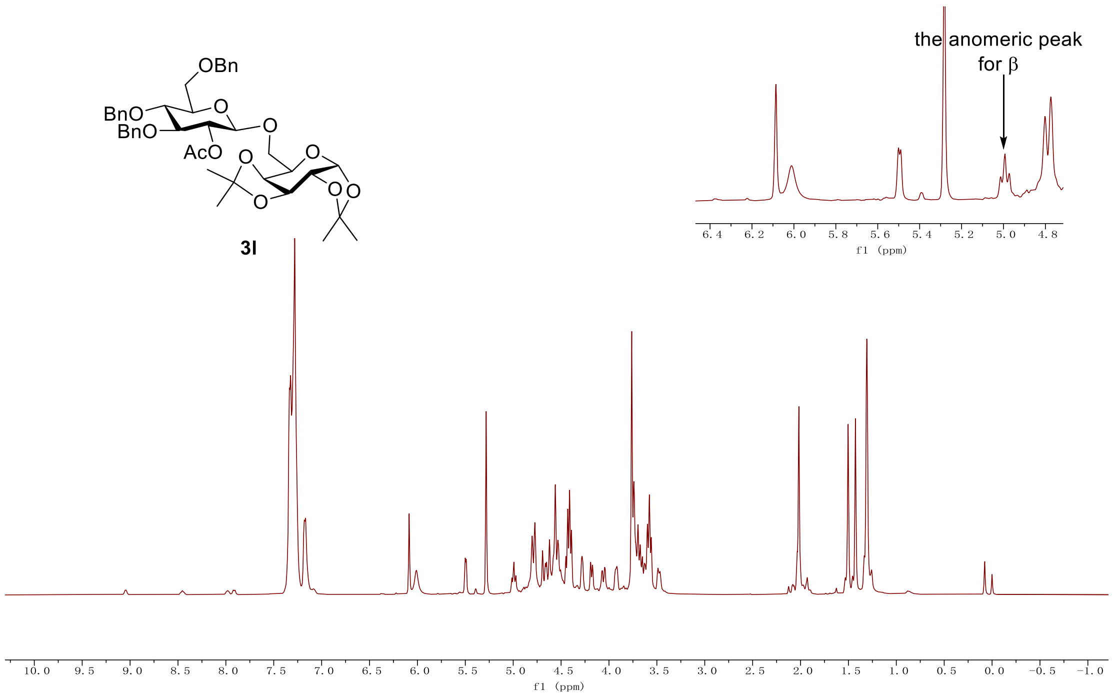


**Supplementary Figure S230. $^{1}$H NMR (400 MHz, $CDCl_3$) Spectra for crude compound 3l**

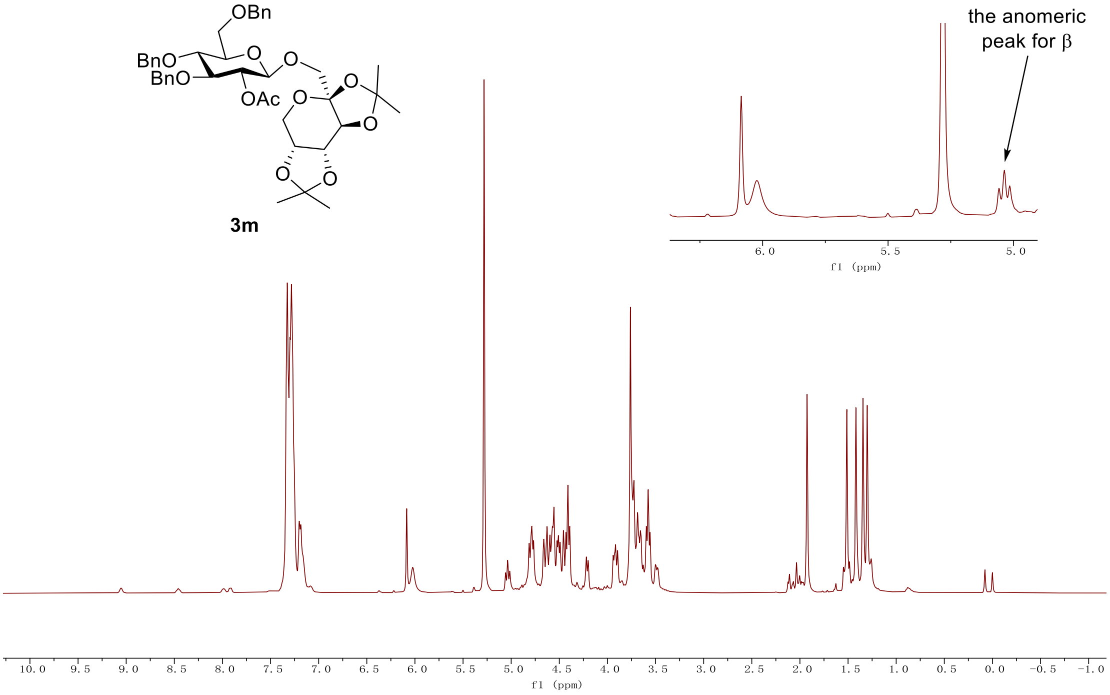


**Supplementary Figure S231. $^{1}$H NMR (400 MHz, CDCl3) Spectra for crude compound 3m**

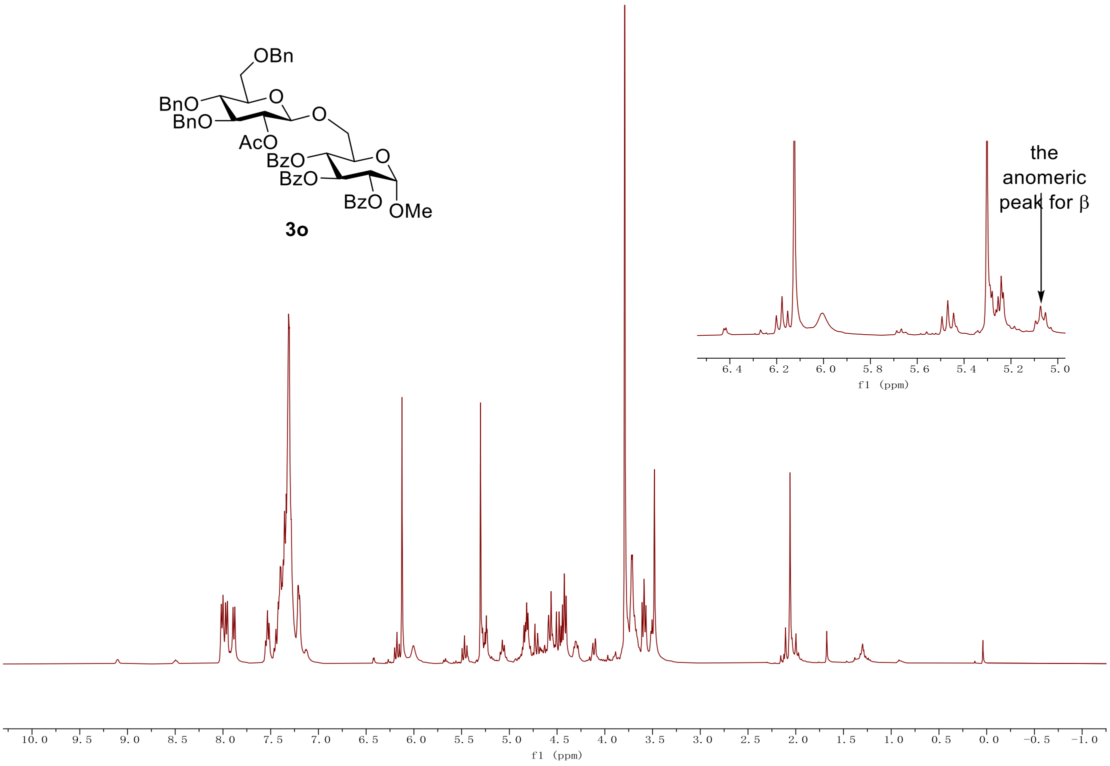


**Supplementary Figure S232. $^{1}$H NMR (400 MHz, CDCl3) Spectra for crude compound 3o**

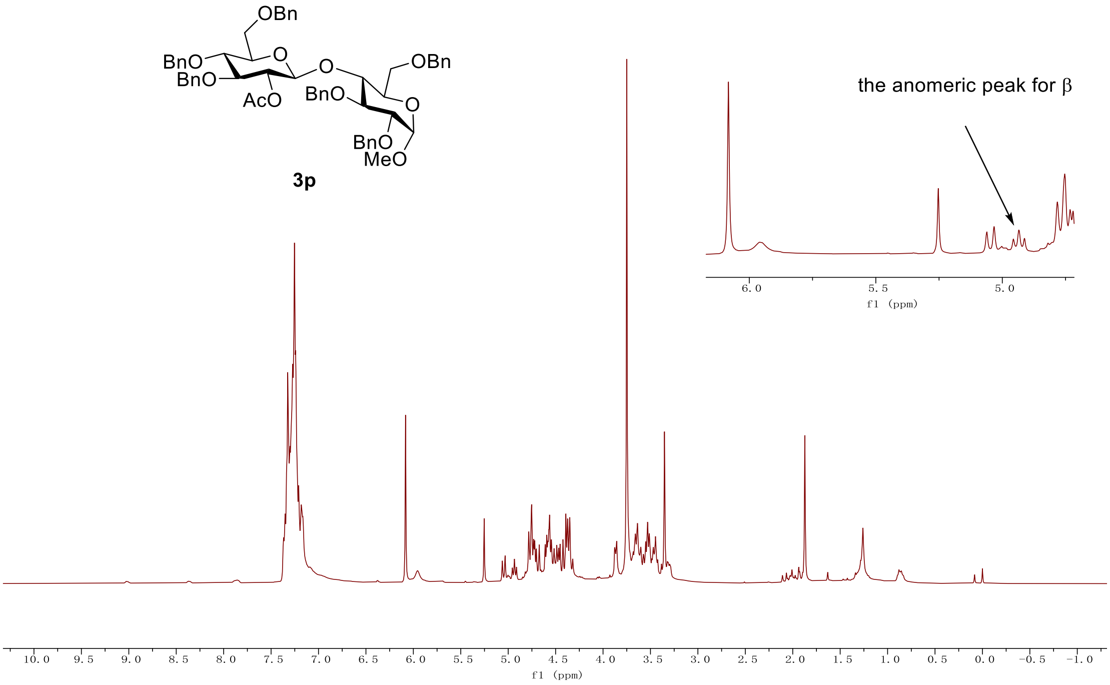


**Supplementary Figure S233. $^{1}$H NMR (400 MHz, $CDCl_3$) Spectra for crude compound 3p**

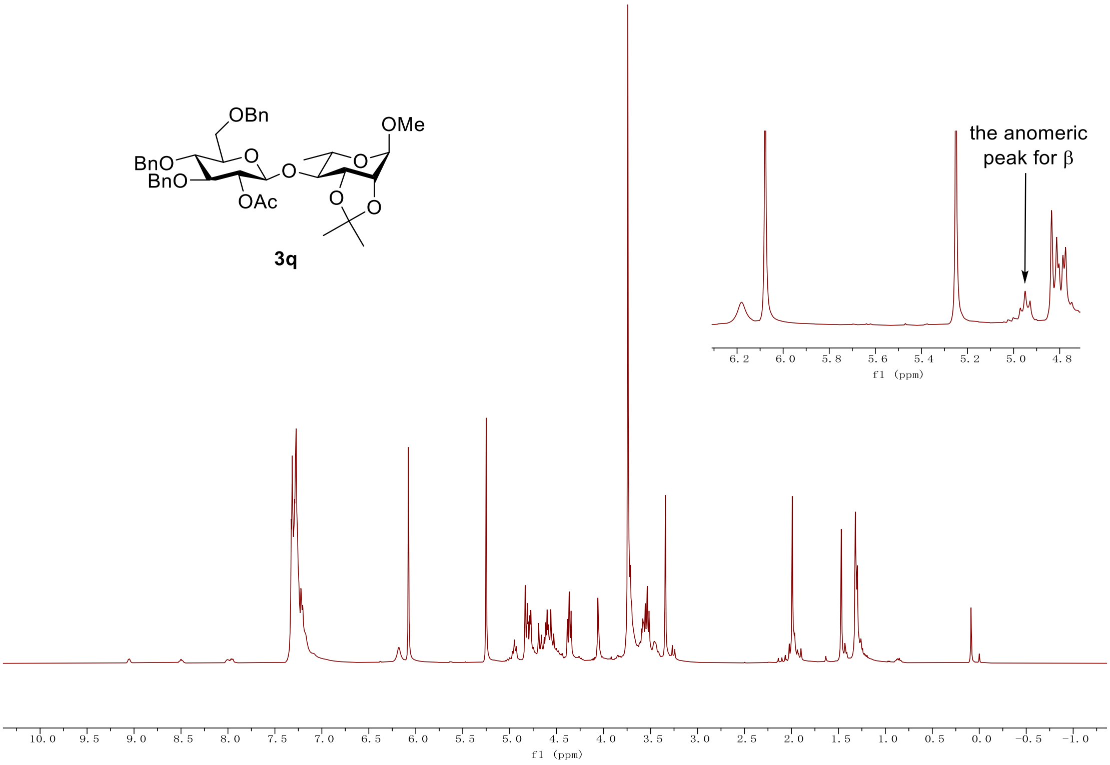


**Supplementary Figure S234. $^{1}$H NMR (400 MHz, $CDCl_3$) Spectra for crude compound 3q**

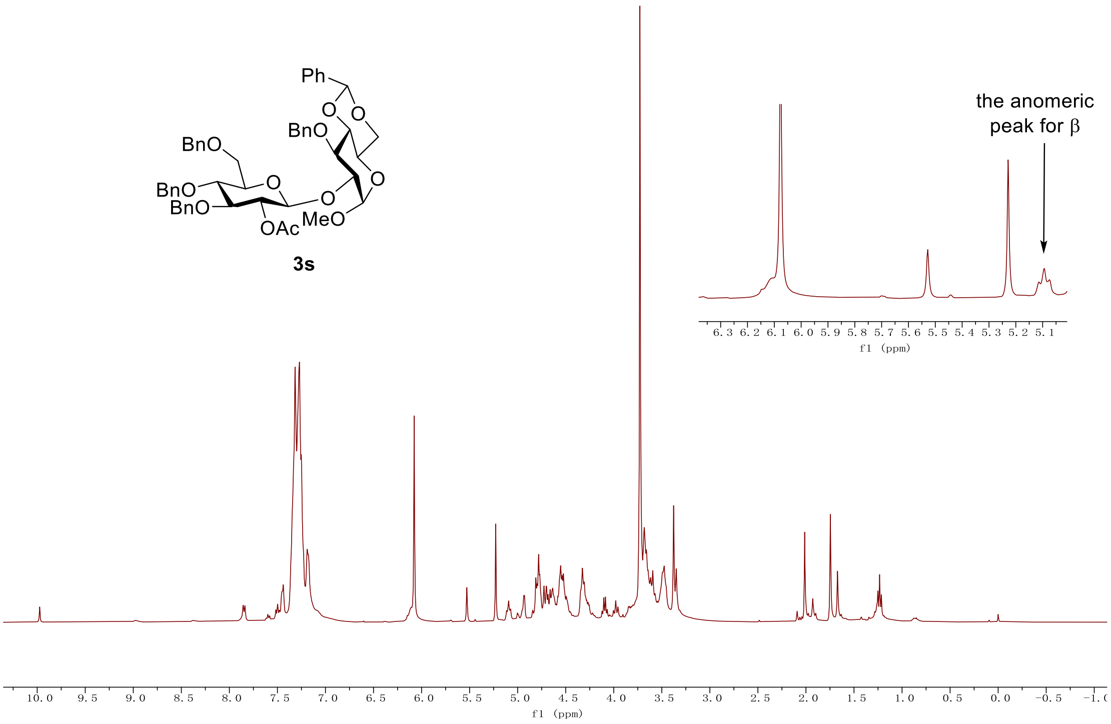


**Supplementary Figure S235. $^{1}$H NMR (400 MHz, $CDCl_3$) Spectra for crude compound 3s**

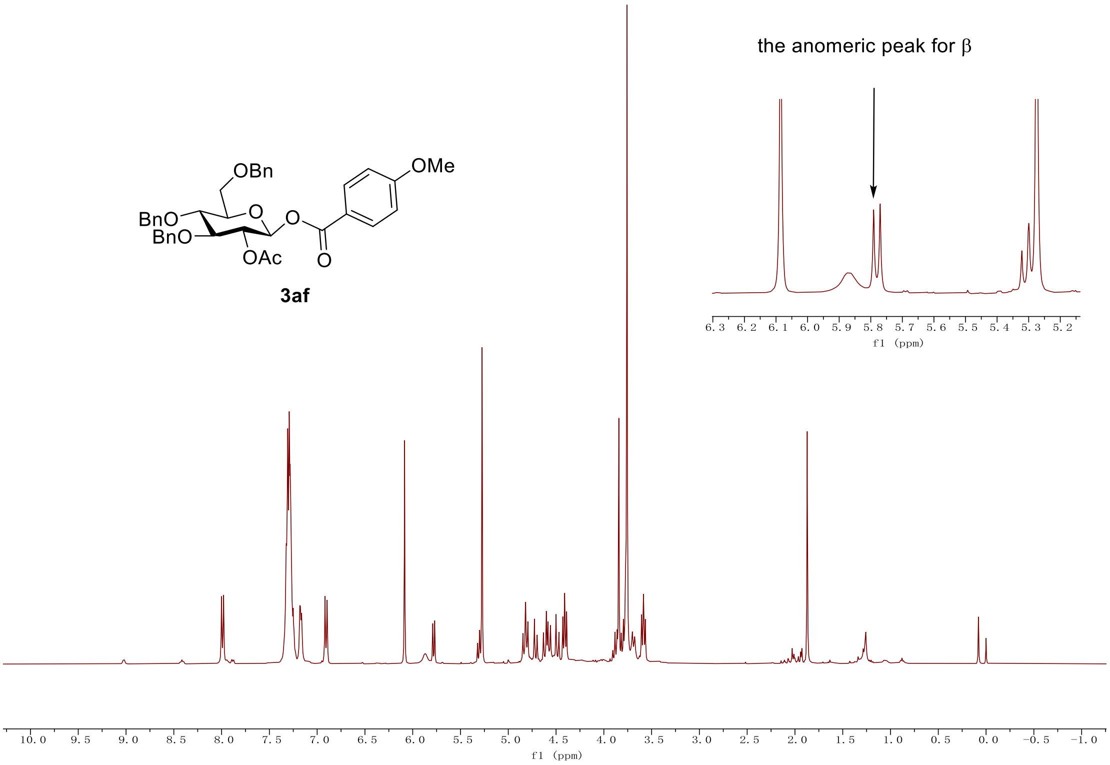


**Supplementary Figure S236. $^{1}$H NMR (400 MHz, $CDCl_3$) Spectra for crude compound 3af**

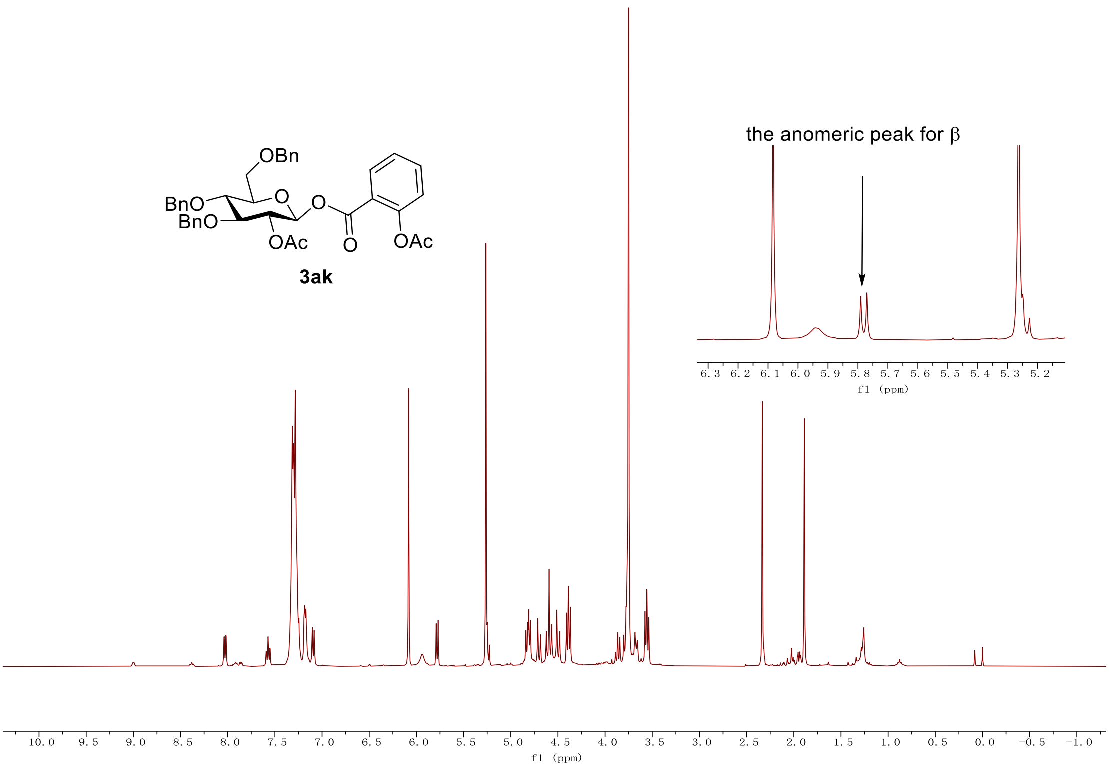


**Supplementary Figure S237. $^1$H NMR (400 MHz, $CDCl_3$) Spectra for crude compound 3ak**

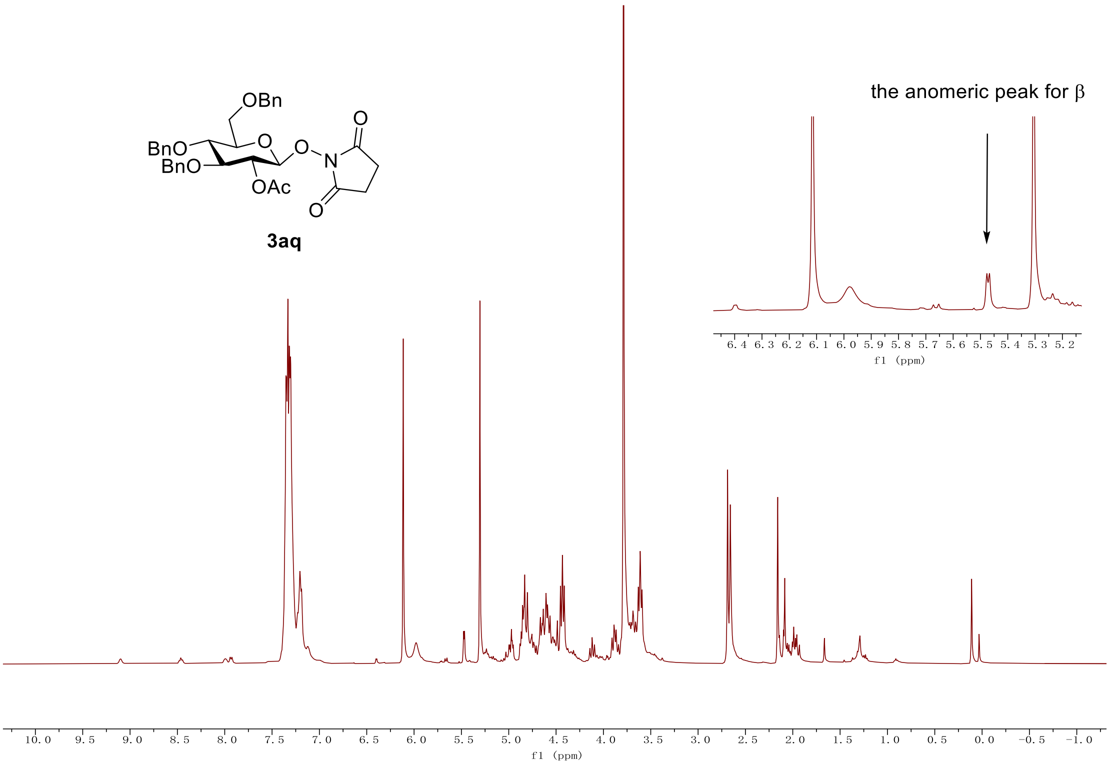


**Supplementary Figure S238. $^1$H NMR (400 MHz, $CDCl_3$) Spectra for crude compound 3aq**

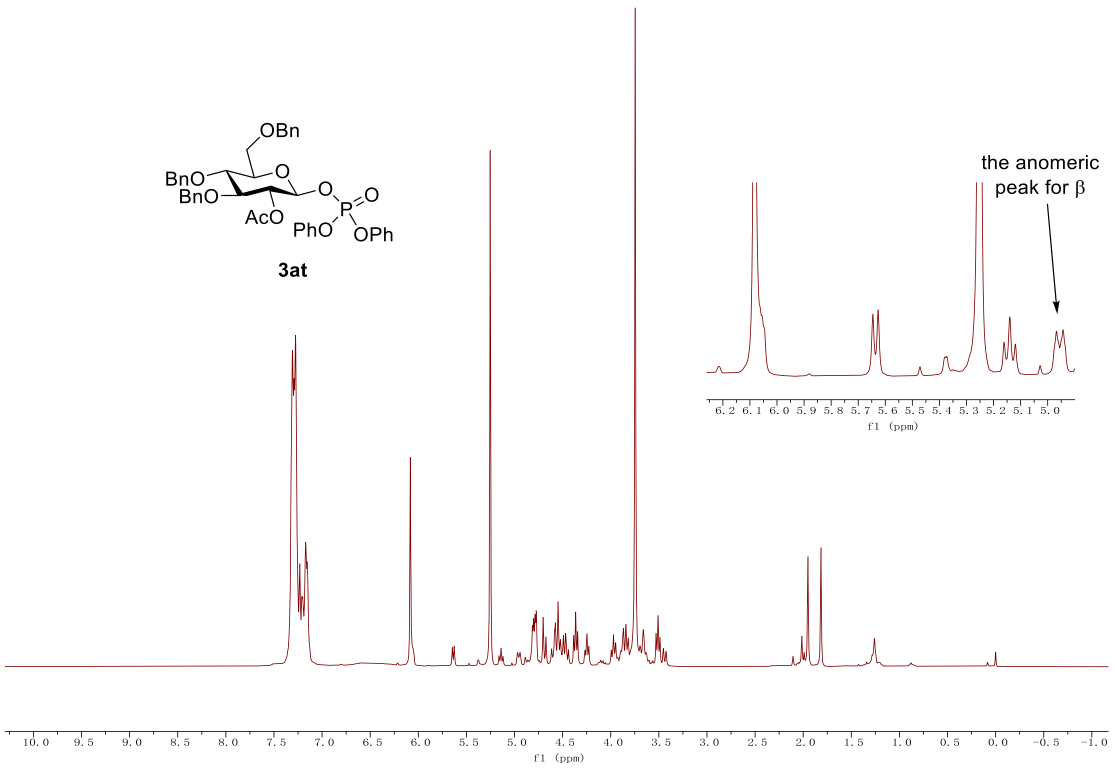


**Supplementary Figure S239. $^1$H NMR (400 MHz, $CDCl_3$) Spectra for crude compound 3at**

## 12.References

[1] Tiwari, V. K.; Kumar, A.; Schmidt, R. R. Disaccharide-Containing Macrocycles by Click Chemistry and Intramolecular Glycosylation. *Eur. J. Org. Chem.* **2012**, *15*, 2945-2956.

[2] Zare, H.; Ghanbari, M. M.; Jamali, M.; Aboodi, A. A novel and efficient strategy for the synthesis of various carbamates using carbamoyl chlorides under solvent-free and grinding conditions using microwave irradiation. *Chin. Chem. Lett.* **2012**, *23*, 883.

[3] Barroso, S.; Geerdink, D.; Horst, B.; Casas-Arce, E.; Minnaard, A. J. Total Synthesis of the Phenolic Glycolipid Mycoside B and the Glycosylated p-Hydroxybenzoic Acid Methyl Ester HBAD-I, Virulence Markers of Mycobacterium tuberculosis. *Eur. J. Org. Chem.* **2013**, 4642.

[4] Hanessian, S.; Banoub, J. Chemistry of the glycosidic linkage. A rapid and efficient synthesis of carbohydrate 1,2-orthoesters. *Carbohydr. Res.* **1975**, *44*, C14.

[5] (a) Sadeh, S.; Warren, C. D.; Jeanloz, R. W. 2-*O*-Acetyl-3,4-di-*O*-benzyl-6-*O*-(*tert*-butyldiphenylsilyl)-d-glucopyranosyl chloride as a d-glycosyl donor. *Carbohydr. Res.* **1983**, *123*, 73. (b) Wang, D.; Xiong, D-C.; Ye, X-Y. A five-component one-pot synthesis of phosphatidylinositol pentamannoside (PIM5). *Chin. Chem. Lett.* **2018**, *29*, 1340.

[6] Indurugalla, D.; Bennet, A. J. A Kinetic Isotope Effect Study on the Hydrolysis Reactions of Methyl Xylopyranosides and Methyl 5-Thioxylopyranosides: Oxygen versus Sulfur Stabilization of Carbenium Ions. *J. Am. Chem. Soc.* **2001**, *123*, 10889.

[7] Yu, D.; Lu, L.; Shen, Q. Palladium-Catalyzed Coupling of Polyfluorinated Arenes with Heteroarenes via C-F/C-H Activation. *Org. Lett.* **2013**, *15*, 940.

[8] Balmond, E. I.; Coe, D. M.; Galan, M. C.; McGarrigle, E. M. α-Selective Organocatalytic Synthesis of 2-Deoxygalactosides. *Angew. Chem. Int. Ed.* **2012**, *51*, 9152.

[9] Elchert, B.; Li, J.; Wang, J. H.; Hui, Y.; Rai, R.; Ptak, R.; Ward, P.; Takemoto, J. Y.; Bensaci, M.; Chang, C-W. T. Application of the Synthetic Aminosugars for Glycodiversification: Synthesis and Antimicrobial Studies of Pyranmycin. *J. Org. Chem.* **2004**, *69*, 1513.

[10] Yousefi, R.; Paul-Gorsline, B. J.; Soltani, O.; Ashtekar, K. D. An Alternative Route to the Anticancer Agent: 2-Fluorofucose from Readily Available L-(-)Rhamnose and Mechanistic Insights into a Zinc/Ammonium Iodide-Mediated Elimination Reaction. *Org. process. Res.* **2022**, *26*, 2475.

[11] Jayakanthan, K.; Vankar, Y. D. Glycosyl trichloroacetylcarbamate: a new glycosyl donor for O-glycosylation. *Carbohydr. Res.* **2005**, *340*, 2688.

[12] Zhang, Y.; Xiang, G.; He, S.; Hu, Y.; Liu, Y.; Xu, L.; Xiao, G. Orthogonal One-Pot

Synthesis of Oligosaccharides Based on Glycosyl ortho-Alkynylbenzoates. *Org. Lett.* **2019**, *21*, 2335.

[13] Li, P.; He, H.; Zhang, Y.; Yang, R.; Xu, L.; Chen, Z.; Huang, Y.; Bao, L.; Xiao, G. Glycosyl ortho-(1-phenylvinyl)benzoates versatile glycosyl donors for highly efficient synthesis of both O-glycosides and nucleosides. *Nat. Commun.* **2020**, *11*, 405.

[14] Wang, Y-H.; Yeh, H-W.; Wang, H-W.; Yu, C-C.; Guh, J-H.; Liu, D-Z.; Liang, P-H. Synthesis of a chlorogenin glycoside library using an orthogonal protecting group strategy. *Carbohydr. Res.* **2013**, *375*, 118.

[15] France, R. R.; Compton, R. G.; Davis, B. G.; Fairbanks, A. J.; Rees, N. V.; Wadhawan, J. D. Selective electrochemical glycosylation by reactivity tuning. *Org. Bio. Chem.* **2004**, *2*, 2195.

[16] Nukada, T.; Berces, A.; Zgierski, M. Z.; Whitfield, D. M. Exploring the Mechanism of Neighboring Group Assisted Glycosylation Reactions. *J. Am. Chem. Soc.* **1998**, *120*, 13291-13295.

[17] Premathilake, H. D.; Mydock, L. K.; Demchenko, A. V. Superarming Common Glycosyl Donors by Simple 2-*O*-Benzoyl-3,4,6-tri-*O*-benzyl Protection. *J. Org. Chem.* **2009**, *75*, 1095-1100.
